# Sorafenib promotes hepatocellular carcinoma invasion *via* interleukin-6/HIF-1α/PFKFB3

**DOI:** 10.7150/jca.84451

**Published:** 2023-06-26

**Authors:** Pengyuan Zhuang, Dong Wang, Kewei Zhang, Jiandong Wang, Jun Shen

**Affiliations:** 1 Department of General Surgery, Xinhua Hospital, School of Medicine, Shanghai Jiao Tong University, Shanghai, 200092, China.; 2 Shanghai Key Laboratory of Biliary Tract Disease Research, Shanghai, 200092, China.; 3 Shanghai Research Center of Biliary Tract Disease, Shanghai, 200092, China.; 4 Department of General Surgery, Tongren Hospital, Shanghai Jiao Tong University School of Medicine, Shanghai, 200050, China.

**Keywords:** hepatocellular carcinoma, sorafenib, IL-6, PFKFB3, invasion

## Abstract

**Background:** Although sorafenib is adopted as the first-line treatment for unresectable liver cancer, the antitumor efficacy is severely limited by the pro-invasive side effect.

**Methods:** To explore the underlying mechanisms, various-dosage sorafenib was applied to survey its effect on cell invasion in HCCLM3 and PLC cell models.

**Results:** Our results revealed that high-dosage sorafenib inhibited liver cancer cell invasion. By contrast, sorafenib with low and median dosages promoted the invasion. *In vivo* studies showed that sorafenib with a median dosage increased the intrahepatic metastasis (IHM) and lung metastasis (LM) of liver cancer cells, while sorafenib with a high dosage inhibited IHM and LM. Then, bioinformatics analysis indicated that HIF-1α, IL-6, and PFKFB3 were associated with the sorafenib resistance. *In vitro* models showed that the pro-invasive effect was mediated by IL-6/HIF-1α/PFKFB3 regulation in dosage- and time-dependent manners. PFKFB3 knockdown confirmed that PFKFB3 promoted HCCLM3 cell migration via modulating EMT-related markers. Furthermore, we found that sorafenib upregulated PFKFB3 by IL-6/HIF-1α in a time-dependent manner, without direct effect on PFKFB3 expression.

**Conclusions:** In summary, these results demonstrated that sorafenib could dose-dependently promote cell invasion, intrahepatic and lung metastasis in hepatocellular carcinoma through IL-6/HIF-1α mediated PFKFB3 activation, providing novel insights to improve the therapeutic efficacy of sorafenib.

## Introduction

Hepatocellular carcinoma (HCC) reached about 841,000 new cases and was associated with 782,000 deaths in 2018^1^. About more than 50% of the cases occur in China^2^-^4^. Patients eligible for surgical resection are those with early HCC and normal liver function and have no serious complications and comorbidities^5^. After surgical resection, the recurrence rate at 5 years is approximately 70%, and two-thirds of the recurrence occurs within 2 years^6^. Therefore, treatment of patients with liver cancer needs further improvement.

Sorafenib is an important therapy for advanced liver cancer but has side effects, such as hyper-progress of tumor after discontinuation and promotion of tumor invasion and migration in cell and animal studies. Therefore, underlying mechanism should be further studied, and new therapies for liver cancer should be discovered to avoid the pro-invasive side effect^7^-^9^. Interleukin-6 (IL-6) is a multifunctional cytokine that participates in multiple biological activities, such as cell proliferation, invasion, and angiogenesis^10^; it is also involved in epithelial-mesenchymal transformation (EMT) and promotes cell metastasis in HCC, which is correlated with the upregulation of IL‑6 expression. Although IL-6 is involved in sorafenib-induced pro-invasion, the underlying mechanism has not been completely understood.

Under oxygen-rich conditions, cancer cells tend to metabolize glucose by using less efficient glycolysis and produce much lactic acid in a unique metabolic change process known as the Warburg effect^11^, ^12^. Aerobic glycolysis, as one of the features of tumor metabolic reprogramming, not only rapidly generates adenosine triphosphate (ATP) to support tumor cells to survive under energy stress but also provides intermediate metabolites to synthesize nucleic acids, proteins, and lipids, which are necessary for cell proliferation^13^. The acidic tumor microenvironment induced by tumor glycolysis could affect the activities of immune cells, resulting in immune escape and tumor progression^14^. Aerobic glycolysis is the active choice of tumor cells compared with anaerobic glycolysis. In the body, PFK2 has four isoenzymes including PFKFB1-4^15^. Among the four isoenzymes, PFKFB3 has higher expression in many tumors, such as breast cancer, HCC, colon cancer, and high-grade astrocytoma^16^-^19^. More importantly, PFKFB3 overexpression can predict poor prognosis in HCC patients, indicating that PFKFB3 could be a prognostic factor for HCC^19^. However, limited research has focused on whether PFKFB3 can affect cell invasion in HCC. The present study aims to further discover the exact role and potential mechanism of PFKFB3.

Tumor hypoxia could induce aerobic glycolysis, in which hypoxia-inducing factor-1α (HIF-1α) plays a significant role. PFKFB3 is a hypoxia-inducing gene stimulated by the interaction of a consistent hypoxia-responsive element site in HIF-1α and its promoter region. However, the exact role of regulation of HIF-1α/PFKFB3 in the pro-invasion effect induced by sorafenib in hepatocellular carcinoma needs further investigation. Whether IL-6 mediates pro-invasion under sorafenib treatment by regulating aerobic glycolysis and the underlying molecular mechanism should be uncovered.

## Materials and methods

### Cells and drugs

Hep-G2, HuH-7, and PLC cell lines were obtained from the Type Culture Collection Cell Bank of the Chinese Academy of Sciences, Shanghai, China, and HCC-LM3 and L-02 cell lines were acquired from the Liver Cancer Institute, Fudan University, Shanghai, China. These cells are commonly used in liver cancer research. L-02 is a normal liver cell, and the four other types are hepatocellular carcinoma cells. HCC-LM3 is an HCC cell line with highly invasive lung metastasis. Sorafenib (Bayer Ag) and recombinant human IL‑6 (R&D Systems, Inc.) were administered as in the previous study^20^.

### Western blot analysis

Cell lines were harvested and lysed for Western blot analysis. The primary antibodies used were as follows: IL-6 (NB600‑1131; Novus Biologicals, 1:2000), HIF-1α (ab51608, Abcam, 1:1000), PFKFB3 (#13123S, CST, 1:1000), N-cadherin (#13116T, CST, working dilution 1:1000), HIF-1E-cadherin (#3195T, CST, working dilution 1:1000), Snail (#3879T, CST, 1:1000), vimentin (#5741T, CST, 1:1000), and β-actin (#8457T, CST, 1:1000).

### Quantitative RT-PCR assay

Total RNA was extracted according to the manufacturer's instructions (Invitrogen). Real-time PCR was performed using TB Green®Premix Ex TaqTM II(#RR820A, TAKARA, Japan). Detailed information on the primer sequences is shown as follows: *PFKFB3*, forward, 5ʹ-AGCCCGGATTACAAAGACTGC-3ʹ and reverse, 5ʹ-GGTAGCTGGCTTCATAGCAAC-3ʹ; *GAPDH*, forward, 5ʹ-GACAGTCAGCCGCATCTTCT-3ʹ and reverse, 5 ʹ-GCGCCCAATACGACCAAATC-3ʹ.

### RNA interference

Si-Control and PFKFB3-specific siRNA were purchased from GenePharma. RFect siRNA/miRNA transfection reagent (#11012,BAIDAI) was used to transfect HCC cells at 37 ℃ for 48 h. The siRNA sequences are indicated below: RNAi-1, forward, 5ʹ-GGAAGGCGCUCAAUGAGAUTT-3ʹ and reverse, 5ʹ-AUCUCAUUGAGCGCCUUCCTT-3ʹ; RNAi-2, forward, 5'-UCUUCACACCGUCCUGAAATT-3ʹ and reverse, 5ʹ-UUUCAGGACGGUGUGAAGATT-3ʹ; RNAi-3, forward, 5ʹ-GCCUCGCAUCAACAGCUUUTT-3ʹ and reverse, 5ʹ-AAAGCUGUUGAUGCGAGGCTT-3ʹ.

### CCK8 assay

CCK-8 assay kit was used in the study. Transfected HCC cells were cultured at different times (0, 24, 48, and 72 h). The absorbance of each well was recorded using a microplate reader (Bio-Tek).

### Colony formation assay

A total of 1,000 transfected HCC cells with si-Control or si-PFKFB3 were seeded in six-well plates. Colonies were then fixed in 4% paraformaldehyde (#P0099, Beyotime). Colony numbers were then counted.

### Cell migration assay at room temperature

Cell migration was evaluated using an 8 μm Transwell cavity (#3422, Corning). In total, 50,000 HCC cells were added to the upper chamber. After being incubated under different conditions using sorafenib or a vehicle, cells on the bottom chamber were collected. The migrated cells were counted under a light microscope.

### Cell culture and hypoxia

Cobalt chloride (CoCl_2_, 100 μmol/L) was adopted to mimic hypoxia with its minimal effect on cell viability. The effects of hypoxia on PFKFB3 expression in HCCLM3 cells were determined in the presence or absence of CoCl_2_.

### Animals, treatment in vivo, intrahepatic metastasis, and lung metastasis

Male BALB/c nu/nu nude mice, weighing approximately 20 g (Shanghai Institute of Materia Medica, Chinese Academy of Sciences, Shanghai, PR China), were housed in laminar flow cabinet under specific pathogen-free condition and used at the age of 6 wk. The mice were cared for and handled in accordance with the National Institutes of Health Guidelines for the Care and Use of Laboratory Animals. The experimental protocol was approved by the Shanghai Medical Experimental Animal Care Committee. The HCC tumor model was established by the orthotropic implantation of a histologically intact tumor tissue derived from HCCLM3-wt and HCCLM3-PFKFB3(-) cell lines. When the average tumor volume had reached 100 mm3 (first week of tumor implantation), sorafenib (6 mice, 20 mg/kg; 6 mice 40mg/kg), and normal saline (6 mice, NS, as control group) were injected subcutaneously every day for six wks to observe their effects on tumor growth. The tumor size was assessed using the formula width × length × depth × π/6.

All institutional and national guidelines for the care and use of laboratory animals were followed.

### Bioinformatics analysis

The mRNA expression profile dataset GSE109211 was downloaded from the GEO database (https://www.ncbi.nlm.nih.gov/geo/) consisting of 67 sorafenib-treated HCC patients, including 21 sorafenib responders and 46 non-responders. Data from GSE109211 were normalized by the “limma” R package, and the eBayes algorithm was used to identify differentially expressed genes (DEGs) between the responder and non-responder groups. RNA with |log2 fold change (FC)| >0.7 and P-value <0.05 were considered as DEGs. GO enrichment analysis of the activation pathways of DEGs was performed by the R package “clusterProfiler.” Enrichment score was obtained by single sample gene set enrichment analysis (ssGSEA) method. The RNA-seq mRNA expression profile data of the TCGA-LIHC cohort were downloaded from the TCGA database (https://portal.gdc.cancer.gov/). Based on TCGA transcriptomic data, the “pRRophetic” package was applied to assess sorafenib chemosensitivity and presented in the form of half-maximal inhibitory concentration (IC_50_).

### Statistical analysis

Data analysis was performed with SPSS 18.0 (SPSS Inc.). Two-tailed Student t test was used for comparison. *P* < 0.05 was considered to be statistically significant.

## Results

### Side pro-invasive effect induced by sorafenib in dosage- and time-specific manner

**Figure 1:**
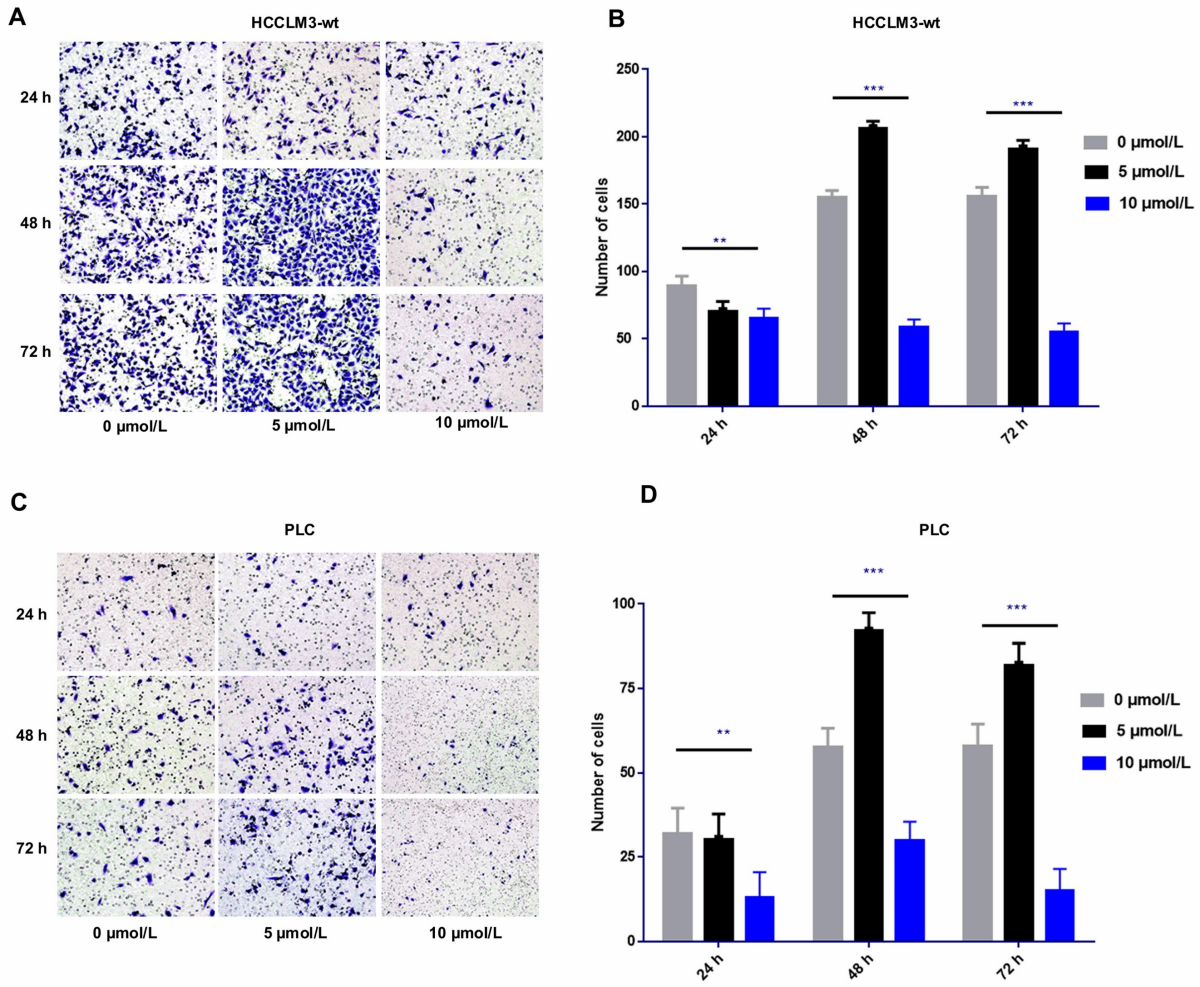
Effect of sorafenib on cell invasion in time- and dosage-specific manner.** (A-D)** Transwell assay revealed that low and median dosages of sorafenib (5 μmol/L) inhibited cell invasion within 24 h of treatment and increased the cell invasion at 48 and 72 h after administration. High dosage of sorafenib (10 μmol/L) inhibited cell invasion from 24 h to 72 h. (**A-B**: HCCLM3; **C-D**: PLC; *** *P* < 0.001; ** P < 0.01)

The transwell cell migration assay revealed that high-dose sorafenib (10 µmol/L) inhibited cell invasion in HCCLM3-wt cells and PLC cells from 24 h to 72 h. Administration of low and median dosages of sorafenib (2.5-7.5 µmol/L) for 24 h inhibited the invasion of HCCLM3-wt cells and PLC cells but promote the cell invasion of both cell lines after treatment for 48 and 72 h (**Fig. 1**).

### HIF-1α, IL-6, and PFKFB3 associated with sorafenib resistance

**Figure 2:**
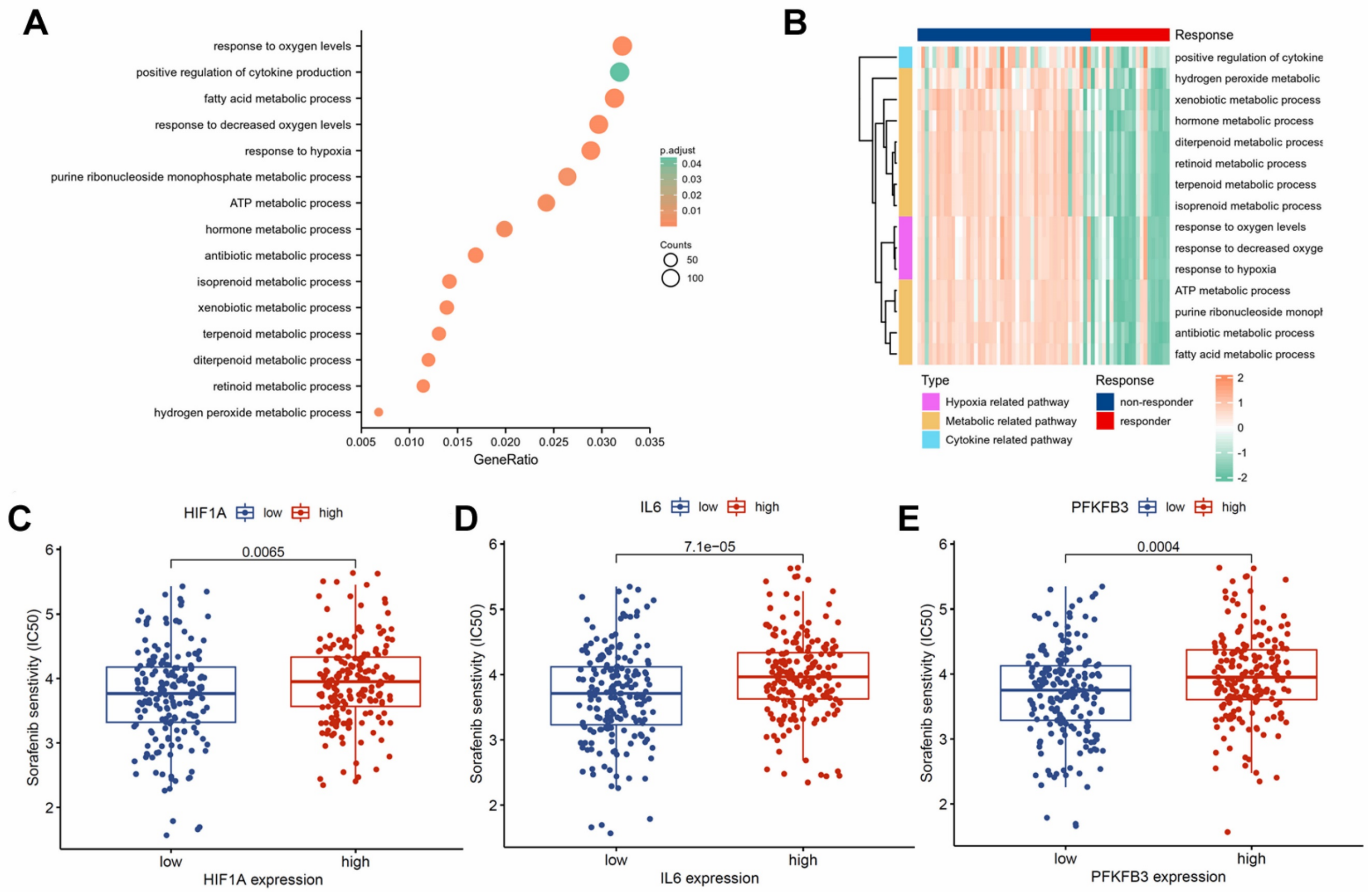
Association of HIF-1α, IL-6, and PFKFB3 with sorafenib resistance. GO enrichment analysis of DEGs (**A**). Cytokine, hypoxia, and metabolism-related pathways were significantly activated in the sorafenib non-responder group (**B**). Group with lower expression of HIF1A (**C**), IL6 (**D**), and PFKFB3 (**E**) showed higher sensitivity to sorafenib.

To investigate the molecular mechanisms of sorafenib resistance in HCC, we performed a differential analysis on the GSE109211 dataset. We identified 2,437 significantly upregulated genes and 2,377 significantly downregulated genes in the sorafenib response group compared with those in the sorafenib non-response group (**Supplementary Table 1**). The GO enrichment analysis highlighted the dysregulation of several signaling pathways associated with drug resistance (**Supplementary Table 2**), including positive regulation of cytokine production, response to hypoxia, and various metabolism-related pathways (**Fig. 2A**). Hypoxia, metabolic, and cytokine-related pathways were activated in the non-responsive group (**Fig**. **2B**). Drug sensitivity prediction based on the TCGA dataset showed that HIF1A (**Fig. 2C**), IL6 (**Fig. 2D**), and PFKFB3 (**Fig. 2E**) in the low-expression groups had higher sensitivity to sorafenib. Hence, HIF-1α, IL-6, and PFKFB3 may be associated with sorafenib resistance.

### Abnormally high expression of PFKFB3 in HCC cells

**Figure 3:**
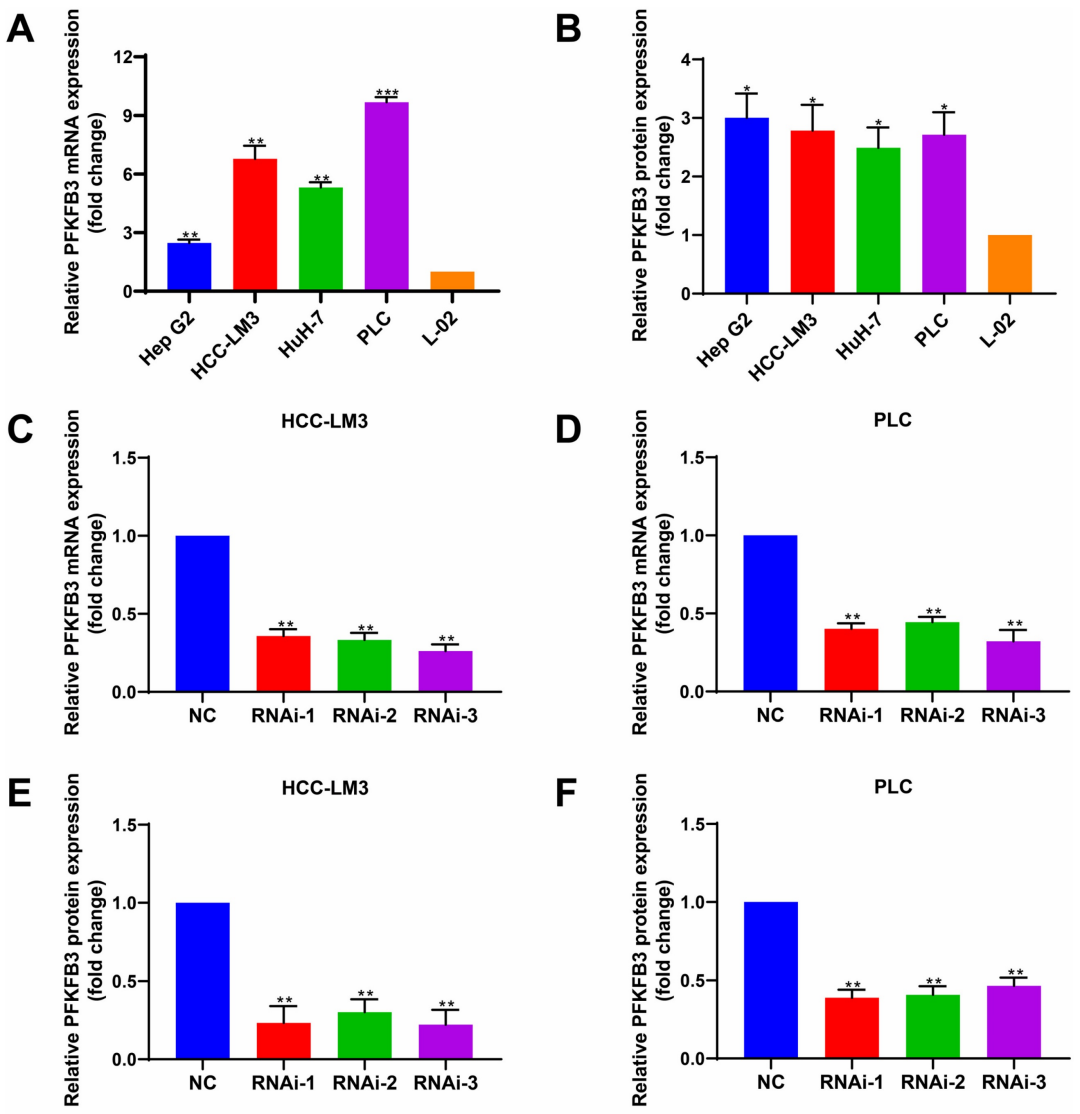
HCC cells expressed higher levels of PFKFB3 than hepatocytes. **(A)** and** (B)** RT-PCR and Western blot analysis of PFKFB3 expression in Hep G2, HCC-LM3, HuH-7, PLC, and L-02 cells. **(C-F)** PFKFB3 expression in HCC-LM3 and PLC cells infected with si-Control or si-PFKFB3 for 48 h confirmed through RT-PCR and Western blot analysis. Data are presented as normalized mean ± SD. * *P* < 0.05; ** *P* < 0.01; *** *P* < 0.001.

To determine the expression level of PFKFB3 in different HCC cells and hepatocytes under normal oxygen, we performed RT-PCR (**Fig. 3A**) and Western blot analyses (**Fig. 3B**). PFKFB3 was expressed in liver cancer cells and liver cells. HCC cells (Hep G2, HCC-LM3, HuH-7, and PLC) expressed higher levels of PFKFB3 than hepatocytes (L-02). The mRNA expression (**Fig. 3A**) from high to low was as follows: PLC (9.68-fold, *P* = 0.0003), HCC-LM3 (6.78-fold, *P* = 0.0044), HuH-7 (5.31-fold, *P* = 0.0013), and Hep G2 (2.47-fold, *P* = 0.0049). The protein level (**Fig. 3B**) from high to low was as follows: Hep G2 (3.00-fold, *P* = 0.0140), HCC-LM3 (2.79-fold, *P* = 0.0195), PLC (2.71-fold, *P* = 0.0165), and HuH-7 (2.49-fold, *P* = 0.0175). Therefore, PFKFB3 has overexpression in HCC cells and might promote its progression.

### Knockdown of PFKFB3 inhibited cell proliferation and migration in vitro

**Figure 4:**
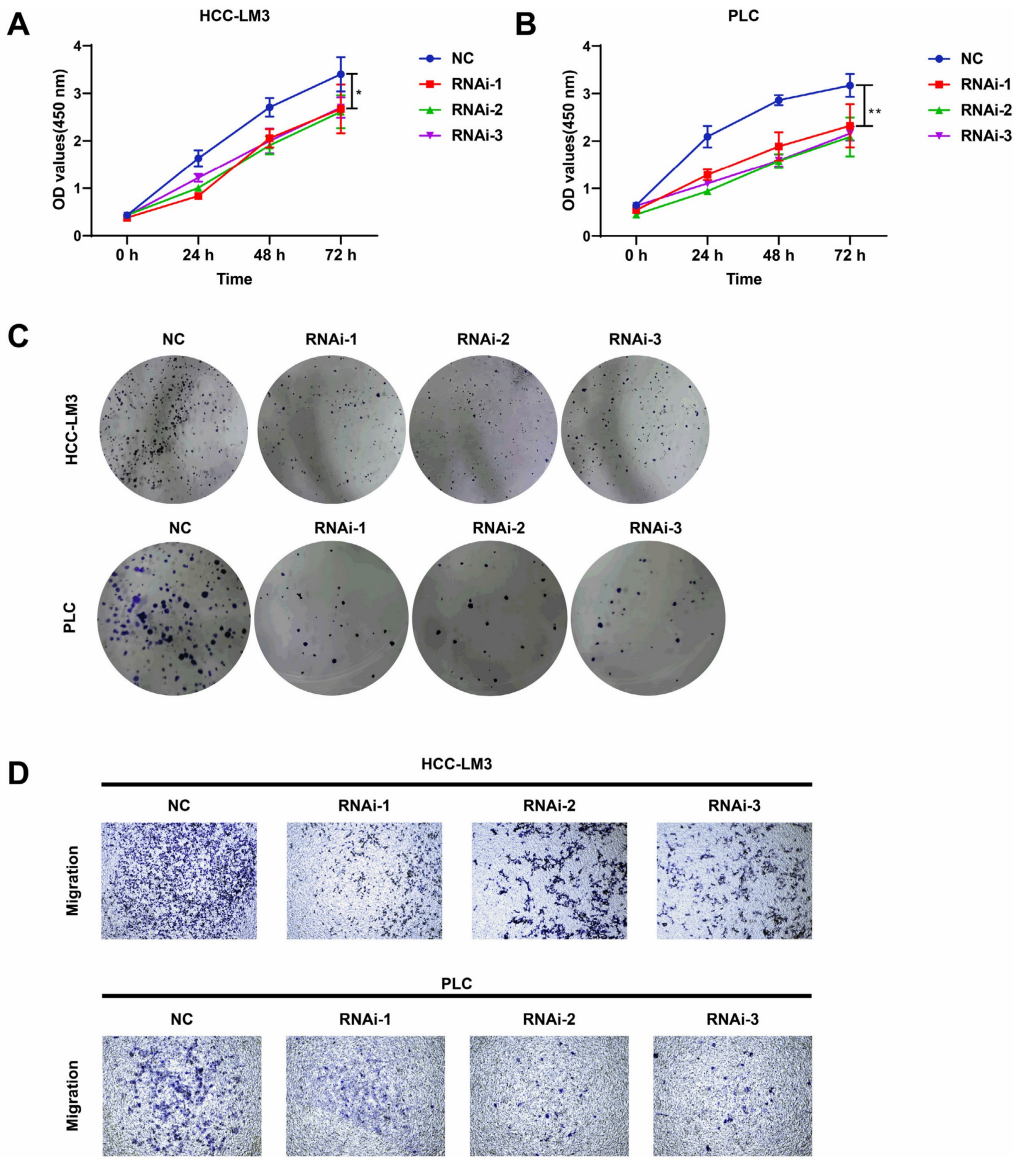
PFKFB3 knockdown suppressed the aggressive behavior of HCC cells in vitro. **(A-D)** HCC-LM3 and PLC cells were infected with si-Control or si-PFKFB3. The cells were harvested for CCK8 **(A-B)**, colony formation **(C)**, and Transwell migration assays** (D)** after 48 h of culture. Each RNAi group was compared with the control group. Data are presented as mean ± SD. * *P* < 0.05; ** *P* < 0.01; *** *P* < 0.001.

PFKFB3 was knocked down to explore the biological role of PFKFB3 in HCC, HCC-LM3, and PLC cells (**Fig. 3C-F**). CCK8 assay (**Fig. 4A-B**) showed that after 72 h of culture, the OD values (450 nm) of HCC-LM3 and PLC in the RNAi group were significantly reduced compared with those in the NC group (*P* < 0.05 in 72 h). Colony formation assay (**Fig. 4C**) displayed the lower number of colonies of HCC-LM3 and PLC in the RNAi groups than that in the NC group after two weeks of incubation (59 ± 9, 46 ± 12, 58 ± 8 *vs.* 138 ± 17 for RNAi-1, RNAi-2, RNAi-3 *vs.* control in HCC-LM3, *P* = 0.0020, 0.0014, 0.0017 respectively; 31 ± 7, 35 ± 10, 35 ± 5 *vs.* 126 ± 20 for RNAi-1, RNAi-2, RNAi-3 *vs.* control in PLC, *P* = 0.0014, 0.0019, 0.0014 respectively). The above data proved that PFKFB3 inhibition significantly impaired the proliferation of HCC cells. Meanwhile, transwell assay (**Fig. 4D**) revealed that PFKFB3 inhibition decreased the metastatic ability of HCC cells. After 48 h of incubation, the transmembrane cell numbers of HCC-LM3 and PLC in the three RNAi groups *vs.* the NC group were 58 ± 18, 83 ± 24, 84 ± 13 *vs.* 154 ± 20 for HCC-LM3 (*P* = 0.0001, 0.0020, 0.0006) and 26 ± 7, 21 ± 8, 26 ± 7 *vs.* 57 ± 8 for PLC (*P* = 0.0074, 0.0050, 0.0070).

### PFKFB3 facilitated HCC cell migration by affecting the expression of EMT-related proteins

**Figure 5:**
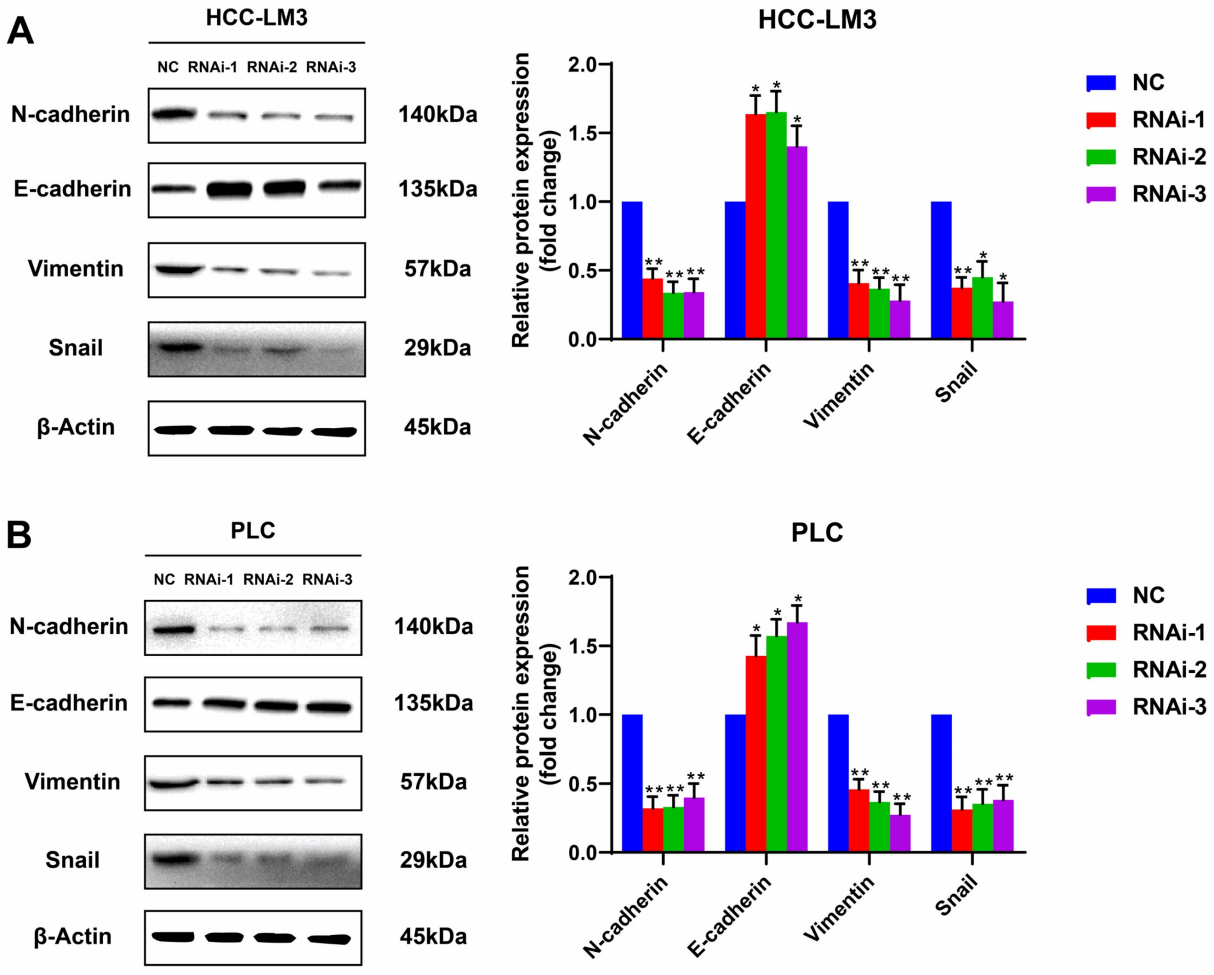
PFKFB3 improved the metastatic ability of HCC cells through EMT-related proteins. HCC-LM3 cells (**A**) and PLC (**B**) infected with si-Control or si-PFKFB3 were harvested for Western blot analysis after 48 h of culture. Each RNAi group was compared with the control group. (* *P* < 0.05; **, *P* < 0.01; *** P < 0.001).

Silencing PFKFB3 could decrease the proliferation and migration of HCC-LM3 cells and PLC. Potential proteins associated with these effects are worthy of exploration. Western blot analysis showed that E-cadherin, the protein marker of EMT, was significantly upregulated in PFKFB3-silenced HCC-LM3 and PLC; by contrast, N-cadherin, vimentin, and Snail were downregulated. These results suggested that PFKFB3 promoted the migration of HCC cells through EMT-related proteins (**Fig. 5**).

### Upregulation of IL‑6 by sorafenib is dosage specific

**Figure 6:**
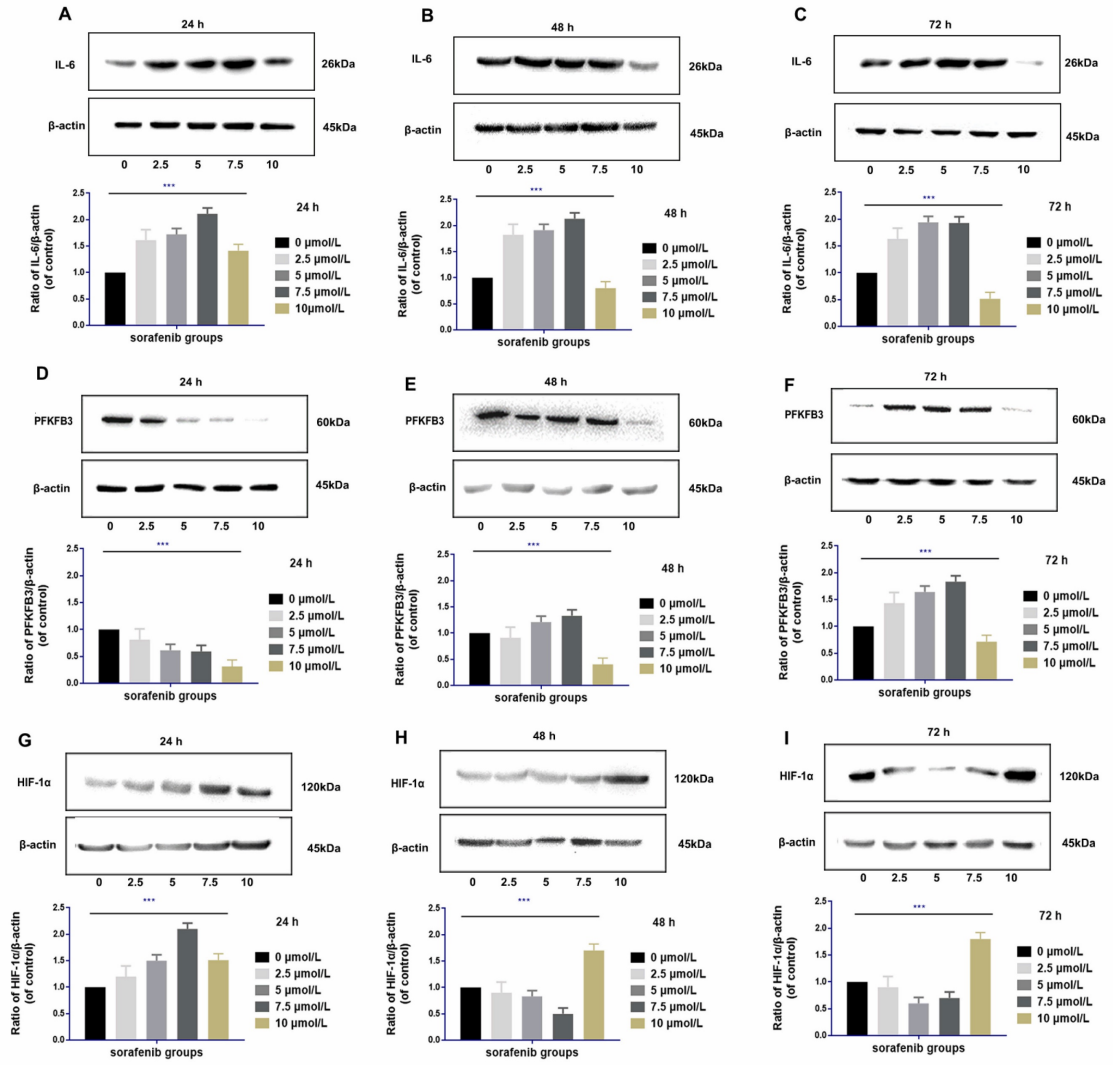
Regulation of IL-6 by sorafenib was dosage and time specific **(A-C)**. Low and median dosages of sorafenib (2.5-7.5 µmol/L) upregulated IL-6 expression after 24 h to 72 h of administration in HCCLM3‑wt cells; high dosage of sorafenib (10 µmol/L) upregulated IL-6 expression in 24 h but downregulated IL-6 expression from 48 h to 72 h of administration (Western blot analysis, *** *P* < 0.001); Regulation of PFKFB3 by sorafenib is dosage and time specific** (D-F)**. Low and median dosages of sorafenib (2.5-7.5 µmol/L) downregulated PFKFB3 expression in 24 h but upregulated it from 48 h to 72 h. A high dosage of sorafenib (10 µmol/L) down-regulated PFKFB3 expression after 24 h to 72 h of administration in HCCLM3‑wt cells (Western blot analysis, *** *P* < 0.001); Regulation of HIF-1α by sorafenib is dosage and time specific **(G-I)**. Low and median dosage of sorafenib (2.5-7.5 µmol/L) upregulated HIF-1α expression in 24 h but downregulated it from 48 h to 72 h of administration. High dosage of sorafenib (10 µmol/L) up-regulated PFKFB3 expression after 24 h to 72 h of administration in HCCLM3‑wt cells (Western blot analysis, *** *P* < 0.001)

IL-6 was upregulated at 24 h to 72 h after sorafenib administration at moderate dosages (2.5-7.5 µmol/L) in HCCLM3‑wt cells. The degree of upregulation of IL-6 was positively correlated with the dosage of sorafenib within the moderate dosage range (rr = 0.721, *P* <0.01). Compared with the control group, high dosage of sorafenib (10 µmol/L) upregulated IL-6 expression within 24 h. However, from 48 h to 72 h, high dosages of sorafenib downregulated IL-6, and the decreased expression was the most prominent in 72 h (**Fig. 6A-C**).

### Regulation of PFKFB3 by sorafenib is dosage and time specific

The regulation of PFKFB3 by sorafenib was related to dosage and treatment time. PFKFB3 was significantly downregulated by moderate dosages of sorafenib (2.5-7.5 µmol/L) in 24 h. However, the expression of PFKFB3 was inversely upregulated within 48 and 72 h. Compared with the control group, high dosage of sorafenib (10 µmol/L) downregulated the expression of PFKFB3 throughout the treatment, and the downregulation effect was gradually weakened with prolonged treatment (**Fig. 6D-F**).

### Regulation of HIF-1α by sorafenib is dosage and time specific

High dosages of sorafenib (10 µmol/L) consistently upregulated HIF-1α at 24 h of treatment. Under prolonged treatment, HIF-1α expression was upregulated by high dosages of sorafenib at 48 and 72 h. After treatment with low and median dosages of sorafenib (2.5-7.5 µmol/L) for 24 h, HIF-1α was upregulated. However, HIF-1α expression was gradually downregulated at 48 h and 72 h of administration (**Fig. 6G-I**).

### Regulation of IL-6 on HIF-1α is time specific

**Figure 7:**
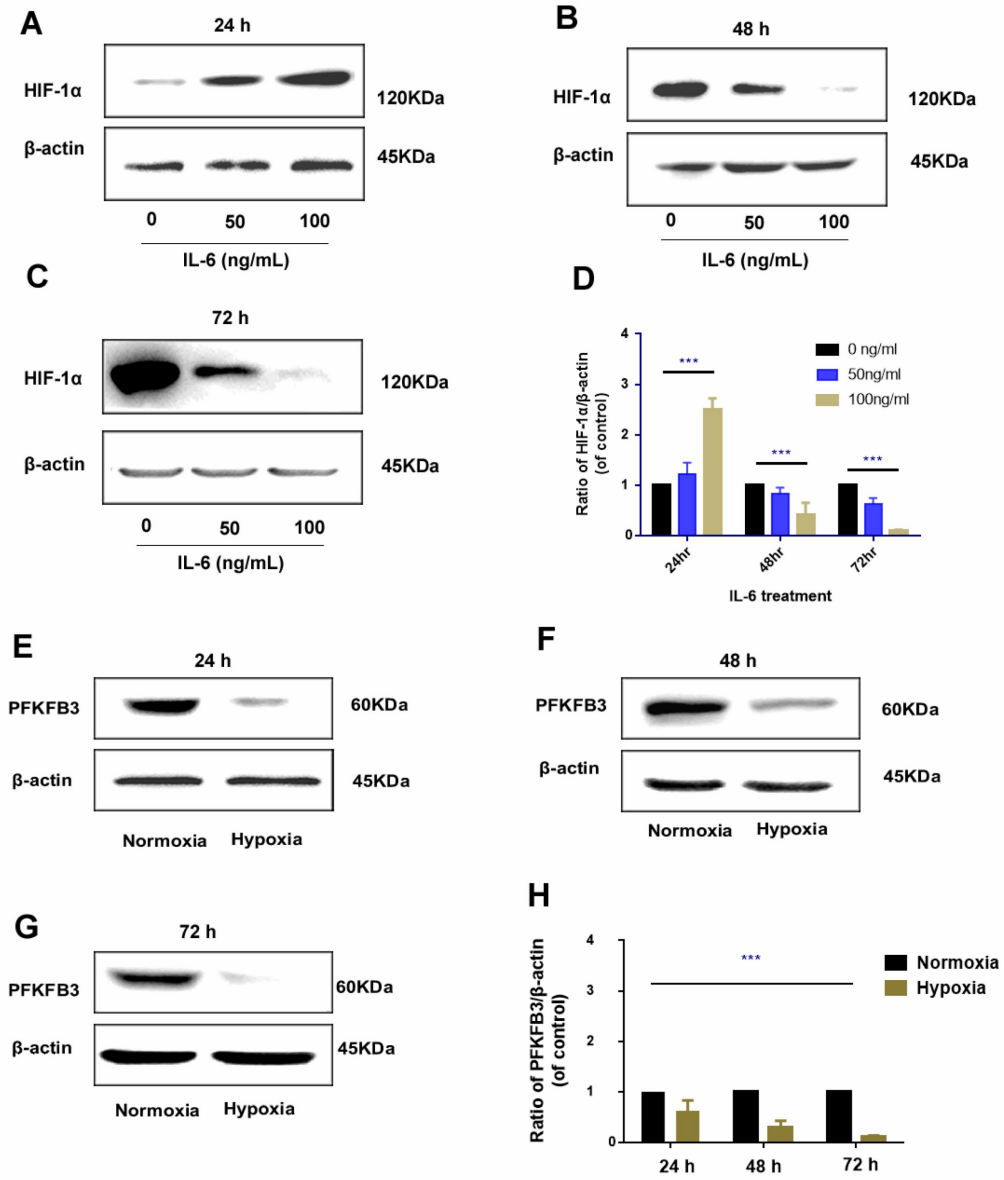
Time-specific regulation of IL-6 on HIF-1α** (A-D)**. In 24 h, HIF-1α was upregulated by exogenous administration of IL-6 and positively correlated with IL-6 dosage. However, in the 48 h and 72 h, HIF-1α was inversely downregulated by exogenous IL-6 and negatively correlated with IL-6 dosage (Western blot analysis, *** *P* < 0.001); negative regulation of hypoxia on PFKFB3 **(E-H)**. Hypoxia negatively inhibited PFKFB3 expression, and negative regulation was the most prominent 72 h under hypoxia condition (Western blot analysis, *** *P* < 0.001).

The regulatory role of IL-6 in HIF-1α was found to be time specific. After 24 h of administration, exogenous IL-6 upregulated HIF-1α expression, and the upregulation was positively correlated with the dosage of IL-6 (rr = 0.812, *P* < 0.01); as IL-6 was continued to be administrated for 48 h and 72 h, HIF-1α was gradually downregulated, which was more prominent in treatments with higher dosage of IL-6 (**Fig. 7A-D**).

### Negative regulation of hypoxia and PFKFB3

Hypoxia could promote HIF-1α upregulation; the regulatory pattern between hypoxia and PFKFB3 is negative feedback, that is, under hypoxia condition, PFKFB3 was downregulated; under prolonged hypoxia condition, the expression of PFKFB3 was gradually downregulated and was prominent in 72 h (**Fig. 7E-H).**

### Sorafenib has no direct effect on the regulation of PFKFB3 and HIF-1α

**Figure 8:**
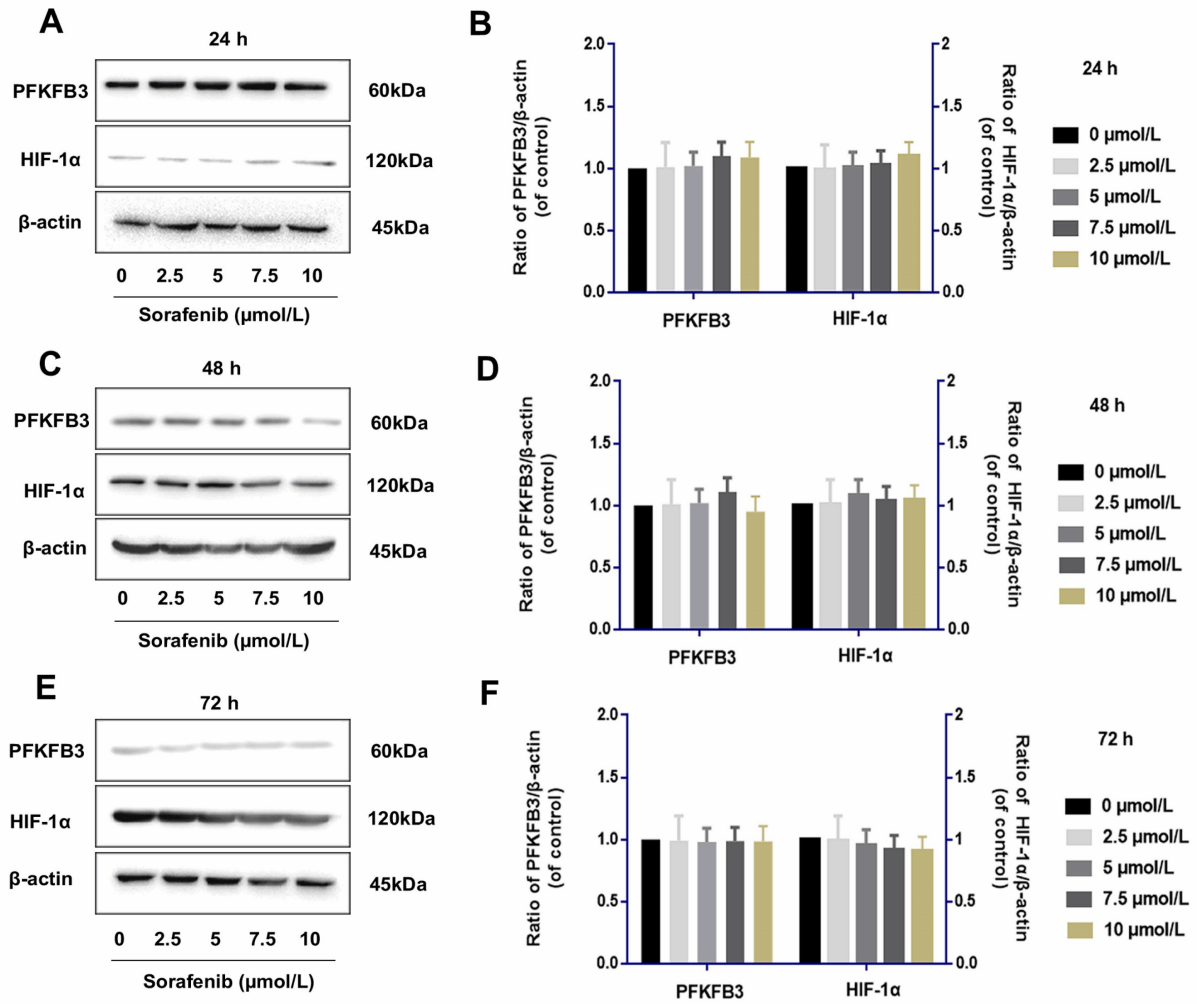
Sorafenib had no direct effect on the regulation of PFKFB3 and HIF-1α (**A-F**)**.** PFKFB3 and HIF-1α levels were similar among various dosages of sorafenib treatment in HCCLM3-IL-6(-) cells from 24 h to 72 h; PFKFB3 was gradually downregulated after 72 h of treatment, while HIF-1α was gradually upregulated after 72 h of treatment.

Tumor cells were examined by Western blot assay to determine whether IL-6 knockout affects the role of sorafenib in regulating PFKFB3 and HIF-1α. The levels of PFKFB3 and HIF-1α were similar among various dosages of sorafenib treatment from 24 h to 72 h. This finding suggested the lack of direct effect of sorafenib on the regulation of PFKFB3 and HIF-1α. However, PFKFB3 was gradually downregulated after 72 h of treatment, while HIF-1α was gradually upregulated after 72 h of treatment (**Fig. 8**).

### PFKFB3 silencing attenuated the side pro-invasive effect of sorafenib

**Figure 9:**
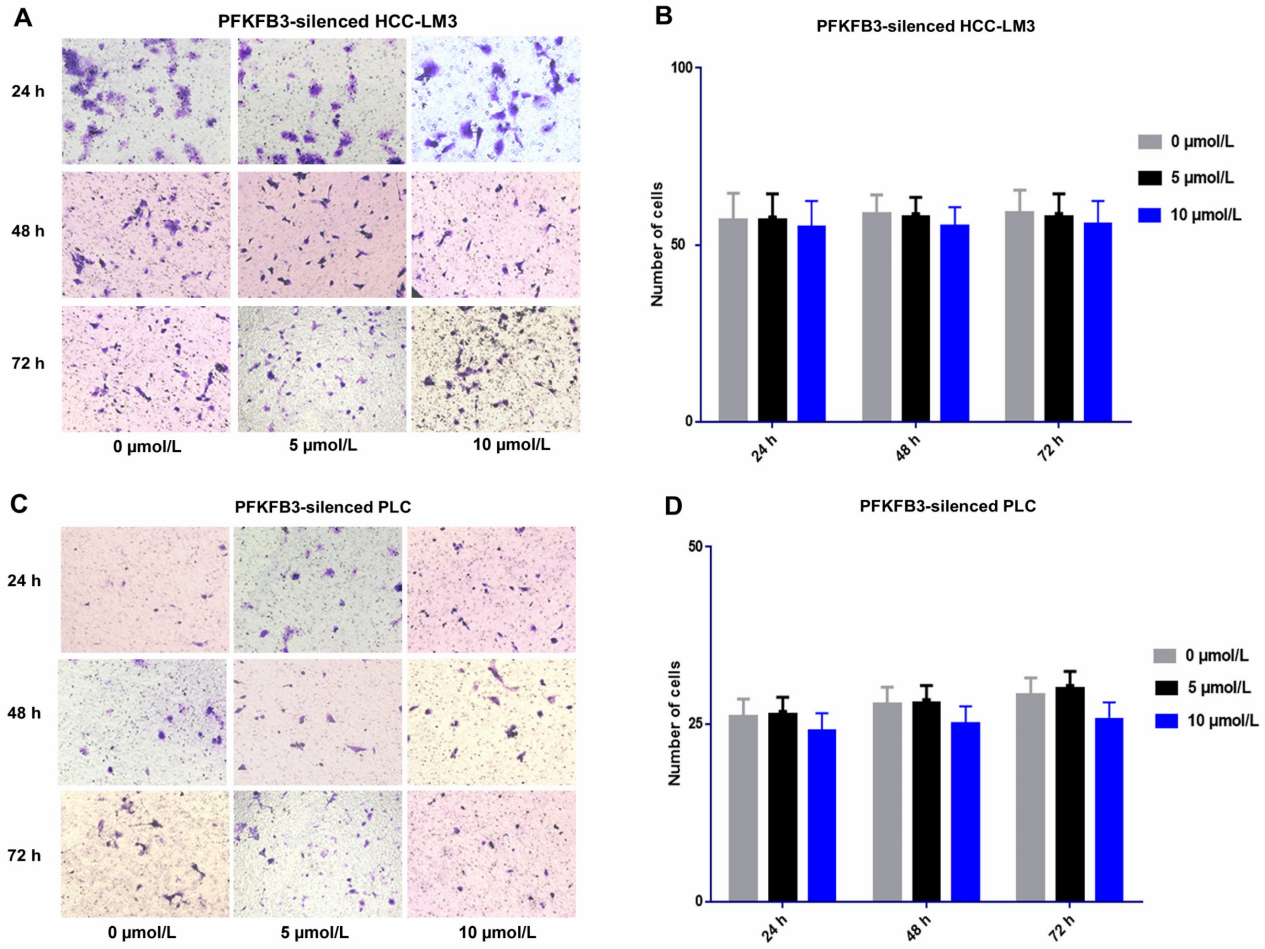
Sorafenib increased the metastatic potential of liver cancer cells, and PFKFB3 silencing attenuated the pro-invasive effect induced by sorafenib treatment. (**A-D**) Transwell assays revealed that PFKFB3 silencing attenuated the pro-invasive effect induced by sorafenib in PFKFB3-silenced HCCLM3 cells (**A-B**) and PFKFB3-silenced PLC cells (**C-D**).

For PFKFB3-silenced HCC-LM3 cells, the promotion effect was attenuated under sorafenib treatment with various dosages (5 and 10 µmol/L). No significant difference was found among various dosages of sorafenib. Sorafenib promoted the invasion of HCCLM3-wt cells more significantly than that of PFKFB3-silenced HCC-LM3 cells. Moreover, PFKFB3 silencing may reduce the pro-invasion side effect of sorafenib. Similar results were observed in PFKFB3-silenced PLC (**Fig. 9**).

### IL-6 knockout could weaken the pro-invasion effect induced by low and median dosages of sorafenib

**Figure 10:**
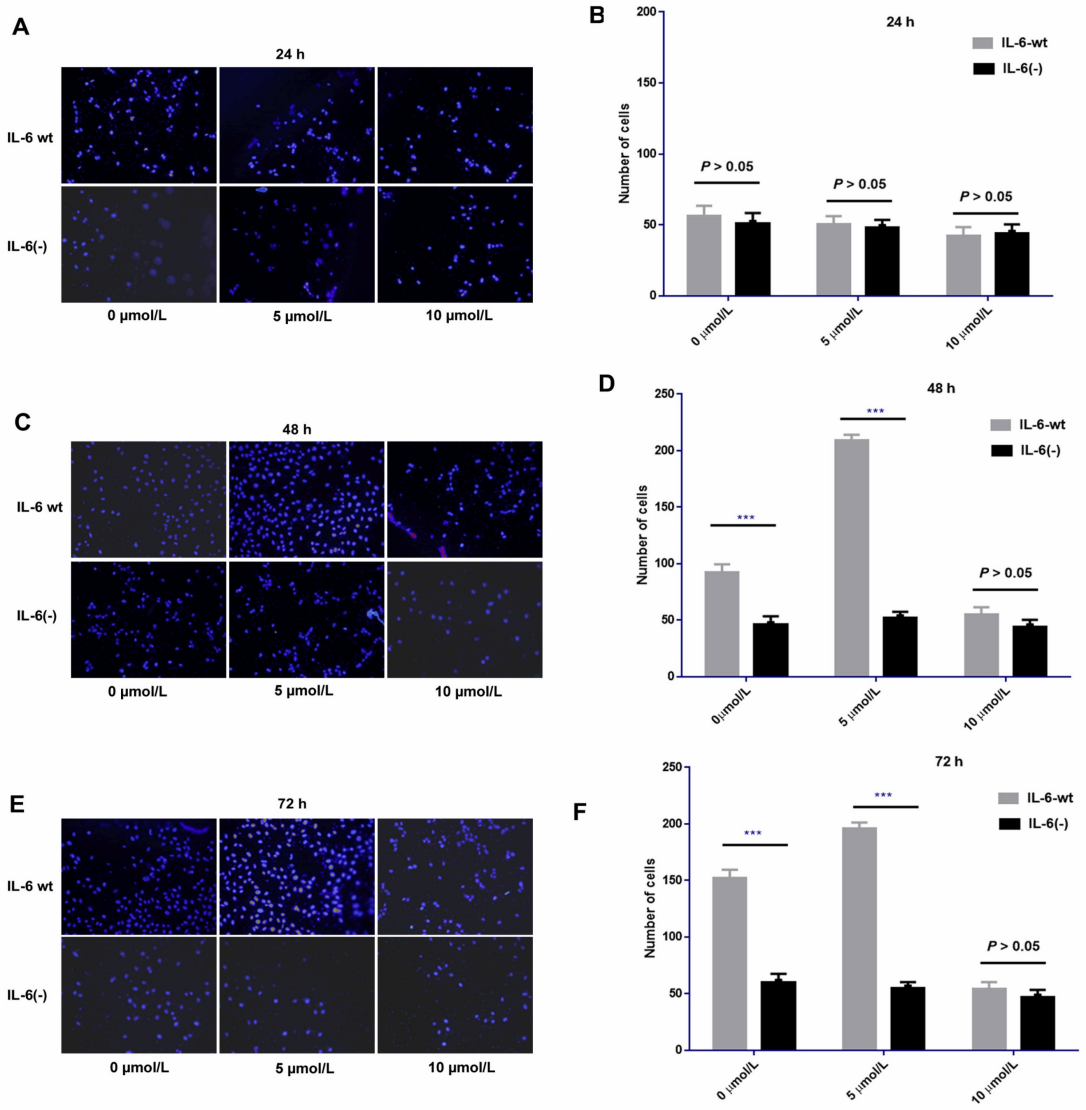
IL-6 knockout affected cell invasion independent of various dosages of sorafenib and weakened the pro-invasion effect induced by the low and median dosages of sorafenib from 48 h to 72 h. Sorafenib had no significant effect on the invasion of HCC-LM3 cells after 24 h of treatment compared with that of HCCLM3-wt cells** (A-B, *P* > 0.05)**; after 48 h and 72 h of treatment, the pro-invasive effect induced by median dosage of sorafenib was significantly weakened compared with that in HCCLM3-wt cells (48 h, C-D; 72 h, E-F; *** P < 0.001)

After IL-6 was knocked out, sorafenib exerted no obvious effect on cell invasion compared with HCCLM3-wt cells after 24 h of treatment. No significant difference was found among low, medium, and high dosages of sorafenib. However, after 48 and 72 h of treatment, the pro-invasive effect exerted by low or median dosage of sorafenib was significantly weakened compared with that in HCCLM3-wt cells. However, the effect on cell invasion at each time point was independent of sorafenib dosage. IL-6 knockout affected cell invasion independent of sorafenib dosage, that is, it had an obvious inhibitory effect on cell invasion from 48 h to 72 h. Moreover, the knock-out weakened the pro-invasion effect of low and median dosages of sorafenib from 48 h to 72 h (**Fig. 10**).

### PFKFB3 knocked down reduces the pro-invasive effect of sorafenib in vivo

**Figure 11:**
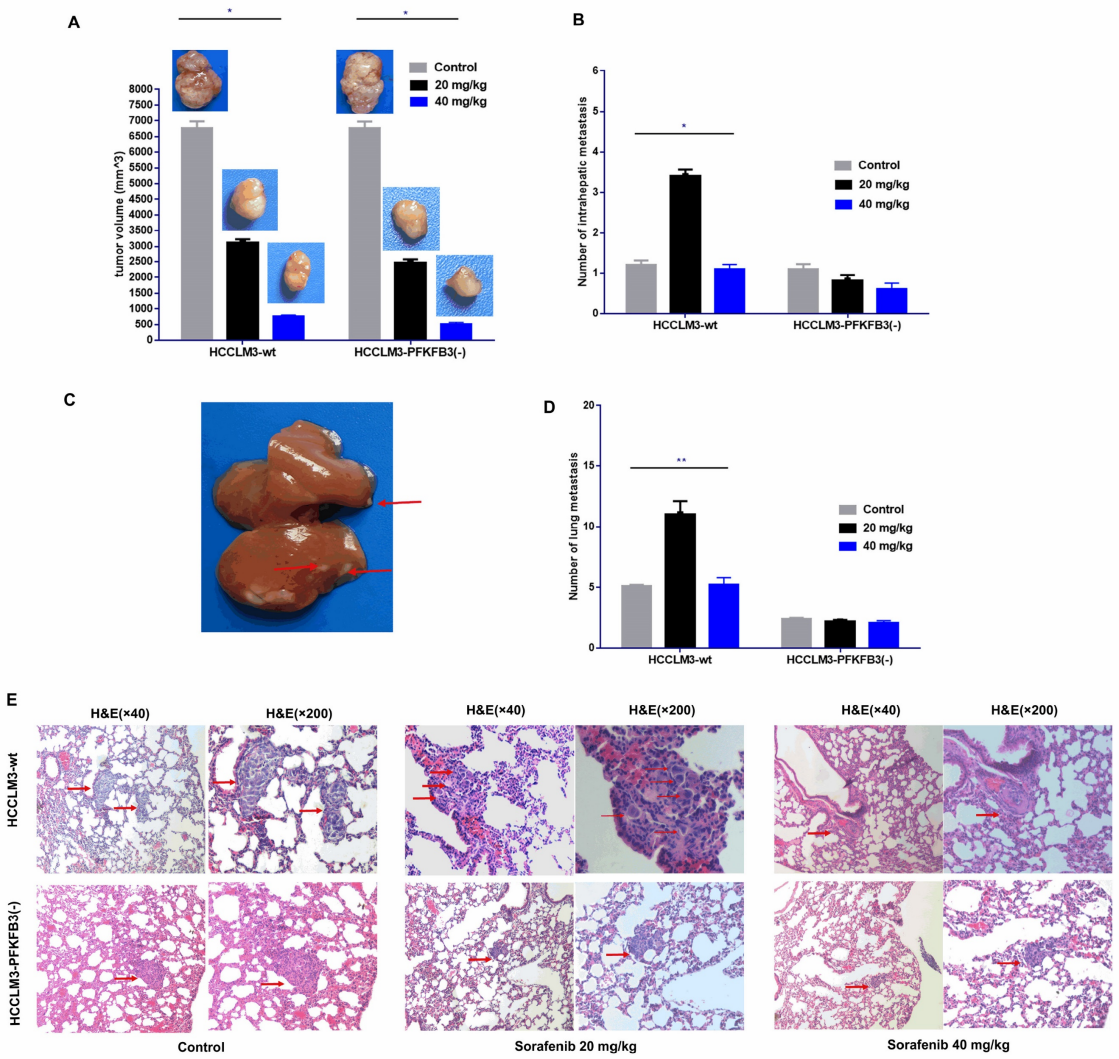
Sorafenib decreased the tumor volume, and the administration of sorafenib with a median dosage (20 mg/kg) increased the intrahepatic metastatic potential and lung metastatic potential of liver cancer cells; sorafenib with a high dosage (40 mg/kg) inhibited intrahepatic metastasis and lung metastasis. PFKFB3 knockout attenuated the pro-invasive effect of sorafenib treatment in vivo. (**A**) Sorafenib decreased the tumor volume in the HCCLM3‑wt and HCCLM3-PFKFB3(-) groups (both * *P* < 0.05, respectively). (**B and C**) Sorafenib with a median dosage (20 mg/kg) increased the number of IHM in the HCCLM3‑wt sorafenib group compared with that in the HCCLM3-wt control group (* *P* < 0.05), while sorafenib with a high dosage (40 mg/kg) did not increase the number of IHMs in the HCCLM3-wt group; sorafenib did not increase the number of IHMs in the HCCLM3-PFKFB3(-) sorafenib group compared with that in the HCCLM3-PFKFB3(-) control group. The number of IHMs in the HCCLM3-wt control was not higher than that in the HCCLM3-PFKFB3(-) control (*P* > 0.05). **(D and E)** Sorafenib increased the number of lung metastases in the HCCLM3‑wt sorafenib group compared with that in the HCCLM3‑wt control, while sorafenib did not increase the number of lung metastases in the HCCLM3-PFKFB3(-) sorafenib group compared with that in the HCCLM3-PFKFB3(-) control. (** *P* < 0.01). The number of lung metastases in the HCCLM3-wt control was higher than that in the HCCLM3-PFKFB3(-) control.

An orthotopic tumor model in nude mice was established to discover whether PFKFB3 influences the pro-invasive effect of sorafenib. Sorafenib significantly decreased the tumor size in the HCCLM3-wt and HCCLM3-PFKFB3(-) groups (**Fig. 11A**). Inversely, more intrahepatic metastasis (IHM) was found in the HCCLM3-wt group treated with median dosage of sorafenib (20 mg/kg) but not in the group with higher dosage (40 mg/kg). The pro-invasive effect was inhibited, that is, IHM was significantly inhibited in the HCCLM3-PFKFB3(-) group under sorafenib treatment with median dosage (**Fig. 11B-C**). The IHM in the HCCLM3-wt control group was similar to that in the HCCLM3‑PFKFB3(‑a) control (**Fig. 11B**). In terms of lung metastasis, the metastasis nodules were higher in HCCLM3‑wt cells under sorafenib administration with a median dosage (20 mg/kg) (**Fig. 11D-E**). When PFKFB3 was knocked down, lung metastasis was similar between the control and sorafenib group. Our in vivo study indicated that PFKFB3 affected cell invasion but had no direct effect on tumor growth or metastasis. PFKFB3 knockdown reduced the pro-invasive effect of sorafenib with median dosage.

## Discussion

Although sorafenib treatment could inhibit the proliferation of tumors through multiple targets^21^-^23^, in the present study, the in vitro results revealed that high-dosage sorafenib (10 µmol/L) inhibited the invasion of HCCLM3-wt cells from 24 h to 72 h. Sorafenib with low and median dosages (2.5-7.5 µmol/L) promoted cell invasion in 48 and 72 h. Sorafenib with median dosage of 20 mg/kg increased the intrahepatic metastasis (IHM) and lung metastasis (LM) of liver cancer cells, while sorafenib with a high dosage of 40 mg/kg inhibited IHM and LM. The pro-invasive effect was mediated by IL-6/HIF-1α/PFKFB3 regulation, which was specific to sorafenib dosage and treatment time.

In the early stage of treatment, various dosages of sorafenib could up-regulate the expression of IL-6, thereby up-regulating HIF-1α and down-regulating PFKFB3. In the later stage, low and medium dosages of sorafenib could still up-regulate the expression of IL-6 but inversely downregulate HIF-1α, resulting in the upregulation of PFKFB3. In the later stage of treatment, high dosages of sorafenib could continuously induce the downregulation of IL-6 expression and the upregulation of HIF-1α expression, thereby continuing to downregulate the expression of PFKFB3 and inhibiting tumor growth and invasiveness. Therefore, although sorafenib could inhibit cell proliferation in HCC, it affected the level of PFKFB3 through time-specific mediation of IL-6/HIF-1α at different times, thereby affecting the cell invasion in HCC. Hence, dosage-specific sorafenib could exert different effects on cell invasion in HCC in different treatment stages.

Our previous studies suggested that sorafenib can inhibit tumor cell proliferation, but we could not specifically distinguish among different treatment times and drug dosages. Therefore, our present study suggested that the effect of sorafenib was time and dosage specific. Low and medium dosages of sorafenib could inhibit tumor invasion in the early stage (24 h). After administration for 48 h and 72 h, although it could still inhibit tumor proliferation, it gradually increased tumor invasiveness. High dosages of sorafenib could inhibit tumor proliferation and invasion throughout the treatment process. In in vivo study, the pro‑migratory effect was observed after treatment with the median dosage of sorafenib. As such, sorafenib with median dosage (20 mg/kg) could increase the intrahepatic metastasis (IHM) and lung metastasis (LM) of liver cancer cells, while sorafenib with high dosage (40 mg/kg) could inhibit both IHM and LM.

By further exploring the mechanism, our previous work suggested that IL-6 was associated with sorafenib-induced tumor invasiveness but only through IL-6, and its subsequent signaling pathways such as PI3K/JAK/STAT may not fully explain the time and dosage-specific effects of sorafenib on HCC cells. PFKFB3 in aerobic glycolysis is related to the mediation of IL-6, as was confirmed in other studies^24^, ^25^. The present research suggested that low and high dosages of sorafenib could inhibit cell proliferation and invasion in the early stage. Subsequent biological effects could not be explained by the upregulation of IL-6. However, the downregulation of the expression of PFKFB3 may explain the biological effect of the downregulation of tumor invasion and proliferation by sorafenib despite the upregulation of IL-6 at this time.

Our study found that the regulation of IL-6 and PFKFB3 was also time specific. Previous works to explain such specificity suggested that IL-6 can further regulate the expression of PFKFB3 by regulating HIF-1α^26^-^28^. In the present study, the effect of IL-6 on HIF-1α may also be time specific. That is, the regulation between IL-6 and HIF-1α was positive feedback in the early stage (24 h) and negative feedback in the later stage (48 and 72 h). IL-6 mainly initiates subsequent signaling pathways through IL-6R by binding to gp130. In the early process, the IL-6/IL-6R/gp130 signal pathway could initiate subsequent signaling pathways, such as the JAK/STAT pathway, thereby up-regulating HIF-1α. In the later stage of treatment, some molecules could generate soluble sgp130 though proteolysis or alternative splicing, weakening the subsequent effects of the IL-6/IL-6R/gp130 signal pathway. In the later stage (48 and 72 h), the upregulation of IL-6 could not further induce HIF-1α upregulation; the existence and properties of this molecule as well as the expression level of sgp130 need further confirmation in our future studies.

We elucidated the relationship between hypoxia and PFKFB3, which was upregulated in tumor aerobic glycolysis^29^-^32^. We found differences in PFKFB3 expression in tumor cells under normoxia and hypoxia. As such, hypoxia could downregulate the expression of PFKFB3; by contrast, under normoxia, the upregulation of PFKFB3 in tumor cells was observed. Moreover, sorafenib had no direct effect on PFKFB3. The time-dependent and dosage-specific effects of sorafenib on tumor invasion may be due to differences in the regulation pattern of the effect of IL-6 on HIF-1α, namely, positive regulation in the early stage and negative regulation in the later stage. This phenomenon led to the biological effects of HIF-1α/PFKFB3-related signaling pathways.

To further discover the function of PFKFB3 on hepatocellular carcinoma, our functional studies of PFKFB3 in vitro confirmed its effects on cell activities. The effect of sorafenib on cell invasiveness was correlated with aerobic glycolysis. First, PFKFB3 was overexpressed in HCC cells. Second, silenced PFKFB3 reduced the cell proliferation and migration in HCC. Finally, PFKFB3 promoted cell migration in HCC cells through EMT-related proteins.

Enhanced glycolysis levels are important for tumorigenesis and cell proliferation because tumor cells need to metabolize glucose to meet the increased energy and material metabolism demands of malignant cells. For the abnormal vasculature that provides oxygen and nutrients to tumor tissues, most tumors are in a state of relative hypoxia, further activating glycolysis. Intracellular Fru-2,6-P_2_ is mostly regulated by PFKFB3, which is activated by hypoxia stimuli^32^, ^33^. The mRNA and protein levels of PFKFB3 increased in different tumors, and PFKFB3 overexpression was observed to be associated with lymphatic metastasis, tumor staging, and poor prognosis^34^-^36^.

In colorectal cancer, the proinflammatory cytokine IL-6 regulated PFKFB3 expression by activating STAT3 to enhance glycolysis; this finding suggests that chronic inflammation-related PFKFB3 could promote the occurrence and development of colorectal cancer 24, 25. To synthesize ATP for energy, endothelial cells rely more on glycolysis; in addition, the lack of glycolysis catalyst PFKFB3 impedes the proliferation of endothelial cells^37^. The inhibition of glycolysis of endothelial cells by targeting PFKFB3 can reduce the metastasis of tumor cells by normalizing tumor blood vessels. This process can improve chemotherapeutic drug delivery and efficacy, thereby providing a promising treatment strategy^38^. Therefore, the exact roles of PFKFB3 in HCC are worthy of further exploration.

In this study, PFKFB3 was examined in different HCC cell lines and normal hepatocytes. PFKFB3 was expressed in liver cancer cells and liver cells, while PFKFB3 was significantly upregulated in liver cancer cells. Hence, PFKFB3 may be a tumor-associated antigen rather than a tumor-specific antigen. PFKFB3 was distinct in different categories of HCC cells. The mRNA expression of PFKFB3 from high to low was arranged as follows: PLC, HCC-LM3, HuH-7, and Hep G2. The protein level was organized from high to low as follows: Hep G2, HCC-LM3, PLC, and HuH-7. The inconsistency in the mRNA and protein levels of PFKFB3 was observed in Hep G2. The processes of transcription and translation may have some modifications for regulating protein synthesis or degradation. The result could also be attributed to absolute quantitative methods used for data processing in RT-PCR and Western blot analysis. Therefore, Hep G2 was not selected as the experimental model, but HCC-LM3 and PLC were used for subsequent experiments.

After confirming the high expression of PFKFB3 in HCC cells, the present study explored its role in cell proliferation and migration. The knockdown of PFKFB3 inhibited the proliferation and migration of HCC-LM3 and PLC cells, consistent with the results in a variety of tumors^36^, ^39^-^44^. PFKFB3 was downregulated, and Snail was subsequentially downregulated among EMT-TFs. As a result, E-cadherin, an epithelial marker, was upregulated, while N-cadherin and vimentin, which are mesenchymal markers, were inversely downregulated. Combined with the above findings of the cell migration experiment, PFKFB3 may promote HCC cell migration in vitro by affecting the EMT progress. This study aims to explore the function of PFKFB3 in the pro-invasive effect of sorafenib and sheds new light on the treatment of HCC by targeting PFKFB3 in sorafenib administration.

Although both of our studies and Zhang et al 20 found that certain dosage of sorafenib could promote the invasion and metastasis of liver cancer; However, there were certain difference between these two studies, in terms of the design, the adopted dosage of sorafenib and the main mechanisms related to the pro-invasive effect of sorafenib. Firstly, our study was performed both in vitro and in vivo, and we adopted different dosage of sorafenib in vitro and in vivo which was determined according to the conversion relationship; Zhang's study focused only on in vivo studies; then, because different dosage of sorafenib could exert different effects on invasion and metastasis of liver cancer, therefore we designed different dosages in vitro and in vivo studies. Meanwhile, in Zhang's study, 30mg/kg of sorafenib was administrated in vivo studies; Finally, since sorafenib is a multi-target antitumor drug with multiple signaling pathways involved, the mechanism of promoting invasion and metastasis found in our study mainly relied on IL-6/HIF-1α/PFKFB3 signaling pathway, and Zhang et al found Sorafenib promotes invasiveness and the metastatic potential of HCC by down-regulating expression of HTATIP2 via JAK-STAT3 signaling. I think there is no conflict and contradiction between these two studies, and because of the existence of multiple targets and the interaction of multiple signaling pathways which may be involved in the pro-invasive effect of sorafenib, the subsequent further studies may prompt us to explore whether there is interaction between these two signaling pathways.

Although the idea of this study is comparatively simple, its logic is considerably rigorous. Sorafenib inhibited tumor growth, but its side pro-invasive effect promoted IHM and LM, leading to poor prognosis. PFKFB3, the key factor in the Warburg effect, was overexpressed in liver cancer, and the regulation of IL-6/HIF-1α/PFKFB3 played an important role in dosage-specific pro-invasive effect of sorafenib. The present work has limitations. Firstly, although some studies have confirmed that high expression of PFKFB3 in HCC cases is related to poor prognosis, pathological specimens and clinical data of HCC in our center need to be collected. More importantly, after determining the prognostic value between PFKFB3 and HCC, the therapeutic efficacy and prognosis of patients based on the different expression levels of PFKFB3 after sorafenib treatment should be further studied.

## Conclusions

Specific dosage of sorafenib could exert different effects on cell invasion in HCC in different treatment stages. IL-6 mediated HIF-1α/PFKFB3 distinct regulation in different treatment times and could be targeted to avoid the side pro-invasive effect and improve the therapeutic efficacy of sorafenib.

## Supplementary Material

Supplementary tables.
