# Supplementary material for: Sorafenib promotes hepatocellular carcinoma invasion via interleukin-6/HIF-1α/PFKFB3
Source: J Cancer. 2023 Jun 26;14(10):1859–74. doi: 10.7150/jca.84451 (PMC10355201; doi:10.7150/jca.84451)
Supplement: Supplementary file 1 — Supplementary tables. [file jcav14p1859s1.pdf]

**supplementary Table 1**

| id | logFC | AveExpr | t | P.Value | adj.P.Val | B |
|---|---|---|---|---|---|---|
| PACRGL | 2.12784 | 8.468473 | 14.34171 | 3.72E-22 | 7.62E-18 | 39.97897 |
| TTC21B | 2.396608 | 8.978036 | 14.11486 | 8.36E-22 | 7.62E-18 | 39.18892 |
| ZNF273 | 2.537343 | 9.195216 | 13.95995 | 1.46E-21 | 7.62E-18 | 38.64546 |
| PNPT1 | 1.324461 | 10.19059 | 13.95743 | 1.47E-21 | 7.62E-18 | 38.63657 |
| LILRB1 | 1.620886 | 9.364815 | 13.63215 | 4.79E-21 | 1.94E-17 | 37.48474 |
| ACBD7 | 2.10588 | 8.164139 | 13.58729 | 5.64E-21 | 1.94E-17 | 37.32477 |
| RAX2 | 2.376325 | 9.104268 | 13.44462 | 9.51E-21 | 2.81E-17 | 36.81418 |
| SMCR5 | 2.743575 | 8.983701 | 13.3821 | 1.20E-20 | 3.09E-17 | 36.58959 |
| TERF1 | 2.130588 | 8.5944 | 13.17218 | 2.60E-20 | 5.97E-17 | 35.83166 |
| TNR | 1.895449 | 7.366158 | 13.13496 | 2.98E-20 | 6.17E-17 | 35.69665 |
| ARHGAP8 | 2.013771 | 8.202183 | 13.10404 | 3.34E-20 | 6.29E-17 | 35.58435 |
| CNGB1 | 1.77259 | 7.812317 | 12.92728 | 6.46E-20 | 1.11E-16 | 34.93995 |
| PPA2 | 1.459204 | 10.15234 | 12.74485 | 1.28E-19 | 1.98E-16 | 34.27057 |
| GSR | 1.705046 | 9.736318 | 12.73291 | 1.34E-19 | 1.98E-16 | 34.2266 |
| LIN28 | 2.603898 | 7.659965 | 12.68222 | 1.62E-19 | 2.10E-16 | 34.03976 |
| FLJ32784 | 2.209579 | 8.76542 | 12.67572 | 1.66E-19 | 2.10E-16 | 34.01576 |
| ZNF786 | 1.656747 | 8.240833 | 12.66546 | 1.72E-19 | 2.10E-16 | 33.97792 |
| FKTN | 1.287652 | 9.324049 | 12.64083 | 1.89E-19 | 2.18E-16 | 33.88693 |
| FGD2 | 2.014527 | 9.673196 | 12.6009 | 2.20E-19 | 2.40E-16 | 33.73929 |
| XCR1 | 1.893088 | 7.605287 | 12.5634 | 2.54E-19 | 2.62E-16 | 33.60045 |
| NPTX1 | 2.277526 | 7.415811 | 12.53465 | 2.83E-19 | 2.79E-16 | 33.49388 |
| C2orf69 | 2.097993 | 9.797851 | 12.4859 | 3.40E-19 | 3.20E-16 | 33.3129 |
| OR6X1 | 1.034787 | 6.867085 | 12.47418 | 3.55E-19 | 3.20E-16 | 33.26935 |
| LOC90586 | 2.230207 | 9.727103 | 12.46074 | 3.74E-19 | 3.23E-16 | 33.21941 |
| GALNT3 | 1.393314 | 7.210788 | 12.43283 | 4.16E-19 | 3.44E-16 | 33.11557 |
| OCIAD1 | 1.075787 | 11.28515 | 12.38274 | 5.03E-19 | 4.00E-16 | 32.92898 |
| SFTPB | 1.64182 | 7.211016 | 12.37255 | 5.23E-19 | 4.01E-16 | 32.89099 |
| GJC1 | 0.866109 | 11.2262 | 12.33983 | 5.92E-19 | 4.38E-16 | 32.76885 |
| ZNF266 | 1.597554 | 8.069446 | 12.26976 | 7.74E-19 | 5.52E-16 | 32.50689 |
| HTR2A | 2.937905 | 8.347487 | 12.22739 | 9.10E-19 | 6.27E-16 | 32.34819 |
| OR2A12 | 1.334323 | 7.007939 | 12.21086 | 9.69E-19 | 6.27E-16 | 32.28621 |
| SLC16A12 | 1.209795 | 10.94184 | 12.20249 | 1.00E-18 | 6.27E-16 | 32.25482 |
| HSPC268 | 1.98314 | 8.159231 | 12.19488 | 1.03E-18 | 6.27E-16 | 32.22629 |
| CYCSL1 | 2.029611 | 8.768694 | 12.19475 | 1.03E-18 | 6.27E-16 | 32.22576 |
| SLC35F3 | 2.366717 | 7.559676 | 12.12365 | 1.35E-18 | 7.79E-16 | 31.95866 |
| POU4F1 | 2.962533 | 8.029725 | 12.12325 | 1.36E-18 | 7.79E-16 | 31.95714 |
| GYG2 | 1.59956 | 7.896889 | 12.09316 | 1.52E-18 | 8.32E-16 | 31.84389 |
| NLRP8 | 1.662796 | 9.745601 | 12.08995 | 1.54E-18 | 8.32E-16 | 31.83183 |
| TRIM16L | 2.325843 | 9.934863 | 12.08533 | 1.57E-18 | 8.32E-16 | 31.81439 |
| PRAMEF7 | 3.110577 | 7.824825 | 12.07796 | 1.61E-18 | 8.35E-16 | 31.78665 |
| SERPINB6 | -2.38912 | 9.325981 | -12.0004 | 2.17E-18 | 1.08E-15 | 31.49408 |
| LILRA6 | 1.870202 | 7.939954 | 11.99856 | 2.19E-18 | 1.08E-15 | 31.48711 |
| FHL2 | 1.720342 | 8.669475 | 11.97998 | 2.35E-18 | 1.11E-15 | 31.41689 |
| OR1J1 | 1.553368 | 7.309702 | 11.9782 | 2.37E-18 | 1.11E-15 | 31.41019 |
| ECH1 | -2.30106 | 8.288092 | -11.9704 | 2.44E-18 | 1.12E-15 | 31.38081 |
| SNRNP48 | 1.677244 | 9.808614 | 11.95217 | 2.62E-18 | 1.16E-15 | 31.31175 |
| FLJ44124 | 1.728393 | 11.13532 | 11.94422 | 2.70E-18 | 1.16E-15 | 31.28166 |
| SPDYE1 | 1.781361 | 7.331603 | 11.94035 | 2.74E-18 | 1.16E-15 | 31.26703 |
| NME4 | -1.51626 | 9.960672 | -11.94 | 2.74E-18 | 1.16E-15 | 31.26567 |
| RRP7A | -2.06307 | 9.349117 | -11.8929 | 3.29E-18 | 1.36E-15 | 31.08746 |
| ZNF430 | 1.873647 | 10.58551 | 11.85137 | 3.86E-18 | 1.57E-15 | 30.92975 |
| CCBE1 | 1.295368 | 9.811031 | 11.70672 | 6.77E-18 | 2.69E-15 | 30.3794 |
| POT1 | 1.444115 | 7.779291 | 11.69702 | 7.03E-18 | 2.75E-15 | 30.34241 |
| GSDMA | 1.291149 | 7.279798 | 11.65457 | 8.29E-18 | 3.18E-15 | 30.18035 |
| ZNF826 | 1.784597 | 7.843111 | 11.64736 | 8.53E-18 | 3.21E-15 | 30.15282 |
| FAM183B | 2.678743 | 7.835654 | 11.59714 | 1.04E-17 | 3.83E-15 | 29.96077 |

| | | | | | | |
|---|---|---|---|---|---|---|
| GABPB2 | 1.628551 | 9.929237 | 11.5919 | 1.06E-17 | 3.84E-15 | 29.9407 |
| ITPRIPL1 | 2.028704 | 7.685238 | 11.57889 | 1.11E-17 | 3.97E-15 | 29.8909 |
| FAM123C | 1.398076 | 6.999738 | 11.56946 | 1.16E-17 | 4.05E-15 | 29.8548 |
| HECTD2 | 1.18862 | 8.258199 | 11.55239 | 1.23E-17 | 4.26E-15 | 29.78939 |
| NUBPL | 1.330752 | 10.62766 | 11.53948 | 1.30E-17 | 4.41E-15 | 29.73989 |
| SNTN | 1.90293 | 7.911916 | 11.53239 | 1.34E-17 | 4.46E-15 | 29.71274 |
| ASPSCR1 | -2.57588 | 9.550489 | -11.5168 | 1.42E-17 | 4.66E-15 | 29.65301 |
| UBL4B | 2.431376 | 7.659793 | 11.49813 | 1.53E-17 | 4.94E-15 | 29.58126 |
| FLJ40453 | 2.661057 | 9.640603 | 11.47492 | 1.67E-17 | 5.32E-15 | 29.49214 |
| SRRM2 | -2.29794 | 9.357245 | -11.4697 | 1.71E-17 | 5.35E-15 | 29.47205 |
| C2orf14 | 1.959933 | 7.91731 | 11.45868 | 1.78E-17 | 5.50E-15 | 29.42975 |
| MRPL44 | 1.537152 | 10.48292 | 11.44083 | 1.91E-17 | 5.81E-15 | 29.3611 |
| IL1F10 | 1.166493 | 6.944148 | 11.34685 | 2.76E-17 | 8.28E-15 | 28.99914 |
| LOC34299 | 1.549839 | 7.063535 | 11.29377 | 3.40E-17 | 1.01E-14 | 28.79427 |
| LOC55422 | 1.725154 | 7.348081 | 11.25119 | 4.02E-17 | 1.16E-14 | 28.62969 |
| MIR551B | 1.253295 | 6.971911 | 11.249 | 4.06E-17 | 1.16E-14 | 28.62122 |
| CCDC144N | 1.069778 | 7.05277 | 11.24607 | 4.10E-17 | 1.16E-14 | 28.60988 |
| LIMS3 | 1.841722 | 7.656034 | 11.20227 | 4.88E-17 | 1.36E-14 | 28.44033 |
| MIR125B1 | 2.245779 | 7.594347 | 11.17464 | 5.44E-17 | 1.50E-14 | 28.33323 |
| PIGQ | -2.21835 | 9.45803 | -11.1716 | 5.51E-17 | 1.50E-14 | 28.32157 |
| MSH3 | 1.597124 | 10.69522 | 11.15414 | 5.90E-17 | 1.59E-14 | 28.25375 |
| C21orf58 | 1.31723 | 8.035062 | 11.13093 | 6.46E-17 | 1.72E-14 | 28.16368 |
| SLA2 | 2.143297 | 8.372093 | 11.11621 | 6.85E-17 | 1.80E-14 | 28.10652 |
| RASSF7 | -1.99866 | 8.535493 | -11.0993 | 7.33E-17 | 1.87E-14 | 28.04089 |
| LOC64699 | 2.039695 | 9.504462 | 11.09914 | 7.33E-17 | 1.87E-14 | 28.04019 |
| PPP2R2B | 1.977689 | 7.976461 | 11.0628 | 8.46E-17 | 2.14E-14 | 27.89894 |
| CRCP | 1.102194 | 8.501739 | 11.05837 | 8.61E-17 | 2.15E-14 | 27.88171 |
| PSG7 | 1.022059 | 6.871718 | 11.01184 | 1.04E-16 | 2.55E-14 | 27.70059 |
| KCNH6 | 0.959007 | 7.420214 | 10.99337 | 1.11E-16 | 2.71E-14 | 27.62859 |
| GRM6 | 1.118917 | 7.062175 | 10.97146 | 1.22E-16 | 2.91E-14 | 27.54319 |
| REXO1 | -2.10738 | 8.448836 | -10.97 | 1.22E-16 | 2.91E-14 | 27.53742 |
| LOC10019 | 1.841316 | 8.761533 | 10.96104 | 1.27E-16 | 2.98E-14 | 27.50254 |
| LOC26033 | 2.388604 | 8.553938 | 10.95152 | 1.32E-16 | 3.06E-14 | 27.46538 |
| NDUFS7 | -2.80444 | 9.336653 | -10.9442 | 1.35E-16 | 3.12E-14 | 27.43688 |
| ZCCHC2 | -1.70164 | 7.721143 | -10.9357 | 1.40E-16 | 3.19E-14 | 27.40347 |
| SYVN1 | -1.08483 | 7.805149 | -10.9324 | 1.42E-16 | 3.19E-14 | 27.39079 |
| CDCP1 | 1.187085 | 7.378585 | 10.92884 | 1.44E-16 | 3.20E-14 | 27.37688 |
| TPM3 | 1.1302 | 10.2239 | 10.89353 | 1.66E-16 | 3.65E-14 | 27.23895 |
| WFDC10A | 1.661246 | 7.215647 | 10.87123 | 1.81E-16 | 3.94E-14 | 27.15175 |
| NLRP7 | 0.947514 | 6.851658 | 10.85087 | 1.96E-16 | 4.23E-14 | 27.07209 |
| RFX4 | 0.856365 | 6.995934 | 10.84273 | 2.03E-16 | 4.33E-14 | 27.04025 |
| ALPP | 1.701601 | 8.169042 | 10.8353 | 2.09E-16 | 4.39E-14 | 27.01117 |
| CD300LD | 1.886694 | 7.298692 | 10.83188 | 2.12E-16 | 4.39E-14 | 26.99776 |
| GYPE | 1.712225 | 7.239748 | 10.83162 | 2.12E-16 | 4.39E-14 | 26.99677 |
| OR2G6 | 2.086643 | 7.411657 | 10.78912 | 2.51E-16 | 5.15E-14 | 26.83025 |
| DEM1 | 1.431229 | 8.13461 | 10.78212 | 2.58E-16 | 5.21E-14 | 26.8028 |
| LRP3 | -2.68634 | 9.459322 | -10.781 | 2.59E-16 | 5.21E-14 | 26.79841 |
| CCDC90B | -1.55481 | 8.469641 | -10.7772 | 2.63E-16 | 5.24E-14 | 26.78368 |
| C1S | -2.16745 | 8.943368 | -10.7725 | 2.68E-16 | 5.27E-14 | 26.76528 |
| DUB3 | 3.126982 | 8.044079 | 10.77092 | 2.70E-16 | 5.27E-14 | 26.75889 |
| LOC25516 | 1.744945 | 8.765462 | 10.76193 | 2.80E-16 | 5.38E-14 | 26.72365 |
| OR4F5 | 1.062538 | 6.855198 | 10.76104 | 2.81E-16 | 5.38E-14 | 26.72015 |
| LOC64543 | 2.751922 | 8.008923 | 10.7587 | 2.84E-16 | 5.38E-14 | 26.71098 |
| ACADVL | -1.62372 | 8.624309 | -10.752 | 2.91E-16 | 5.48E-14 | 26.68458 |
| SORCS1 | 1.802261 | 7.428748 | 10.7485 | 2.95E-16 | 5.51E-14 | 26.67095 |
| C2orf78 | 1.993575 | 7.576732 | 10.74544 | 2.99E-16 | 5.52E-14 | 26.65895 |
| SNORD48 | -3.11091 | 10.71455 | -10.7238 | 3.26E-16 | 5.93E-14 | 26.5738 |
| PCDH10 | 1.095153 | 7.045321 | 10.72194 | 3.28E-16 | 5.93E-14 | 26.56669 |

| | | | | | | |
|---|---|---|---|---|---|---|
| MTFMT | 1.538442 | 8.126558 | 10.72101 | 3.30E-16 | 5.93E-14 | 26.56302 |
| ALDH2 | -2.195 | 8.672864 | -10.7177 | 3.34E-16 | 5.93E-14 | 26.54986 |
| C1orf110 | 3.574876 | 8.375673 | 10.71676 | 3.35E-16 | 5.93E-14 | 26.54633 |
| LRTM1 | 1.290616 | 7.093654 | 10.71462 | 3.38E-16 | 5.93E-14 | 26.53793 |
| CFL1 | -2.17475 | 9.46425 | -10.7053 | 3.51E-16 | 6.10E-14 | 26.50125 |
| PHACTR4 | 1.467092 | 8.351942 | 10.70122 | 3.57E-16 | 6.15E-14 | 26.48529 |
| MLC1 | 0.882098 | 6.890089 | 10.6986 | 3.60E-16 | 6.17E-14 | 26.47501 |
| C1orf150 | 1.863961 | 8.03208 | 10.69196 | 3.70E-16 | 6.28E-14 | 26.44889 |
| ZNF771 | 2.123113 | 8.511386 | 10.68681 | 3.78E-16 | 6.36E-14 | 26.42868 |
| RPS27L | 1.58513 | 9.055047 | 10.67715 | 3.93E-16 | 6.56E-14 | 26.39068 |
| COASY | -1.81385 | 9.182182 | -10.6397 | 4.56E-16 | 7.55E-14 | 26.24341 |
| CTNNA1 | -1.12015 | 7.720333 | -10.6277 | 4.79E-16 | 7.86E-14 | 26.19598 |
| LOC44142 | 2.20277 | 7.49647 | 10.61872 | 4.96E-16 | 8.09E-14 | 26.16079 |
| ZNF765 | 1.933835 | 8.76328 | 10.61142 | 5.11E-16 | 8.22E-14 | 26.13203 |
| CLDN14 | 1.446954 | 8.121592 | 10.61085 | 5.12E-16 | 8.22E-14 | 26.12977 |
| RXRB | -1.79544 | 8.511053 | -10.587 | 5.64E-16 | 8.90E-14 | 26.03569 |
| ST20 | 1.882797 | 8.743734 | 10.5867 | 5.64E-16 | 8.90E-14 | 26.03461 |
| LMX1B | 2.130602 | 7.41987 | 10.58495 | 5.68E-16 | 8.90E-14 | 26.02774 |
| NT5C | -2.21019 | 9.151782 | -10.5833 | 5.72E-16 | 8.90E-14 | 26.0212 |
| NEUROD4 | 1.823807 | 7.283822 | 10.58124 | 5.77E-16 | 8.91E-14 | 26.0131 |
| FLJ46309 | 1.620617 | 9.600935 | 10.57883 | 5.82E-16 | 8.93E-14 | 26.00361 |
| DEFA1B | 2.421337 | 7.760813 | 10.57085 | 6.01E-16 | 9.15E-14 | 25.97216 |
| GNPTAB | 1.538073 | 8.362886 | 10.56414 | 6.18E-16 | 9.33E-14 | 25.94569 |
| C19orf66 | -2.36199 | 8.954442 | -10.5623 | 6.22E-16 | 9.33E-14 | 25.93848 |
| C11orf2 | -2.58027 | 9.222489 | -10.5521 | 6.48E-16 | 9.65E-14 | 25.89825 |
| TSC2 | -1.4445 | 7.994483 | -10.5367 | 6.90E-16 | 1.01E-13 | 25.83728 |
| CIAO1 | 1.007147 | 8.645787 | 10.53622 | 6.91E-16 | 1.01E-13 | 25.83553 |
| FAM75B | 1.712124 | 7.300375 | 10.53603 | 6.91E-16 | 1.01E-13 | 25.83478 |
| RPL18 | -2.80866 | 9.758902 | -10.5303 | 7.07E-16 | 1.02E-13 | 25.81234 |
| SNAPC1 | 1.826766 | 8.773679 | 10.5167 | 7.47E-16 | 1.07E-13 | 25.7585 |
| RAB3IP | 2.192391 | 9.62392 | 10.50773 | 7.75E-16 | 1.11E-13 | 25.72307 |
| ZMAT5 | 1.350427 | 8.200634 | 10.50292 | 7.90E-16 | 1.12E-13 | 25.70407 |
| CAMK2N1 | -1.62895 | 8.554851 | -10.501 | 7.96E-16 | 1.12E-13 | 25.69636 |
| SEMA3E | 1.074032 | 6.989194 | 10.48128 | 8.61E-16 | 1.20E-13 | 25.61857 |
| CHRM2 | 1.054257 | 7.180374 | 10.45963 | 9.40E-16 | 1.31E-13 | 25.533 |
| NYX | 1.037303 | 6.974447 | 10.44931 | 9.80E-16 | 1.35E-13 | 25.49216 |
| BDNF | 0.957417 | 6.976208 | 10.44045 | 1.02E-15 | 1.38E-13 | 25.45711 |
| GABRB1 | 1.954767 | 7.275002 | 10.44023 | 1.02E-15 | 1.38E-13 | 25.45625 |
| OR6T1 | 1.034799 | 6.843251 | 10.43791 | 1.03E-15 | 1.39E-13 | 25.44708 |
| LOC72938 | 3.060237 | 8.025885 | 10.42374 | 1.09E-15 | 1.44E-13 | 25.39102 |
| CEP27 | 1.888843 | 10.17252 | 10.42361 | 1.09E-15 | 1.44E-13 | 25.39048 |
| ARF4 | -1.40514 | 8.276103 | -10.4234 | 1.09E-15 | 1.44E-13 | 25.38961 |
| SYAP1 | 1.501277 | 9.71286 | 10.41453 | 1.13E-15 | 1.49E-13 | 25.35457 |
| OR2G2 | 2.612112 | 7.575876 | 10.40944 | 1.15E-15 | 1.51E-13 | 25.33441 |
| PSG4 | 0.80301 | 6.843636 | 10.405 | 1.17E-15 | 1.52E-13 | 25.31683 |
| DBNL | -1.0927 | 8.200387 | -10.4029 | 1.18E-15 | 1.52E-13 | 25.30847 |
| DUXAP3 | 1.943178 | 9.674321 | 10.40218 | 1.18E-15 | 1.52E-13 | 25.30568 |
| NOG | 2.852523 | 7.870333 | 10.39754 | 1.21E-15 | 1.54E-13 | 25.28727 |
| FLJ25996 | 2.501394 | 7.851164 | 10.38771 | 1.26E-15 | 1.59E-13 | 25.24834 |
| LOC72792 | 1.976219 | 7.363607 | 10.3851 | 1.27E-15 | 1.60E-13 | 25.23802 |
| CCDC130 | -2.40403 | 9.458738 | -10.3692 | 1.35E-15 | 1.70E-13 | 25.17507 |
| PCDHB9 | 1.752572 | 8.093414 | 10.36011 | 1.40E-15 | 1.75E-13 | 25.13897 |
| COX16 | 1.163692 | 8.140147 | 10.35556 | 1.43E-15 | 1.77E-13 | 25.12092 |
| ZNF488 | 2.408483 | 7.597044 | 10.35221 | 1.45E-15 | 1.78E-13 | 25.10765 |
| LRRC37B2 | 2.181675 | 9.940493 | 10.33998 | 1.52E-15 | 1.86E-13 | 25.05915 |
| ISCU | -1.15759 | 8.172026 | -10.3391 | 1.53E-15 | 1.86E-13 | 25.05558 |
| TACC1 | -1.4328 | 8.210829 | -10.3381 | 1.53E-15 | 1.86E-13 | 25.05157 |
| SOD2 | -2.3042 | 9.423254 | -10.3276 | 1.60E-15 | 1.91E-13 | 25.01023 |

| | | | | | | |
|---|---|---|---|---|---|---|
| PRAMEF2 | 1.139403 | 7.028097 | 10.32701 | 1.60E-15 | 1.91E-13 | 25.0077 |
| OR2A20P | 1.186564 | 7.198684 | 10.32631 | 1.61E-15 | 1.91E-13 | 25.00492 |
| CHAT | 1.486872 | 7.213985 | 10.3169 | 1.67E-15 | 1.98E-13 | 24.96758 |
| ZMYM6 | 1.726083 | 8.643447 | 10.31243 | 1.70E-15 | 2.00E-13 | 24.94987 |
| LOC65029 | 1.115357 | 6.982095 | 10.30786 | 1.73E-15 | 2.03E-13 | 24.93173 |
| MALAT1 | 2.105442 | 8.512096 | 10.28697 | 1.88E-15 | 2.19E-13 | 24.84877 |
| LRP5 | -1.2434 | 8.378108 | -10.2859 | 1.89E-15 | 2.19E-13 | 24.84472 |
| OR11H12 | 2.861994 | 8.066078 | 10.28456 | 1.90E-15 | 2.19E-13 | 24.83921 |
| SPAG11A | 3.027142 | 7.771367 | 10.28139 | 1.93E-15 | 2.20E-13 | 24.82661 |
| PASD1 | 1.951738 | 7.268013 | 10.27222 | 2.00E-15 | 2.27E-13 | 24.7902 |
| CD74 | -1.88235 | 8.949586 | -10.2711 | 2.01E-15 | 2.27E-13 | 24.7859 |
| LOC34817 | 2.141932 | 7.556532 | 10.26443 | 2.06E-15 | 2.32E-13 | 24.75927 |
| OR14C36 | 1.950702 | 7.426612 | 10.25848 | 2.11E-15 | 2.37E-13 | 24.73562 |
| MEOX1 | 0.708558 | 6.918593 | 10.25416 | 2.15E-15 | 2.39E-13 | 24.71845 |
| DDRGK1 | -1.04225 | 7.674289 | -10.2495 | 2.19E-15 | 2.42E-13 | 24.69999 |
| ZNF750 | 2.525117 | 7.691242 | 10.24881 | 2.20E-15 | 2.42E-13 | 24.69717 |
| PSG6 | 1.266192 | 7.019847 | 10.24159 | 2.26E-15 | 2.47E-13 | 24.6685 |
| DMC1 | 1.515649 | 8.474414 | 10.24096 | 2.27E-15 | 2.47E-13 | 24.66599 |
| CCNB1IP1 | 1.530879 | 8.91397 | 10.22046 | 2.47E-15 | 2.67E-13 | 24.58444 |
| MYH9 | -1.91462 | 8.624945 | -10.2168 | 2.50E-15 | 2.69E-13 | 24.56975 |
| LCA5L | 2.536503 | 7.782799 | 10.21507 | 2.52E-15 | 2.69E-13 | 24.56301 |
| SCNN1D | -2.17685 | 9.574461 | -10.215 | 2.52E-15 | 2.69E-13 | 24.56277 |
| SLC5A8 | 1.925898 | 10.10103 | 10.21406 | 2.53E-15 | 2.69E-13 | 24.55899 |
| KIR3DL3 | 2.051186 | 7.433454 | 10.20088 | 2.67E-15 | 2.82E-13 | 24.50657 |
| RRBP1 | -2.37778 | 10.11424 | -10.1929 | 2.76E-15 | 2.90E-13 | 24.47497 |
| PHC3 | -0.83846 | 7.347919 | -10.1915 | 2.77E-15 | 2.90E-13 | 24.46928 |
| CDC5L | -1.23696 | 7.472928 | -10.1834 | 2.86E-15 | 2.98E-13 | 24.43686 |
| WASH2P | -1.36269 | 7.612737 | -10.1764 | 2.95E-15 | 3.05E-13 | 24.40904 |
| ACO2 | -1.61211 | 7.959605 | -10.1726 | 2.99E-15 | 3.08E-13 | 24.39403 |
| LOC28491 | 1.041107 | 6.926791 | 10.16436 | 3.09E-15 | 3.17E-13 | 24.36119 |
| RPS6KB2 | -2.08887 | 8.702825 | -10.1619 | 3.13E-15 | 3.19E-13 | 24.35135 |
| ANXA2P2 | 0.928284 | 11.96947 | 10.15802 | 3.17E-15 | 3.22E-13 | 24.33591 |
| GPS1 | -2.08098 | 8.867449 | -10.1527 | 3.24E-15 | 3.28E-13 | 24.31454 |
| FAM90A7 | 1.800898 | 7.369699 | 10.15043 | 3.27E-15 | 3.29E-13 | 24.30566 |
| SYNPO2L | 1.748122 | 7.181263 | 10.14585 | 3.33E-15 | 3.32E-13 | 24.28742 |
| CIDEA | 1.056273 | 7.106813 | 10.14584 | 3.34E-15 | 3.32E-13 | 24.28737 |
| SPATA12 | 1.518246 | 7.117608 | 10.13048 | 3.55E-15 | 3.51E-13 | 24.22619 |
| SRRM4 | 1.904547 | 7.287237 | 10.12827 | 3.58E-15 | 3.53E-13 | 24.21736 |
| IQCA1 | 1.181088 | 7.088023 | 10.12267 | 3.66E-15 | 3.59E-13 | 24.19503 |
| TRABD | -2.34688 | 8.829062 | -10.1127 | 3.81E-15 | 3.72E-13 | 24.15531 |
| CDKN2AIF | 1.765749 | 9.078609 | 10.11052 | 3.85E-15 | 3.74E-13 | 24.14661 |
| SYCP1 | 1.017086 | 6.809568 | 10.10113 | 4.00E-15 | 3.87E-13 | 24.10917 |
| ATP6V0A1 | -1.98845 | 8.894063 | -10.0981 | 4.05E-15 | 3.87E-13 | 24.09717 |
| PPIL3 | 0.979609 | 9.495558 | 10.09744 | 4.06E-15 | 3.87E-13 | 24.09445 |
| ST8SIA5 | 2.547243 | 7.941245 | 10.09736 | 4.06E-15 | 3.87E-13 | 24.09412 |
| ATP13A1 | -1.89998 | 8.903917 | -10.0963 | 4.08E-15 | 3.87E-13 | 24.0899 |
| RN7SK | -0.92707 | 12.30128 | -10.0937 | 4.12E-15 | 3.89E-13 | 24.07958 |
| FLOT2 | -2.0379 | 11.17673 | -10.0906 | 4.17E-15 | 3.92E-13 | 24.06722 |
| FAM22G | 2.337793 | 7.443153 | 10.08954 | 4.19E-15 | 3.92E-13 | 24.06293 |
| SORBS1 | 1.290391 | 8.669906 | 10.0787 | 4.38E-15 | 4.08E-13 | 24.01968 |
| CACNG6 | 1.490759 | 7.193631 | 10.06695 | 4.59E-15 | 4.25E-13 | 23.97276 |
| N4BP2 | 1.399859 | 8.947696 | 10.06664 | 4.60E-15 | 4.25E-13 | 23.97153 |
| RALGAPB | -1.69474 | 7.986822 | -10.0627 | 4.67E-15 | 4.30E-13 | 23.95567 |
| RIBC1 | 1.046776 | 7.024498 | 10.05927 | 4.74E-15 | 4.34E-13 | 23.9421 |
| FSHR | 1.420291 | 7.035543 | 10.05656 | 4.79E-15 | 4.34E-13 | 23.93129 |
| GTF2IRD2 | 1.330184 | 11.50622 | 10.05609 | 4.80E-15 | 4.34E-13 | 23.92941 |
| FLJ41562 | 2.320347 | 7.755108 | 10.05584 | 4.80E-15 | 4.34E-13 | 23.92843 |
| SLFN13 | 1.892621 | 8.563985 | 10.0491 | 4.94E-15 | 4.44E-13 | 23.90152 |

| | | | | | | |
|---|---|---|---|---|---|---|
| CAPNS1 | -1.28868 | 9.55734 | -10.0421 | 5.08E-15 | 4.55E-13 | 23.87371 |
| CXXC1 | -2.22452 | 8.911195 | -10.036 | 5.21E-15 | 4.64E-13 | 23.84929 |
| EPN1 | -2.6424 | 10.20665 | -10.0225 | 5.50E-15 | 4.89E-13 | 23.79529 |
| UBR5 | -1.11678 | 7.678105 | -10.0185 | 5.59E-15 | 4.94E-13 | 23.77929 |
| ODZ3 | 3.500715 | 9.083503 | 10.00758 | 5.84E-15 | 5.15E-13 | 23.73565 |
| ECHDC2 | -1.8686 | 10.51015 | -9.99746 | 6.09E-15 | 5.33E-13 | 23.69519 |
| SNORA17 | -2.49072 | 9.050207 | -9.99694 | 6.10E-15 | 5.33E-13 | 23.69309 |
| KIR2DS5 | 2.371496 | 7.715087 | 9.99523 | 6.15E-15 | 5.34E-13 | 23.68626 |
| SNORD21 | -2.12979 | 8.716481 | -9.9842 | 6.43E-15 | 5.57E-13 | 23.64216 |
| WWP1 | -1.80265 | 8.18367 | -9.9597 | 7.10E-15 | 6.10E-13 | 23.54415 |
| TGFBI | -1.60468 | 8.413029 | -9.95969 | 7.10E-15 | 6.10E-13 | 23.54413 |
| PBOV1 | 1.492463 | 7.140936 | 9.95017 | 7.38E-15 | 6.31E-13 | 23.50602 |
| ZNF831 | 1.287725 | 7.322826 | 9.948754 | 7.42E-15 | 6.32E-13 | 23.50036 |
| HINT1 | -1.56107 | 7.83408 | -9.94545 | 7.52E-15 | 6.38E-13 | 23.48713 |
| HLA-E | -2.12094 | 9.82419 | -9.9371 | 7.78E-15 | 6.58E-13 | 23.45372 |
| SNORA54 | -2.789 | 9.889056 | -9.93547 | 7.84E-15 | 6.58E-13 | 23.44719 |
| BRD9 | -1.32632 | 8.509624 | -9.93496 | 7.85E-15 | 6.58E-13 | 23.44514 |
| MIIP | -2.43751 | 8.503864 | -9.92782 | 8.08E-15 | 6.75E-13 | 23.41656 |
| PRAMEF2( | 0.938943 | 6.844893 | 9.922329 | 8.27E-15 | 6.87E-13 | 23.39457 |
| CDH18 | 1.228514 | 7.028097 | 9.921163 | 8.31E-15 | 6.88E-13 | 23.3899 |
| GABPA | -1.23542 | 7.71876 | -9.91243 | 8.61E-15 | 7.07E-13 | 23.35494 |
| TDRD1 | 1.568761 | 7.430736 | 9.912353 | 8.61E-15 | 7.07E-13 | 23.35462 |
| KIAA1409 | 0.912976 | 6.893229 | 9.906682 | 8.81E-15 | 7.21E-13 | 23.3319 |
| CLINT1 | -0.87505 | 7.1894 | -9.90513 | 8.87E-15 | 7.22E-13 | 23.32569 |
| OR52R1 | 2.607953 | 7.748379 | 9.900627 | 9.03E-15 | 7.33E-13 | 23.30764 |
| SNORA23 | -2.4104 | 10.89238 | -9.89812 | 9.12E-15 | 7.38E-13 | 23.29761 |
| UNC84B | -2.16942 | 9.559035 | -9.8702 | 1.02E-14 | 8.23E-13 | 23.18571 |
| CDK10 | -1.12226 | 7.918835 | -9.86523 | 1.04E-14 | 8.37E-13 | 23.1658 |
| DCXR | -2.93639 | 10.9055 | -9.86162 | 1.06E-14 | 8.46E-13 | 23.15131 |
| SIM2 | 1.422184 | 7.18795 | 9.854645 | 1.09E-14 | 8.67E-13 | 23.12334 |
| GRM3 | 2.619464 | 7.738399 | 9.853037 | 1.10E-14 | 8.69E-13 | 23.1169 |
| FLJ32658 | 2.658184 | 7.76578 | 9.851894 | 1.10E-14 | 8.70E-13 | 23.11231 |
| RNF126P1 | 1.854704 | 7.502163 | 9.850244 | 1.11E-14 | 8.73E-13 | 23.10569 |
| RANGAP1 | -1.60586 | 8.325161 | -9.84899 | 1.11E-14 | 8.74E-13 | 23.10066 |
| DDX51 | 1.485799 | 9.675541 | 9.846443 | 1.13E-14 | 8.79E-13 | 23.09045 |
| OR51F2 | 1.053063 | 6.944768 | 9.841065 | 1.15E-14 | 8.96E-13 | 23.06888 |
| KLK7 | 1.476048 | 7.43754 | 9.838031 | 1.17E-14 | 9.02E-13 | 23.05671 |
| EIF3K | -1.8007 | 8.768294 | -9.83743 | 1.17E-14 | 9.02E-13 | 23.05428 |
| ZNF577 | 1.140332 | 7.622939 | 9.833558 | 1.19E-14 | 9.13E-13 | 23.03876 |
| NCLN | -1.62959 | 8.100855 | -9.82089 | 1.25E-14 | 9.58E-13 | 22.98795 |
| OR52K1 | 2.753231 | 7.993685 | 9.817431 | 1.27E-14 | 9.68E-13 | 22.97405 |
| C1orf152 | 1.298797 | 7.867232 | 9.815214 | 1.28E-14 | 9.72E-13 | 22.96515 |
| RPS6 | -2.31597 | 9.268524 | -9.8147 | 1.28E-14 | 9.72E-13 | 22.96309 |
| NONO | -1.46797 | 8.492686 | -9.81179 | 1.30E-14 | 9.80E-13 | 22.95143 |
| STAR | 0.984729 | 7.178052 | 9.809675 | 1.31E-14 | 9.85E-13 | 22.94292 |
| DSCR8 | 1.886049 | 7.672723 | 9.806434 | 1.33E-14 | 9.94E-13 | 22.92991 |
| LOC44057 | 3.270413 | 8.363861 | 9.803504 | 1.34E-14 | 1.00E-12 | 22.91815 |
| SLC4A8 | 1.449188 | 7.346024 | 9.796515 | 1.38E-14 | 1.03E-12 | 22.89009 |
| C1orf84 | 1.397175 | 7.735103 | 9.791167 | 1.41E-14 | 1.05E-12 | 22.86862 |
| OR2G3 | 1.637207 | 7.236678 | 9.789952 | 1.42E-14 | 1.05E-12 | 22.86374 |
| C14orf153 | 2.352365 | 9.62728 | 9.785672 | 1.44E-14 | 1.06E-12 | 22.84655 |
| MRPS12 | -1.09938 | 8.03142 | -9.78132 | 1.47E-14 | 1.07E-12 | 22.82909 |
| KIAA0101 | 0.916543 | 10.70817 | 9.781217 | 1.47E-14 | 1.07E-12 | 22.82867 |
| OPA3 | 0.815748 | 7.913877 | 9.781181 | 1.47E-14 | 1.07E-12 | 22.82852 |
| SNORD12 | -1.73137 | 8.050559 | -9.78088 | 1.47E-14 | 1.07E-12 | 22.82732 |
| PSG1 | 1.152851 | 6.917725 | 9.772001 | 1.53E-14 | 1.10E-12 | 22.79165 |
| OR2J3 | 1.668515 | 7.249474 | 9.770138 | 1.54E-14 | 1.11E-12 | 22.78416 |
| RPLP1 | -1.24823 | 7.793872 | -9.76196 | 1.59E-14 | 1.14E-12 | 22.75131 |

| | | | | | | |
|---|---|---|---|---|---|---|
| PFN1 | -2.52289 | 9.570666 | -9.76186 | 1.59E-14 | 1.14E-12 | 22.75092 |
| AURKAIP1 | -2.36819 | 8.765447 | -9.75852 | 1.61E-14 | 1.15E-12 | 22.73748 |
| EML1 | 1.49193 | 7.709333 | 9.758087 | 1.61E-14 | 1.15E-12 | 22.73575 |
| TAX1BP1 | -1.27333 | 9.254535 | -9.75622 | 1.63E-14 | 1.15E-12 | 22.72825 |
| UCP3 | 0.982886 | 7.274692 | 9.754107 | 1.64E-14 | 1.16E-12 | 22.71975 |
| DMRTC2 | 2.149975 | 7.399389 | 9.748125 | 1.68E-14 | 1.18E-12 | 22.69572 |
| GRINA | -1.08121 | 7.972809 | -9.74778 | 1.68E-14 | 1.18E-12 | 22.69435 |
| C17orf70 | -2.16869 | 9.740851 | -9.74773 | 1.68E-14 | 1.18E-12 | 22.69412 |
| NF1 | -1.33079 | 7.569516 | -9.74422 | 1.71E-14 | 1.19E-12 | 22.68002 |
| ALG8 | 1.361175 | 8.647993 | 9.740341 | 1.74E-14 | 1.20E-12 | 22.66443 |
| WDR17 | 1.338466 | 7.362855 | 9.740113 | 1.74E-14 | 1.20E-12 | 22.66352 |
| ZNF554 | 2.093447 | 8.614178 | 9.732229 | 1.79E-14 | 1.24E-12 | 22.63182 |
| CTRB2 | 2.060767 | 7.625807 | 9.730197 | 1.81E-14 | 1.24E-12 | 22.62365 |
| TMEM205 | -3.13373 | 10.40872 | -9.72949 | 1.82E-14 | 1.24E-12 | 22.62082 |
| HARS | -1.80932 | 8.70735 | -9.72741 | 1.83E-14 | 1.25E-12 | 22.61244 |
| OR2A5 | 1.876675 | 7.280121 | 9.726162 | 1.84E-14 | 1.25E-12 | 22.60743 |
| DKFZp451 | 1.969978 | 7.423445 | 9.720431 | 1.88E-14 | 1.27E-12 | 22.58439 |
| ZNF320 | 1.403577 | 8.195516 | 9.719728 | 1.89E-14 | 1.27E-12 | 22.58156 |
| CSF2RA | 1.294274 | 7.879167 | 9.714242 | 1.93E-14 | 1.30E-12 | 22.5595 |
| CXCR5 | 1.069074 | 7.101661 | 9.707086 | 1.99E-14 | 1.33E-12 | 22.53072 |
| ZNF192 | 1.58825 | 8.050702 | 9.704087 | 2.01E-14 | 1.34E-12 | 22.51866 |
| ALOX12B | 3.587038 | 8.476877 | 9.70262 | 2.03E-14 | 1.35E-12 | 22.51276 |
| ALDH1A1 | -2.62905 | 9.586376 | -9.7007 | 2.04E-14 | 1.35E-12 | 22.50504 |
| MSTO2P | 1.467629 | 7.763073 | 9.700497 | 2.04E-14 | 1.35E-12 | 22.50422 |
| TM9SF2 | -1.90914 | 8.588597 | -9.69171 | 2.12E-14 | 1.39E-12 | 22.46888 |
| JMJD8 | -2.25274 | 8.96764 | -9.69165 | 2.12E-14 | 1.39E-12 | 22.46862 |
| MIST | 2.597113 | 7.841493 | 9.669417 | 2.32E-14 | 1.52E-12 | 22.37915 |
| OPN1LW | 1.833984 | 7.261954 | 9.667772 | 2.34E-14 | 1.53E-12 | 22.37254 |
| CYMP | 1.7069 | 7.446094 | 9.666253 | 2.35E-14 | 1.53E-12 | 22.36642 |
| ARL2 | -1.6314 | 8.951579 | -9.66372 | 2.38E-14 | 1.54E-12 | 22.35622 |
| OR11G2 | 1.26691 | 7.107483 | 9.662575 | 2.39E-14 | 1.54E-12 | 22.35162 |
| SEC23B | -0.91167 | 7.529425 | -9.6611 | 2.40E-14 | 1.55E-12 | 22.34566 |
| GPX3 | -1.58821 | 12.03957 | -9.65566 | 2.45E-14 | 1.58E-12 | 22.32376 |
| SON | -0.92097 | 8.198208 | -9.64686 | 2.54E-14 | 1.63E-12 | 22.28833 |
| PTOV1 | -1.85324 | 9.007546 | -9.63497 | 2.67E-14 | 1.71E-12 | 22.24044 |
| SNORA51 | -2.44593 | 10.26932 | -9.63056 | 2.72E-14 | 1.73E-12 | 22.22269 |
| LOC40211 | 1.209265 | 7.169516 | 9.626622 | 2.76E-14 | 1.76E-12 | 22.20683 |
| LOC10013 | 0.769021 | 7.028187 | 9.622678 | 2.81E-14 | 1.77E-12 | 22.19095 |
| JUND | -1.4723 | 11.90356 | -9.62252 | 2.81E-14 | 1.77E-12 | 22.1903 |
| C1orf86 | -1.09609 | 7.877004 | -9.62085 | 2.83E-14 | 1.78E-12 | 22.18357 |
| DUX3 | 3.457643 | 8.587064 | 9.620462 | 2.84E-14 | 1.78E-12 | 22.18202 |
| ZNF695 | 1.321335 | 7.207065 | 9.616239 | 2.88E-14 | 1.80E-12 | 22.165 |
| CENPB | -1.96897 | 8.594393 | -9.61596 | 2.89E-14 | 1.80E-12 | 22.16389 |
| DNHL1 | 2.469548 | 9.170631 | 9.611204 | 2.94E-14 | 1.83E-12 | 22.14471 |
| MACC1 | 0.971825 | 7.042485 | 9.609115 | 2.97E-14 | 1.84E-12 | 22.1363 |
| CNN2 | -1.30378 | 8.890941 | -9.60333 | 3.04E-14 | 1.88E-12 | 22.11297 |
| PIGW | 1.614167 | 7.938884 | 9.598925 | 3.10E-14 | 1.91E-12 | 22.09523 |
| FCAR | 1.179224 | 7.173147 | 9.596611 | 3.13E-14 | 1.92E-12 | 22.08591 |
| CSNK2B | -2.36704 | 8.725347 | -9.59583 | 3.14E-14 | 1.92E-12 | 22.08275 |
| PDE6B | 1.204966 | 7.007567 | 9.593496 | 3.17E-14 | 1.93E-12 | 22.07335 |
| SNORD11 | -2.25415 | 8.969163 | -9.59073 | 3.20E-14 | 1.95E-12 | 22.06219 |
| KLK13 | 1.866293 | 7.274535 | 9.590222 | 3.21E-14 | 1.95E-12 | 22.06015 |
| PRELP | 0.741714 | 6.939492 | 9.585132 | 3.28E-14 | 1.98E-12 | 22.03963 |
| DCLRE1C | 1.120682 | 7.388992 | 9.579551 | 3.35E-14 | 2.02E-12 | 22.01713 |
| C12orf68 | 1.058372 | 7.374453 | 9.573518 | 3.44E-14 | 2.06E-12 | 21.99281 |
| OR2A1 | 2.186312 | 7.956803 | 9.573068 | 3.44E-14 | 2.06E-12 | 21.99099 |
| TFAP2D | 1.656122 | 7.195736 | 9.572739 | 3.45E-14 | 2.06E-12 | 21.98967 |
| LALBA | 1.995117 | 7.54774 | 9.560525 | 3.62E-14 | 2.16E-12 | 21.94041 |

| | | | | | | |
|---|---|---|---|---|---|---|
| PGRMC1 | -1.98049 | 8.824442 | -9.55646 | 3.69E-14 | 2.19E-12 | 21.92399 |
| TDRD12 | 1.841281 | 7.364072 | 9.553981 | 3.72E-14 | 2.21E-12 | 21.91401 |
| POM121L | 1.341778 | 7.190219 | 9.553171 | 3.74E-14 | 2.21E-12 | 21.91075 |
| C7orf64 | 1.604537 | 8.52236 | 9.551245 | 3.77E-14 | 2.22E-12 | 21.90298 |
| OR52I1 | 2.193095 | 7.596194 | 9.547269 | 3.83E-14 | 2.25E-12 | 21.88693 |
| TMEM49 | -1.8661 | 8.479561 | -9.54619 | 3.84E-14 | 2.25E-12 | 21.8826 |
| NUCB1 | -2.30208 | 10.82701 | -9.54203 | 3.91E-14 | 2.29E-12 | 21.86579 |
| RGPD1 | 1.646865 | 7.320068 | 9.540219 | 3.94E-14 | 2.29E-12 | 21.85849 |
| LMNA | -0.88625 | 7.333627 | -9.53978 | 3.95E-14 | 2.29E-12 | 21.85673 |
| STEAP3 | 1.254739 | 8.858931 | 9.537657 | 3.98E-14 | 2.31E-12 | 21.84815 |
| BRIP1 | 1.656505 | 8.179971 | 9.533225 | 4.05E-14 | 2.34E-12 | 21.83027 |
| SIGLEC10 | 2.501117 | 8.207547 | 9.5323 | 4.07E-14 | 2.35E-12 | 21.82654 |
| CHRNB4 | 1.200846 | 7.218302 | 9.530201 | 4.10E-14 | 2.36E-12 | 21.81807 |
| MC3R | 1.917502 | 7.363195 | 9.529932 | 4.11E-14 | 2.36E-12 | 21.81698 |
| C5orf28 | 1.23151 | 8.849411 | 9.521915 | 4.25E-14 | 2.43E-12 | 21.78463 |
| SIL1 | -1.80245 | 9.831255 | -9.51512 | 4.37E-14 | 2.48E-12 | 21.75719 |
| OR4K15 | 1.020525 | 6.973795 | 9.514656 | 4.37E-14 | 2.48E-12 | 21.75533 |
| RPUSD4 | -1.57506 | 8.68686 | -9.51408 | 4.39E-14 | 2.48E-12 | 21.75302 |
| EML3 | -2.55513 | 9.987255 | -9.51178 | 4.43E-14 | 2.49E-12 | 21.74371 |
| OXA1L | -2.07589 | 8.327847 | -9.51171 | 4.43E-14 | 2.49E-12 | 21.74345 |
| OR4F29 | 1.764423 | 7.501372 | 9.504531 | 4.56E-14 | 2.56E-12 | 21.71445 |
| INSC | 1.481914 | 7.503308 | 9.499829 | 4.65E-14 | 2.60E-12 | 21.69547 |
| YIF1B | -0.81628 | 7.475991 | -9.49833 | 4.68E-14 | 2.61E-12 | 21.6894 |
| DLL3 | 1.933451 | 7.373946 | 9.495755 | 4.73E-14 | 2.63E-12 | 21.67902 |
| NNAT | 1.555359 | 7.158112 | 9.492493 | 4.79E-14 | 2.66E-12 | 21.66585 |
| POU6F2 | 2.455729 | 8.030717 | 9.488028 | 4.88E-14 | 2.70E-12 | 21.64781 |
| TAF1D | -0.79388 | 7.128606 | -9.47935 | 5.06E-14 | 2.79E-12 | 21.61276 |
| DUSP23 | -1.5909 | 9.561362 | -9.47879 | 5.07E-14 | 2.79E-12 | 21.61049 |
| PRSS2 | 1.117703 | 7.359159 | 9.471765 | 5.22E-14 | 2.86E-12 | 21.58213 |
| SNORD34 | -1.90888 | 8.520732 | -9.46941 | 5.27E-14 | 2.88E-12 | 21.57262 |
| C8orf45 | 1.330125 | 7.750643 | 9.467688 | 5.30E-14 | 2.90E-12 | 21.56566 |
| CRYBA4 | 1.576138 | 7.248619 | 9.463928 | 5.39E-14 | 2.93E-12 | 21.55047 |
| FAM40B | 1.685918 | 9.388383 | 9.463303 | 5.40E-14 | 2.93E-12 | 21.54794 |
| HS6ST3 | 2.163494 | 7.492262 | 9.46258 | 5.42E-14 | 2.94E-12 | 21.54502 |
| EXOSC6 | -1.86232 | 8.950101 | -9.45993 | 5.48E-14 | 2.95E-12 | 21.53433 |
| INTS1 | -1.01204 | 7.500726 | -9.45805 | 5.52E-14 | 2.97E-12 | 21.5267 |
| C10orf4 | 1.588366 | 8.258758 | 9.45176 | 5.66E-14 | 3.04E-12 | 21.5013 |
| C9orf37 | -1.2318 | 7.781064 | -9.45067 | 5.69E-14 | 3.04E-12 | 21.4969 |
| UTP20 | 2.014723 | 8.906726 | 9.450214 | 5.70E-14 | 3.04E-12 | 21.49505 |
| CEBPA | -2.03276 | 8.912376 | -9.44681 | 5.78E-14 | 3.08E-12 | 21.48131 |
| PRAC | 1.735602 | 7.257692 | 9.443793 | 5.85E-14 | 3.11E-12 | 21.4691 |
| OR7E91P | 3.137668 | 8.274594 | 9.442707 | 5.88E-14 | 3.11E-12 | 21.46471 |
| HIST1H2B | -1.12699 | 8.039109 | -9.43614 | 6.04E-14 | 3.19E-12 | 21.43817 |
| LYZL4 | 1.173699 | 6.851103 | 9.435016 | 6.07E-14 | 3.19E-12 | 21.43362 |
| DIRC1 | 1.525048 | 7.241136 | 9.434066 | 6.09E-14 | 3.20E-12 | 21.42978 |
| ACTR1A | -1.76542 | 8.18795 | -9.43118 | 6.16E-14 | 3.23E-12 | 21.41813 |
| MAG | 1.703364 | 7.473504 | 9.430549 | 6.18E-14 | 3.23E-12 | 21.41556 |
| OR10V1 | 2.74204 | 7.739368 | 9.428759 | 6.22E-14 | 3.25E-12 | 21.40833 |
| SNORD33 | -3.07903 | 11.68565 | -9.42583 | 6.30E-14 | 3.28E-12 | 21.39651 |
| ALG3 | -1.28703 | 8.447574 | -9.42432 | 6.34E-14 | 3.29E-12 | 21.39038 |
| FMO9P | 0.909422 | 6.902654 | 9.422895 | 6.38E-14 | 3.30E-12 | 21.38462 |
| RCHY1 | 1.25514 | 7.698288 | 9.410902 | 6.70E-14 | 3.46E-12 | 21.33613 |
| PTK2 | -1.02125 | 7.600035 | -9.40684 | 6.81E-14 | 3.51E-12 | 21.31969 |
| OR2A42 | 1.355642 | 7.388959 | 9.406138 | 6.83E-14 | 3.51E-12 | 21.31686 |
| OR5AR1 | 1.840741 | 7.349299 | 9.397709 | 7.07E-14 | 3.61E-12 | 21.28277 |
| C7orf38 | 1.36705 | 8.006599 | 9.396351 | 7.11E-14 | 3.63E-12 | 21.27728 |
| GNB1 | -1.49193 | 8.70928 | -9.39449 | 7.17E-14 | 3.64E-12 | 21.26976 |
| C10orf99 | 2.086902 | 7.457319 | 9.393349 | 7.20E-14 | 3.65E-12 | 21.26514 |

| | | | | | | |
|---|---|---|---|---|---|---|
| MCRS1 | -0.83114 | 7.827266 | -9.38417 | 7.48E-14 | 3.78E-12 | 21.228 |
| ATP5H | -1.89748 | 8.95526 | -9.38192 | 7.55E-14 | 3.80E-12 | 21.21891 |
| UCA1 | 3.061976 | 9.395022 | 9.381524 | 7.56E-14 | 3.80E-12 | 21.2173 |
| AIRE | 1.91551 | 9.679069 | 9.378542 | 7.65E-14 | 3.83E-12 | 21.20523 |
| IGFL3 | 3.385386 | 8.886945 | 9.378216 | 7.66E-14 | 3.83E-12 | 21.20391 |
| CCKBR | 1.143519 | 6.970806 | 9.372892 | 7.83E-14 | 3.90E-12 | 21.18237 |
| DUX5 | 3.415283 | 8.613763 | 9.372713 | 7.84E-14 | 3.90E-12 | 21.18165 |
| KRT3 | 2.132391 | 7.492856 | 9.369226 | 7.95E-14 | 3.95E-12 | 21.16754 |
| PLA2G2D | 1.580283 | 9.010731 | 9.36773 | 8.00E-14 | 3.96E-12 | 21.16148 |
| DRD3 | 2.284833 | 7.630034 | 9.3661 | 8.05E-14 | 3.98E-12 | 21.15488 |
| DEFB122 | 1.464904 | 7.140291 | 9.359372 | 8.28E-14 | 4.08E-12 | 21.12766 |
| IL29 | 1.154266 | 6.992994 | 9.355002 | 8.43E-14 | 4.14E-12 | 21.10997 |
| EIF3H | -2.36834 | 8.83304 | -9.35354 | 8.48E-14 | 4.15E-12 | 21.10406 |
| BCAS4 | 1.007224 | 7.941647 | 9.353357 | 8.49E-14 | 4.15E-12 | 21.10331 |
| NR1H3 | -2.13093 | 9.236154 | -9.35263 | 8.51E-14 | 4.16E-12 | 21.10036 |
| TEX11 | 1.084129 | 10.41372 | 9.350432 | 8.59E-14 | 4.18E-12 | 21.09147 |
| ZBTB48 | -2.30707 | 9.850844 | -9.35011 | 8.60E-14 | 4.18E-12 | 21.09018 |
| KCTD8 | 1.479997 | 7.30982 | 9.348194 | 8.67E-14 | 4.20E-12 | 21.08241 |
| KRTAP5-4 | 0.97494 | 6.838577 | 9.347829 | 8.68E-14 | 4.20E-12 | 21.08093 |
| IL23A | 1.781127 | 7.666675 | 9.345223 | 8.78E-14 | 4.23E-12 | 21.07038 |
| MIOX | 2.760629 | 7.963704 | 9.344947 | 8.79E-14 | 4.23E-12 | 21.06926 |
| XIRP2 | 2.439715 | 7.550709 | 9.341416 | 8.91E-14 | 4.28E-12 | 21.05497 |
| USP49 | 1.764377 | 9.026397 | 9.339495 | 8.99E-14 | 4.30E-12 | 21.04719 |
| ADIPOQ | 2.119139 | 7.60618 | 9.337536 | 9.06E-14 | 4.33E-12 | 21.03926 |
| NCRNA00 | 1.051832 | 7.031188 | 9.335298 | 9.14E-14 | 4.36E-12 | 21.0302 |
| CFH | -1.70288 | 8.206748 | -9.33175 | 9.28E-14 | 4.41E-12 | 21.01583 |
| GLRX5 | -1.59849 | 8.737994 | -9.33124 | 9.30E-14 | 4.41E-12 | 21.01377 |
| DAPP1 | 1.17579 | 11.21065 | 9.323798 | 9.58E-14 | 4.54E-12 | 20.98363 |
| TCEB3CL | 2.097397 | 7.613707 | 9.32212 | 9.65E-14 | 4.56E-12 | 20.97684 |
| CSRNP3 | 1.316576 | 7.523956 | 9.320986 | 9.70E-14 | 4.57E-12 | 20.97225 |
| MRPL37 | -2.31207 | 9.428487 | -9.31829 | 9.80E-14 | 4.61E-12 | 20.96133 |
| NFE2L2 | -1.25312 | 7.557986 | -9.3161 | 9.89E-14 | 4.64E-12 | 20.95244 |
| NCOA4 | -1.82675 | 9.219488 | -9.31551 | 9.92E-14 | 4.64E-12 | 20.95006 |
| USP17 | 2.943522 | 8.392874 | 9.314218 | 9.97E-14 | 4.66E-12 | 20.94483 |
| SEC13 | -0.90388 | 7.258075 | -9.31046 | 1.01E-13 | 4.71E-12 | 20.92961 |
| ZRANB2 | -1.00274 | 8.95686 | -9.31006 | 1.01E-13 | 4.71E-12 | 20.92798 |
| LSM14B | -1.88838 | 8.147327 | -9.30997 | 1.01E-13 | 4.71E-12 | 20.92763 |
| SIPA1 | -1.55768 | 7.977916 | -9.30553 | 1.03E-13 | 4.78E-12 | 20.90963 |
| TMED9 | -1.08909 | 7.866991 | -9.30432 | 1.04E-13 | 4.80E-12 | 20.90475 |
| THBS3 | -1.33496 | 7.76983 | -9.30352 | 1.04E-13 | 4.80E-12 | 20.90152 |
| DOPEY2 | 1.188704 | 10.47623 | 9.300086 | 1.06E-13 | 4.86E-12 | 20.88759 |
| C5orf32 | -1.48573 | 8.391509 | -9.29871 | 1.06E-13 | 4.88E-12 | 20.88202 |
| C15orf32 | 1.564763 | 7.142886 | 9.292671 | 1.09E-13 | 4.98E-12 | 20.85755 |
| BEND3 | 1.141988 | 7.447545 | 9.287928 | 1.11E-13 | 5.06E-12 | 20.83833 |
| OR2L1P | 0.965931 | 6.89249 | 9.285868 | 1.12E-13 | 5.09E-12 | 20.82998 |
| LINGO2 | 1.737643 | 7.137561 | 9.28556 | 1.12E-13 | 5.09E-12 | 20.82873 |
| IL1RAPL1 | 2.32699 | 7.724535 | 9.283699 | 1.13E-13 | 5.12E-12 | 20.82119 |
| OR6S1 | 1.339333 | 7.01718 | 9.281246 | 1.14E-13 | 5.15E-12 | 20.81125 |
| ATP1A1 | -1.58932 | 8.314635 | -9.28073 | 1.14E-13 | 5.15E-12 | 20.80915 |
| MOBKL2A | -1.31416 | 7.921475 | -9.28063 | 1.14E-13 | 5.15E-12 | 20.80874 |
| KRT1 | 1.200275 | 6.988197 | 9.279155 | 1.15E-13 | 5.17E-12 | 20.80278 |
| FBP2 | 1.707963 | 7.388415 | 9.278587 | 1.15E-13 | 5.17E-12 | 20.80048 |
| PFDN5 | -1.11455 | 7.85917 | -9.27522 | 1.17E-13 | 5.23E-12 | 20.78682 |
| SNORA22 | -1.62431 | 8.280215 | -9.27144 | 1.19E-13 | 5.30E-12 | 20.77153 |
| FIBP | -2.05666 | 9.018689 | -9.27077 | 1.19E-13 | 5.30E-12 | 20.76878 |
| LOC44126 | 2.104833 | 8.190718 | 9.270732 | 1.19E-13 | 5.30E-12 | 20.76864 |
| S100A7A | 0.957441 | 6.906285 | 9.269983 | 1.20E-13 | 5.30E-12 | 20.7656 |
| ZNF669 | 0.812245 | 7.507708 | 9.269409 | 1.20E-13 | 5.30E-12 | 20.76328 |

| | | | | | | |
|---|---|---|---|---|---|---|
| CDH10 | 1.917213 | 7.423308 | 9.267458 | 1.21E-13 | 5.33E-12 | 20.75537 |
| OTOP2 | 1.846029 | 7.352754 | 9.265814 | 1.22E-13 | 5.36E-12 | 20.74871 |
| AK2 | -0.88131 | 7.979785 | -9.2639 | 1.23E-13 | 5.38E-12 | 20.74097 |
| CLTB | -1.48468 | 10.30098 | -9.26373 | 1.23E-13 | 5.38E-12 | 20.74025 |
| C18orf26 | 1.155943 | 7.126002 | 9.262353 | 1.23E-13 | 5.40E-12 | 20.73468 |
| RPL23 | -1.70507 | 8.377095 | -9.26201 | 1.24E-13 | 5.40E-12 | 20.73331 |
| VENTXP7 | 2.146187 | 7.468905 | 9.25995 | 1.25E-13 | 5.42E-12 | 20.72494 |
| OR10G9 | 1.454261 | 7.128615 | 9.259897 | 1.25E-13 | 5.42E-12 | 20.72472 |
| RPL19 | -1.94828 | 9.919259 | -9.25635 | 1.27E-13 | 5.49E-12 | 20.71034 |
| SGSM2 | -0.99033 | 7.352817 | -9.25448 | 1.28E-13 | 5.52E-12 | 20.70278 |
| SHCBP1 | 1.707199 | 9.554767 | 9.254181 | 1.28E-13 | 5.52E-12 | 20.70155 |
| MAP1LC3( | 2.67189 | 7.937511 | 9.253406 | 1.28E-13 | 5.52E-12 | 20.69841 |
| LGMN | 0.97645 | 10.25264 | 9.2528 | 1.28E-13 | 5.52E-12 | 20.69595 |
| CCDC29 | 1.460274 | 7.685242 | 9.248736 | 1.31E-13 | 5.61E-12 | 20.67947 |
| HNRPDL | -0.71406 | 7.217643 | -9.24232 | 1.34E-13 | 5.74E-12 | 20.65344 |
| KCNN2 | 1.683229 | 7.665159 | 9.236095 | 1.38E-13 | 5.87E-12 | 20.62822 |
| MACF1 | -1.21433 | 7.630684 | -9.23601 | 1.38E-13 | 5.87E-12 | 20.62786 |
| SLC30A6 | 1.420641 | 8.247638 | 9.234557 | 1.38E-13 | 5.89E-12 | 20.62198 |
| ATAD3C | 1.38612 | 7.747152 | 9.230656 | 1.41E-13 | 5.98E-12 | 20.60616 |
| CYB5R1 | -1.70426 | 8.122694 | -9.22853 | 1.42E-13 | 6.02E-12 | 20.59754 |
| VPS29 | -1.11798 | 8.09437 | -9.22412 | 1.45E-13 | 6.12E-12 | 20.57963 |
| PNPLA6 | -1.25133 | 8.480922 | -9.22064 | 1.47E-13 | 6.19E-12 | 20.56553 |
| LOC38983 | 1.532939 | 7.423795 | 9.216614 | 1.49E-13 | 6.28E-12 | 20.54921 |
| SMAD4 | -0.75576 | 7.153668 | -9.2157 | 1.50E-13 | 6.29E-12 | 20.54549 |
| TYK2 | -1.89065 | 8.741466 | -9.21097 | 1.53E-13 | 6.40E-12 | 20.52632 |
| MAP1LC3/ | -0.92632 | 7.746809 | -9.19917 | 1.60E-13 | 6.71E-12 | 20.47842 |
| NAMPT | -1.93743 | 8.824906 | -9.19785 | 1.61E-13 | 6.73E-12 | 20.47308 |
| CLDND2 | -1.11577 | 7.509717 | -9.19732 | 1.61E-13 | 6.73E-12 | 20.47092 |
| ATP6V0E2 | -2.06146 | 9.431895 | -9.1911 | 1.66E-13 | 6.90E-12 | 20.44568 |
| SLC10A2 | 1.272435 | 7.024375 | 9.189766 | 1.67E-13 | 6.92E-12 | 20.44028 |
| SNRP70 | -1.12903 | 7.596501 | -9.18616 | 1.69E-13 | 7.01E-12 | 20.42565 |
| RAB7A | -1.13633 | 8.549771 | -9.1847 | 1.70E-13 | 7.04E-12 | 20.41972 |
| LCE6A | 2.355362 | 7.748126 | 9.175014 | 1.77E-13 | 7.31E-12 | 20.38041 |
| C5orf49 | 0.741701 | 7.001146 | 9.169171 | 1.81E-13 | 7.47E-12 | 20.35669 |
| OR52J3 | 1.264912 | 7.00671 | 9.16693 | 1.83E-13 | 7.52E-12 | 20.34759 |
| CPSF1 | -1.67101 | 9.774081 | -9.16654 | 1.83E-13 | 7.52E-12 | 20.34602 |
| LOC64395 | 1.008339 | 7.004252 | 9.166052 | 1.84E-13 | 7.52E-12 | 20.34403 |
| SKIL | -0.93202 | 7.439905 | -9.16286 | 1.86E-13 | 7.61E-12 | 20.33107 |
| MTHFD2 | 1.698345 | 8.390386 | 9.155802 | 1.92E-13 | 7.82E-12 | 20.30242 |
| CEACAM1 | 1.529434 | 7.199279 | 9.149702 | 1.96E-13 | 8.00E-12 | 20.27765 |
| CRNN | 1.009361 | 6.908538 | 9.149227 | 1.97E-13 | 8.00E-12 | 20.27572 |
| MIR1913 | 1.459478 | 7.309028 | 9.144026 | 2.01E-13 | 8.16E-12 | 20.2546 |
| OR4K14 | 1.18001 | 7.02374 | 9.141698 | 2.03E-13 | 8.22E-12 | 20.24515 |
| CNNM3 | -1.37271 | 8.349624 | -9.14088 | 2.04E-13 | 8.23E-12 | 20.24183 |
| PGBD5 | 2.492678 | 9.027522 | 9.140082 | 2.04E-13 | 8.25E-12 | 20.23859 |
| CLEC9A | 0.755595 | 6.909014 | 9.138991 | 2.05E-13 | 8.27E-12 | 20.23416 |
| REXO1L1 | 3.901643 | 8.972772 | 9.138504 | 2.06E-13 | 8.27E-12 | 20.23218 |
| POU3F2 | 0.785136 | 6.867818 | 9.128768 | 2.14E-13 | 8.59E-12 | 20.19264 |
| GPX5 | 2.010868 | 7.405131 | 9.127023 | 2.16E-13 | 8.63E-12 | 20.18555 |
| SNORD71 | -3.12719 | 10.35469 | -9.12641 | 2.16E-13 | 8.64E-12 | 20.18306 |
| ZC3HAV1I | 0.961071 | 7.56058 | 9.12184 | 2.20E-13 | 8.79E-12 | 20.1645 |
| BRD2 | -1.91272 | 8.639498 | -9.12078 | 2.21E-13 | 8.81E-12 | 20.1602 |
| SCARNA1 | -2.3939 | 9.464414 | -9.11833 | 2.24E-13 | 8.87E-12 | 20.15026 |
| IL17RD | 1.073746 | 7.439914 | 9.118231 | 2.24E-13 | 8.87E-12 | 20.14984 |
| SKA2 | 1.427922 | 7.718341 | 9.116888 | 2.25E-13 | 8.89E-12 | 20.14438 |
| CHRNA1 | 1.494784 | 7.082419 | 9.116662 | 2.25E-13 | 8.89E-12 | 20.14346 |
| IRX2 | 1.299939 | 7.113084 | 9.115777 | 2.26E-13 | 8.91E-12 | 20.13987 |
| FKSG44 | 1.513628 | 9.482091 | 9.114095 | 2.28E-13 | 8.95E-12 | 20.13304 |

| | | | | | | |
|---|---|---|---|---|---|---|
| REXO1L2F | 4.043304 | 9.156899 | 9.11317 | 2.28E-13 | 8.97E-12 | 20.12928 |
| NICN1 | -1.82926 | 8.555798 | -9.10828 | 2.33E-13 | 9.14E-12 | 20.10939 |
| HNRPR | -1.09093 | 8.218099 | -9.1049 | 2.36E-13 | 9.22E-12 | 20.09569 |
| CAPZB | -1.16445 | 8.286324 | -9.10461 | 2.37E-13 | 9.22E-12 | 20.09449 |
| GBX1 | 1.303169 | 7.120527 | 9.100623 | 2.41E-13 | 9.36E-12 | 20.07831 |
| SPC25 | 1.66491 | 8.027941 | 9.100038 | 2.41E-13 | 9.36E-12 | 20.07593 |
| IDH2 | -1.58388 | 8.694229 | -9.09803 | 2.43E-13 | 9.42E-12 | 20.06779 |
| HES4 | -2.01572 | 8.599553 | -9.09575 | 2.45E-13 | 9.49E-12 | 20.05851 |
| DHRS13 | -1.1726 | 7.761686 | -9.09397 | 2.47E-13 | 9.55E-12 | 20.05129 |
| FLJ40434 | 2.84728 | 8.027998 | 9.092966 | 2.48E-13 | 9.57E-12 | 20.04719 |
| PPFIA2 | 2.543899 | 7.914443 | 9.092275 | 2.49E-13 | 9.58E-12 | 20.04439 |
| QRFPR | 1.666287 | 7.876492 | 9.090697 | 2.51E-13 | 9.62E-12 | 20.03797 |
| ERGIC3 | -0.88577 | 7.382834 | -9.08386 | 2.58E-13 | 9.88E-12 | 20.01018 |
| LOC10012 | 1.193574 | 7.487251 | 9.080662 | 2.61E-13 | 9.99E-12 | 19.99719 |
| GALR2 | 1.463754 | 7.271521 | 9.078282 | 2.64E-13 | 1.01E-11 | 19.98752 |
| MLXIP | -1.53218 | 7.799228 | -9.07785 | 2.64E-13 | 1.01E-11 | 19.98576 |
| ATP6AP2 | -1.48096 | 8.201313 | -9.07765 | 2.64E-13 | 1.01E-11 | 19.98494 |
| LOC14995 | 1.529222 | 7.087714 | 9.072943 | 2.70E-13 | 1.02E-11 | 19.96582 |
| UBR2 | -1.00247 | 7.600595 | -9.06767 | 2.76E-13 | 1.04E-11 | 19.94436 |
| SPANXA1 | 1.029571 | 6.891423 | 9.065412 | 2.78E-13 | 1.05E-11 | 19.9352 |
| ATP6AP1 | -1.91888 | 8.354422 | -9.06516 | 2.78E-13 | 1.05E-11 | 19.93416 |
| QKI | -0.7763 | 7.227103 | -9.06359 | 2.80E-13 | 1.06E-11 | 19.92779 |
| MCF2L2 | 1.007048 | 7.403115 | 9.063024 | 2.81E-13 | 1.06E-11 | 19.9255 |
| WDR34 | -1.31581 | 7.635771 | -9.06279 | 2.81E-13 | 1.06E-11 | 19.92453 |
| OR2T27 | 1.905799 | 7.45513 | 9.061975 | 2.82E-13 | 1.06E-11 | 19.92123 |
| SLC4A5 | 1.837221 | 8.831596 | 9.055511 | 2.90E-13 | 1.08E-11 | 19.89495 |
| SPAG6 | 0.787377 | 6.864448 | 9.047382 | 3.00E-13 | 1.12E-11 | 19.8619 |
| WDR1 | -1.1603 | 7.996693 | -9.04613 | 3.01E-13 | 1.12E-11 | 19.8568 |
| GCNT7 | 0.908862 | 6.845324 | 9.042861 | 3.05E-13 | 1.13E-11 | 19.84352 |
| TRIM13 | 0.853023 | 7.657612 | 9.040351 | 3.09E-13 | 1.14E-11 | 19.83331 |
| FAM50A | -2.51076 | 10.7034 | -9.03964 | 3.09E-13 | 1.15E-11 | 19.83044 |
| GH1 | 1.092984 | 6.922913 | 9.033753 | 3.17E-13 | 1.17E-11 | 19.80648 |
| SNORA45 | -2.9224 | 11.55694 | -9.0317 | 3.20E-13 | 1.18E-11 | 19.79812 |
| LYPLAL1 | -1.2381 | 7.588752 | -9.03077 | 3.21E-13 | 1.18E-11 | 19.79437 |
| FOLR1 | 1.406767 | 7.408382 | 9.03009 | 3.22E-13 | 1.18E-11 | 19.79159 |
| C3orf34 | 0.846717 | 7.102702 | 9.029681 | 3.22E-13 | 1.18E-11 | 19.78992 |
| AP1M1 | -1.97973 | 8.366116 | -9.02561 | 3.28E-13 | 1.20E-11 | 19.77336 |
| OR9G4 | 1.29119 | 6.936217 | 9.025377 | 3.28E-13 | 1.20E-11 | 19.77242 |
| KRTCAP2 | -2.10851 | 9.649298 | -9.02419 | 3.30E-13 | 1.20E-11 | 19.76758 |
| SNORA4 | -1.29904 | 7.869051 | -9.02381 | 3.30E-13 | 1.20E-11 | 19.76606 |
| C12orf72 | 1.923237 | 9.183145 | 9.019114 | 3.37E-13 | 1.22E-11 | 19.74694 |
| TMCO1 | -1.80851 | 9.230881 | -9.00838 | 3.52E-13 | 1.27E-11 | 19.70329 |
| C19orf41 | 2.061509 | 7.415742 | 9.00751 | 3.53E-13 | 1.28E-11 | 19.69974 |
| HERPUD1 | -1.15715 | 7.816973 | -9.0069 | 3.54E-13 | 1.28E-11 | 19.69725 |
| NDUFC1 | -1.73288 | 8.103021 | -9.00632 | 3.55E-13 | 1.28E-11 | 19.6949 |
| SC4MOL | 1.430785 | 9.168249 | 9.001527 | 3.62E-13 | 1.30E-11 | 19.6754 |
| CSNK1G2 | -1.22371 | 8.549409 | -9.00069 | 3.64E-13 | 1.30E-11 | 19.672 |
| HIST2H2A | -1.3958 | 7.796319 | -9.00025 | 3.64E-13 | 1.30E-11 | 19.67021 |
| HUS1B | 0.947218 | 7.499449 | 8.999702 | 3.65E-13 | 1.30E-11 | 19.66798 |
| C19orf43 | -0.99626 | 7.676609 | -8.99921 | 3.66E-13 | 1.31E-11 | 19.66598 |
| C6orf153 | -1.31888 | 8.51576 | -8.99838 | 3.67E-13 | 1.31E-11 | 19.6626 |
| H1F0 | -1.93767 | 8.725725 | -8.99605 | 3.71E-13 | 1.32E-11 | 19.65312 |
| BCKDK | -2.12835 | 10.58954 | -8.99554 | 3.71E-13 | 1.32E-11 | 19.65105 |
| PFKL | -0.97856 | 7.713538 | -8.99454 | 3.73E-13 | 1.32E-11 | 19.64696 |
| OKL38 | -2.72155 | 10.07978 | -8.99315 | 3.75E-13 | 1.33E-11 | 19.6413 |
| SNORD16 | -2.39328 | 9.499507 | -8.99249 | 3.76E-13 | 1.33E-11 | 19.63862 |
| FTHL3 | -1.41434 | 8.355998 | -8.99159 | 3.77E-13 | 1.33E-11 | 19.63497 |
| ZNF595 | 2.197387 | 9.877007 | 8.991319 | 3.78E-13 | 1.33E-11 | 19.63387 |

| | | | | | | |
|---|---|---|---|---|---|---|
| REG3G | 1.149051 | 7.029851 | 8.990896 | 3.79E-13 | 1.33E-11 | 19.63215 |
| RPN1 | -1.12245 | 8.121678 | -8.98852 | 3.82E-13 | 1.34E-11 | 19.62247 |
| FRG2B | 1.014829 | 6.966571 | 8.987752 | 3.84E-13 | 1.34E-11 | 19.61935 |
| KIR3DL2 | 2.759197 | 8.090218 | 8.98515 | 3.88E-13 | 1.35E-11 | 19.60876 |
| KIR3DL1 | 1.825414 | 7.383554 | 8.982147 | 3.92E-13 | 1.37E-11 | 19.59655 |
| OR56A4 | 2.466997 | 7.586179 | 8.977127 | 4.01E-13 | 1.39E-11 | 19.57612 |
| MED12 | -1.56299 | 7.715913 | -8.97536 | 4.04E-13 | 1.40E-11 | 19.56894 |
| MAGEA10 | 2.408442 | 7.659293 | 8.96438 | 4.22E-13 | 1.46E-11 | 19.52424 |
| C21orf54 | 2.193716 | 7.564033 | 8.960442 | 4.29E-13 | 1.48E-11 | 19.50821 |
| GPR152 | 3.048556 | 8.289103 | 8.955025 | 4.39E-13 | 1.51E-11 | 19.48616 |
| SNORD11 | -1.95973 | 8.693516 | -8.95486 | 4.39E-13 | 1.51E-11 | 19.48547 |
| PRKCSH | -1.27792 | 8.268447 | -8.95341 | 4.42E-13 | 1.52E-11 | 19.4796 |
| GPR139 | 1.599908 | 7.038897 | 8.949498 | 4.49E-13 | 1.54E-11 | 19.46366 |
| DDX3X | -1.11393 | 7.881928 | -8.94919 | 4.50E-13 | 1.54E-11 | 19.46241 |
| LOC14841 | -1.10815 | 7.812233 | -8.94749 | 4.53E-13 | 1.55E-11 | 19.4555 |
| C19orf15 | 1.105499 | 7.107194 | 8.945963 | 4.56E-13 | 1.55E-11 | 19.44927 |
| MPV17L | 2.108051 | 8.657588 | 8.945452 | 4.57E-13 | 1.56E-11 | 19.44718 |
| MTHFD1 | -2.72252 | 9.643186 | -8.94314 | 4.61E-13 | 1.57E-11 | 19.43777 |
| AOX2P | 0.956704 | 6.964971 | 8.940719 | 4.66E-13 | 1.58E-11 | 19.42792 |
| NMNAT2 | 0.875551 | 6.959058 | 8.936376 | 4.74E-13 | 1.61E-11 | 19.41023 |
| NR2E3 | 0.851537 | 6.923341 | 8.931661 | 4.84E-13 | 1.64E-11 | 19.39103 |
| GAMT | -1.64568 | 9.675009 | -8.92473 | 4.98E-13 | 1.68E-11 | 19.36282 |
| DDX39 | -1.53604 | 7.851849 | -8.92136 | 5.05E-13 | 1.70E-11 | 19.34908 |
| ENY2 | -1.49207 | 7.751923 | -8.92009 | 5.07E-13 | 1.71E-11 | 19.3439 |
| HSPB1 | -1.98036 | 9.586609 | -8.91789 | 5.12E-13 | 1.72E-11 | 19.33497 |
| SNORA75 | -2.31777 | 9.331437 | -8.9162 | 5.16E-13 | 1.73E-11 | 19.32809 |
| ZSCAN10 | 2.093412 | 7.84075 | 8.909377 | 5.30E-13 | 1.78E-11 | 19.30029 |
| KCNK12 | 2.624533 | 8.218845 | 8.908425 | 5.32E-13 | 1.78E-11 | 19.29641 |
| ASCC2 | -1.65148 | 9.350139 | -8.90759 | 5.34E-13 | 1.78E-11 | 19.29303 |
| C11orf63 | 0.855048 | 7.176456 | 8.901315 | 5.48E-13 | 1.83E-11 | 19.26745 |
| C5 | -1.55481 | 9.427661 | -8.89714 | 5.58E-13 | 1.86E-11 | 19.25045 |
| DNAH9 | 1.024218 | 7.013536 | 8.892319 | 5.69E-13 | 1.89E-11 | 19.2308 |
| NUFIP1 | 1.016291 | 7.159562 | 8.887282 | 5.81E-13 | 1.93E-11 | 19.21029 |
| LOC73081 | 1.303476 | 7.065771 | 8.887138 | 5.82E-13 | 1.93E-11 | 19.2097 |
| SH2D7 | 1.766054 | 7.243119 | 8.886422 | 5.83E-13 | 1.93E-11 | 19.20678 |
| OR1L4 | 1.978799 | 7.353405 | 8.883878 | 5.89E-13 | 1.94E-11 | 19.19642 |
| MLEC | -1.10054 | 8.120316 | -8.88328 | 5.91E-13 | 1.94E-11 | 19.194 |
| HGD | -1.94459 | 9.422256 | -8.88192 | 5.94E-13 | 1.95E-11 | 19.18844 |
| CIB1 | -2.54057 | 11.0963 | -8.87646 | 6.08E-13 | 1.99E-11 | 19.16621 |
| IFITM3 | -2.00107 | 9.489567 | -8.87421 | 6.14E-13 | 2.01E-11 | 19.15702 |
| CT45A1 | 1.974184 | 7.295087 | 8.873655 | 6.15E-13 | 2.01E-11 | 19.15476 |
| SERF2 | -1.30589 | 7.922863 | -8.86683 | 6.33E-13 | 2.06E-11 | 19.12697 |
| H6PD | 1.240795 | 9.618679 | 8.863347 | 6.42E-13 | 2.09E-11 | 19.11276 |
| NOP10 | -1.24099 | 8.068233 | -8.86181 | 6.46E-13 | 2.10E-11 | 19.1065 |
| TOM1 | -2.39489 | 9.567299 | -8.86063 | 6.49E-13 | 2.11E-11 | 19.10167 |
| NLRP12 | 0.857423 | 6.983417 | 8.860423 | 6.50E-13 | 2.11E-11 | 19.10085 |
| OR4C15 | 1.045859 | 7.01198 | 8.859534 | 6.52E-13 | 2.11E-11 | 19.09722 |
| PDCD7 | 1.133421 | 9.222638 | 8.856543 | 6.60E-13 | 2.13E-11 | 19.08504 |
| NT5C1B | 1.37777 | 7.012 | 8.85322 | 6.69E-13 | 2.16E-11 | 19.07149 |
| RBM39 | -1.82715 | 8.528241 | -8.85171 | 6.73E-13 | 2.17E-11 | 19.06533 |
| TMEM211 | 0.888292 | 6.814961 | 8.848974 | 6.81E-13 | 2.19E-11 | 19.05419 |
| RPS11 | -1.56876 | 8.204237 | -8.84861 | 6.82E-13 | 2.19E-11 | 19.05269 |
| SF3B1 | -1.01207 | 8.06834 | -8.84746 | 6.85E-13 | 2.20E-11 | 19.04801 |
| C14orf173 | -2.16988 | 9.567626 | -8.84667 | 6.88E-13 | 2.20E-11 | 19.04478 |
| NAPRT1 | -2.1854 | 9.482816 | -8.84516 | 6.92E-13 | 2.21E-11 | 19.03865 |
| CRYGB | 1.727225 | 7.350532 | 8.844535 | 6.94E-13 | 2.21E-11 | 19.0361 |
| ZNF835 | 2.101325 | 7.588449 | 8.8445 | 6.94E-13 | 2.21E-11 | 19.03595 |
| C19orf53 | -2.51657 | 10.00699 | -8.84092 | 7.04E-13 | 2.24E-11 | 19.02136 |

| | | | | | | |
|---|---|---|---|---|---|---|
| GDF3 | 2.490943 | 7.714882 | 8.839896 | 7.07E-13 | 2.24E-11 | 19.01719 |
| USP10 | 0.791108 | 7.594069 | 8.835935 | 7.19E-13 | 2.28E-11 | 19.00104 |
| LOC40080 | 1.079467 | 7.205329 | 8.834407 | 7.24E-13 | 2.29E-11 | 18.99481 |
| PKP1 | 1.236916 | 7.01015 | 8.831595 | 7.32E-13 | 2.31E-11 | 18.98335 |
| SCN2A | 0.869668 | 6.881107 | 8.829094 | 7.40E-13 | 2.33E-11 | 18.97315 |
| SPPL2A | -1.38358 | 8.135167 | -8.8285 | 7.41E-13 | 2.33E-11 | 18.97074 |
| STAG3L1 | 1.625469 | 8.995086 | 8.828489 | 7.41E-13 | 2.33E-11 | 18.97069 |
| FBXO11 | -0.91358 | 7.683728 | -8.82803 | 7.43E-13 | 2.33E-11 | 18.96883 |
| SCG3 | 1.241759 | 7.226821 | 8.827904 | 7.43E-13 | 2.33E-11 | 18.96831 |
| C5orf40 | 1.766041 | 7.724554 | 8.827294 | 7.45E-13 | 2.33E-11 | 18.96582 |
| SERPINA3 | -2.69908 | 9.190463 | -8.82609 | 7.49E-13 | 2.34E-11 | 18.9609 |
| CST2 | 1.887065 | 7.643134 | 8.822462 | 7.60E-13 | 2.37E-11 | 18.94612 |
| PCYT1B | 1.068561 | 7.013661 | 8.820354 | 7.67E-13 | 2.39E-11 | 18.93752 |
| AP3M1 | 1.380014 | 8.298194 | 8.818714 | 7.72E-13 | 2.40E-11 | 18.93084 |
| FAM24A | 1.571499 | 7.126571 | 8.818517 | 7.73E-13 | 2.40E-11 | 18.93004 |
| ZNF557 | 1.059286 | 8.263556 | 8.817456 | 7.76E-13 | 2.40E-11 | 18.92571 |
| LGR8 | 0.872632 | 6.815886 | 8.816242 | 7.80E-13 | 2.41E-11 | 18.92076 |
| FAM181B | 1.016882 | 7.025038 | 8.814303 | 7.86E-13 | 2.43E-11 | 18.91285 |
| PODXL | 1.653021 | 9.537145 | 8.812198 | 7.93E-13 | 2.44E-11 | 18.90427 |
| KIAA0087 | 1.20042 | 6.944711 | 8.812189 | 7.93E-13 | 2.44E-11 | 18.90424 |
| ARF1 | -1.51244 | 9.176929 | -8.81201 | 7.94E-13 | 2.44E-11 | 18.9035 |
| DECR1 | -1.53943 | 8.058075 | -8.81148 | 7.96E-13 | 2.44E-11 | 18.90136 |
| SNORD12 | -2.56923 | 11.16737 | -8.80919 | 8.03E-13 | 2.46E-11 | 18.89201 |
| SFTA3 | 1.447971 | 7.135644 | 8.808781 | 8.05E-13 | 2.46E-11 | 18.89034 |
| SCARNA4 | -1.69112 | 9.705066 | -8.8057 | 8.15E-13 | 2.49E-11 | 18.87779 |
| IGDCC3 | 2.328609 | 7.722343 | 8.798705 | 8.39E-13 | 2.56E-11 | 18.84926 |
| ZMAT2 | -1.296 | 7.969916 | -8.79742 | 8.43E-13 | 2.57E-11 | 18.844 |
| MBD3L5 | 1.779865 | 7.302556 | 8.796881 | 8.45E-13 | 2.57E-11 | 18.84182 |
| GC | -2.15985 | 8.832324 | -8.79379 | 8.56E-13 | 2.60E-11 | 18.82922 |
| PACRG | 1.448764 | 7.538524 | 8.79297 | 8.59E-13 | 2.60E-11 | 18.82587 |
| BOP1 | -0.85377 | 7.207258 | -8.79122 | 8.65E-13 | 2.62E-11 | 18.81875 |
| RAB40C | -1.72252 | 8.193582 | -8.78867 | 8.75E-13 | 2.64E-11 | 18.80834 |
| PLA2G4B | -1.71093 | 9.403877 | -8.78272 | 8.96E-13 | 2.70E-11 | 18.78405 |
| SCAP | -1.28865 | 8.091359 | -8.77904 | 9.10E-13 | 2.74E-11 | 18.76904 |
| ALDH4A1 | -1.72075 | 8.80584 | -8.77845 | 9.12E-13 | 2.74E-11 | 18.76667 |
| MIR1324 | 1.160542 | 7.023751 | 8.778361 | 9.13E-13 | 2.74E-11 | 18.76629 |
| SEPX1 | -1.44048 | 8.31806 | -8.77713 | 9.17E-13 | 2.75E-11 | 18.76128 |
| HOXA3 | 1.270829 | 7.426828 | 8.77577 | 9.23E-13 | 2.76E-11 | 18.75572 |
| ENSA | -1.12505 | 8.290634 | -8.76625 | 9.60E-13 | 2.87E-11 | 18.71688 |
| ACAT1 | -1.10806 | 7.528422 | -8.76536 | 9.63E-13 | 2.87E-11 | 18.71326 |
| SPATA8 | 1.477539 | 7.122886 | 8.755635 | 1.00E-12 | 2.98E-11 | 18.67359 |
| MUC21 | 1.230496 | 6.999274 | 8.754559 | 1.01E-12 | 2.99E-11 | 18.6692 |
| CSNK1E | -1.37477 | 8.600246 | -8.75162 | 1.02E-12 | 3.02E-11 | 18.65722 |
| MGC7199 | -1.12061 | 8.411879 | -8.75063 | 1.02E-12 | 3.03E-11 | 18.65317 |
| C20orf20 | -2.23548 | 9.131226 | -8.74801 | 1.04E-12 | 3.06E-11 | 18.64249 |
| C3orf51 | 2.196607 | 7.346862 | 8.742171 | 1.06E-12 | 3.13E-11 | 18.61866 |
| TSG1 | 1.747941 | 7.349759 | 8.741269 | 1.06E-12 | 3.14E-11 | 18.61498 |
| LOC44195 | 2.172147 | 7.632485 | 8.740253 | 1.07E-12 | 3.15E-11 | 18.61083 |
| EIF4G2 | -1.58874 | 8.381431 | -8.73686 | 1.08E-12 | 3.19E-11 | 18.59697 |
| HPCA | 1.917873 | 7.481135 | 8.734674 | 1.09E-12 | 3.21E-11 | 18.58807 |
| SNORA9 | -1.95739 | 8.80505 | -8.72261 | 1.15E-12 | 3.37E-11 | 18.53882 |
| SNRPD2 | -1.99528 | 8.605878 | -8.72121 | 1.16E-12 | 3.39E-11 | 18.53312 |
| TMEM115 | -2.29698 | 9.6515 | -8.72059 | 1.16E-12 | 3.39E-11 | 18.53059 |
| WFDC6 | 0.86204 | 6.832039 | 8.717351 | 1.18E-12 | 3.43E-11 | 18.51738 |
| ITGA1 | -1.25542 | 8.020153 | -8.71683 | 1.18E-12 | 3.43E-11 | 18.51524 |
| HLA-B | -2.80915 | 9.828529 | -8.71302 | 1.20E-12 | 3.48E-11 | 18.49972 |
| SNORD58 | -1.47226 | 7.7275 | -8.70702 | 1.23E-12 | 3.57E-11 | 18.4752 |
| ZC3H3 | -2.31122 | 8.883755 | -8.70408 | 1.24E-12 | 3.60E-11 | 18.46322 |

| | | | | | | |
|---|---|---|---|---|---|---|
| TPST2 | -1.19551 | 7.787149 | -8.70401 | 1.24E-12 | 3.60E-11 | 18.46295 |
| SERPINF1 | -2.57963 | 9.232903 | -8.70369 | 1.24E-12 | 3.60E-11 | 18.46163 |
| SLC36A2 | 2.930346 | 7.968859 | 8.70351 | 1.24E-12 | 3.60E-11 | 18.46089 |
| CLCN7 | -1.88381 | 10.42478 | -8.70315 | 1.25E-12 | 3.60E-11 | 18.4594 |
| PTMS | -1.05078 | 11.48655 | -8.70236 | 1.25E-12 | 3.61E-11 | 18.45618 |
| KCNJ13 | 2.010661 | 7.763706 | 8.699341 | 1.27E-12 | 3.65E-11 | 18.44388 |
| CREB3L3 | -2.19601 | 9.013863 | -8.69757 | 1.28E-12 | 3.67E-11 | 18.43666 |
| DAND5 | 2.691268 | 8.430992 | 8.694192 | 1.29E-12 | 3.71E-11 | 18.42286 |
| CAMLG | -1.54611 | 9.999585 | -8.69039 | 1.31E-12 | 3.77E-11 | 18.40735 |
| MLF2 | -1.17647 | 9.350483 | -8.69017 | 1.32E-12 | 3.77E-11 | 18.40645 |
| MGC1192 | 1.357261 | 7.231147 | 8.689309 | 1.32E-12 | 3.77E-11 | 18.40293 |
| SGCG | 0.963968 | 6.868507 | 8.688783 | 1.32E-12 | 3.78E-11 | 18.40078 |
| C9orf41 | 1.653117 | 8.869112 | 8.688487 | 1.32E-12 | 3.78E-11 | 18.39957 |
| ISM2 | 1.03751 | 7.000839 | 8.686139 | 1.34E-12 | 3.81E-11 | 18.38998 |
| SMARCA5 | -1.06299 | 7.38506 | -8.68315 | 1.35E-12 | 3.85E-11 | 18.37777 |
| SNORA38 | -1.85849 | 10.37719 | -8.68091 | 1.37E-12 | 3.88E-11 | 18.36864 |
| MGAT5B | 1.490762 | 7.461278 | 8.67741 | 1.39E-12 | 3.93E-11 | 18.35435 |
| RNASE8 | 1.130504 | 6.951499 | 8.676406 | 1.39E-12 | 3.94E-11 | 18.35025 |
| CCDC153 | 0.939348 | 7.043825 | 8.676074 | 1.39E-12 | 3.94E-11 | 18.3489 |
| GCNT1 | 1.622183 | 7.723176 | 8.67573 | 1.40E-12 | 3.94E-11 | 18.34749 |
| SNORA32 | -1.84273 | 8.279287 | -8.67222 | 1.42E-12 | 4.00E-11 | 18.33314 |
| NDUFB10 | -1.17268 | 7.883806 | -8.66906 | 1.44E-12 | 4.04E-11 | 18.32024 |
| FAM163A | 1.434455 | 7.135492 | 8.667791 | 1.44E-12 | 4.06E-11 | 18.31508 |
| SHISA5 | -1.00832 | 8.236794 | -8.66474 | 1.46E-12 | 4.11E-11 | 18.30263 |
| OR52I2 | 1.368186 | 7.036126 | 8.66354 | 1.47E-12 | 4.12E-11 | 18.29773 |
| CDV3 | -1.23294 | 8.771857 | -8.66203 | 1.48E-12 | 4.14E-11 | 18.29157 |
| C1orf50 | -0.9274 | 7.333149 | -8.66055 | 1.49E-12 | 4.16E-11 | 18.28553 |
| RPS19 | -2.24439 | 10.88218 | -8.65715 | 1.51E-12 | 4.21E-11 | 18.27162 |
| SNORA65 | -3.07807 | 10.82254 | -8.65681 | 1.51E-12 | 4.21E-11 | 18.27026 |
| C1orf118 | 0.83908 | 6.939569 | 8.653684 | 1.53E-12 | 4.26E-11 | 18.25748 |
| KPRP | 2.615796 | 7.876894 | 8.651341 | 1.55E-12 | 4.29E-11 | 18.24792 |
| PSCD1 | -1.05906 | 7.586693 | -8.65113 | 1.55E-12 | 4.29E-11 | 18.24706 |
| KIAA0090 | -0.83988 | 7.173512 | -8.65059 | 1.55E-12 | 4.30E-11 | 18.24486 |
| TAGLN3 | 0.958046 | 6.931695 | 8.648208 | 1.57E-12 | 4.33E-11 | 18.23512 |
| LOC15266 | 2.542862 | 7.89951 | 8.647452 | 1.57E-12 | 4.34E-11 | 18.23203 |
| HPD | -2.12125 | 8.247841 | -8.64719 | 1.57E-12 | 4.34E-11 | 18.23096 |
| KCNH5 | 2.334442 | 7.6793 | 8.646587 | 1.58E-12 | 4.34E-11 | 18.2285 |
| C16orf58 | -2.0196 | 10.25501 | -8.64425 | 1.59E-12 | 4.38E-11 | 18.21897 |
| SHMT2 | -1.76826 | 8.960907 | -8.6438 | 1.59E-12 | 4.38E-11 | 18.21712 |
| PTBP1 | -2.10668 | 10.03735 | -8.64202 | 1.61E-12 | 4.41E-11 | 18.20983 |
| UBA52 | -2.27195 | 10.96514 | -8.64052 | 1.62E-12 | 4.43E-11 | 18.20373 |
| ARL16 | 1.158955 | 11.34685 | 8.638256 | 1.63E-12 | 4.46E-11 | 18.19448 |
| E2F6 | 1.700882 | 8.211043 | 8.637749 | 1.64E-12 | 4.47E-11 | 18.19241 |
| CHPT1 | -1.76156 | 8.463434 | -8.6342 | 1.66E-12 | 4.53E-11 | 18.17793 |
| LOC16263 | 1.963652 | 7.988342 | 8.63051 | 1.69E-12 | 4.59E-11 | 18.16285 |
| MET | -1.0211 | 7.81543 | -8.62609 | 1.72E-12 | 4.67E-11 | 18.14479 |
| PPP2CA | -1.70823 | 8.195848 | -8.62161 | 1.75E-12 | 4.75E-11 | 18.1265 |
| KCNH3 | 2.087326 | 8.118064 | 8.621005 | 1.75E-12 | 4.76E-11 | 18.12403 |
| SCARNA5 | -1.84725 | 8.745882 | -8.61838 | 1.77E-12 | 4.80E-11 | 18.11331 |
| NUDT3 | -1.04712 | 7.837393 | -8.61758 | 1.78E-12 | 4.81E-11 | 18.11006 |
| FTHL7 | -1.08445 | 11.27977 | -8.61645 | 1.79E-12 | 4.82E-11 | 18.10543 |
| ETFB | -1.69459 | 9.44113 | -8.61644 | 1.79E-12 | 4.82E-11 | 18.10538 |
| UNC5D | 1.054946 | 6.887359 | 8.607929 | 1.85E-12 | 4.98E-11 | 18.07062 |
| KDELR2 | -1.45387 | 8.494818 | -8.60706 | 1.86E-12 | 4.99E-11 | 18.06708 |
| SNORD25 | -1.76576 | 8.571449 | -8.60623 | 1.86E-12 | 5.00E-11 | 18.0637 |
| SCARNA1 | -2.27452 | 10.19052 | -8.60605 | 1.87E-12 | 5.00E-11 | 18.06294 |
| PRR20B | 3.08633 | 8.355208 | 8.603929 | 1.88E-12 | 5.04E-11 | 18.05428 |
| KIAA1751 | 1.463928 | 11.56764 | 8.602972 | 1.89E-12 | 5.05E-11 | 18.05037 |

| | | | | | | |
|---|---|---|---|---|---|---|
| CEBPD | -1.79101 | 8.422565 | -8.60145 | 1.90E-12 | 5.08E-11 | 18.04414 |
| CYP3A5 | -2.65816 | 9.41234 | -8.59932 | 1.92E-12 | 5.12E-11 | 18.03546 |
| GIT2 | -1.28534 | 7.701913 | -8.59904 | 1.92E-12 | 5.12E-11 | 18.03431 |
| HSD17B4 | -1.37296 | 8.326254 | -8.59762 | 1.93E-12 | 5.13E-11 | 18.02853 |
| LOC37439 | -3.17756 | 10.4942 | -8.59754 | 1.93E-12 | 5.13E-11 | 18.02818 |
| FLJ45337 | 1.900723 | 8.094052 | 8.596086 | 1.94E-12 | 5.16E-11 | 18.02224 |
| OR7E37P | 1.554248 | 7.654205 | 8.595771 | 1.95E-12 | 5.16E-11 | 18.02095 |
| HDHD2 | -1.23614 | 7.977903 | -8.59489 | 1.95E-12 | 5.17E-11 | 18.01735 |
| FLJ32679 | 0.817931 | 6.97324 | 8.59208 | 1.98E-12 | 5.22E-11 | 18.00587 |
| SERPING1 | -1.33198 | 8.238236 | -8.59195 | 1.98E-12 | 5.22E-11 | 18.00535 |
| BEND4 | 1.071613 | 7.141362 | 8.588211 | 2.01E-12 | 5.30E-11 | 17.99007 |
| BCKDHA | -1.81298 | 9.196816 | -8.58644 | 2.02E-12 | 5.32E-11 | 17.98284 |
| CLPS | 0.99814 | 6.85925 | 8.58164 | 2.06E-12 | 5.42E-11 | 17.96322 |
| C12orf36 | 1.163385 | 7.015538 | 8.580206 | 2.08E-12 | 5.45E-11 | 17.95737 |
| CROP | -1.00562 | 7.566673 | -8.57838 | 2.09E-12 | 5.48E-11 | 17.94992 |
| TCEB2 | -1.09578 | 9.209735 | -8.57516 | 2.12E-12 | 5.54E-11 | 17.93674 |
| TAF11 | 1.635715 | 8.77563 | 8.575139 | 2.12E-12 | 5.54E-11 | 17.93666 |
| LOC22296 | 1.984993 | 7.506514 | 8.574986 | 2.12E-12 | 5.54E-11 | 17.93604 |
| OR2T4 | 1.45666 | 7.10915 | 8.574 | 2.13E-12 | 5.55E-11 | 17.93201 |
| KRT72 | 1.544464 | 7.145766 | 8.569074 | 2.18E-12 | 5.66E-11 | 17.91188 |
| MIR663B | 2.195583 | 7.525658 | 8.567025 | 2.19E-12 | 5.70E-11 | 17.90351 |
| NTRK1 | 1.384958 | 7.207458 | 8.566939 | 2.19E-12 | 5.70E-11 | 17.90316 |
| MCM8 | 1.633325 | 8.982473 | 8.565621 | 2.21E-12 | 5.72E-11 | 17.89777 |
| FAU | -1.69659 | 8.656276 | -8.56267 | 2.23E-12 | 5.79E-11 | 17.88571 |
| ITGB1 | -1.211 | 7.893947 | -8.56199 | 2.24E-12 | 5.80E-11 | 17.88295 |
| PRSS21 | 0.826354 | 6.94105 | 8.561384 | 2.25E-12 | 5.80E-11 | 17.88046 |
| HTA | 1.846379 | 7.270338 | 8.560296 | 2.26E-12 | 5.82E-11 | 17.87602 |
| HNRPA2B | -1.99936 | 9.059519 | -8.55878 | 2.27E-12 | 5.85E-11 | 17.86982 |
| LOC72894 | 0.789397 | 6.812452 | 8.552669 | 2.33E-12 | 5.98E-11 | 17.84485 |
| TKT | -1.80687 | 8.305925 | -8.55224 | 2.33E-12 | 5.98E-11 | 17.84311 |
| SLC44A4 | 1.729747 | 9.680655 | 8.548735 | 2.37E-12 | 6.06E-11 | 17.82877 |
| RAD52 | 1.856608 | 8.782611 | 8.548538 | 2.37E-12 | 6.06E-11 | 17.82797 |
| PRDM14 | 1.363154 | 7.140388 | 8.547741 | 2.38E-12 | 6.07E-11 | 17.82471 |
| NRM | 1.681749 | 9.158963 | 8.547037 | 2.38E-12 | 6.08E-11 | 17.82183 |
| NCRNA00 | 1.199338 | 7.031808 | 8.546208 | 2.39E-12 | 6.10E-11 | 17.81845 |
| AXUD1 | -1.75424 | 8.144724 | -8.54105 | 2.44E-12 | 6.22E-11 | 17.79736 |
| C3 | -2.09689 | 10.14998 | -8.5404 | 2.45E-12 | 6.23E-11 | 17.79472 |
| NDST3 | 1.240591 | 7.013763 | 8.539375 | 2.46E-12 | 6.25E-11 | 17.79052 |
| RAXL1 | 2.094385 | 8.296301 | 8.537915 | 2.48E-12 | 6.28E-11 | 17.78455 |
| KILLIN | 1.483869 | 7.80276 | 8.537458 | 2.48E-12 | 6.28E-11 | 17.78269 |
| OR5P3 | 1.58353 | 7.269632 | 8.537117 | 2.48E-12 | 6.28E-11 | 17.78129 |
| TSPO | -1.29674 | 8.277521 | -8.53055 | 2.55E-12 | 6.45E-11 | 17.75447 |
| SSU72 | -1.16103 | 8.991661 | -8.52655 | 2.60E-12 | 6.55E-11 | 17.73812 |
| CACNG5 | 1.534688 | 7.243239 | 8.525772 | 2.60E-12 | 6.56E-11 | 17.73493 |
| FOXN1 | 1.324352 | 7.177511 | 8.522137 | 2.64E-12 | 6.65E-11 | 17.72007 |
| KLF2 | -1.41051 | 8.401486 | -8.51897 | 2.68E-12 | 6.72E-11 | 17.70714 |
| ZNF365 | 0.778216 | 6.814723 | 8.518707 | 2.68E-12 | 6.72E-11 | 17.70605 |
| MIR1289- | 1.747892 | 7.367262 | 8.516364 | 2.71E-12 | 6.78E-11 | 17.69647 |
| TTR | -2.91688 | 9.858921 | -8.51445 | 2.73E-12 | 6.82E-11 | 17.68864 |
| OR52L1 | 1.133123 | 6.992966 | 8.513933 | 2.74E-12 | 6.83E-11 | 17.68654 |
| ARPC1B | -1.47491 | 7.773304 | -8.50929 | 2.79E-12 | 6.95E-11 | 17.66754 |
| NDUFA10 | -1.19549 | 7.680048 | -8.50853 | 2.80E-12 | 6.97E-11 | 17.66445 |
| ICAM4 | 1.266663 | 7.312546 | 8.500224 | 2.90E-12 | 7.20E-11 | 17.6305 |
| MIR1272 | 1.11277 | 7.073262 | 8.493034 | 2.98E-12 | 7.40E-11 | 17.60111 |
| UBA7 | -2.10522 | 8.799132 | -8.49083 | 3.01E-12 | 7.46E-11 | 17.5921 |
| LOC40125 | 2.133813 | 10.01149 | 8.490702 | 3.01E-12 | 7.46E-11 | 17.59158 |
| BTG1 | -1.16142 | 7.98501 | -8.48741 | 3.05E-12 | 7.55E-11 | 17.57811 |
| SNORA48 | -1.27585 | 7.715934 | -8.486 | 3.07E-12 | 7.59E-11 | 17.57234 |

| | | | | | | |
|---|---|---|---|---|---|---|
| F7 | -1.96212 | 9.09952 | -8.4851 | 3.08E-12 | 7.60E-11 | 17.56869 |
| MGC1099 | 1.91108 | 8.467308 | 8.484583 | 3.09E-12 | 7.60E-11 | 17.56656 |
| MRGPRX3 | 0.768293 | 6.905681 | 8.484562 | 3.09E-12 | 7.60E-11 | 17.56647 |
| PSMB7 | -0.8939 | 7.56329 | -8.47962 | 3.15E-12 | 7.75E-11 | 17.54626 |
| PPIAL4G | 2.561137 | 8.028551 | 8.477585 | 3.18E-12 | 7.81E-11 | 17.53795 |
| KLHDC3 | -2.28969 | 8.837676 | -8.47611 | 3.20E-12 | 7.84E-11 | 17.53194 |
| COX4I1 | -2.89311 | 9.803781 | -8.47601 | 3.20E-12 | 7.84E-11 | 17.53151 |
| EEF1A1 | -1.46265 | 9.051374 | -8.47547 | 3.21E-12 | 7.85E-11 | 17.52932 |
| OR10G8 | 2.634444 | 7.741756 | 8.474756 | 3.22E-12 | 7.87E-11 | 17.52638 |
| SLC8A2 | 2.58137 | 8.014387 | 8.47024 | 3.28E-12 | 8.00E-11 | 17.50792 |
| CCT8L1 | 0.910249 | 6.913975 | 8.468849 | 3.30E-12 | 8.04E-11 | 17.50223 |
| ATP2A1 | 1.526185 | 7.599811 | 8.467668 | 3.32E-12 | 8.07E-11 | 17.4974 |
| ZNF787 | -1.14279 | 7.642288 | -8.46706 | 3.32E-12 | 8.08E-11 | 17.4949 |
| OR2B2 | 1.18886 | 7.079168 | 8.466553 | 3.33E-12 | 8.08E-11 | 17.49284 |
| GRIN1 | 0.994743 | 7.063855 | 8.461802 | 3.40E-12 | 8.23E-11 | 17.47342 |
| EWSR1 | -1.3664 | 8.193359 | -8.46132 | 3.40E-12 | 8.23E-11 | 17.47147 |
| DTWD2 | 0.796316 | 12.36192 | 8.461256 | 3.40E-12 | 8.23E-11 | 17.47119 |
| TMEM123 | -1.64475 | 8.579265 | -8.46035 | 3.42E-12 | 8.25E-11 | 17.46748 |
| PRDM7 | 2.245284 | 8.709288 | 8.459455 | 3.43E-12 | 8.27E-11 | 17.46382 |
| MT1P2 | 0.898215 | 6.834562 | 8.457818 | 3.45E-12 | 8.32E-11 | 17.45713 |
| GPR141 | 0.773584 | 7.04147 | 8.453114 | 3.52E-12 | 8.48E-11 | 17.4379 |
| SPATA20 | -1.89418 | 8.706187 | -8.45249 | 3.53E-12 | 8.49E-11 | 17.43535 |
| DENR | 1.758858 | 9.544229 | 8.441565 | 3.70E-12 | 8.85E-11 | 17.39067 |
| FGF8 | 1.674191 | 7.125402 | 8.437466 | 3.76E-12 | 8.99E-11 | 17.37391 |
| ALDH5A1 | -1.3641 | 7.969162 | -8.43301 | 3.83E-12 | 9.14E-11 | 17.35567 |
| ZNF542 | 1.105382 | 7.563259 | 8.43072 | 3.87E-12 | 9.22E-11 | 17.34632 |
| KRT16 | 1.918686 | 7.622359 | 8.428609 | 3.90E-12 | 9.27E-11 | 17.33769 |
| C6orf125 | -1.60664 | 7.952637 | -8.42825 | 3.91E-12 | 9.27E-11 | 17.33623 |
| LOC64915 | 2.478673 | 7.802493 | 8.428204 | 3.91E-12 | 9.27E-11 | 17.33603 |
| CDH29 | 1.037435 | 6.924257 | 8.427704 | 3.91E-12 | 9.28E-11 | 17.33399 |
| EDG7 | 1.65473 | 7.176783 | 8.425798 | 3.95E-12 | 9.34E-11 | 17.32619 |
| CD14 | -1.36021 | 9.897266 | -8.42316 | 3.99E-12 | 9.43E-11 | 17.31539 |
| ZNF682 | 0.976613 | 9.468244 | 8.422201 | 4.00E-12 | 9.46E-11 | 17.31148 |
| FLJ46321 | 0.851275 | 6.782548 | 8.421201 | 4.02E-12 | 9.49E-11 | 17.30739 |
| DAOA | 1.11689 | 6.926873 | 8.420908 | 4.03E-12 | 9.49E-11 | 17.30619 |
| MAT1A | -1.45439 | 7.793325 | -8.41971 | 4.05E-12 | 9.53E-11 | 17.3013 |
| RFPL3S | 1.046782 | 7.095827 | 8.415085 | 4.13E-12 | 9.69E-11 | 17.28238 |
| ZFP36L2 | -1.16521 | 8.127406 | -8.41507 | 4.13E-12 | 9.69E-11 | 17.2823 |
| HSD17B11 | -0.89794 | 7.601716 | -8.41207 | 4.18E-12 | 9.79E-11 | 17.27004 |
| MIR1299 | 1.424181 | 7.9171 | 8.409125 | 4.23E-12 | 9.90E-11 | 17.258 |
| SNORA20 | -2.344 | 8.903713 | -8.40488 | 4.30E-12 | 1.01E-10 | 17.24064 |
| KRT5 | 1.751813 | 7.513889 | 8.404671 | 4.31E-12 | 1.01E-10 | 17.23979 |
| KLK3 | 1.351903 | 7.079199 | 8.404298 | 4.31E-12 | 1.01E-10 | 17.23826 |
| NUDC | -2.03995 | 10.39922 | -8.40383 | 4.32E-12 | 1.01E-10 | 17.23636 |
| TFIP11 | 1.566202 | 9.161462 | 8.403743 | 4.32E-12 | 1.01E-10 | 17.23599 |
| ATP6V0C | -1.26928 | 7.912905 | -8.40023 | 4.39E-12 | 1.02E-10 | 17.22163 |
| DUXA | 1.764357 | 7.243125 | 8.399425 | 4.40E-12 | 1.02E-10 | 17.21833 |
| PSMG2 | -2.30614 | 8.968948 | -8.39904 | 4.41E-12 | 1.02E-10 | 17.21676 |
| LY6G5C | 1.750331 | 8.216243 | 8.397976 | 4.43E-12 | 1.03E-10 | 17.2124 |
| TIMP4 | 1.958899 | 7.667258 | 8.395338 | 4.48E-12 | 1.03E-10 | 17.20161 |
| ARMET | -2.0114 | 9.527993 | -8.39523 | 4.48E-12 | 1.03E-10 | 17.20116 |
| DPPA3 | 1.20974 | 7.016878 | 8.394465 | 4.49E-12 | 1.04E-10 | 17.19804 |
| TAGLN2 | -1.36139 | 8.895527 | -8.39293 | 4.52E-12 | 1.04E-10 | 17.19176 |
| APOBEC3I | 1.568062 | 7.53345 | 8.391818 | 4.54E-12 | 1.04E-10 | 17.18721 |
| HYALP1 | 0.823335 | 6.941754 | 8.388521 | 4.61E-12 | 1.06E-10 | 17.17373 |
| PPP6C | -1.38199 | 8.39977 | -8.38758 | 4.63E-12 | 1.06E-10 | 17.16986 |
| KCNK10 | 0.700196 | 6.817789 | 8.38436 | 4.69E-12 | 1.07E-10 | 17.15671 |
| HNRNPD | -1.4888 | 9.098911 | -8.38346 | 4.70E-12 | 1.08E-10 | 17.15305 |

| | | | | | | |
|---|---|---|---|---|---|---|
| GRAMD1A | -1.153 | 7.611274 | -8.38207 | 4.73E-12 | 1.08E-10 | 17.14734 |
| PPM1K | 1.072204 | 7.367693 | 8.381344 | 4.75E-12 | 1.08E-10 | 17.14437 |
| RPS6KA3 | -0.95401 | 7.820549 | -8.37619 | 4.85E-12 | 1.11E-10 | 17.12331 |
| C2orf27B | 0.822974 | 6.849764 | 8.375741 | 4.86E-12 | 1.11E-10 | 17.12145 |
| HDHD3 | -1.96293 | 10.18474 | -8.37521 | 4.87E-12 | 1.11E-10 | 17.11928 |
| SNX15 | 1.256423 | 8.569966 | 8.370353 | 4.97E-12 | 1.13E-10 | 17.09941 |
| ELK3 | -0.99036 | 7.21331 | -8.37003 | 4.98E-12 | 1.13E-10 | 17.09809 |
| PLIN5 | 1.145134 | 8.37353 | 8.369212 | 4.99E-12 | 1.13E-10 | 17.09475 |
| PRSS38 | 1.361941 | 7.084495 | 8.368851 | 5.00E-12 | 1.13E-10 | 17.09327 |
| FAM75A5 | 1.094006 | 7.049892 | 8.367627 | 5.03E-12 | 1.14E-10 | 17.08826 |
| VIT | 1.492577 | 7.268602 | 8.366253 | 5.05E-12 | 1.14E-10 | 17.08264 |
| FGF9 | 1.363876 | 7.118603 | 8.36555 | 5.07E-12 | 1.14E-10 | 17.07976 |
| ATP5B | -1.5117 | 9.900804 | -8.3652 | 5.08E-12 | 1.14E-10 | 17.07833 |
| GPR31 | 1.220198 | 7.115086 | 8.364859 | 5.08E-12 | 1.14E-10 | 17.07694 |
| RBM9 | -1.65836 | 8.309201 | -8.36308 | 5.12E-12 | 1.15E-10 | 17.06966 |
| UROS | -1.55979 | 8.291562 | -8.36048 | 5.18E-12 | 1.16E-10 | 17.05904 |
| RBL1 | 1.554456 | 8.388597 | 8.357997 | 5.23E-12 | 1.17E-10 | 17.04887 |
| FLJ45256 | 1.996934 | 9.697139 | 8.352875 | 5.34E-12 | 1.20E-10 | 17.02791 |
| TNFRSF13 | 2.232882 | 7.939193 | 8.348446 | 5.44E-12 | 1.22E-10 | 17.00979 |
| PPP1R13B | -1.38079 | 9.23234 | -8.34449 | 5.53E-12 | 1.24E-10 | 16.9936 |
| C10orf27 | 1.895349 | 7.468253 | 8.343446 | 5.56E-12 | 1.24E-10 | 16.98934 |
| SLC4A11 | 1.201502 | 7.107517 | 8.339538 | 5.65E-12 | 1.26E-10 | 16.97335 |
| RNF216L | 1.662407 | 7.800587 | 8.338915 | 5.66E-12 | 1.26E-10 | 16.9708 |
| FGA | -2.10215 | 9.065755 | -8.33499 | 5.76E-12 | 1.28E-10 | 16.95476 |
| CLRN2 | 1.37789 | 7.194236 | 8.334774 | 5.76E-12 | 1.28E-10 | 16.95386 |
| SLC37A4 | -1.55564 | 8.344409 | -8.33462 | 5.76E-12 | 1.28E-10 | 16.95324 |
| ERLIN2 | -0.76666 | 7.22461 | -8.33356 | 5.79E-12 | 1.28E-10 | 16.94889 |
| NCF1B | 1.653904 | 7.536874 | 8.331898 | 5.83E-12 | 1.29E-10 | 16.94209 |
| SEC61A1 | -1.98445 | 9.612011 | -8.33137 | 5.84E-12 | 1.29E-10 | 16.93993 |
| KRTAP3-2 | 2.6558 | 7.783828 | 8.328628 | 5.91E-12 | 1.30E-10 | 16.92871 |
| CD99L2 | -1.35205 | 8.284627 | -8.32861 | 5.91E-12 | 1.30E-10 | 16.92863 |
| PNLIPRP1 | 1.340638 | 7.094781 | 8.328539 | 5.91E-12 | 1.30E-10 | 16.92835 |
| C21orf49 | 0.948648 | 7.11418 | 8.327935 | 5.93E-12 | 1.31E-10 | 16.92588 |
| SNORD49 | -1.89734 | 8.517561 | -8.32581 | 5.98E-12 | 1.32E-10 | 16.91716 |
| OR13A1 | 1.641257 | 7.238483 | 8.325388 | 5.99E-12 | 1.32E-10 | 16.91546 |
| CRYGA | 2.217177 | 7.784513 | 8.324692 | 6.01E-12 | 1.32E-10 | 16.91261 |
| C17orf61 | -1.2725 | 7.815331 | -8.32407 | 6.02E-12 | 1.32E-10 | 16.91008 |
| CD81 | -2.17701 | 10.18351 | -8.32182 | 6.08E-12 | 1.33E-10 | 16.90084 |
| CD8B | 1.103312 | 7.214694 | 8.319574 | 6.14E-12 | 1.34E-10 | 16.89167 |
| DKFZp686 | 2.036237 | 8.446361 | 8.318805 | 6.16E-12 | 1.35E-10 | 16.88852 |
| ERI3 | -2.09802 | 9.112262 | -8.31696 | 6.20E-12 | 1.36E-10 | 16.88097 |
| ACCSL | 1.957786 | 7.440272 | 8.31515 | 6.25E-12 | 1.36E-10 | 16.87357 |
| RAET1G | 1.785353 | 7.331251 | 8.314536 | 6.27E-12 | 1.37E-10 | 16.87106 |
| IKBKAP | 1.001421 | 7.290676 | 8.314397 | 6.27E-12 | 1.37E-10 | 16.87049 |
| WDR18 | -2.43671 | 10.67614 | -8.31292 | 6.31E-12 | 1.37E-10 | 16.86446 |
| RNF160 | -0.70473 | 7.623942 | -8.31209 | 6.33E-12 | 1.38E-10 | 16.86103 |
| VCY | 1.388304 | 7.244203 | 8.31136 | 6.35E-12 | 1.38E-10 | 16.85806 |
| GSG1L | 1.404572 | 7.044639 | 8.310715 | 6.37E-12 | 1.38E-10 | 16.85542 |
| KLK14 | 1.818897 | 7.625801 | 8.310544 | 6.37E-12 | 1.38E-10 | 16.85472 |
| EIF3G | -0.98415 | 7.637802 | -8.31048 | 6.37E-12 | 1.38E-10 | 16.85447 |
| TSPAN4 | -1.24718 | 7.805655 | -8.31044 | 6.37E-12 | 1.38E-10 | 16.85431 |
| HLA-A | -1.9938 | 9.037921 | -8.30724 | 6.46E-12 | 1.40E-10 | 16.84118 |
| RPL38 | -2.5384 | 9.754437 | -8.30517 | 6.52E-12 | 1.41E-10 | 16.83272 |
| RBM10 | -1.33911 | 8.724789 | -8.30458 | 6.53E-12 | 1.41E-10 | 16.83032 |
| ZC3H11A | -1.19047 | 7.601587 | -8.30218 | 6.60E-12 | 1.42E-10 | 16.82051 |
| SDHAP3 | 1.482401 | 8.623644 | 8.300259 | 6.65E-12 | 1.43E-10 | 16.81264 |
| ART5 | 0.938582 | 6.883628 | 8.299771 | 6.66E-12 | 1.43E-10 | 16.81064 |
| IL18 | 1.604736 | 8.707664 | 8.29537 | 6.79E-12 | 1.46E-10 | 16.79263 |

| | | | | | | |
|---|---|---|---|---|---|---|
| ANKRD44 | 0.890997 | 7.597685 | 8.293071 | 6.85E-12 | 1.47E-10 | 16.78323 |
| OR5M9 | 0.807663 | 6.847071 | 8.289924 | 6.94E-12 | 1.49E-10 | 16.77035 |
| GSDMB | 1.123039 | 10.08052 | 8.289443 | 6.96E-12 | 1.49E-10 | 16.76838 |
| C8orf37 | 1.125725 | 9.667967 | 8.289267 | 6.96E-12 | 1.49E-10 | 16.76766 |
| TPRXL | 1.056357 | 7.008832 | 8.288387 | 6.99E-12 | 1.49E-10 | 16.76406 |
| C6orf111 | -1.41532 | 8.323246 | -8.2864 | 7.05E-12 | 1.50E-10 | 16.75592 |
| KCNC2 | 1.304093 | 7.163522 | 8.28632 | 7.05E-12 | 1.50E-10 | 16.7556 |
| KLHL22 | -0.93911 | 8.227801 | -8.28596 | 7.06E-12 | 1.50E-10 | 16.75412 |
| THOC2 | -0.91073 | 7.31026 | -8.28394 | 7.12E-12 | 1.51E-10 | 16.74588 |
| CEP290 | -1.20065 | 7.53531 | -8.28211 | 7.17E-12 | 1.52E-10 | 16.73838 |
| FAM175A | 1.634 | 8.892323 | 8.281357 | 7.19E-12 | 1.53E-10 | 16.73529 |
| TFAMP1 | 1.518499 | 10.43764 | 8.280736 | 7.21E-12 | 1.53E-10 | 16.73275 |
| KCNH2 | 0.958855 | 7.061156 | 8.280046 | 7.23E-12 | 1.53E-10 | 16.72992 |
| SNORD80 | -1.43783 | 7.83324 | -8.27827 | 7.29E-12 | 1.54E-10 | 16.72264 |
| UBXN4 | -1.13718 | 7.798927 | -8.2779 | 7.30E-12 | 1.54E-10 | 16.72113 |
| SNORD96 | -1.69721 | 8.146795 | -8.27573 | 7.37E-12 | 1.55E-10 | 16.71225 |
| KRT77 | 0.904011 | 6.834346 | 8.274888 | 7.39E-12 | 1.56E-10 | 16.70882 |
| ST6GAL1 | -1.50901 | 8.817266 | -8.2739 | 7.42E-12 | 1.56E-10 | 16.70476 |
| CTCFL | 1.255053 | 7.072286 | 8.270172 | 7.54E-12 | 1.58E-10 | 16.68952 |
| ST3GAL2 | -1.38093 | 8.387781 | -8.26814 | 7.60E-12 | 1.59E-10 | 16.68122 |
| KAAG1 | 1.879969 | 7.522326 | 8.263965 | 7.73E-12 | 1.62E-10 | 16.66412 |
| VPS52 | -0.94638 | 8.391247 | -8.26018 | 7.86E-12 | 1.64E-10 | 16.64862 |
| C1orf61 | 1.054691 | 7.160075 | 8.259923 | 7.87E-12 | 1.64E-10 | 16.64758 |
| FSHB | 0.745131 | 6.791317 | 8.259517 | 7.88E-12 | 1.64E-10 | 16.64591 |
| ACY1 | -2.12474 | 8.768761 | -8.25829 | 7.92E-12 | 1.65E-10 | 16.64089 |
| CLDN18 | 1.03246 | 6.951834 | 8.255943 | 8.00E-12 | 1.66E-10 | 16.63129 |
| OR8U8 | 1.219788 | 6.957602 | 8.25426 | 8.05E-12 | 1.67E-10 | 16.6244 |
| ZNF804B | 2.372631 | 7.735101 | 8.252241 | 8.12E-12 | 1.68E-10 | 16.61614 |
| CST3 | -1.22712 | 7.971184 | -8.25219 | 8.12E-12 | 1.68E-10 | 16.61594 |
| NPSR1 | 1.25447 | 7.209445 | 8.251341 | 8.15E-12 | 1.69E-10 | 16.61246 |
| ORC6L | 1.270382 | 10.93468 | 8.250016 | 8.20E-12 | 1.69E-10 | 16.60703 |
| CD151 | -1.79588 | 8.757249 | -8.24948 | 8.22E-12 | 1.70E-10 | 16.60486 |
| U2AF2 | -1.27611 | 7.719201 | -8.24944 | 8.22E-12 | 1.70E-10 | 16.60467 |
| LOC15018 | 1.175317 | 6.947399 | 8.24736 | 8.29E-12 | 1.71E-10 | 16.59617 |
| FAM71F1 | 0.701864 | 6.875632 | 8.24718 | 8.29E-12 | 1.71E-10 | 16.59543 |
| C14orf121 | 2.550386 | 8.560151 | 8.245421 | 8.36E-12 | 1.72E-10 | 16.58823 |
| LOC72834 | 1.163945 | 6.942055 | 8.245196 | 8.36E-12 | 1.72E-10 | 16.58731 |
| PRAMEF1! | 1.052535 | 7.077833 | 8.238749 | 8.59E-12 | 1.76E-10 | 16.56092 |
| XKR3 | 1.235585 | 6.994967 | 8.238241 | 8.61E-12 | 1.77E-10 | 16.55884 |
| SNORD36 | -1.90007 | 8.413151 | -8.23506 | 8.72E-12 | 1.79E-10 | 16.54582 |
| MFSD3 | -1.27984 | 9.826074 | -8.23338 | 8.78E-12 | 1.80E-10 | 16.53896 |
| PON2 | -1.19164 | 7.807418 | -8.22973 | 8.92E-12 | 1.82E-10 | 16.52401 |
| DHX37 | -1.46728 | 8.530466 | -8.22819 | 8.98E-12 | 1.83E-10 | 16.51769 |
| SNORD68 | -1.80611 | 8.287151 | -8.22685 | 9.03E-12 | 1.84E-10 | 16.51222 |
| CLIC1 | -0.95169 | 8.212073 | -8.22541 | 9.08E-12 | 1.85E-10 | 16.50635 |
| TFPT | -1.16381 | 7.4816 | -8.22245 | 9.19E-12 | 1.87E-10 | 16.49422 |
| GPR179 | 1.204245 | 7.146631 | 8.220645 | 9.26E-12 | 1.88E-10 | 16.48683 |
| PKD1L1 | 1.134709 | 7.318859 | 8.216585 | 9.42E-12 | 1.91E-10 | 16.47021 |
| LOC40098 | -0.87627 | 7.321209 | -8.21431 | 9.51E-12 | 1.93E-10 | 16.46092 |
| PRKCB1 | 1.435768 | 7.824531 | 8.213688 | 9.53E-12 | 1.93E-10 | 16.45836 |
| SNORD10 | -2.01022 | 8.655858 | -8.20747 | 9.78E-12 | 1.98E-10 | 16.43291 |
| QRICH1 | -1.03858 | 8.164181 | -8.20584 | 9.85E-12 | 1.99E-10 | 16.42621 |
| LILRB3 | 1.388138 | 11.04667 | 8.203992 | 9.93E-12 | 2.00E-10 | 16.41867 |
| FOXD4L2 | 1.566555 | 7.21606 | 8.203939 | 9.93E-12 | 2.00E-10 | 16.41845 |
| BNIPL | 0.99189 | 7.131673 | 8.203124 | 9.96E-12 | 2.01E-10 | 16.41512 |
| CARS2 | -0.86307 | 7.136064 | -8.20214 | 1.00E-11 | 2.01E-10 | 16.41108 |
| RAD51 | 1.641514 | 9.335369 | 8.201751 | 1.00E-11 | 2.02E-10 | 16.4095 |
| IL9R | 1.532514 | 7.288196 | 8.200714 | 1.01E-11 | 2.02E-10 | 16.40525 |

| | | | | | | |
|---|---|---|---|---|---|---|
| UQCRC1 | -2.12354 | 8.721044 | -8.19984 | 1.01E-11 | 2.03E-10 | 16.40167 |
| TIGD1 | 1.402203 | 7.902875 | 8.198104 | 1.02E-11 | 2.04E-10 | 16.39457 |
| C5orf33 | -1.00706 | 7.452149 | -8.19658 | 1.02E-11 | 2.05E-10 | 16.38832 |
| HRB | -0.93485 | 8.315431 | -8.19537 | 1.03E-11 | 2.06E-10 | 16.38337 |
| HES2 | 0.743767 | 6.815833 | 8.193875 | 1.04E-11 | 2.07E-10 | 16.37726 |
| GFOD2 | -1.17059 | 7.529676 | -8.19319 | 1.04E-11 | 2.07E-10 | 16.37446 |
| FLJ40113 | 2.370505 | 9.43866 | 8.192202 | 1.04E-11 | 2.08E-10 | 16.37042 |
| SNORD67 | -1.90613 | 10.55798 | -8.19218 | 1.04E-11 | 2.08E-10 | 16.37034 |
| SPTBN4 | 1.447166 | 7.407375 | 8.191677 | 1.04E-11 | 2.08E-10 | 16.36827 |
| TMEM127 | -1.86957 | 8.758569 | -8.19166 | 1.05E-11 | 2.08E-10 | 16.36818 |
| MIR744 | 1.072722 | 7.131895 | 8.190683 | 1.05E-11 | 2.09E-10 | 16.3642 |
| MRPS18A | -0.85196 | 7.4197 | -8.19056 | 1.05E-11 | 2.09E-10 | 16.36368 |
| GHRH | 0.802796 | 6.812059 | 8.187214 | 1.06E-11 | 2.11E-10 | 16.35 |
| UBQLN1 | -0.92681 | 7.494062 | -8.18711 | 1.06E-11 | 2.11E-10 | 16.34959 |
| RAB5C | -1.09513 | 8.595837 | -8.18649 | 1.07E-11 | 2.11E-10 | 16.34703 |
| RESP18 | 0.948285 | 6.876988 | 8.184284 | 1.08E-11 | 2.13E-10 | 16.338 |
| RPL14 | -1.33592 | 8.759555 | -8.1819 | 1.09E-11 | 2.15E-10 | 16.32824 |
| CD164 | -1.11094 | 7.785921 | -8.17982 | 1.10E-11 | 2.17E-10 | 16.31975 |
| OR2L8 | 1.73234 | 7.277341 | 8.178577 | 1.10E-11 | 2.18E-10 | 16.31465 |
| C21orf2 | -1.39402 | 8.132275 | -8.17747 | 1.11E-11 | 2.19E-10 | 16.3101 |
| EIF2AK4 | 1.437722 | 10.57771 | 8.177126 | 1.11E-11 | 2.19E-10 | 16.30871 |
| TNRC6A | -1.03506 | 7.903478 | -8.17607 | 1.12E-11 | 2.19E-10 | 16.30439 |
| FLJ45422 | 1.891853 | 7.834312 | 8.17271 | 1.13E-11 | 2.22E-10 | 16.29063 |
| SLC44A2 | -1.60139 | 7.978695 | -8.17142 | 1.14E-11 | 2.23E-10 | 16.28535 |
| LIME1 | -2.25832 | 9.53485 | -8.16955 | 1.15E-11 | 2.25E-10 | 16.2777 |
| SPACA5B | 2.156064 | 7.557893 | 8.167993 | 1.15E-11 | 2.26E-10 | 16.27132 |
| C14orf85 | 1.776134 | 9.490488 | 8.166216 | 1.16E-11 | 2.27E-10 | 16.26405 |
| PJCG6 | 1.341021 | 7.17592 | 8.164545 | 1.17E-11 | 2.29E-10 | 16.25721 |
| CDH8 | 1.278691 | 7.063856 | 8.163891 | 1.17E-11 | 2.29E-10 | 16.25453 |
| LOC64136 | 0.930329 | 7.135046 | 8.163347 | 1.18E-11 | 2.30E-10 | 16.25231 |
| LRRC37A4 | 1.084241 | 7.131999 | 8.158495 | 1.20E-11 | 2.34E-10 | 16.23245 |
| STXBP3 | -0.93176 | 7.257563 | -8.15687 | 1.21E-11 | 2.35E-10 | 16.22579 |
| DUSP8 | 1.250719 | 8.205031 | 8.154483 | 1.22E-11 | 2.37E-10 | 16.21602 |
| SNORD8 | -2.06464 | 9.125634 | -8.15358 | 1.22E-11 | 2.38E-10 | 16.21232 |
| PCBP2 | -0.94616 | 7.970243 | -8.15274 | 1.23E-11 | 2.39E-10 | 16.20888 |
| CTSD | -1.18343 | 7.978295 | -8.14738 | 1.26E-11 | 2.44E-10 | 16.18697 |
| SNORD52 | -1.57863 | 8.105698 | -8.14387 | 1.27E-11 | 2.47E-10 | 16.1726 |
| SPHK2 | -1.28448 | 7.689148 | -8.14373 | 1.28E-11 | 2.47E-10 | 16.172 |
| EIF4EBP2 | -1.50932 | 9.142825 | -8.14372 | 1.28E-11 | 2.47E-10 | 16.17196 |
| PTPRK | -0.86145 | 7.490063 | -8.14246 | 1.28E-11 | 2.48E-10 | 16.16681 |
| SNORD66 | -2.51449 | 11.14765 | -8.14185 | 1.29E-11 | 2.48E-10 | 16.16429 |
| XKR5 | 1.415959 | 7.179877 | 8.139957 | 1.30E-11 | 2.50E-10 | 16.15656 |
| IL17F | 2.416301 | 7.499624 | 8.138439 | 1.30E-11 | 2.51E-10 | 16.15035 |
| CTDSP2 | -0.80322 | 8.027099 | -8.1381 | 1.31E-11 | 2.51E-10 | 16.14897 |
| LY9 | 0.992825 | 7.221219 | 8.136571 | 1.31E-11 | 2.53E-10 | 16.1427 |
| WBP2 | -2.00711 | 9.031884 | -8.13406 | 1.33E-11 | 2.55E-10 | 16.13243 |
| SOX17 | 1.958008 | 8.529077 | 8.133137 | 1.33E-11 | 2.56E-10 | 16.12865 |
| ZNF860 | 0.956304 | 7.703629 | 8.127212 | 1.37E-11 | 2.62E-10 | 16.10439 |
| OR52W1 | 2.515455 | 8.018565 | 8.127158 | 1.37E-11 | 2.62E-10 | 16.10418 |
| ARL17P1 | 1.940079 | 8.057315 | 8.125913 | 1.37E-11 | 2.63E-10 | 16.09908 |
| LMOD3 | 1.049659 | 7.114102 | 8.125514 | 1.38E-11 | 2.63E-10 | 16.09744 |
| GFRA4 | 0.822508 | 6.860908 | 8.125438 | 1.38E-11 | 2.63E-10 | 16.09713 |
| COX5B | -1.57902 | 8.044107 | -8.1249 | 1.38E-11 | 2.63E-10 | 16.09492 |
| APOC2 | -2.42473 | 9.564749 | -8.12305 | 1.39E-11 | 2.65E-10 | 16.08736 |
| NDFIP1 | -1.56671 | 8.516014 | -8.12184 | 1.40E-11 | 2.66E-10 | 16.0824 |
| TCEA1 | -1.18455 | 8.577467 | -8.12115 | 1.40E-11 | 2.67E-10 | 16.07956 |
| OR5F1 | 1.271249 | 7.133025 | 8.117796 | 1.42E-11 | 2.70E-10 | 16.06585 |
| OR2M4 | 2.29079 | 7.809731 | 8.116592 | 1.43E-11 | 2.71E-10 | 16.06092 |

| | | | | | | |
|---|---|---|---|---|---|---|
| ALDOA | -1.18027 | 8.430677 | -8.11604 | 1.43E-11 | 2.72E-10 | 16.05866 |
| VWF | -2.45427 | 10.57152 | -8.11216 | 1.45E-11 | 2.76E-10 | 16.04277 |
| RPS16 | -2.3971 | 10.04933 | -8.11137 | 1.46E-11 | 2.76E-10 | 16.03954 |
| IL32 | -1.95718 | 8.677197 | -8.11133 | 1.46E-11 | 2.76E-10 | 16.03937 |
| RDBP | -1.10245 | 7.938814 | -8.11066 | 1.46E-11 | 2.77E-10 | 16.03666 |
| PROC | -2.03251 | 9.054973 | -8.11023 | 1.47E-11 | 2.77E-10 | 16.03486 |
| ZSCAN12l | 1.008032 | 7.299009 | 8.107631 | 1.48E-11 | 2.80E-10 | 16.02424 |
| RMRP | -2.62518 | 11.49293 | -8.1075 | 1.48E-11 | 2.80E-10 | 16.0237 |
| RARRES2 | -1.69381 | 9.060965 | -8.10643 | 1.49E-11 | 2.81E-10 | 16.01934 |
| DPPA2 | 0.875832 | 6.997337 | 8.104261 | 1.50E-11 | 2.83E-10 | 16.01045 |
| C12orf50 | 1.180408 | 6.974614 | 8.102461 | 1.51E-11 | 2.85E-10 | 16.00308 |
| SPRR2B | 1.46555 | 7.004375 | 8.101126 | 1.52E-11 | 2.86E-10 | 15.99762 |
| SNORD27 | -1.42997 | 7.819091 | -8.09653 | 1.55E-11 | 2.91E-10 | 15.97881 |
| MYOZ3 | 1.463555 | 7.164768 | 8.094446 | 1.57E-11 | 2.94E-10 | 15.97027 |
| CAPRIN1 | -1.03827 | 7.61272 | -8.09349 | 1.57E-11 | 2.95E-10 | 15.96635 |
| RAN | -0.86987 | 8.301715 | -8.09258 | 1.58E-11 | 2.95E-10 | 15.96262 |
| LY6G6E | 0.964269 | 6.919118 | 8.091352 | 1.59E-11 | 2.97E-10 | 15.9576 |
| PPP2R5C | -1.02283 | 8.140151 | -8.08922 | 1.60E-11 | 2.99E-10 | 15.94886 |
| STUB1 | -1.05245 | 7.3237 | -8.08827 | 1.61E-11 | 3.00E-10 | 15.94501 |
| RPL5 | -2.22185 | 8.672307 | -8.08779 | 1.61E-11 | 3.00E-10 | 15.94302 |
| FMO6P | 0.985755 | 7.195632 | 8.085969 | 1.62E-11 | 3.02E-10 | 15.93557 |
| CYB5A | -2.02635 | 9.538334 | -8.08519 | 1.63E-11 | 3.03E-10 | 15.93237 |
| FAM86C | 1.868261 | 8.619049 | 8.085076 | 1.63E-11 | 3.03E-10 | 15.93192 |
| SLC25A28 | -1.17069 | 8.191521 | -8.08441 | 1.63E-11 | 3.03E-10 | 15.92919 |
| ZMYM2 | -1.07587 | 7.561124 | -8.08225 | 1.65E-11 | 3.06E-10 | 15.92034 |
| PPCS | -1.52461 | 8.284431 | -8.08128 | 1.65E-11 | 3.07E-10 | 15.91636 |
| ACACB | -2.4238 | 9.369778 | -8.07996 | 1.66E-11 | 3.08E-10 | 15.91099 |
| STX7 | -0.72232 | 7.191603 | -8.07987 | 1.66E-11 | 3.08E-10 | 15.91061 |
| IL5RA | 0.887463 | 7.035513 | 8.079273 | 1.67E-11 | 3.09E-10 | 15.90816 |
| MGMT | -1.68154 | 8.644123 | -8.07884 | 1.67E-11 | 3.09E-10 | 15.90638 |
| POU2F3 | 0.713499 | 6.855095 | 8.075651 | 1.69E-11 | 3.13E-10 | 15.89333 |
| SDC3 | -1.29404 | 7.978657 | -8.07453 | 1.70E-11 | 3.14E-10 | 15.88872 |
| COBLL1 | -1.6285 | 9.815466 | -8.07304 | 1.71E-11 | 3.16E-10 | 15.88266 |
| CCT3 | -1.19395 | 7.747435 | -8.0695 | 1.74E-11 | 3.20E-10 | 15.86817 |
| OR4F17 | 2.684062 | 8.104916 | 8.068554 | 1.74E-11 | 3.21E-10 | 15.86428 |
| INSL6 | 0.989279 | 6.972279 | 8.065956 | 1.76E-11 | 3.24E-10 | 15.85365 |
| ATF4 | -1.87375 | 8.699659 | -8.0649 | 1.77E-11 | 3.25E-10 | 15.84932 |
| FAM96A | -0.74705 | 7.311453 | -8.06193 | 1.79E-11 | 3.29E-10 | 15.83717 |
| KRT71 | 1.830048 | 7.254203 | 8.061468 | 1.80E-11 | 3.29E-10 | 15.83527 |
| MIR370 | 1.963937 | 7.533356 | 8.060885 | 1.80E-11 | 3.30E-10 | 15.83289 |
| MED25 | -1.82181 | 7.901087 | -8.06018 | 1.81E-11 | 3.30E-10 | 15.82999 |
| OR6F1 | 1.108147 | 6.813468 | 8.059041 | 1.82E-11 | 3.32E-10 | 15.82534 |
| MYO18B | 1.485678 | 7.188851 | 8.058848 | 1.82E-11 | 3.32E-10 | 15.82455 |
| KLK2 | 0.802256 | 6.85497 | 8.057866 | 1.82E-11 | 3.33E-10 | 15.82053 |
| PROX2 | 1.190765 | 7.016308 | 8.057552 | 1.83E-11 | 3.33E-10 | 15.81924 |
| CYC1 | -1.37375 | 7.85646 | -8.057 | 1.83E-11 | 3.33E-10 | 15.81699 |
| MGC1670 | 1.413529 | 8.690323 | 8.055972 | 1.84E-11 | 3.34E-10 | 15.81278 |
| SSX2 | 1.104099 | 7.214292 | 8.054339 | 1.85E-11 | 3.36E-10 | 15.80609 |
| A2ML1 | 0.701103 | 6.733532 | 8.053317 | 1.86E-11 | 3.37E-10 | 15.80191 |
| OR51V1 | 1.16579 | 6.994014 | 8.052437 | 1.87E-11 | 3.38E-10 | 15.79831 |
| CCDC106 | -1.82248 | 8.351979 | -8.05174 | 1.87E-11 | 3.39E-10 | 15.79544 |
| XBP1 | -1.03715 | 7.673679 | -8.05053 | 1.88E-11 | 3.40E-10 | 15.79051 |
| OR51Q1 | 2.495968 | 7.961659 | 8.047956 | 1.90E-11 | 3.43E-10 | 15.77996 |
| THRAP3 | -0.8423 | 7.798197 | -8.04795 | 1.90E-11 | 3.43E-10 | 15.77994 |
| ZNF613 | 1.021145 | 7.533693 | 8.047636 | 1.90E-11 | 3.43E-10 | 15.77865 |
| LOC10012 | -1.9014 | 8.504056 | -8.04716 | 1.91E-11 | 3.44E-10 | 15.77669 |
| ARD1A | -1.63163 | 8.511038 | -8.0453 | 1.92E-11 | 3.46E-10 | 15.7691 |
| C1R | -1.86757 | 9.050339 | -8.04488 | 1.93E-11 | 3.46E-10 | 15.76739 |

| | | | | | | |
|---|---|---|---|---|---|---|
| PLA2G2E | 1.6183 | 7.345349 | 8.04481 | 1.93E-11 | 3.46E-10 | 15.76709 |
| GRHPR | -1.2188 | 7.980347 | -8.04424 | 1.93E-11 | 3.47E-10 | 15.76473 |
| PAH | -1.7928 | 8.227215 | -8.04238 | 1.95E-11 | 3.49E-10 | 15.75712 |
| PSMA5 | -1.71288 | 8.123884 | -8.04229 | 1.95E-11 | 3.49E-10 | 15.75678 |
| HOXD13 | 1.190478 | 7.004402 | 8.041933 | 1.95E-11 | 3.49E-10 | 15.75531 |
| GGA1 | 1.241011 | 8.815983 | 8.041555 | 1.95E-11 | 3.49E-10 | 15.75376 |
| AKR1C2 | -1.82422 | 9.369277 | -8.04151 | 1.95E-11 | 3.49E-10 | 15.75359 |
| ZNF674 | 1.71196 | 11.40713 | 8.040085 | 1.96E-11 | 3.51E-10 | 15.74774 |
| F2R | 0.810616 | 11.37333 | 8.040057 | 1.96E-11 | 3.51E-10 | 15.74763 |
| CPB2 | -1.25646 | 7.653131 | -8.03811 | 1.98E-11 | 3.53E-10 | 15.73965 |
| PRDX5 | -1.224 | 7.873236 | -8.03749 | 1.99E-11 | 3.54E-10 | 15.73713 |
| FIZ1 | -1.07976 | 7.687569 | -8.03748 | 1.99E-11 | 3.54E-10 | 15.7371 |
| SEC1 | 1.550648 | 7.315692 | 8.036905 | 1.99E-11 | 3.54E-10 | 15.73473 |
| TM4SF4 | -2.73849 | 10.42019 | -8.0339 | 2.02E-11 | 3.58E-10 | 15.72242 |
| GTF2IRD2 | 1.77215 | 10.66953 | 8.032459 | 2.03E-11 | 3.60E-10 | 15.71653 |
| SOD1 | -1.88766 | 8.83149 | -8.03222 | 2.03E-11 | 3.60E-10 | 15.71553 |
| GALK1 | -2.23165 | 9.688896 | -8.03127 | 2.04E-11 | 3.61E-10 | 15.71166 |
| AGXT | -2.59721 | 9.853196 | -8.02943 | 2.05E-11 | 3.64E-10 | 15.70414 |
| FOLR4 | 1.259478 | 7.068831 | 8.028288 | 2.06E-11 | 3.65E-10 | 15.69945 |
| GSTO1 | -1.65078 | 8.801136 | -8.02621 | 2.08E-11 | 3.68E-10 | 15.69095 |
| GLTSCR1 | -1.41106 | 7.930933 | -8.02571 | 2.09E-11 | 3.68E-10 | 15.68889 |
| NUCB2 | -1.59312 | 7.928132 | -8.02446 | 2.10E-11 | 3.70E-10 | 15.68378 |
| SNORA36 | -2.11162 | 8.601311 | -8.02322 | 2.11E-11 | 3.71E-10 | 15.6787 |
| TPT1 | -2.28966 | 10.71028 | -8.02203 | 2.12E-11 | 3.73E-10 | 15.67385 |
| A2BP1 | 2.240027 | 7.781494 | 8.020605 | 2.13E-11 | 3.75E-10 | 15.668 |
| OR2T3 | 1.494371 | 7.201288 | 8.020509 | 2.13E-11 | 3.75E-10 | 15.66761 |
| SLC41A2 | -1.36737 | 7.856052 | -8.02042 | 2.13E-11 | 3.75E-10 | 15.66726 |
| ACTL6B | 2.371336 | 7.801311 | 8.020157 | 2.13E-11 | 3.75E-10 | 15.66617 |
| DDX25 | 0.947501 | 7.003357 | 8.019627 | 2.14E-11 | 3.75E-10 | 15.664 |
| GATA3 | 1.513403 | 7.239617 | 8.019296 | 2.14E-11 | 3.75E-10 | 15.66264 |
| C11orf48 | -0.89906 | 7.453896 | -8.01803 | 2.15E-11 | 3.77E-10 | 15.65746 |
| DHX8 | -1.2246 | 8.282018 | -8.01746 | 2.16E-11 | 3.78E-10 | 15.65512 |
| SLC39A3 | -1.09055 | 8.297503 | -8.01401 | 2.19E-11 | 3.82E-10 | 15.641 |
| OR6Y1 | 0.880501 | 6.924751 | 8.013887 | 2.19E-11 | 3.82E-10 | 15.6405 |
| CIB2 | 1.047391 | 7.799689 | 8.01382 | 2.19E-11 | 3.82E-10 | 15.64023 |
| PSCA | 2.26449 | 7.999871 | 8.012819 | 2.20E-11 | 3.83E-10 | 15.63613 |
| ADRB1 | 1.354368 | 7.148035 | 8.007914 | 2.25E-11 | 3.91E-10 | 15.61606 |
| OR5A1 | 0.938775 | 6.937273 | 8.007386 | 2.25E-11 | 3.91E-10 | 15.61389 |
| SNORA25 | -1.54331 | 8.063346 | -8.00702 | 2.25E-11 | 3.92E-10 | 15.61238 |
| KIR2DL3 | 2.359843 | 7.970459 | 8.00669 | 2.26E-11 | 3.92E-10 | 15.61104 |
| GOLPH3 | -0.84289 | 7.557577 | -8.00668 | 2.26E-11 | 3.92E-10 | 15.61099 |
| BGN | -2.69407 | 9.713761 | -8.00466 | 2.28E-11 | 3.95E-10 | 15.60274 |
| CD46 | -0.79103 | 7.329277 | -8.0018 | 2.30E-11 | 3.99E-10 | 15.59101 |
| XRCC1 | -1.04574 | 7.338817 | -7.99909 | 2.33E-11 | 4.03E-10 | 15.57994 |
| SERPINA1 | -1.67646 | 9.209878 | -7.99799 | 2.34E-11 | 4.04E-10 | 15.57543 |
| PSORS1C2 | 2.633096 | 8.167319 | 7.997174 | 2.35E-11 | 4.05E-10 | 15.57209 |
| SNORA55 | -2.00279 | 8.874651 | -7.99714 | 2.35E-11 | 4.05E-10 | 15.57194 |
| SDF4 | -0.98216 | 7.978027 | -7.99095 | 2.41E-11 | 4.15E-10 | 15.54662 |
| UTS2D | 1.087827 | 7.245601 | 7.987623 | 2.44E-11 | 4.20E-10 | 15.533 |
| VKORC1L1 | -0.8431 | 7.33796 | -7.98695 | 2.45E-11 | 4.21E-10 | 15.53025 |
| PRAMEF18 | 2.420124 | 7.757334 | 7.986714 | 2.45E-11 | 4.21E-10 | 15.52928 |
| HAAO | -1.2108 | 7.85947 | -7.9865 | 2.46E-11 | 4.21E-10 | 15.52841 |
| OR52B6 | 2.829958 | 8.129794 | 7.985545 | 2.46E-11 | 4.22E-10 | 15.52449 |
| MEI1 | 1.225828 | 7.454085 | 7.984814 | 2.47E-11 | 4.23E-10 | 15.5215 |
| DEFB129 | 1.526786 | 7.204745 | 7.981597 | 2.51E-11 | 4.28E-10 | 15.50833 |
| NPC2 | -2.12296 | 9.646691 | -7.97959 | 2.53E-11 | 4.31E-10 | 15.50014 |
| TBCA | -1.94663 | 10.75957 | -7.97828 | 2.54E-11 | 4.33E-10 | 15.49477 |
| C7orf4 | 0.78373 | 6.792665 | 7.976507 | 2.56E-11 | 4.36E-10 | 15.4875 |

| | | | | | | |
|---|---|---|---|---|---|---|
| OR2B11 | 2.51408 | 7.715366 | 7.975285 | 2.57E-11 | 4.37E-10 | 15.48249 |
| MYBPC1 | 0.896547 | 6.975539 | 7.973665 | 2.59E-11 | 4.40E-10 | 15.47586 |
| RPL4 | -1.42671 | 8.321018 | -7.97024 | 2.63E-11 | 4.45E-10 | 15.46186 |
| CP | -2.04869 | 9.366425 | -7.96981 | 2.63E-11 | 4.45E-10 | 15.46008 |
| C2orf29 | -1.62401 | 8.403629 | -7.96949 | 2.64E-11 | 4.45E-10 | 15.45879 |
| GP6 | 1.422729 | 7.384288 | 7.969378 | 2.64E-11 | 4.45E-10 | 15.45832 |
| BARHL1 | 1.478544 | 7.146165 | 7.968913 | 2.64E-11 | 4.46E-10 | 15.45641 |
| HYI | -1.43476 | 7.726826 | -7.96801 | 2.65E-11 | 4.47E-10 | 15.45272 |
| RPN2 | -2.11944 | 8.614431 | -7.96763 | 2.66E-11 | 4.48E-10 | 15.45115 |
| SNORA73 | -2.13116 | 9.881873 | -7.96573 | 2.68E-11 | 4.50E-10 | 15.44338 |
| FGFR3 | -1.24122 | 9.651274 | -7.96492 | 2.69E-11 | 4.51E-10 | 15.44007 |
| LRRC10 | 0.946918 | 7.067889 | 7.964359 | 2.69E-11 | 4.52E-10 | 15.43777 |
| HSPA9 | -1.7266 | 9.242579 | -7.96267 | 2.71E-11 | 4.55E-10 | 15.43084 |
| FAM83A | 0.9737 | 6.961205 | 7.961346 | 2.73E-11 | 4.56E-10 | 15.42544 |
| YIPF3 | -1.28712 | 8.583712 | -7.96127 | 2.73E-11 | 4.56E-10 | 15.42512 |
| SNORA1 | -1.25552 | 7.655915 | -7.96126 | 2.73E-11 | 4.56E-10 | 15.4251 |
| SEC31A | -1.05412 | 7.715383 | -7.96123 | 2.73E-11 | 4.56E-10 | 15.42495 |
| EFNA1 | -1.97963 | 8.375154 | -7.96108 | 2.73E-11 | 4.56E-10 | 15.42437 |
| NME5 | 1.529074 | 7.263181 | 7.956916 | 2.78E-11 | 4.63E-10 | 15.40731 |
| ZNF652 | 1.318681 | 10.76766 | 7.954596 | 2.80E-11 | 4.68E-10 | 15.39781 |
| ARHGEF2 | -0.83369 | 7.822401 | -7.95239 | 2.83E-11 | 4.72E-10 | 15.38877 |
| KCNS1 | 1.698999 | 7.555846 | 7.946371 | 2.90E-11 | 4.83E-10 | 15.36415 |
| STK36 | -1.56411 | 8.540763 | -7.94614 | 2.90E-11 | 4.83E-10 | 15.36321 |
| MIXL1 | 1.710948 | 7.29154 | 7.945509 | 2.91E-11 | 4.84E-10 | 15.36062 |
| SELV | 2.058862 | 7.634942 | 7.942962 | 2.94E-11 | 4.88E-10 | 15.3502 |
| BRIX1 | -1.17692 | 7.665319 | -7.94284 | 2.94E-11 | 4.88E-10 | 15.34969 |
| RPL37A | -1.26764 | 8.178821 | -7.94113 | 2.97E-11 | 4.91E-10 | 15.34269 |
| BVES | 1.05447 | 7.103262 | 7.937149 | 3.02E-11 | 4.99E-10 | 15.3264 |
| TACR3 | 1.357097 | 7.401447 | 7.936331 | 3.03E-11 | 5.01E-10 | 15.32305 |
| IFITM2 | -1.7397 | 10.56712 | -7.93502 | 3.04E-11 | 5.03E-10 | 15.31768 |
| SNORD85 | -2.36451 | 10.07054 | -7.93356 | 3.06E-11 | 5.05E-10 | 15.31173 |
| STRA13 | -1.66579 | 8.85295 | -7.93345 | 3.06E-11 | 5.05E-10 | 15.31126 |
| CSF3 | 1.603994 | 7.340303 | 7.933 | 3.07E-11 | 5.06E-10 | 15.30942 |
| PICK1 | -1.36983 | 8.03748 | -7.93231 | 3.08E-11 | 5.07E-10 | 15.30659 |
| ATP5I | -1.98007 | 10.64608 | -7.92727 | 3.14E-11 | 5.17E-10 | 15.28599 |
| KRT13 | 1.854009 | 7.475038 | 7.925812 | 3.16E-11 | 5.19E-10 | 15.28 |
| ZNF280B | 1.384511 | 7.226531 | 7.925794 | 3.16E-11 | 5.19E-10 | 15.27993 |
| NEFL | 2.738025 | 8.182715 | 7.925223 | 3.17E-11 | 5.20E-10 | 15.27759 |
| HORMAD: | 1.481665 | 7.359656 | 7.924805 | 3.17E-11 | 5.21E-10 | 15.27588 |
| MIR575 | 1.446567 | 7.19712 | 7.922976 | 3.20E-11 | 5.24E-10 | 15.2684 |
| TOMM20 | -1.3229 | 8.907682 | -7.92296 | 3.20E-11 | 5.24E-10 | 15.26833 |
| TSSC4 | -0.8678 | 7.499432 | -7.92162 | 3.22E-11 | 5.26E-10 | 15.26284 |
| ESR2 | 0.873492 | 6.85682 | 7.92067 | 3.23E-11 | 5.28E-10 | 15.25896 |
| SLC24A2 | 1.927026 | 7.505103 | 7.920494 | 3.23E-11 | 5.28E-10 | 15.25824 |
| SERPINC1 | -2.3676 | 9.773591 | -7.91942 | 3.25E-11 | 5.30E-10 | 15.25385 |
| BAT3 | -1.04606 | 8.487195 | -7.91871 | 3.26E-11 | 5.31E-10 | 15.25094 |
| C9orf129 | 1.034979 | 6.935492 | 7.917213 | 3.28E-11 | 5.34E-10 | 15.24481 |
| B2M | -1.41758 | 7.935239 | -7.91599 | 3.29E-11 | 5.36E-10 | 15.23981 |
| LAMP2 | -0.96052 | 7.60103 | -7.91517 | 3.30E-11 | 5.38E-10 | 15.23646 |
| COLQ | 0.733909 | 7.114533 | 7.914823 | 3.31E-11 | 5.38E-10 | 15.23503 |
| SLC26A8 | 1.189303 | 7.197944 | 7.912897 | 3.34E-11 | 5.42E-10 | 15.22714 |
| TEKT3 | 0.911048 | 6.834947 | 7.911887 | 3.35E-11 | 5.43E-10 | 15.22301 |
| GTF2I | -1.35517 | 8.056711 | -7.91183 | 3.35E-11 | 5.43E-10 | 15.22279 |
| TCHHL1 | 0.764302 | 6.732984 | 7.911525 | 3.35E-11 | 5.44E-10 | 15.22153 |
| OGT | -0.82368 | 7.437065 | -7.91125 | 3.36E-11 | 5.44E-10 | 15.2204 |
| WFDC5 | 1.113166 | 7.128846 | 7.91053 | 3.37E-11 | 5.45E-10 | 15.21746 |
| SP7 | 0.88399 | 6.882048 | 7.908739 | 3.39E-11 | 5.48E-10 | 15.21013 |
| HIST1H2B | -1.47326 | 8.400601 | -7.90807 | 3.40E-11 | 5.49E-10 | 15.20739 |

| | | | | | | |
|---|---|---|---|---|---|---|
| SNORA63 | -1.57437 | 8.588514 | -7.90764 | 3.41E-11 | 5.50E-10 | 15.20565 |
| ZNF804A | 1.809321 | 7.595968 | 7.907367 | 3.41E-11 | 5.50E-10 | 15.20451 |
| INO80B | -1.15158 | 7.640078 | -7.90688 | 3.42E-11 | 5.51E-10 | 15.2025 |
| TIMP2 | -1.31532 | 7.827789 | -7.90616 | 3.43E-11 | 5.52E-10 | 15.19958 |
| PAWR | -1.02786 | 7.755416 | -7.90483 | 3.45E-11 | 5.55E-10 | 15.19412 |
| MAN2B2 | -0.76343 | 8.264995 | -7.90351 | 3.47E-11 | 5.57E-10 | 15.18873 |
| NINJ1 | -1.07429 | 7.925909 | -7.90349 | 3.47E-11 | 5.57E-10 | 15.18863 |
| SNORD89 | -2.3089 | 10.70138 | -7.89936 | 3.53E-11 | 5.66E-10 | 15.17175 |
| GAD2 | 1.35976 | 6.973657 | 7.899319 | 3.53E-11 | 5.66E-10 | 15.17158 |
| CYP4V2 | -1.40898 | 8.153298 | -7.89896 | 3.53E-11 | 5.66E-10 | 15.17009 |
| TNPO2 | -1.16757 | 7.804026 | -7.89693 | 3.56E-11 | 5.71E-10 | 15.16178 |
| GOLGA6B | 0.919237 | 7.138742 | 7.894722 | 3.60E-11 | 5.75E-10 | 15.15276 |
| ITGB4BP | -0.83798 | 7.205637 | -7.89431 | 3.60E-11 | 5.76E-10 | 15.15106 |
| NCRNA00 | 0.812136 | 6.854199 | 7.89386 | 3.61E-11 | 5.77E-10 | 15.14924 |
| OR9I1 | 1.112874 | 7.033972 | 7.891661 | 3.64E-11 | 5.81E-10 | 15.14024 |
| MIR96 | 0.899233 | 6.872439 | 7.889674 | 3.67E-11 | 5.86E-10 | 15.1321 |
| RPS25 | -2.91874 | 10.62391 | -7.88845 | 3.69E-11 | 5.88E-10 | 15.1271 |
| CDC37 | -2.49141 | 11.13429 | -7.88368 | 3.77E-11 | 6.00E-10 | 15.10756 |
| NOP14 | -1.22606 | 8.477273 | -7.88268 | 3.78E-11 | 6.02E-10 | 15.10348 |
| FAM153B | 2.184236 | 9.902379 | 7.881024 | 3.81E-11 | 6.05E-10 | 15.09671 |
| DSP | -0.94782 | 7.779726 | -7.88013 | 3.82E-11 | 6.07E-10 | 15.09306 |
| KRT10 | -1.00783 | 7.820703 | -7.87942 | 3.83E-11 | 6.09E-10 | 15.09014 |
| HACL1 | -1.55121 | 7.810551 | -7.87878 | 3.84E-11 | 6.10E-10 | 15.08751 |
| LONRF3 | -0.77628 | 7.348641 | -7.87737 | 3.87E-11 | 6.13E-10 | 15.08176 |
| LOR | 2.071813 | 7.641454 | 7.876343 | 3.88E-11 | 6.15E-10 | 15.07755 |
| CNOT2 | -0.71073 | 7.064478 | -7.87514 | 3.90E-11 | 6.18E-10 | 15.07262 |
| PEA15 | -1.85826 | 8.370982 | -7.87229 | 3.95E-11 | 6.24E-10 | 15.06098 |
| TCEAL6 | 1.150879 | 6.991486 | 7.872091 | 3.95E-11 | 6.25E-10 | 15.06015 |
| PTPRT | 1.476199 | 7.291869 | 7.871011 | 3.97E-11 | 6.27E-10 | 15.05573 |
| TTLL10 | 1.530849 | 7.201842 | 7.870761 | 3.98E-11 | 6.27E-10 | 15.05471 |
| SNORA18 | -1.98012 | 9.141784 | -7.87002 | 3.99E-11 | 6.29E-10 | 15.05166 |
| XAGE1 | 1.179413 | 7.299696 | 7.869202 | 4.00E-11 | 6.30E-10 | 15.04833 |
| LAMA1 | 1.422419 | 7.155937 | 7.86539 | 4.06E-11 | 6.40E-10 | 15.03273 |
| DNAJC17 | -1.20418 | 8.109781 | -7.86325 | 4.10E-11 | 6.45E-10 | 15.02396 |
| LPO | 1.588954 | 7.202119 | 7.863137 | 4.10E-11 | 6.45E-10 | 15.02351 |
| HSPA8 | -1.34322 | 8.806701 | -7.85975 | 4.16E-11 | 6.53E-10 | 15.00967 |
| FKBP1P1 | 1.351831 | 7.130433 | 7.859666 | 4.16E-11 | 6.53E-10 | 15.00931 |
| OR6B1 | 1.505528 | 7.18676 | 7.85897 | 4.17E-11 | 6.54E-10 | 15.00646 |
| PRL | 0.982637 | 6.917925 | 7.858419 | 4.18E-11 | 6.55E-10 | 15.0042 |
| SERPINB9 | 1.356242 | 8.273022 | 7.85836 | 4.19E-11 | 6.55E-10 | 15.00396 |
| HADHA | -1.56763 | 8.866435 | -7.8569 | 4.21E-11 | 6.58E-10 | 14.998 |
| C7orf50 | -1.76644 | 8.995714 | -7.85605 | 4.23E-11 | 6.60E-10 | 14.99451 |
| TXNL1 | -1.22719 | 8.270645 | -7.8556 | 4.23E-11 | 6.61E-10 | 14.99265 |
| HNRNPUL | -1.28699 | 8.174109 | -7.85499 | 4.24E-11 | 6.62E-10 | 14.99017 |
| HNRNPAE | -1.2677 | 8.948655 | -7.8509 | 4.32E-11 | 6.73E-10 | 14.97344 |
| SFRS6 | -1.48143 | 8.112505 | -7.84927 | 4.35E-11 | 6.76E-10 | 14.96678 |
| LOC10013 | 0.896429 | 6.851263 | 7.848973 | 4.35E-11 | 6.77E-10 | 14.96555 |
| MSN | -1.15214 | 8.042777 | -7.84744 | 4.38E-11 | 6.79E-10 | 14.95928 |
| TSPAN33 | -1.7794 | 8.759533 | -7.84743 | 4.38E-11 | 6.79E-10 | 14.95922 |
| KRT6C | 1.675811 | 7.342605 | 7.847267 | 4.38E-11 | 6.79E-10 | 14.95857 |
| MMRN1 | 1.870788 | 8.233976 | 7.845667 | 4.41E-11 | 6.83E-10 | 14.95202 |
| PIK3R1 | -0.78612 | 7.733668 | -7.84472 | 4.43E-11 | 6.85E-10 | 14.94814 |
| C2orf40 | 1.124896 | 7.19377 | 7.844701 | 4.43E-11 | 6.85E-10 | 14.94807 |
| DDT | -1.81599 | 11.23659 | -7.84431 | 4.44E-11 | 6.85E-10 | 14.94648 |
| ZSCAN4 | 1.442341 | 7.214229 | 7.84316 | 4.46E-11 | 6.88E-10 | 14.94176 |
| NELL1 | 1.02266 | 6.913708 | 7.841604 | 4.49E-11 | 6.92E-10 | 14.9354 |
| PAFAH1B3 | -1.69074 | 8.480828 | -7.84074 | 4.50E-11 | 6.94E-10 | 14.93188 |
| RASSF6 | 0.872047 | 7.377694 | 7.840131 | 4.52E-11 | 6.95E-10 | 14.92937 |

| | | | | | | |
|---|---|---|---|---|---|---|
| LOC15221 | 0.882172 | 6.956184 | 7.837446 | 4.57E-11 | 7.02E-10 | 14.91839 |
| CLDN10 | 0.758474 | 6.80254 | 7.835199 | 4.61E-11 | 7.08E-10 | 14.90919 |
| RABEPK | -1.37101 | 8.231848 | -7.83386 | 4.64E-11 | 7.11E-10 | 14.90372 |
| TCEB3B | 2.242019 | 7.733032 | 7.83277 | 4.66E-11 | 7.13E-10 | 14.89925 |
| DSCR10 | 2.24763 | 7.938212 | 7.831302 | 4.68E-11 | 7.16E-10 | 14.89325 |
| PHF3 | -0.83637 | 7.445382 | -7.82981 | 4.71E-11 | 7.20E-10 | 14.88712 |
| HOXA2 | 1.636325 | 7.746731 | 7.828506 | 4.74E-11 | 7.23E-10 | 14.88181 |
| LOC64814 | 2.40443 | 7.966001 | 7.828397 | 4.74E-11 | 7.23E-10 | 14.88136 |
| LOC10012 | 0.788784 | 6.959816 | 7.826907 | 4.77E-11 | 7.27E-10 | 14.87527 |
| PRR20E | 1.141408 | 7.023864 | 7.823332 | 4.84E-11 | 7.37E-10 | 14.86064 |
| OR4K5 | 1.276974 | 6.967601 | 7.822245 | 4.86E-11 | 7.40E-10 | 14.85619 |
| DRAP1 | -1.2062 | 9.025801 | -7.822 | 4.87E-11 | 7.40E-10 | 14.8552 |
| RPL26 | -2.09705 | 8.749161 | -7.82078 | 4.89E-11 | 7.43E-10 | 14.85022 |
| PPFIA4 | 2.246143 | 8.114533 | 7.820677 | 4.90E-11 | 7.43E-10 | 14.84978 |
| KLK8 | 1.387745 | 7.040276 | 7.819453 | 4.92E-11 | 7.47E-10 | 14.84477 |
| VBP1 | -0.86384 | 7.435687 | -7.81914 | 4.93E-11 | 7.47E-10 | 14.84349 |
| DPP6 | 0.940228 | 6.937087 | 7.818394 | 4.94E-11 | 7.49E-10 | 14.84043 |
| SCGB1A1 | 2.706388 | 8.417822 | 7.816123 | 4.99E-11 | 7.56E-10 | 14.83114 |
| PCBD2 | 0.935444 | 8.065068 | 7.815336 | 5.01E-11 | 7.57E-10 | 14.82792 |
| SEBOX | 1.184839 | 7.235752 | 7.815095 | 5.01E-11 | 7.57E-10 | 14.82694 |
| PRAMEF1( | 1.871813 | 8.054422 | 7.814981 | 5.01E-11 | 7.57E-10 | 14.82647 |
| A2M | -2.31697 | 8.57384 | -7.81489 | 5.02E-11 | 7.57E-10 | 14.8261 |
| MIR422A | 1.148361 | 7.056131 | 7.814847 | 5.02E-11 | 7.57E-10 | 14.82592 |
| SYN1 | 1.208617 | 7.359236 | 7.812515 | 5.07E-11 | 7.64E-10 | 14.81638 |
| AUH | -1.71895 | 8.339778 | -7.81057 | 5.11E-11 | 7.69E-10 | 14.80841 |
| ANKRD38 | 1.442689 | 7.485389 | 7.809712 | 5.13E-11 | 7.71E-10 | 14.80492 |
| VTN | -1.85996 | 8.300473 | -7.80892 | 5.14E-11 | 7.73E-10 | 14.80167 |
| KRTAP5-2 | 1.148256 | 7.044202 | 7.807349 | 5.18E-11 | 7.77E-10 | 14.79525 |
| RNF165 | 2.555174 | 9.148652 | 7.806547 | 5.19E-11 | 7.79E-10 | 14.79197 |
| KRTAP10- | 1.798243 | 7.350038 | 7.80638 | 5.20E-11 | 7.79E-10 | 14.79128 |
| C4BPA | -1.90389 | 8.629236 | -7.80612 | 5.20E-11 | 7.80E-10 | 14.79022 |
| C6orf59 | 0.873749 | 7.233389 | 7.805912 | 5.21E-11 | 7.80E-10 | 14.78937 |
| MAPT | 1.170798 | 7.466544 | 7.802641 | 5.28E-11 | 7.90E-10 | 14.77598 |
| VSTM2A | 0.981926 | 7.029374 | 7.801936 | 5.29E-11 | 7.92E-10 | 14.7731 |
| ARPM2 | 1.123513 | 6.919376 | 7.799148 | 5.36E-11 | 8.00E-10 | 14.7617 |
| CDC42EP1 | -1.21015 | 7.902317 | -7.79871 | 5.37E-11 | 8.01E-10 | 14.75989 |
| SNORA24 | -2.53848 | 10.95729 | -7.79823 | 5.38E-11 | 8.02E-10 | 14.75795 |
| SPRR3 | 1.522936 | 7.169716 | 7.798122 | 5.38E-11 | 8.02E-10 | 14.7575 |
| SNORD38 | -2.15905 | 9.263892 | -7.79687 | 5.41E-11 | 8.06E-10 | 14.75239 |
| RET | 1.346481 | 7.484176 | 7.796413 | 5.42E-11 | 8.07E-10 | 14.75051 |
| HSPA4 | -1.22048 | 7.906048 | -7.79608 | 5.42E-11 | 8.07E-10 | 14.74916 |
| TXNRD2 | -1.5752 | 9.19178 | -7.7945 | 5.46E-11 | 8.12E-10 | 14.7427 |
| NDUFA2 | -1.33236 | 9.142304 | -7.7904 | 5.55E-11 | 8.25E-10 | 14.7259 |
| ADD1 | -1.66854 | 8.189836 | -7.78985 | 5.57E-11 | 8.26E-10 | 14.72366 |
| ZNF223 | 1.983639 | 9.680748 | 7.789664 | 5.57E-11 | 8.26E-10 | 14.7229 |
| OR8D2 | 1.042703 | 6.967501 | 7.789422 | 5.58E-11 | 8.26E-10 | 14.72191 |
| VIM | -1.39686 | 8.383081 | -7.78935 | 5.58E-11 | 8.26E-10 | 14.7216 |
| CCDC19 | 1.730497 | 7.61957 | 7.787055 | 5.63E-11 | 8.33E-10 | 14.71223 |
| ECGF1 | -2.03671 | 9.279611 | -7.78668 | 5.64E-11 | 8.34E-10 | 14.71067 |
| LOC44104 | 1.01631 | 7.060426 | 7.786602 | 5.64E-11 | 8.34E-10 | 14.71037 |
| CYP2F1 | 0.893385 | 6.952713 | 7.786027 | 5.66E-11 | 8.35E-10 | 14.70802 |
| OR10H2 | 1.24458 | 7.118527 | 7.785826 | 5.66E-11 | 8.35E-10 | 14.7072 |
| PDE6H | 0.740051 | 6.787642 | 7.782525 | 5.74E-11 | 8.46E-10 | 14.6937 |
| PRAP1 | -2.55126 | 9.154369 | -7.78158 | 5.76E-11 | 8.49E-10 | 14.68982 |
| COPB2 | -1.60031 | 9.853344 | -7.78112 | 5.77E-11 | 8.50E-10 | 14.68794 |
| TRAM1 | -1.66503 | 8.58007 | -7.78093 | 5.78E-11 | 8.50E-10 | 14.68715 |
| GJA10 | 0.965464 | 6.852124 | 7.779925 | 5.80E-11 | 8.52E-10 | 14.68306 |
| LOC10013 | 0.794594 | 7.097394 | 7.778489 | 5.84E-11 | 8.57E-10 | 14.67718 |

| | | | | | | |
|---|---|---|---|---|---|---|
| RETNLB | 1.352271 | 7.232812 | 7.778006 | 5.85E-11 | 8.58E-10 | 14.67521 |
| SS18L2 | -1.92155 | 8.489219 | -7.7777 | 5.86E-11 | 8.58E-10 | 14.67397 |
| KRTAP1-3 | 1.609245 | 7.266819 | 7.776675 | 5.88E-11 | 8.61E-10 | 14.66976 |
| LOC10013 | 0.760864 | 7.126975 | 7.77652 | 5.88E-11 | 8.61E-10 | 14.66913 |
| RBM46 | 0.87574 | 6.913309 | 7.773648 | 5.96E-11 | 8.71E-10 | 14.65738 |
| RPS4X | -1.24405 | 7.791937 | -7.77294 | 5.97E-11 | 8.73E-10 | 14.65448 |
| MED16 | -1.32994 | 9.714324 | -7.77235 | 5.99E-11 | 8.75E-10 | 14.65208 |
| C10orf90 | 1.006384 | 6.970476 | 7.771851 | 6.00E-11 | 8.76E-10 | 14.65003 |
| CNOT1 | -0.80709 | 7.36621 | -7.76934 | 6.06E-11 | 8.84E-10 | 14.63975 |
| C11orf85 | 0.85322 | 6.888558 | 7.768471 | 6.09E-11 | 8.86E-10 | 14.63621 |
| N6AMT1 | 1.486086 | 8.406985 | 7.767291 | 6.12E-11 | 8.90E-10 | 14.63138 |
| OR8G2 | 0.868396 | 7.072545 | 7.767194 | 6.12E-11 | 8.90E-10 | 14.63098 |
| TMEM106 | 1.752175 | 9.072838 | 7.764554 | 6.19E-11 | 8.99E-10 | 14.62018 |
| RPL23AP7 | 1.266731 | 7.95876 | 7.762837 | 6.23E-11 | 9.05E-10 | 14.61316 |
| OR4E2 | 1.624726 | 7.192539 | 7.762447 | 6.24E-11 | 9.05E-10 | 14.61156 |
| RHOB | -1.66699 | 8.382833 | -7.76081 | 6.28E-11 | 9.10E-10 | 14.60486 |
| PCDHB17 | 1.747014 | 7.817684 | 7.760309 | 6.30E-11 | 9.12E-10 | 14.60282 |
| CDK3 | 1.687305 | 8.970844 | 7.759896 | 6.31E-11 | 9.13E-10 | 14.60113 |
| SYF2 | -0.72779 | 7.331328 | -7.75779 | 6.36E-11 | 9.20E-10 | 14.59253 |
| GBP2 | -1.41691 | 7.709455 | -7.75583 | 6.41E-11 | 9.26E-10 | 14.58452 |
| CCDC127 | 1.694864 | 8.637627 | 7.755456 | 6.42E-11 | 9.27E-10 | 14.58297 |
| DSG4 | 0.979075 | 7.05779 | 7.7543 | 6.45E-11 | 9.30E-10 | 14.57824 |
| GUK1 | -0.89521 | 7.461697 | -7.75427 | 6.46E-11 | 9.30E-10 | 14.57812 |
| TSKU | -1.42327 | 10.59347 | -7.75349 | 6.48E-11 | 9.32E-10 | 14.57492 |
| S100A2 | 1.093008 | 6.984521 | 7.752851 | 6.49E-11 | 9.34E-10 | 14.57232 |
| FN1 | -2.1968 | 9.462198 | -7.75246 | 6.50E-11 | 9.35E-10 | 14.57073 |
| RNU4ATA | -1.77341 | 11.2877 | -7.75233 | 6.51E-11 | 9.35E-10 | 14.57019 |
| TMEM150 | -1.37606 | 7.933282 | -7.7499 | 6.57E-11 | 9.43E-10 | 14.56023 |
| NEUROD1 | 0.711444 | 6.878369 | 7.749079 | 6.60E-11 | 9.45E-10 | 14.55689 |
| CRIM1 | -1.05115 | 7.872289 | -7.74615 | 6.68E-11 | 9.56E-10 | 14.54493 |
| CAPN9 | 2.330285 | 8.073692 | 7.74576 | 6.69E-11 | 9.57E-10 | 14.54331 |
| TMEM176 | -1.08487 | 7.356847 | -7.74448 | 6.72E-11 | 9.62E-10 | 14.53809 |
| TMEM146 | 1.808671 | 7.202449 | 7.743158 | 6.76E-11 | 9.66E-10 | 14.53267 |
| OR2L2 | 1.333343 | 7.061041 | 7.74278 | 6.77E-11 | 9.67E-10 | 14.53113 |
| TBC1D26 | 0.856863 | 6.824405 | 7.741054 | 6.82E-11 | 9.72E-10 | 14.52407 |
| ZFR | -0.80508 | 7.324567 | -7.73948 | 6.87E-11 | 9.78E-10 | 14.51763 |
| RPS13 | -1.21949 | 7.839782 | -7.73868 | 6.89E-11 | 9.80E-10 | 14.51436 |
| SLC26A6 | -0.96169 | 7.601542 | -7.7338 | 7.03E-11 | 9.99E-10 | 14.49442 |
| TFPI | -1.18917 | 7.988395 | -7.73364 | 7.03E-11 | 9.99E-10 | 14.49373 |
| C3orf36 | 1.301738 | 7.610417 | 7.733571 | 7.04E-11 | 9.99E-10 | 14.49347 |
| LRRN4CL | 1.422321 | 7.323508 | 7.732927 | 7.06E-11 | 1.00E-09 | 14.49083 |
| FLJ42627 | 1.530533 | 7.970497 | 7.732827 | 7.06E-11 | 1.00E-09 | 14.49042 |
| LMF2 | -1.12011 | 7.845751 | -7.73264 | 7.06E-11 | 1.00E-09 | 14.48964 |
| ZNF808 | 1.270948 | 7.783142 | 7.731689 | 7.09E-11 | 1.00E-09 | 14.48577 |
| OR4F4 | 1.342261 | 7.180722 | 7.730383 | 7.13E-11 | 1.01E-09 | 14.48043 |
| FOXI2 | 1.191768 | 7.079542 | 7.729551 | 7.16E-11 | 1.01E-09 | 14.47703 |
| MIR758 | 1.351531 | 7.560399 | 7.729147 | 7.17E-11 | 1.01E-09 | 14.47537 |
| HLA-DMA | -1.64221 | 7.912372 | -7.72812 | 7.20E-11 | 1.02E-09 | 14.47116 |
| MUSK | 0.74291 | 6.87098 | 7.728019 | 7.20E-11 | 1.02E-09 | 14.47076 |
| ACSS2 | -1.65587 | 8.344456 | -7.72798 | 7.20E-11 | 1.02E-09 | 14.47061 |
| SLC13A4 | 0.988442 | 7.066354 | 7.726169 | 7.26E-11 | 1.02E-09 | 14.46319 |
| RGS17 | 1.074896 | 7.043205 | 7.725424 | 7.28E-11 | 1.02E-09 | 14.46015 |
| SF3A3 | -2.04675 | 9.361162 | -7.72354 | 7.34E-11 | 1.03E-09 | 14.45244 |
| APEX1 | -0.81783 | 8.150644 | -7.72282 | 7.36E-11 | 1.03E-09 | 14.44951 |
| C6orf25 | 1.185801 | 7.061668 | 7.721088 | 7.41E-11 | 1.04E-09 | 14.44242 |
| SNX32 | 1.874967 | 7.753176 | 7.720815 | 7.42E-11 | 1.04E-09 | 14.4413 |
| KRTAP8-1 | 2.36834 | 7.738665 | 7.720162 | 7.44E-11 | 1.04E-09 | 14.43863 |
| PLRG1 | -0.74294 | 7.450649 | -7.71822 | 7.50E-11 | 1.05E-09 | 14.4307 |

| | | | | | | |
|---|---|---|---|---|---|---|
| COX7C | -1.42772 | 9.308267 | -7.7179 | 7.51E-11 | 1.05E-09 | 14.42937 |
| NDUFB8 | -2.46134 | 9.354513 | -7.71385 | 7.64E-11 | 1.07E-09 | 14.41281 |
| GSTM5 | 2.207781 | 7.873471 | 7.713368 | 7.65E-11 | 1.07E-09 | 14.41085 |
| LOXL2 | 1.62333 | 7.426305 | 7.710189 | 7.76E-11 | 1.08E-09 | 14.39785 |
| CMTM8 | -2.4666 | 9.091272 | -7.70899 | 7.79E-11 | 1.09E-09 | 14.39296 |
| SNORA49 | -2.32613 | 9.749669 | -7.70876 | 7.80E-11 | 1.09E-09 | 14.39202 |
| CDC42SE2 | -0.72383 | 7.410357 | -7.70786 | 7.83E-11 | 1.09E-09 | 14.38832 |
| QPRT | -2.57641 | 10.16502 | -7.70778 | 7.83E-11 | 1.09E-09 | 14.38798 |
| OR6M1 | 2.616103 | 8.072138 | 7.700574 | 8.07E-11 | 1.12E-09 | 14.35853 |
| TMBIM6 | -1.53654 | 11.01767 | -7.69924 | 8.12E-11 | 1.13E-09 | 14.35308 |
| HDAC2 | -0.83628 | 7.706367 | -7.69919 | 8.12E-11 | 1.13E-09 | 14.35287 |
| MGC2712 | 0.759587 | 6.78131 | 7.698185 | 8.15E-11 | 1.13E-09 | 14.34876 |
| CACNG2 | 1.371468 | 7.036804 | 7.695762 | 8.24E-11 | 1.14E-09 | 14.33886 |
| ATG9B | 1.534754 | 7.251392 | 7.694167 | 8.29E-11 | 1.15E-09 | 14.33234 |
| ANXA8L1 | 2.027311 | 7.773996 | 7.693791 | 8.30E-11 | 1.15E-09 | 14.3308 |
| LRRC37A2 | 1.160802 | 7.899032 | 7.692913 | 8.33E-11 | 1.15E-09 | 14.32721 |
| PEX7 | -1.29779 | 7.603212 | -7.69174 | 8.37E-11 | 1.16E-09 | 14.32241 |
| GAGE4 | 3.698573 | 9.407052 | 7.690139 | 8.43E-11 | 1.17E-09 | 14.31587 |
| OR6C65 | 0.97048 | 6.841902 | 7.689038 | 8.47E-11 | 1.17E-09 | 14.31136 |
| GADD45B | -1.57815 | 8.712454 | -7.68621 | 8.57E-11 | 1.18E-09 | 14.29981 |
| DNAH8 | 0.917264 | 6.980433 | 7.684807 | 8.62E-11 | 1.19E-09 | 14.29407 |
| SMARCA2 | -1.32728 | 7.859 | -7.68356 | 8.66E-11 | 1.19E-09 | 14.28897 |
| WFDC10B | 1.011788 | 6.980466 | 7.67971 | 8.80E-11 | 1.21E-09 | 14.27323 |
| LOC72902 | 1.391884 | 9.83442 | 7.67906 | 8.83E-11 | 1.21E-09 | 14.27057 |
| AFAP1L2 | 0.917169 | 7.10538 | 7.678375 | 8.85E-11 | 1.22E-09 | 14.26777 |
| TLN1 | -1.59535 | 8.032702 | -7.67688 | 8.91E-11 | 1.22E-09 | 14.26165 |
| OGG1 | -1.13757 | 8.048378 | -7.67673 | 8.91E-11 | 1.22E-09 | 14.26106 |
| NRBP2 | -1.72733 | 8.549064 | -7.67544 | 8.96E-11 | 1.23E-09 | 14.25579 |
| RIBC2 | 1.4701 | 7.299891 | 7.674514 | 9.00E-11 | 1.23E-09 | 14.25199 |
| SNTB2 | -0.9675 | 7.526246 | -7.67445 | 9.00E-11 | 1.23E-09 | 14.25173 |
| TACC2 | -0.77087 | 7.570893 | -7.67443 | 9.00E-11 | 1.23E-09 | 14.25164 |
| PABPC1L2 | 1.600581 | 7.279959 | 7.673567 | 9.03E-11 | 1.23E-09 | 14.24811 |
| ACTA2 | -2.14307 | 9.199572 | -7.67264 | 9.07E-11 | 1.24E-09 | 14.24431 |
| OR13F1 | 1.096922 | 6.950011 | 7.669989 | 9.17E-11 | 1.25E-09 | 14.23349 |
| AMELX | 1.945973 | 7.485606 | 7.668111 | 9.24E-11 | 1.26E-09 | 14.22581 |
| ALX1 | 1.285664 | 7.267937 | 7.667481 | 9.26E-11 | 1.26E-09 | 14.22323 |
| MIR548I1 | 2.111496 | 7.913119 | 7.666646 | 9.30E-11 | 1.27E-09 | 14.21982 |
| PTEN | -1.12653 | 8.09843 | -7.66576 | 9.33E-11 | 1.27E-09 | 14.2162 |
| SNORA16 | -1.72142 | 10.45083 | -7.66524 | 9.35E-11 | 1.27E-09 | 14.21407 |
| SLC25A39 | -2.26474 | 10.39435 | -7.6636 | 9.41E-11 | 1.28E-09 | 14.20736 |
| UQCRQ | -0.93052 | 7.528716 | -7.66325 | 9.43E-11 | 1.28E-09 | 14.20595 |
| FAM26C | 1.223132 | 6.925213 | 7.661701 | 9.49E-11 | 1.29E-09 | 14.19961 |
| CDC123 | -2.24625 | 8.724853 | -7.65561 | 9.73E-11 | 1.32E-09 | 14.17469 |
| BMS1P5 | 1.732845 | 8.825016 | 7.654951 | 9.76E-11 | 1.32E-09 | 14.17202 |
| C20orf27 | -1.22464 | 7.814264 | -7.65469 | 9.77E-11 | 1.32E-09 | 14.17094 |
| CCDC62 | 1.492422 | 7.249545 | 7.654434 | 9.78E-11 | 1.32E-09 | 14.1699 |
| HECTD1 | -0.75004 | 7.459594 | -7.6516 | 9.90E-11 | 1.34E-09 | 14.1583 |
| ERN2 | 0.996485 | 7.228422 | 7.650917 | 9.92E-11 | 1.34E-09 | 14.15553 |
| GNS | -1.82446 | 8.629393 | -7.6493 | 9.99E-11 | 1.35E-09 | 14.14894 |
| RPRM | 0.76063 | 6.83372 | 7.646961 | 1.01E-10 | 1.36E-09 | 14.13936 |
| ZNF428 | -1.32421 | 8.318509 | -7.64601 | 1.01E-10 | 1.36E-09 | 14.13548 |
| SHROOM4 | 1.027497 | 7.062131 | 7.644352 | 1.02E-10 | 1.37E-09 | 14.1287 |
| NEDD8 | -1.91298 | 9.78402 | -7.64394 | 1.02E-10 | 1.37E-09 | 14.12701 |
| ZNF645 | 1.150017 | 6.938606 | 7.643637 | 1.02E-10 | 1.37E-09 | 14.12577 |
| MIF | -1.30933 | 7.619026 | -7.64294 | 1.03E-10 | 1.38E-09 | 14.12293 |
| OAZ1 | -1.98908 | 10.42175 | -7.64109 | 1.03E-10 | 1.39E-09 | 14.11535 |
| LRTOMT | 1.025994 | 7.790634 | 7.638713 | 1.04E-10 | 1.40E-09 | 14.10565 |
| CD84 | 1.317016 | 8.344548 | 7.637361 | 1.05E-10 | 1.41E-09 | 14.10012 |

| | | | | | | |
|---|---|---|---|---|---|---|
| ATP9B | 1.438392 | 9.714055 | 7.637072 | 1.05E-10 | 1.41E-09 | 14.09894 |
| SNORA26 | -1.59642 | 8.205736 | -7.63541 | 1.06E-10 | 1.42E-09 | 14.09216 |
| FFAR3 | 1.660717 | 7.26932 | 7.634072 | 1.06E-10 | 1.42E-09 | 14.08668 |
| ZNF821 | -0.8871 | 7.682596 | -7.6323 | 1.07E-10 | 1.43E-09 | 14.07945 |
| CALN1 | 0.79399 | 7.039043 | 7.631228 | 1.08E-10 | 1.44E-09 | 14.07506 |
| OR2T11 | 0.903107 | 7.113819 | 7.623498 | 1.11E-10 | 1.48E-09 | 14.04347 |
| GAPDH | -1.19369 | 9.803254 | -7.62059 | 1.13E-10 | 1.50E-09 | 14.03158 |
| HAND1 | 2.170278 | 7.622227 | 7.619317 | 1.13E-10 | 1.50E-09 | 14.02638 |
| SUV39H1 | -0.74921 | 7.140418 | -7.61929 | 1.13E-10 | 1.50E-09 | 14.02628 |
| DEFB113 | 1.070378 | 6.93635 | 7.619228 | 1.13E-10 | 1.50E-09 | 14.02602 |
| TNFRSF8 | 1.380428 | 7.209458 | 7.617251 | 1.14E-10 | 1.51E-09 | 14.01794 |
| APOB | -2.69788 | 11.42399 | -7.61682 | 1.14E-10 | 1.51E-09 | 14.01619 |
| HDAC1 | -0.82342 | 7.812904 | -7.61619 | 1.15E-10 | 1.52E-09 | 14.01359 |
| ZNF286A | 1.246594 | 7.506294 | 7.613966 | 1.16E-10 | 1.53E-09 | 14.00451 |
| OR6C1 | 1.050246 | 6.864028 | 7.613611 | 1.16E-10 | 1.53E-09 | 14.00306 |
| NCKAP1 | -1.09693 | 7.780261 | -7.61249 | 1.16E-10 | 1.54E-09 | 13.99847 |
| SPSB3 | -1.39401 | 8.362232 | -7.61222 | 1.17E-10 | 1.54E-09 | 13.99738 |
| BAAT | -1.55645 | 8.178766 | -7.61053 | 1.17E-10 | 1.55E-09 | 13.99047 |
| GEMIN7 | 0.930075 | 7.177156 | 7.609996 | 1.18E-10 | 1.55E-09 | 13.98829 |
| RPS14 | -1.22036 | 10.08783 | -7.60994 | 1.18E-10 | 1.55E-09 | 13.98805 |
| SPRR2E | 2.043771 | 7.263918 | 7.608066 | 1.19E-10 | 1.56E-09 | 13.98041 |
| DNTTIP2 | -0.70707 | 7.137507 | -7.60727 | 1.19E-10 | 1.57E-09 | 13.97714 |
| RARS | -0.90059 | 7.730103 | -7.60689 | 1.19E-10 | 1.57E-09 | 13.97559 |
| TRIM8 | -1.38202 | 8.570578 | -7.60651 | 1.19E-10 | 1.57E-09 | 13.97405 |
| HRH4 | 1.124554 | 7.207599 | 7.605901 | 1.20E-10 | 1.57E-09 | 13.97156 |
| TBX19 | 1.302433 | 7.974487 | 7.604901 | 1.20E-10 | 1.58E-09 | 13.96747 |
| SPG3A | 1.488352 | 7.776368 | 7.59811 | 1.24E-10 | 1.62E-09 | 13.93972 |
| OR2AK2 | 0.998567 | 6.944469 | 7.596043 | 1.25E-10 | 1.63E-09 | 13.93128 |
| RPL15 | -0.87182 | 7.537043 | -7.59467 | 1.25E-10 | 1.64E-09 | 13.92567 |
| SLC9A3R2 | -1.19754 | 8.004577 | -7.59344 | 1.26E-10 | 1.65E-09 | 13.92065 |
| SNORA8 | -1.81548 | 8.394476 | -7.59296 | 1.26E-10 | 1.65E-09 | 13.91867 |
| PRKAR1A | -1.03626 | 7.696337 | -7.59169 | 1.27E-10 | 1.65E-09 | 13.9135 |
| SNORA77 | -1.81504 | 9.121415 | -7.59002 | 1.28E-10 | 1.67E-09 | 13.90667 |
| OR5D14 | 1.189017 | 7.001333 | 7.589622 | 1.28E-10 | 1.67E-09 | 13.90505 |
| GPR12 | 2.042661 | 7.497602 | 7.589301 | 1.28E-10 | 1.67E-09 | 13.90374 |
| TMEM225 | 1.537511 | 7.182449 | 7.589142 | 1.28E-10 | 1.67E-09 | 13.90309 |
| MYH10 | -1.15813 | 8.046717 | -7.58905 | 1.28E-10 | 1.67E-09 | 13.9027 |
| RPS5 | -0.80997 | 7.142537 | -7.58781 | 1.29E-10 | 1.68E-09 | 13.89765 |
| OR5L2 | 1.065008 | 6.924173 | 7.587359 | 1.29E-10 | 1.68E-09 | 13.8958 |
| FAM115A | 1.143675 | 8.523771 | 7.58714 | 1.29E-10 | 1.68E-09 | 13.8949 |
| OSBP | -1.10069 | 8.207056 | -7.58613 | 1.30E-10 | 1.68E-09 | 13.89078 |
| CBR1 | -1.41627 | 9.256324 | -7.58594 | 1.30E-10 | 1.68E-09 | 13.89 |
| PRG1 | 1.150929 | 6.988908 | 7.584732 | 1.31E-10 | 1.69E-09 | 13.88507 |
| HAGH | -1.0424 | 7.663444 | -7.58406 | 1.31E-10 | 1.69E-09 | 13.88232 |
| HDGF | -1.0472 | 7.728709 | -7.58332 | 1.31E-10 | 1.70E-09 | 13.87931 |
| GAP43 | 2.359142 | 8.11952 | 7.582471 | 1.32E-10 | 1.70E-09 | 13.87583 |
| LOC12653 | 1.016768 | 6.830119 | 7.582138 | 1.32E-10 | 1.70E-09 | 13.87447 |
| SLITRK1 | 0.842627 | 6.842754 | 7.580632 | 1.33E-10 | 1.71E-09 | 13.86832 |
| CAT | -1.25331 | 7.931435 | -7.57657 | 1.35E-10 | 1.74E-09 | 13.85172 |
| OR2F2 | 1.291701 | 7.00678 | 7.574653 | 1.36E-10 | 1.75E-09 | 13.8439 |
| KRT27 | 1.755858 | 7.682953 | 7.57167 | 1.38E-10 | 1.77E-09 | 13.83171 |
| PCMT1 | -2.28705 | 9.106982 | -7.57149 | 1.38E-10 | 1.77E-09 | 13.83099 |
| TRIM34 | 0.953425 | 7.037668 | 7.571399 | 1.38E-10 | 1.77E-09 | 13.8306 |
| NOS1 | 1.785284 | 7.578004 | 7.570181 | 1.39E-10 | 1.78E-09 | 13.82563 |
| ZDHHC1 | -1.28767 | 7.870458 | -7.56999 | 1.39E-10 | 1.78E-09 | 13.82484 |
| HRNBP3 | 2.382602 | 7.621237 | 7.568553 | 1.40E-10 | 1.79E-09 | 13.81898 |
| POTE2 | 1.52352 | 7.492526 | 7.566798 | 1.41E-10 | 1.80E-09 | 13.81181 |
| MBTD1 | 1.004427 | 11.51906 | 7.566716 | 1.41E-10 | 1.80E-09 | 13.81147 |

| | | | | | | |
|---|---|---|---|---|---|---|
| FXYD3 | 0.759181 | 6.90939 | 7.566585 | 1.41E-10 | 1.80E-09 | 13.81094 |
| AMPD1 | 0.732042 | 6.778571 | 7.566167 | 1.41E-10 | 1.80E-09 | 13.80923 |
| BTNL3 | 1.247506 | 7.256849 | 7.565622 | 1.42E-10 | 1.81E-09 | 13.80701 |
| PCDHA5 | 1.14222 | 7.188865 | 7.565559 | 1.42E-10 | 1.81E-09 | 13.80675 |
| GAGE12J | 3.301241 | 9.082686 | 7.565221 | 1.42E-10 | 1.81E-09 | 13.80537 |
| LOC65354 | 2.5099 | 8.087581 | 7.564471 | 1.42E-10 | 1.81E-09 | 13.8023 |
| RPLP2 | -2.55426 | 10.35061 | -7.56408 | 1.42E-10 | 1.81E-09 | 13.8007 |
| NCKIPSD | -0.86547 | 7.498365 | -7.56333 | 1.43E-10 | 1.82E-09 | 13.79765 |
| CD96 | 1.357581 | 9.704875 | 7.563221 | 1.43E-10 | 1.82E-09 | 13.7972 |
| FBXW12 | 0.740962 | 6.834476 | 7.561177 | 1.44E-10 | 1.83E-09 | 13.78885 |
| PTPLA | 1.666138 | 7.788334 | 7.560095 | 1.45E-10 | 1.84E-09 | 13.78443 |
| KHDRBS1 | -0.71476 | 7.373674 | -7.55988 | 1.45E-10 | 1.84E-09 | 13.78357 |
| IL10 | 1.580464 | 8.247609 | 7.55982 | 1.45E-10 | 1.84E-09 | 13.78331 |
| GNG12 | -1.08929 | 8.168884 | -7.55943 | 1.45E-10 | 1.84E-09 | 13.7817 |
| FAM120A | -1.4616 | 8.25627 | -7.55764 | 1.46E-10 | 1.85E-09 | 13.7744 |
| RAD1 | 1.91259 | 8.021449 | 7.557587 | 1.46E-10 | 1.85E-09 | 13.77418 |
| KRTAP11- | 1.430751 | 7.227162 | 7.556185 | 1.47E-10 | 1.86E-09 | 13.76846 |
| DUX4 | 2.965026 | 8.651425 | 7.553276 | 1.49E-10 | 1.88E-09 | 13.75658 |
| TCF25 | -1.00988 | 7.909808 | -7.55295 | 1.49E-10 | 1.88E-09 | 13.75523 |
| OR4F3 | 1.227928 | 7.374021 | 7.551773 | 1.50E-10 | 1.89E-09 | 13.75044 |
| ENKUR | 1.736914 | 7.584422 | 7.550543 | 1.51E-10 | 1.90E-09 | 13.74541 |
| C2orf25 | -0.80089 | 7.44603 | -7.54859 | 1.52E-10 | 1.91E-09 | 13.73745 |
| HIST1H4E | -1.20848 | 8.065229 | -7.54577 | 1.54E-10 | 1.93E-09 | 13.72593 |
| FCGRT | -1.31457 | 8.94594 | -7.54576 | 1.54E-10 | 1.93E-09 | 13.72587 |
| C1orf92 | 1.132987 | 7.102291 | 7.545287 | 1.54E-10 | 1.94E-09 | 13.72395 |
| GIP | 0.951186 | 6.926358 | 7.544279 | 1.55E-10 | 1.94E-09 | 13.71983 |
| BTBD2 | -0.89881 | 7.673151 | -7.54427 | 1.55E-10 | 1.94E-09 | 13.7198 |
| HES1 | -0.84526 | 7.359764 | -7.5391 | 1.58E-10 | 1.98E-09 | 13.69868 |
| KCNQ2 | 1.775175 | 7.494758 | 7.537825 | 1.59E-10 | 1.99E-09 | 13.69348 |
| ABCC2 | -1.06144 | 7.571826 | -7.53556 | 1.60E-10 | 2.00E-09 | 13.68422 |
| OR1K1 | 1.585988 | 7.365716 | 7.535247 | 1.61E-10 | 2.00E-09 | 13.68295 |
| AKR1A1 | -1.19474 | 7.831655 | -7.53516 | 1.61E-10 | 2.00E-09 | 13.68261 |
| ITM2B | -0.72954 | 7.168809 | -7.533 | 1.62E-10 | 2.02E-09 | 13.67376 |
| ATP6V1A | -0.7158 | 7.201648 | -7.53287 | 1.62E-10 | 2.02E-09 | 13.67325 |
| AGT | -2.10573 | 9.082552 | -7.53265 | 1.62E-10 | 2.02E-09 | 13.67236 |
| RNPEP | -1.06124 | 7.601499 | -7.53226 | 1.63E-10 | 2.02E-09 | 13.67075 |
| ANGPTL4 | -1.75246 | 8.88402 | -7.53224 | 1.63E-10 | 2.02E-09 | 13.67069 |
| NOL7 | -1.83116 | 9.686422 | -7.53202 | 1.63E-10 | 2.02E-09 | 13.66977 |
| FBL | -2.15048 | 8.54132 | -7.53036 | 1.64E-10 | 2.03E-09 | 13.663 |
| CCDC155 | 1.364344 | 7.269503 | 7.529505 | 1.64E-10 | 2.04E-09 | 13.6595 |
| RHOC | -1.16225 | 8.263768 | -7.52873 | 1.65E-10 | 2.04E-09 | 13.65635 |
| SUCLG1 | -2.24909 | 10.08869 | -7.52698 | 1.66E-10 | 2.06E-09 | 13.64918 |
| ITGA5 | -1.81598 | 8.694846 | -7.52417 | 1.68E-10 | 2.08E-09 | 13.6377 |
| FIGN | 1.986111 | 8.668393 | 7.522703 | 1.69E-10 | 2.09E-09 | 13.63173 |
| TRIM58 | 0.720612 | 6.933967 | 7.521713 | 1.70E-10 | 2.10E-09 | 13.62769 |
| MAP4K2 | -1.63139 | 8.633478 | -7.52138 | 1.70E-10 | 2.10E-09 | 13.62633 |
| KNG1 | -2.13316 | 10.33818 | -7.52052 | 1.71E-10 | 2.11E-09 | 13.6228 |
| ACRBP | 0.836204 | 7.011999 | 7.519158 | 1.72E-10 | 2.12E-09 | 13.61726 |
| GHSR | 2.021863 | 7.756697 | 7.515084 | 1.75E-10 | 2.15E-09 | 13.60062 |
| LOC64231 | 1.093113 | 7.001477 | 7.513746 | 1.76E-10 | 2.16E-09 | 13.59516 |
| CA1 | 1.004991 | 7.03755 | 7.51365 | 1.76E-10 | 2.16E-09 | 13.59477 |
| CEBPZ | -1.01619 | 8.361441 | -7.51226 | 1.77E-10 | 2.17E-09 | 13.58911 |
| PLAG1 | 0.966151 | 7.337504 | 7.509508 | 1.79E-10 | 2.19E-09 | 13.57786 |
| PLA2G12E | -1.51308 | 8.334925 | -7.50934 | 1.79E-10 | 2.19E-09 | 13.57718 |
| TP53TG1 | -1.30381 | 7.879482 | -7.50899 | 1.79E-10 | 2.20E-09 | 13.57575 |
| SV2C | 0.836554 | 6.74512 | 7.507344 | 1.80E-10 | 2.21E-09 | 13.56902 |
| STOM | -1.09028 | 7.903734 | -7.50609 | 1.81E-10 | 2.22E-09 | 13.56392 |
| BARX2 | 0.868558 | 6.784346 | 7.505504 | 1.82E-10 | 2.22E-09 | 13.56151 |

| | | | | | | |
|---|---|---|---|---|---|---|
| LOC38772 | 1.000137 | 6.931414 | 7.504973 | 1.82E-10 | 2.23E-09 | 13.55934 |
| UBQLNL | 1.958023 | 7.935401 | 7.504376 | 1.83E-10 | 2.23E-09 | 13.5569 |
| DUPD1 | 0.883757 | 6.911837 | 7.503211 | 1.83E-10 | 2.24E-09 | 13.55215 |
| TMEM141 | -1.61486 | 8.521345 | -7.50181 | 1.84E-10 | 2.25E-09 | 13.54643 |
| ALDOB | -2.28603 | 10.62382 | -7.49882 | 1.87E-10 | 2.28E-09 | 13.53423 |
| GAGE12E | 1.354226 | 7.615685 | 7.498499 | 1.87E-10 | 2.28E-09 | 13.53292 |
| LSM11 | 1.650049 | 9.886163 | 7.498392 | 1.87E-10 | 2.28E-09 | 13.53248 |
| IL11 | 1.033445 | 7.084605 | 7.497632 | 1.88E-10 | 2.29E-09 | 13.52938 |
| VCX2 | 1.294515 | 7.210494 | 7.495513 | 1.89E-10 | 2.31E-09 | 13.52072 |
| LOC15038 | 1.292122 | 7.435755 | 7.493155 | 1.91E-10 | 2.32E-09 | 13.5111 |
| LRCH4 | -1.29614 | 8.485799 | -7.49272 | 1.92E-10 | 2.33E-09 | 13.50932 |
| FLJ42957 | 2.519284 | 8.522993 | 7.491878 | 1.92E-10 | 2.33E-09 | 13.50589 |
| CCDC68 | 0.863452 | 7.879301 | 7.491718 | 1.92E-10 | 2.33E-09 | 13.50523 |
| OR2AE1 | 1.488369 | 7.27288 | 7.491593 | 1.93E-10 | 2.33E-09 | 13.50472 |
| A1BG | 1.054957 | 7.433151 | 7.491168 | 1.93E-10 | 2.34E-09 | 13.50299 |
| ABLIM2 | 2.105454 | 8.647703 | 7.490644 | 1.93E-10 | 2.34E-09 | 13.50085 |
| ID2 | -1.34395 | 8.280797 | -7.49038 | 1.93E-10 | 2.34E-09 | 13.49978 |
| NBPF10 | 1.022383 | 11.29166 | 7.489013 | 1.95E-10 | 2.35E-09 | 13.49419 |
| SNORA61 | -2.01876 | 11.98586 | -7.4885 | 1.95E-10 | 2.36E-09 | 13.49209 |
| HIGD2A | -1.17662 | 7.89429 | -7.48571 | 1.97E-10 | 2.38E-09 | 13.48073 |
| FNDC3B | -0.90323 | 8.115437 | -7.48561 | 1.97E-10 | 2.38E-09 | 13.48031 |
| CD1A | 2.103187 | 7.773363 | 7.485274 | 1.98E-10 | 2.38E-09 | 13.47893 |
| DBF4B | 1.238676 | 7.269418 | 7.484525 | 1.98E-10 | 2.39E-09 | 13.47587 |
| FAM90A1 | 1.336184 | 7.104318 | 7.480936 | 2.01E-10 | 2.42E-09 | 13.46123 |
| PRDX3 | -1.14377 | 8.158665 | -7.47907 | 2.03E-10 | 2.44E-09 | 13.45359 |
| KLF6 | -1.32244 | 7.896833 | -7.4781 | 2.04E-10 | 2.45E-09 | 13.44967 |
| PLS3 | -1.63017 | 8.173493 | -7.47616 | 2.05E-10 | 2.46E-09 | 13.44175 |
| OR5M8 | 0.97037 | 6.971906 | 7.474587 | 2.07E-10 | 2.48E-09 | 13.43532 |
| MRPS6 | -1.65648 | 8.362209 | -7.47139 | 2.09E-10 | 2.51E-09 | 13.42227 |
| COMT | -1.00787 | 7.809572 | -7.47119 | 2.10E-10 | 2.51E-09 | 13.42147 |
| RPS17 | -1.33828 | 8.236047 | -7.47119 | 2.10E-10 | 2.51E-09 | 13.42145 |
| OR10AD1 | 1.145378 | 7.134535 | 7.469438 | 2.11E-10 | 2.53E-09 | 13.4143 |
| FGB | -1.79959 | 8.623492 | -7.46745 | 2.13E-10 | 2.54E-09 | 13.4062 |
| TXLNA | -0.88577 | 7.183694 | -7.46541 | 2.15E-10 | 2.56E-09 | 13.39786 |
| C8orf31 | 1.624197 | 7.589829 | 7.462331 | 2.17E-10 | 2.59E-09 | 13.3853 |
| OR4F21 | 1.608126 | 7.702133 | 7.462164 | 2.18E-10 | 2.59E-09 | 13.38462 |
| LCAT | -2.06902 | 9.019923 | -7.46165 | 2.18E-10 | 2.60E-09 | 13.38252 |
| TNFRSF14 | -2.11032 | 10.18158 | -7.45967 | 2.20E-10 | 2.62E-09 | 13.37443 |
| TSC22D1 | -0.88381 | 7.433407 | -7.45964 | 2.20E-10 | 2.62E-09 | 13.37433 |
| CST4 | 0.773151 | 6.831471 | 7.456415 | 2.23E-10 | 2.65E-09 | 13.36117 |
| DEFA5 | 1.447899 | 7.340988 | 7.455916 | 2.23E-10 | 2.65E-09 | 13.35913 |
| LSM7 | -1.20701 | 7.740822 | -7.455 | 2.24E-10 | 2.66E-09 | 13.35539 |
| C3orf24 | 0.784224 | 6.879276 | 7.453856 | 2.25E-10 | 2.67E-09 | 13.35072 |
| NUCKS1 | -1.67378 | 8.734814 | -7.4534 | 2.26E-10 | 2.67E-09 | 13.34887 |
| CFB | -1.95178 | 8.501384 | -7.45043 | 2.28E-10 | 2.71E-09 | 13.33675 |
| C15orf63 | 0.813556 | 7.065445 | 7.449902 | 2.29E-10 | 2.71E-09 | 13.33459 |
| IDH1 | -0.75279 | 7.366439 | -7.44442 | 2.34E-10 | 2.77E-09 | 13.31221 |
| SUMF2 | -1.24914 | 9.943913 | -7.44076 | 2.38E-10 | 2.81E-09 | 13.29728 |
| PTS | -1.05502 | 7.828838 | -7.44047 | 2.38E-10 | 2.81E-09 | 13.29611 |
| VENTXP1 | 1.437766 | 7.304765 | 7.437856 | 2.41E-10 | 2.84E-09 | 13.28545 |
| CLEC4E | 0.757491 | 6.913277 | 7.436866 | 2.42E-10 | 2.85E-09 | 13.28141 |
| ROCK1 | -1.02669 | 7.925625 | -7.43652 | 2.42E-10 | 2.85E-09 | 13.28 |
| TIAM2 | 1.432533 | 7.621541 | 7.43474 | 2.44E-10 | 2.87E-09 | 13.27273 |
| CARS | -1.43175 | 8.039686 | -7.4342 | 2.44E-10 | 2.87E-09 | 13.27052 |
| SH3BGRL3 | -0.97381 | 8.493979 | -7.43411 | 2.44E-10 | 2.87E-09 | 13.27017 |
| CD163 | -0.98348 | 7.370379 | -7.43399 | 2.45E-10 | 2.87E-09 | 13.26966 |
| TMEM91 | -0.79675 | 7.241825 | -7.43324 | 2.45E-10 | 2.88E-09 | 13.2666 |
| SLC24A4 | 0.728637 | 6.94534 | 7.426744 | 2.52E-10 | 2.95E-09 | 13.24012 |

| | | | | | | |
|---|---|---|---|---|---|---|
| COPB1 | -1.01236 | 9.037306 | -7.42557 | 2.53E-10 | 2.96E-09 | 13.23535 |
| CSPG4 | -0.79309 | 7.276833 | -7.42507 | 2.54E-10 | 2.97E-09 | 13.23327 |
| TUBA1C | -1.18023 | 8.770317 | -7.4219 | 2.57E-10 | 3.00E-09 | 13.22035 |
| GPR3 | 1.220331 | 7.298749 | 7.421193 | 2.58E-10 | 3.01E-09 | 13.21748 |
| FAT1 | -0.80239 | 7.488259 | -7.42116 | 2.58E-10 | 3.01E-09 | 13.21734 |
| ZSWIM3 | 1.002503 | 7.460183 | 7.420135 | 2.59E-10 | 3.02E-09 | 13.21316 |
| PSAP | -2.28712 | 9.096778 | -7.41948 | 2.60E-10 | 3.03E-09 | 13.2105 |
| SURF1 | -0.92423 | 7.316855 | -7.41902 | 2.60E-10 | 3.03E-09 | 13.20863 |
| FAIM2 | 1.80546 | 7.601253 | 7.417 | 2.62E-10 | 3.05E-09 | 13.20038 |
| GATM | -1.83782 | 8.9126 | -7.41537 | 2.64E-10 | 3.07E-09 | 13.19375 |
| FSD2 | 0.885578 | 6.854138 | 7.415099 | 2.65E-10 | 3.07E-09 | 13.19262 |
| IRF2BP2 | -1.27835 | 8.945764 | -7.41469 | 2.65E-10 | 3.08E-09 | 13.19097 |
| SNORA59 | -0.7652 | 7.32443 | -7.41251 | 2.67E-10 | 3.10E-09 | 13.18208 |
| NOMO3 | -0.87654 | 7.188837 | -7.41214 | 2.68E-10 | 3.10E-09 | 13.18054 |
| SRM | -0.9058 | 7.812304 | -7.41085 | 2.69E-10 | 3.12E-09 | 13.17531 |
| SNORD95 | -2.47581 | 9.994723 | -7.40968 | 2.71E-10 | 3.13E-09 | 13.17051 |
| VASN | -1.33618 | 8.340403 | -7.40794 | 2.72E-10 | 3.15E-09 | 13.16343 |
| OR11H6 | 1.052902 | 7.026205 | 7.4064 | 2.74E-10 | 3.16E-09 | 13.15715 |
| HLA-DRA | -1.46848 | 8.731049 | -7.40633 | 2.74E-10 | 3.16E-09 | 13.15686 |
| B3GALT5 | 0.987826 | 6.997457 | 7.406307 | 2.74E-10 | 3.16E-09 | 13.15677 |
| OTOP1 | 1.286965 | 7.088521 | 7.405409 | 2.75E-10 | 3.18E-09 | 13.1531 |
| KRTAP13- | 1.418485 | 7.266771 | 7.404309 | 2.77E-10 | 3.19E-09 | 13.14862 |
| GADL1 | 0.728954 | 6.748017 | 7.402922 | 2.78E-10 | 3.20E-09 | 13.14296 |
| RNF167 | -0.76972 | 7.285391 | -7.40274 | 2.78E-10 | 3.20E-09 | 13.14221 |
| DYNC1I2 | -0.85247 | 7.242924 | -7.40253 | 2.79E-10 | 3.20E-09 | 13.14138 |
| LOC10013 | 1.474747 | 10.94614 | 7.402296 | 2.79E-10 | 3.20E-09 | 13.14041 |
| SCGBL | 1.173247 | 7.121452 | 7.402293 | 2.79E-10 | 3.20E-09 | 13.1404 |
| DCI | -0.85103 | 7.486537 | -7.40198 | 2.79E-10 | 3.20E-09 | 13.13911 |
| FAM98A | -1.67324 | 8.875132 | -7.40178 | 2.80E-10 | 3.21E-09 | 13.13829 |
| HSPG2 | -1.42976 | 7.714957 | -7.39847 | 2.83E-10 | 3.25E-09 | 13.12482 |
| GNG5 | -1.07329 | 7.542491 | -7.39828 | 2.84E-10 | 3.25E-09 | 13.12402 |
| ACTR2 | -1.15437 | 7.906769 | -7.39772 | 2.84E-10 | 3.25E-09 | 13.12175 |
| AGBL4 | 1.435342 | 7.069404 | 7.397688 | 2.84E-10 | 3.25E-09 | 13.12162 |
| ADCY2 | 1.232658 | 7.206765 | 7.397495 | 2.85E-10 | 3.25E-09 | 13.12083 |
| DCDC5 | 1.286318 | 7.416156 | 7.394314 | 2.88E-10 | 3.29E-09 | 13.10786 |
| EZR | -2.20764 | 8.692673 | -7.39423 | 2.88E-10 | 3.29E-09 | 13.10754 |
| GDI2 | -0.90178 | 7.439114 | -7.39374 | 2.89E-10 | 3.30E-09 | 13.10552 |
| C17orf50 | 2.356152 | 7.826142 | 7.392351 | 2.91E-10 | 3.31E-09 | 13.09986 |
| HCN4 | 2.265892 | 7.857322 | 7.39227 | 2.91E-10 | 3.31E-09 | 13.09953 |
| MGC4236 | 1.531756 | 8.116214 | 7.391866 | 2.91E-10 | 3.32E-09 | 13.09788 |
| SRPK3 | 1.411187 | 7.027399 | 7.391461 | 2.92E-10 | 3.32E-09 | 13.09623 |
| ZSWIM2 | 0.842903 | 6.834925 | 7.389798 | 2.94E-10 | 3.34E-09 | 13.08945 |
| CARTPT | 0.839428 | 7.021293 | 7.389315 | 2.94E-10 | 3.35E-09 | 13.08748 |
| ULK1 | -1.08073 | 9.485693 | -7.38816 | 2.96E-10 | 3.36E-09 | 13.08277 |
| PSORS1C1 | 1.946192 | 7.979839 | 7.386609 | 2.98E-10 | 3.38E-09 | 13.07645 |
| PRAMEF6 | 1.60299 | 7.307948 | 7.386328 | 2.98E-10 | 3.38E-09 | 13.0753 |
| FZD3 | 1.848903 | 8.346435 | 7.385448 | 2.99E-10 | 3.39E-09 | 13.07172 |
| ADAM21P | 0.954864 | 6.861194 | 7.384228 | 3.01E-10 | 3.41E-09 | 13.06674 |
| FTHL11 | 0.722831 | 6.861546 | 7.383637 | 3.01E-10 | 3.42E-09 | 13.06433 |
| ZFP36L1 | -1.12352 | 7.847352 | -7.38343 | 3.02E-10 | 3.42E-09 | 13.06349 |
| KCNA10 | 0.758619 | 6.798112 | 7.383413 | 3.02E-10 | 3.42E-09 | 13.06342 |
| IL2RA | 1.330235 | 7.620138 | 7.38318 | 3.02E-10 | 3.42E-09 | 13.06247 |
| NKX2-1 | 2.564186 | 7.945538 | 7.382547 | 3.03E-10 | 3.42E-09 | 13.05989 |
| C11orf45 | 2.005877 | 7.670902 | 7.380291 | 3.06E-10 | 3.45E-09 | 13.05069 |
| DNAJA1 | -1.34764 | 8.035478 | -7.38011 | 3.06E-10 | 3.45E-09 | 13.04994 |
| SRP54 | -0.97564 | 7.525038 | -7.37964 | 3.06E-10 | 3.46E-09 | 13.04804 |
| C1orf151 | 1.027089 | 7.385194 | 7.379287 | 3.07E-10 | 3.46E-09 | 13.0466 |
| EMR4P | 1.224185 | 7.406905 | 7.378926 | 3.07E-10 | 3.46E-09 | 13.04513 |

| | | | | | | |
|---|---|---|---|---|---|---|
| C14orf166 | 0.873688 | 6.794028 | 7.378205 | 3.08E-10 | 3.47E-09 | 13.04219 |
| OR52N4 | 1.396771 | 7.137489 | 7.377408 | 3.09E-10 | 3.48E-09 | 13.03894 |
| NAG18 | 1.537878 | 10.94513 | 7.375432 | 3.12E-10 | 3.51E-09 | 13.03088 |
| KRTAP5-8 | 1.51881 | 7.254393 | 7.375088 | 3.12E-10 | 3.51E-09 | 13.02948 |
| CIR1 | -0.72491 | 7.574684 | -7.37444 | 3.13E-10 | 3.52E-09 | 13.02686 |
| MIR200A | 1.135815 | 7.078875 | 7.372658 | 3.15E-10 | 3.54E-09 | 13.01958 |
| CYP2S1 | 1.471727 | 7.956691 | 7.368938 | 3.20E-10 | 3.60E-09 | 13.00441 |
| LOC40046 | 1.207029 | 7.508092 | 7.368127 | 3.21E-10 | 3.61E-09 | 13.00111 |
| TAS2R9 | 0.975523 | 6.974972 | 7.367045 | 3.23E-10 | 3.62E-09 | 12.9967 |
| PNMAL2 | 1.694603 | 7.524447 | 7.366569 | 3.24E-10 | 3.62E-09 | 12.99476 |
| SLC39A14 | -1.63165 | 9.04781 | -7.36341 | 3.28E-10 | 3.67E-09 | 12.98188 |
| OPLAH | -1.78554 | 10.07203 | -7.36315 | 3.28E-10 | 3.67E-09 | 12.9808 |
| TOB1 | -1.55193 | 8.869553 | -7.36271 | 3.29E-10 | 3.67E-09 | 12.97902 |
| SNORD56 | -2.07583 | 9.971776 | -7.36025 | 3.32E-10 | 3.71E-09 | 12.969 |
| MTCP1 | -1.18326 | 8.000425 | -7.35997 | 3.33E-10 | 3.71E-09 | 12.96787 |
| KBTBD5 | 1.120056 | 7.024525 | 7.358496 | 3.35E-10 | 3.73E-09 | 12.96185 |
| LOC34052 | 1.589737 | 7.238369 | 7.354643 | 3.40E-10 | 3.79E-09 | 12.94615 |
| C2 | -1.53737 | 8.859923 | -7.35435 | 3.40E-10 | 3.79E-09 | 12.94495 |
| OR7C1 | 1.290795 | 7.041779 | 7.353695 | 3.41E-10 | 3.79E-09 | 12.94229 |
| PRDX1 | -1.59554 | 9.078414 | -7.35364 | 3.41E-10 | 3.79E-09 | 12.94207 |
| FRG2C | 2.189364 | 7.725159 | 7.353215 | 3.42E-10 | 3.80E-09 | 12.94033 |
| SCAF1 | -0.94316 | 7.652701 | -7.35219 | 3.43E-10 | 3.81E-09 | 12.93617 |
| C1orf200 | 0.831532 | 6.942368 | 7.351347 | 3.45E-10 | 3.82E-09 | 12.93272 |
| PSMC6 | -0.90212 | 7.837065 | -7.35066 | 3.46E-10 | 3.83E-09 | 12.92993 |
| LIMK1 | 1.345674 | 7.883341 | 7.350235 | 3.46E-10 | 3.84E-09 | 12.92819 |
| CARHSP1 | -2.17808 | 9.610171 | -7.34794 | 3.50E-10 | 3.87E-09 | 12.91884 |
| METTL13 | -1.2699 | 8.588243 | -7.34686 | 3.51E-10 | 3.88E-09 | 12.91445 |
| WEE2 | 1.168304 | 6.976024 | 7.34591 | 3.53E-10 | 3.90E-09 | 12.91056 |
| ACOX2 | -1.64991 | 7.998112 | -7.34493 | 3.54E-10 | 3.91E-09 | 12.90658 |
| CTSB | -1.25451 | 9.650895 | -7.34364 | 3.56E-10 | 3.93E-09 | 12.90132 |
| PRCP | -1.36285 | 8.546874 | -7.34083 | 3.60E-10 | 3.97E-09 | 12.88987 |
| OR13G1 | 0.764175 | 6.802567 | 7.338742 | 3.63E-10 | 4.00E-09 | 12.88136 |
| SLC25A6 | -1.45443 | 8.422919 | -7.33447 | 3.70E-10 | 4.07E-09 | 12.86395 |
| ALDH6A1 | -1.07134 | 8.816468 | -7.33359 | 3.71E-10 | 4.08E-09 | 12.86037 |
| LOC64599 | 1.340987 | 7.286898 | 7.333357 | 3.71E-10 | 4.09E-09 | 12.85942 |
| SNORA66 | -2.08885 | 9.646283 | -7.33313 | 3.72E-10 | 4.09E-09 | 12.85851 |
| LRAT | 1.318791 | 7.875361 | 7.331518 | 3.74E-10 | 4.11E-09 | 12.85192 |
| DKC1 | -0.90241 | 7.893617 | -7.33117 | 3.75E-10 | 4.12E-09 | 12.8505 |
| JAK1 | -1.19775 | 8.18815 | -7.33086 | 3.75E-10 | 4.12E-09 | 12.84924 |
| C1orf165 | 1.758197 | 7.570854 | 7.328772 | 3.79E-10 | 4.15E-09 | 12.84074 |
| HSP90AA1 | -1.73055 | 9.554996 | -7.32831 | 3.79E-10 | 4.16E-09 | 12.83886 |
| CTSZ | -1.77771 | 8.873175 | -7.32694 | 3.81E-10 | 4.17E-09 | 12.83327 |
| LEP | 1.146135 | 8.032735 | 7.326891 | 3.81E-10 | 4.17E-09 | 12.83308 |
| TRPM4 | -1.74371 | 8.681088 | -7.32457 | 3.85E-10 | 4.21E-09 | 12.82361 |
| FAM187B | 1.562439 | 7.231523 | 7.324212 | 3.86E-10 | 4.21E-09 | 12.82216 |
| ARPC5 | -1.1905 | 8.471121 | -7.32293 | 3.88E-10 | 4.23E-09 | 12.81693 |
| STAB2 | 1.657579 | 7.714698 | 7.322628 | 3.88E-10 | 4.24E-09 | 12.81571 |
| PQLC1 | -1.73954 | 8.927241 | -7.32249 | 3.89E-10 | 4.24E-09 | 12.81515 |
| HDDC2 | -1.44906 | 8.324499 | -7.31945 | 3.93E-10 | 4.29E-09 | 12.80277 |
| AMTN | 0.983575 | 6.932539 | 7.315725 | 4.00E-10 | 4.35E-09 | 12.78759 |
| SFTPD | 0.980197 | 7.187991 | 7.314825 | 4.01E-10 | 4.36E-09 | 12.78392 |
| LOC40101 | -1.62243 | 11.10331 | -7.31473 | 4.01E-10 | 4.36E-09 | 12.78353 |
| IGSF11 | 1.064864 | 7.36453 | 7.312231 | 4.05E-10 | 4.40E-09 | 12.77336 |
| C3orf22 | 1.276193 | 7.252124 | 7.308891 | 4.11E-10 | 4.45E-09 | 12.75976 |
| PCA3 | 1.524728 | 7.209811 | 7.30819 | 4.12E-10 | 4.46E-09 | 12.7569 |
| SLC16A3 | -1.32872 | 7.805176 | -7.30784 | 4.13E-10 | 4.47E-09 | 12.75549 |
| NCRNA00 | 0.883248 | 6.912034 | 7.304471 | 4.19E-10 | 4.53E-09 | 12.74176 |
| PHYH | -1.284 | 8.333553 | -7.30441 | 4.19E-10 | 4.53E-09 | 12.74151 |

| | | | | | | |
|---|---|---|---|---|---|---|
| ANGPTL5 | 1.445721 | 7.112646 | 7.302835 | 4.22E-10 | 4.55E-09 | 12.73509 |
| TFAP2C | 0.905142 | 6.905921 | 7.301044 | 4.25E-10 | 4.58E-09 | 12.7278 |
| C6orf145 | -1.02906 | 7.996119 | -7.30038 | 4.26E-10 | 4.59E-09 | 12.7251 |
| ARC | 1.584215 | 7.410068 | 7.296353 | 4.33E-10 | 4.67E-09 | 12.7087 |
| MED6 | -1.69558 | 8.467328 | -7.29594 | 4.34E-10 | 4.67E-09 | 12.707 |
| APOC1 | -2.14052 | 9.7957 | -7.29518 | 4.35E-10 | 4.68E-09 | 12.70393 |
| MGST1 | -1.80283 | 9.459018 | -7.29425 | 4.37E-10 | 4.70E-09 | 12.70014 |
| IL13RA1 | -1.27668 | 7.867928 | -7.29371 | 4.38E-10 | 4.71E-09 | 12.69793 |
| RAB25 | 1.136428 | 7.314561 | 7.292896 | 4.39E-10 | 4.72E-09 | 12.69462 |
| PKD1L2 | 0.736584 | 6.890167 | 7.291776 | 4.41E-10 | 4.74E-09 | 12.69006 |
| KIAA0319 | 1.253035 | 7.062294 | 7.290365 | 4.44E-10 | 4.77E-09 | 12.68431 |
| LOC39974 | 1.411054 | 8.860872 | 7.289858 | 4.45E-10 | 4.77E-09 | 12.68225 |
| ZNF827 | 2.266673 | 8.786009 | 7.289388 | 4.46E-10 | 4.78E-09 | 12.68034 |
| LYPD6B | 0.720969 | 6.827162 | 7.288202 | 4.48E-10 | 4.80E-09 | 12.6755 |
| CD7 | -1.38065 | 7.911124 | -7.28794 | 4.48E-10 | 4.80E-09 | 12.67445 |
| AUP1 | -1.30495 | 8.687233 | -7.28731 | 4.50E-10 | 4.81E-09 | 12.67186 |
| C1orf95 | 1.399576 | 7.566724 | 7.286561 | 4.51E-10 | 4.82E-09 | 12.66883 |
| DNTT | 0.824232 | 6.876623 | 7.284419 | 4.55E-10 | 4.86E-09 | 12.6601 |
| SCN1A | 0.829113 | 6.880854 | 7.282937 | 4.58E-10 | 4.89E-09 | 12.65407 |
| TMEM218 | -1.09144 | 7.584817 | -7.28245 | 4.59E-10 | 4.90E-09 | 12.65211 |
| PRSS1 | 2.646841 | 8.156152 | 7.281523 | 4.60E-10 | 4.91E-09 | 12.64832 |
| HCN1 | 1.272849 | 7.219902 | 7.281044 | 4.61E-10 | 4.92E-09 | 12.64637 |
| PGA5 | 2.595608 | 8.179833 | 7.279307 | 4.65E-10 | 4.95E-09 | 12.63929 |
| ZNF100 | 1.694998 | 9.878855 | 7.277712 | 4.68E-10 | 4.98E-09 | 12.6328 |
| LOC64152 | 0.919289 | 7.745888 | 7.276559 | 4.70E-10 | 5.00E-09 | 12.62811 |
| TAC4 | 1.030023 | 6.983512 | 7.275279 | 4.73E-10 | 5.03E-09 | 12.62289 |
| CHIT1 | 2.432024 | 7.835576 | 7.274498 | 4.74E-10 | 5.04E-09 | 12.61972 |
| TSPAN17 | -1.47512 | 8.173369 | -7.27411 | 4.75E-10 | 5.05E-09 | 12.61815 |
| HYOU1 | -0.80583 | 7.701295 | -7.27345 | 4.76E-10 | 5.06E-09 | 12.61544 |
| TFAP2A | 1.051549 | 7.132561 | 7.273186 | 4.77E-10 | 5.06E-09 | 12.61437 |
| RNF121 | -0.90929 | 8.03347 | -7.27103 | 4.81E-10 | 5.10E-09 | 12.60558 |
| MSRB2 | -2.06286 | 8.581297 | -7.27094 | 4.81E-10 | 5.10E-09 | 12.60523 |
| LOC7311C | 1.020767 | 6.999461 | 7.269655 | 4.84E-10 | 5.13E-09 | 12.6 |
| TAF6L | -1.74519 | 8.767862 | -7.26701 | 4.89E-10 | 5.18E-09 | 12.58923 |
| ST8SIA2 | 1.100387 | 7.023591 | 7.263886 | 4.95E-10 | 5.24E-09 | 12.57652 |
| SCARNA8 | -2.17858 | 9.441416 | -7.25985 | 5.04E-10 | 5.33E-09 | 12.56011 |
| C8ORFK36 | 0.792657 | 6.982147 | 7.259598 | 5.04E-10 | 5.33E-09 | 12.55907 |
| DHRS7 | -0.84347 | 7.366066 | -7.25841 | 5.07E-10 | 5.35E-09 | 12.55425 |
| PPIB | -0.98187 | 7.414629 | -7.25661 | 5.11E-10 | 5.39E-09 | 12.5469 |
| SDF2L1 | -2.13023 | 10.0437 | -7.25616 | 5.12E-10 | 5.40E-09 | 12.54506 |
| HCRTR2 | 0.771765 | 7.113897 | 7.25408 | 5.16E-10 | 5.44E-09 | 12.53661 |
| HMGCS2 | -1.86749 | 8.689358 | -7.25366 | 5.17E-10 | 5.45E-09 | 12.53491 |
| C11orf72 | 1.375598 | 7.06485 | 7.253279 | 5.18E-10 | 5.46E-09 | 12.53335 |
| OR10G7 | 1.123427 | 7.08669 | 7.252354 | 5.20E-10 | 5.47E-09 | 12.52959 |
| CFI | -1.1932 | 8.151971 | -7.25222 | 5.20E-10 | 5.47E-09 | 12.52905 |
| OR10X1 | 0.916498 | 6.843411 | 7.250792 | 5.23E-10 | 5.50E-09 | 12.52323 |
| KIF4B | 1.117978 | 7.113021 | 7.250153 | 5.24E-10 | 5.52E-09 | 12.52063 |
| FAHD2A | -1.06705 | 7.677991 | -7.24875 | 5.28E-10 | 5.54E-09 | 12.51491 |
| ZNF528 | 1.375495 | 7.981143 | 7.248498 | 5.28E-10 | 5.55E-09 | 12.5139 |
| RPL12 | -1.68076 | 9.96032 | -7.24814 | 5.29E-10 | 5.55E-09 | 12.51243 |
| KRTAP12- | 1.342032 | 7.084277 | 7.246298 | 5.33E-10 | 5.59E-09 | 12.50494 |
| ELA2A | 2.126143 | 7.68722 | 7.246247 | 5.33E-10 | 5.59E-09 | 12.50473 |
| RAET1E | 1.749103 | 7.379407 | 7.244236 | 5.37E-10 | 5.63E-09 | 12.49655 |
| CDAN1 | 1.333504 | 9.886764 | 7.242157 | 5.42E-10 | 5.68E-09 | 12.4881 |
| GPR32 | 1.035354 | 7.017057 | 7.239236 | 5.49E-10 | 5.74E-09 | 12.47621 |
| PLIN2 | -1.25184 | 8.721272 | -7.23858 | 5.50E-10 | 5.75E-09 | 12.47356 |
| ADCYAP1 | 0.765138 | 6.760126 | 7.238557 | 5.50E-10 | 5.75E-09 | 12.47345 |
| C19orf2 | -1.17129 | 8.634116 | -7.23767 | 5.52E-10 | 5.77E-09 | 12.46985 |

| | | | | | | |
|---|---|---|---|---|---|---|
| ABHD14B | -1.10391 | 8.003277 | -7.23625 | 5.56E-10 | 5.80E-09 | 12.46404 |
| OSBPL8 | -1.12151 | 7.852471 | -7.23623 | 5.56E-10 | 5.80E-09 | 12.46398 |
| IFRG15 | -0.85538 | 7.315345 | -7.23517 | 5.58E-10 | 5.82E-09 | 12.45965 |
| OSTN | 0.861774 | 6.739235 | 7.234994 | 5.58E-10 | 5.82E-09 | 12.45895 |
| PSMD4 | -0.89716 | 7.855021 | -7.23459 | 5.59E-10 | 5.83E-09 | 12.45729 |
| CUTA | -0.8446 | 7.47489 | -7.23282 | 5.64E-10 | 5.87E-09 | 12.45009 |
| KRT15 | 1.107498 | 7.062999 | 7.232379 | 5.65E-10 | 5.87E-09 | 12.44831 |
| PDE4B | 0.915976 | 7.378319 | 7.232053 | 5.65E-10 | 5.88E-09 | 12.44698 |
| NCAM1 | 0.704475 | 7.116339 | 7.230581 | 5.69E-10 | 5.91E-09 | 12.441 |
| EIF5A | -0.73356 | 7.563052 | -7.22939 | 5.72E-10 | 5.94E-09 | 12.43615 |
| FLJ30851 | 1.366753 | 7.150041 | 7.226638 | 5.78E-10 | 6.00E-09 | 12.42496 |
| MGC1629 | 0.914772 | 6.887045 | 7.22478 | 5.83E-10 | 6.04E-09 | 12.4174 |
| BRD7 | -0.91715 | 7.375491 | -7.22035 | 5.93E-10 | 6.15E-09 | 12.39939 |
| C12orf59 | 0.842366 | 6.95102 | 7.219469 | 5.96E-10 | 6.17E-09 | 12.3958 |
| WFS1 | -1.16781 | 7.618235 | -7.21783 | 6.00E-10 | 6.21E-09 | 12.38913 |
| MTIF3 | -0.9702 | 7.297565 | -7.21374 | 6.10E-10 | 6.31E-09 | 12.37248 |
| HLA-DMB | -1.87342 | 8.568372 | -7.2133 | 6.11E-10 | 6.32E-09 | 12.37071 |
| GNMT | -2.22332 | 8.18509 | -7.21177 | 6.15E-10 | 6.35E-09 | 12.3645 |
| NENF | -1.8011 | 9.241699 | -7.21124 | 6.16E-10 | 6.36E-09 | 12.36233 |
| ZSWIM4 | 1.193166 | 10.27578 | 7.211018 | 6.17E-10 | 6.36E-09 | 12.36143 |
| ZNF593 | -1.30102 | 8.175741 | -7.20951 | 6.21E-10 | 6.40E-09 | 12.35529 |
| PTK6 | 1.675753 | 7.936432 | 7.208215 | 6.24E-10 | 6.43E-09 | 12.35003 |
| LRIT1 | 1.65831 | 7.254753 | 7.206097 | 6.30E-10 | 6.49E-09 | 12.34141 |
| OR2M5 | 1.335691 | 7.215751 | 7.202479 | 6.39E-10 | 6.58E-09 | 12.3267 |
| CPEB1 | 0.994132 | 7.142979 | 7.201861 | 6.41E-10 | 6.59E-09 | 12.32419 |
| FLJ45966 | 0.845429 | 7.188495 | 7.201617 | 6.41E-10 | 6.59E-09 | 12.3232 |
| LOC40135 | 1.237571 | 11.21511 | 7.201291 | 6.42E-10 | 6.60E-09 | 12.32187 |
| MAPK1 | -0.7407 | 7.912157 | -7.20092 | 6.43E-10 | 6.61E-09 | 12.32035 |
| FRMPD2 | 1.012731 | 7.159288 | 7.198914 | 6.49E-10 | 6.66E-09 | 12.31221 |
| TMEM180 | -1.0582 | 7.502122 | -7.19858 | 6.49E-10 | 6.66E-09 | 12.31084 |
| SOX11 | 2.259951 | 7.895979 | 7.198297 | 6.50E-10 | 6.66E-09 | 12.30969 |
| INA | 1.09534 | 6.845691 | 7.1974 | 6.53E-10 | 6.68E-09 | 12.30605 |
| TCEB1 | -0.97204 | 7.639326 | -7.1966 | 6.55E-10 | 6.70E-09 | 12.30279 |
| CFD | -1.71413 | 8.712864 | -7.19464 | 6.60E-10 | 6.75E-09 | 12.29483 |
| EIF4E2 | -0.85814 | 8.335891 | -7.19446 | 6.61E-10 | 6.75E-09 | 12.2941 |
| KBTBD12 | 1.380885 | 7.132917 | 7.194314 | 6.61E-10 | 6.76E-09 | 12.2935 |
| TBC1D3B | 1.219921 | 8.065636 | 7.193018 | 6.65E-10 | 6.79E-09 | 12.28823 |
| SNORD59 | -1.3423 | 8.382976 | -7.19204 | 6.67E-10 | 6.81E-09 | 12.28427 |
| OR2L13 | 1.547717 | 7.311318 | 7.191332 | 6.69E-10 | 6.83E-09 | 12.28138 |
| TRMT1 | -0.83111 | 8.253576 | -7.19021 | 6.72E-10 | 6.86E-09 | 12.27683 |
| ABCC1 | 1.390574 | 8.238846 | 7.190056 | 6.73E-10 | 6.86E-09 | 12.27619 |
| ATAD3B | -1.23799 | 7.495236 | -7.19004 | 6.73E-10 | 6.86E-09 | 12.27611 |
| ITPRIP | -0.73775 | 7.274787 | -7.18907 | 6.76E-10 | 6.88E-09 | 12.27218 |
| PRDM11 | 0.995059 | 6.943943 | 7.1865 | 6.83E-10 | 6.95E-09 | 12.26173 |
| DBC1 | 0.823503 | 6.885638 | 7.183934 | 6.90E-10 | 7.01E-09 | 12.2513 |
| ATP5J2 | -1.89945 | 8.899948 | -7.18377 | 6.91E-10 | 7.01E-09 | 12.25065 |
| TMEM14C | -0.89695 | 7.332385 | -7.18368 | 6.91E-10 | 7.01E-09 | 12.25027 |
| LOC73041 | 1.701621 | 7.905161 | 7.181649 | 6.97E-10 | 7.07E-09 | 12.24202 |
| TNFRSF10 | -0.71318 | 7.646882 | -7.18097 | 6.99E-10 | 7.09E-09 | 12.23925 |
| LOC38928 | 1.714756 | 10.91956 | 7.179653 | 7.02E-10 | 7.12E-09 | 12.2339 |
| EGFR | -0.77188 | 7.603138 | -7.17918 | 7.04E-10 | 7.13E-09 | 12.23197 |
| GTPBP6 | -1.96014 | 11.31292 | -7.17889 | 7.05E-10 | 7.14E-09 | 12.23082 |
| SLC32A1 | 1.03558 | 6.905283 | 7.177887 | 7.08E-10 | 7.16E-09 | 12.22672 |
| ADAM15 | -0.91018 | 9.323388 | -7.17722 | 7.10E-10 | 7.18E-09 | 12.22401 |
| C1orf140 | 1.086358 | 7.065566 | 7.176627 | 7.11E-10 | 7.19E-09 | 12.2216 |
| SNORA72 | -1.68415 | 8.176665 | -7.17595 | 7.13E-10 | 7.21E-09 | 12.21887 |
| EDARADD | 0.8153 | 7.107667 | 7.175828 | 7.14E-10 | 7.21E-09 | 12.21836 |
| TIMP1 | -2.04224 | 9.890357 | -7.1756 | 7.14E-10 | 7.21E-09 | 12.21743 |

| | | | | | | |
|---|---|---|---|---|---|---|
| APOC3 | -2.33789 | 11.21214 | -7.17172 | 7.26E-10 | 7.32E-09 | 12.20165 |
| MGC3990 | 0.701293 | 6.821411 | 7.171676 | 7.26E-10 | 7.32E-09 | 12.20148 |
| POLR2F | -1.12495 | 8.143221 | -7.17086 | 7.28E-10 | 7.34E-09 | 12.19818 |
| NPFFR2 | 1.007834 | 7.087316 | 7.16862 | 7.35E-10 | 7.40E-09 | 12.18906 |
| CBWD5 | -0.93905 | 7.334564 | -7.16757 | 7.38E-10 | 7.43E-09 | 12.18478 |
| SPCS3 | -0.86874 | 7.351954 | -7.1672 | 7.40E-10 | 7.44E-09 | 12.18328 |
| PDIA5 | -0.82972 | 7.095853 | -7.16225 | 7.55E-10 | 7.57E-09 | 12.16319 |
| HIC2 | 1.467024 | 8.734513 | 7.161384 | 7.58E-10 | 7.60E-09 | 12.15965 |
| SLC9A2 | 1.103349 | 7.296873 | 7.15973 | 7.63E-10 | 7.65E-09 | 12.15293 |
| SLC30A1 | -0.95541 | 7.73377 | -7.15905 | 7.65E-10 | 7.66E-09 | 12.15018 |
| FGL1 | -1.6926 | 9.035109 | -7.15861 | 7.66E-10 | 7.67E-09 | 12.14837 |
| HIATL1 | -1.54957 | 8.672807 | -7.15794 | 7.68E-10 | 7.69E-09 | 12.14567 |
| CSN1S1 | 0.924321 | 6.980353 | 7.156781 | 7.72E-10 | 7.72E-09 | 12.14095 |
| GOLGA3 | -1.16575 | 7.828495 | -7.15618 | 7.74E-10 | 7.74E-09 | 12.1385 |
| RNASET2 | -0.96 | 7.269582 | -7.15507 | 7.78E-10 | 7.77E-09 | 12.13398 |
| PCSK6 | -0.91885 | 8.100479 | -7.15395 | 7.81E-10 | 7.79E-09 | 12.12946 |
| RUNDC2A | 1.096947 | 7.475523 | 7.153908 | 7.81E-10 | 7.79E-09 | 12.12928 |
| MIR1911 | 1.232371 | 6.988956 | 7.15371 | 7.82E-10 | 7.79E-09 | 12.12847 |
| OR2AT4 | 1.83481 | 7.378363 | 7.153505 | 7.83E-10 | 7.80E-09 | 12.12764 |
| C6orf27 | 1.95271 | 8.495948 | 7.152543 | 7.86E-10 | 7.82E-09 | 12.12373 |
| ETV3L | 2.618629 | 8.206523 | 7.150194 | 7.94E-10 | 7.90E-09 | 12.11419 |
| GBX2 | 1.666638 | 7.293797 | 7.146432 | 8.06E-10 | 8.01E-09 | 12.09891 |
| OR8K5 | 0.771051 | 6.934931 | 7.145057 | 8.11E-10 | 8.05E-09 | 12.09332 |
| PHPT1 | -0.99432 | 8.295575 | -7.14418 | 8.14E-10 | 8.08E-09 | 12.08976 |
| ADI1 | -1.2814 | 7.855634 | -7.14376 | 8.15E-10 | 8.09E-09 | 12.08807 |
| FLJ35390 | 1.356703 | 11.50523 | 7.141349 | 8.23E-10 | 8.15E-09 | 12.07826 |
| LAS1L | -1.06134 | 7.816519 | -7.14041 | 8.26E-10 | 8.18E-09 | 12.07445 |
| ACTB | -1.73013 | 8.828658 | -7.13853 | 8.33E-10 | 8.24E-09 | 12.0668 |
| ENPEP | -0.72007 | 7.225722 | -7.13824 | 8.34E-10 | 8.24E-09 | 12.06562 |
| ZNHIT1 | -1.29101 | 7.844035 | -7.13686 | 8.39E-10 | 8.29E-09 | 12.06002 |
| PAX8 | 0.895145 | 7.385388 | 7.135982 | 8.42E-10 | 8.31E-09 | 12.05645 |
| ACER1 | 1.003463 | 7.056713 | 7.135707 | 8.43E-10 | 8.32E-09 | 12.05534 |
| C14orf102 | -1.01435 | 7.654486 | -7.13176 | 8.56E-10 | 8.45E-09 | 12.0393 |
| TMEM156 | 1.474693 | 8.390279 | 7.131546 | 8.57E-10 | 8.46E-09 | 12.03844 |
| PARP3 | -0.94462 | 7.705577 | -7.13138 | 8.58E-10 | 8.46E-09 | 12.03778 |
| CCR7 | 1.544995 | 7.716494 | 7.129738 | 8.64E-10 | 8.50E-09 | 12.03109 |
| C10orf116 | -2.13352 | 10.09524 | -7.12805 | 8.70E-10 | 8.55E-09 | 12.02426 |
| HOXB1 | 1.882379 | 7.517913 | 7.12708 | 8.73E-10 | 8.58E-09 | 12.0203 |
| MYO3B | 0.866317 | 6.979306 | 7.127057 | 8.73E-10 | 8.58E-09 | 12.02021 |
| C2orf66 | 2.58097 | 8.291206 | 7.126079 | 8.77E-10 | 8.61E-09 | 12.01624 |
| DAD1L | 1.419514 | 7.191425 | 7.126003 | 8.77E-10 | 8.61E-09 | 12.01593 |
| IGFBP7 | -1.47634 | 8.02541 | -7.12565 | 8.78E-10 | 8.62E-09 | 12.01449 |
| VWA5B1 | 1.844313 | 7.417844 | 7.12498 | 8.81E-10 | 8.64E-09 | 12.01177 |
| KIF17 | 1.58972 | 7.982984 | 7.124817 | 8.81E-10 | 8.64E-09 | 12.01111 |
| OR5P2 | 0.726666 | 6.835312 | 7.124617 | 8.82E-10 | 8.64E-09 | 12.0103 |
| GAPT | 1.157433 | 7.309427 | 7.12454 | 8.82E-10 | 8.64E-09 | 12.00998 |
| LOC44129 | 1.27091 | 7.386509 | 7.124032 | 8.84E-10 | 8.65E-09 | 12.00792 |
| FAM65A | -1.66592 | 8.160298 | -7.12217 | 8.91E-10 | 8.70E-09 | 12.00038 |
| SAMD9 | 0.817848 | 7.104261 | 7.122041 | 8.92E-10 | 8.70E-09 | 11.99984 |
| SNORA3 | -2.17992 | 11.42194 | -7.12204 | 8.92E-10 | 8.70E-09 | 11.99983 |
| UBE2H | -0.76865 | 7.537957 | -7.12141 | 8.94E-10 | 8.72E-09 | 11.99727 |
| MIR1471 | 0.704919 | 6.848345 | 7.121191 | 8.95E-10 | 8.72E-09 | 11.99639 |
| PIK3CD | 1.517917 | 8.52498 | 7.121128 | 8.95E-10 | 8.72E-09 | 11.99613 |
| RNU4-2 | -2.28428 | 10.81722 | -7.12075 | 8.96E-10 | 8.72E-09 | 11.9946 |
| SLCO2B1 | -1.04668 | 7.831281 | -7.12067 | 8.97E-10 | 8.72E-09 | 11.99426 |
| COX6C | -1.14548 | 7.951857 | -7.12013 | 8.99E-10 | 8.74E-09 | 11.99207 |
| KRT85 | 1.491817 | 7.307746 | 7.119641 | 9.01E-10 | 8.75E-09 | 11.99009 |
| MT1IP | 1.097552 | 7.05512 | 7.118984 | 9.03E-10 | 8.77E-09 | 11.98743 |

| | | | | | | |
|---|---|---|---|---|---|---|
| APOM | -1.53615 | 7.84821 | -7.11893 | 9.03E-10 | 8.77E-09 | 11.98721 |
| PRR20C | 2.995591 | 8.577458 | 7.118525 | 9.05E-10 | 8.77E-09 | 11.98556 |
| SP8 | 0.836639 | 6.923851 | 7.117803 | 9.07E-10 | 8.80E-09 | 11.98263 |
| PTRF | -1.14593 | 7.681708 | -7.11658 | 9.12E-10 | 8.83E-09 | 11.97768 |
| TTC21A | 1.564901 | 7.878089 | 7.115805 | 9.15E-10 | 8.86E-09 | 11.97452 |
| IPO11 | -0.99562 | 7.881855 | -7.11563 | 9.16E-10 | 8.86E-09 | 11.97381 |
| NPHS2 | 1.270975 | 7.106312 | 7.112994 | 9.26E-10 | 8.95E-09 | 11.9631 |
| KIAA0802 | 1.560837 | 8.345694 | 7.112064 | 9.29E-10 | 8.98E-09 | 11.95933 |
| EPHA3 | 0.932025 | 7.509773 | 7.11193 | 9.30E-10 | 8.98E-09 | 11.95878 |
| MOSC2 | -0.73577 | 7.050415 | -7.11052 | 9.35E-10 | 9.03E-09 | 11.95306 |
| CIB3 | 1.362577 | 7.181686 | 7.110237 | 9.36E-10 | 9.04E-09 | 11.95191 |
| LOC10012 | 0.992813 | 7.005946 | 7.10915 | 9.40E-10 | 9.07E-09 | 11.9475 |
| ZNF207 | -1.34518 | 8.378144 | -7.1074 | 9.47E-10 | 9.11E-09 | 11.94039 |
| HRSP12 | -0.96379 | 7.35975 | -7.10733 | 9.48E-10 | 9.11E-09 | 11.94011 |
| SST | 0.814281 | 6.870568 | 7.106912 | 9.49E-10 | 9.12E-09 | 11.93841 |
| VIPR2 | 0.801526 | 6.939919 | 7.106516 | 9.51E-10 | 9.14E-09 | 11.9368 |
| CNIH4 | -0.9926 | 8.183957 | -7.10622 | 9.52E-10 | 9.14E-09 | 11.9356 |
| C11orf44 | 0.767874 | 6.746269 | 7.105786 | 9.54E-10 | 9.15E-09 | 11.93384 |
| SNORA73. | -1.78115 | 9.050245 | -7.10574 | 9.54E-10 | 9.15E-09 | 11.93364 |
| OR10H3 | 0.798444 | 6.833761 | 7.1044 | 9.59E-10 | 9.20E-09 | 11.92822 |
| NOX1 | 0.914273 | 6.977119 | 7.103822 | 9.61E-10 | 9.22E-09 | 11.92587 |
| PSMA1 | -0.80961 | 7.860072 | -7.10367 | 9.62E-10 | 9.22E-09 | 11.92526 |
| POM121C | -1.384 | 8.643827 | -7.10143 | 9.71E-10 | 9.30E-09 | 11.91616 |
| TPRG1L | -1.81968 | 8.615045 | -7.10038 | 9.75E-10 | 9.34E-09 | 11.9119 |
| PADI3 | 1.190224 | 7.09316 | 7.099238 | 9.80E-10 | 9.37E-09 | 11.90726 |
| TNIP3 | 2.275376 | 8.193973 | 7.098864 | 9.81E-10 | 9.38E-09 | 11.90575 |
| FABP6 | 2.429558 | 7.789039 | 7.098072 | 9.85E-10 | 9.40E-09 | 11.90253 |
| DPP7 | -1.09938 | 7.988576 | -7.09801 | 9.85E-10 | 9.40E-09 | 11.90229 |
| ARSA | -1.48433 | 8.16844 | -7.09765 | 9.86E-10 | 9.41E-09 | 11.90084 |
| DNAJB2 | -1.24429 | 8.253185 | -7.09634 | 9.92E-10 | 9.46E-09 | 11.89551 |
| OR51I2 | 0.838882 | 6.864678 | 7.095017 | 9.97E-10 | 9.51E-09 | 11.89013 |
| MPP6 | -1.01765 | 7.423901 | -7.09456 | 9.99E-10 | 9.52E-09 | 11.88828 |
| TMED1 | -1.7194 | 9.114202 | -7.08935 | 1.02E-09 | 9.72E-09 | 11.86714 |
| GNL2 | -1.89975 | 8.298811 | -7.08753 | 1.03E-09 | 9.78E-09 | 11.85976 |
| WDR51A | -1.02498 | 7.900006 | -7.08107 | 1.06E-09 | 1.00E-08 | 11.83355 |
| EEF2 | -1.40363 | 8.232022 | -7.08073 | 1.06E-09 | 1.00E-08 | 11.83216 |
| MAL | 1.431017 | 7.459356 | 7.079644 | 1.06E-09 | 1.01E-08 | 11.82774 |
| OTOA | 0.943938 | 6.941117 | 7.079451 | 1.06E-09 | 1.01E-08 | 11.82696 |
| OR8U1 | 1.157902 | 7.00683 | 7.078453 | 1.07E-09 | 1.01E-08 | 11.82291 |
| SERPINF2 | -1.30415 | 10.30206 | -7.07768 | 1.07E-09 | 1.02E-08 | 11.81976 |
| PTH1R | 0.888797 | 7.411433 | 7.077593 | 1.07E-09 | 1.02E-08 | 11.81942 |
| FRMPD2L: | 0.977073 | 6.958752 | 7.076858 | 1.07E-09 | 1.02E-08 | 11.81644 |
| CORO1B | -0.87184 | 8.080576 | -7.07606 | 1.08E-09 | 1.02E-08 | 11.81319 |
| RUNDC2B | 1.593008 | 10.2859 | 7.075799 | 1.08E-09 | 1.02E-08 | 11.81214 |
| BAMBI | -1.78802 | 8.504495 | -7.07494 | 1.08E-09 | 1.02E-08 | 11.80867 |
| KIF5B | -0.7542 | 7.616689 | -7.07462 | 1.08E-09 | 1.03E-08 | 11.80738 |
| C1orf158 | 1.407488 | 7.307554 | 7.073762 | 1.09E-09 | 1.03E-08 | 11.80388 |
| LMO3 | 0.818436 | 6.961621 | 7.071873 | 1.10E-09 | 1.04E-08 | 11.79622 |
| EOMES | 1.713513 | 8.34822 | 7.070263 | 1.10E-09 | 1.04E-08 | 11.78968 |
| SPATA2L | -1.34766 | 8.181682 | -7.0679 | 1.12E-09 | 1.05E-08 | 11.78011 |
| OR2A14 | 1.510409 | 7.340099 | 7.067864 | 1.12E-09 | 1.05E-08 | 11.77995 |
| MUCL1 | 1.855203 | 7.580516 | 7.061615 | 1.14E-09 | 1.08E-08 | 11.7546 |
| OR51I1 | 0.851542 | 6.854086 | 7.060162 | 1.15E-09 | 1.08E-08 | 11.74871 |
| POLR2I | -1.0385 | 7.481205 | -7.05827 | 1.16E-09 | 1.09E-08 | 11.74105 |
| NCOA2 | -1.08803 | 7.991945 | -7.05607 | 1.17E-09 | 1.10E-08 | 11.73213 |
| FAM158A | -1.55565 | 8.74047 | -7.05588 | 1.17E-09 | 1.10E-08 | 11.73133 |
| ELSPBP1 | 1.181883 | 6.973026 | 7.055646 | 1.17E-09 | 1.10E-08 | 11.73039 |
| DLX4 | 1.105841 | 7.173208 | 7.052447 | 1.19E-09 | 1.11E-08 | 11.71742 |

| | | | | | | |
|---|---|---|---|---|---|---|
| RPLP0P2 | 1.312089 | 7.939178 | 7.052305 | 1.19E-09 | 1.11E-08 | 11.71684 |
| TAS1R2 | 1.115448 | 7.016649 | 7.05214 | 1.19E-09 | 1.11E-08 | 11.71618 |
| MRPL22 | -0.97777 | 7.468134 | -7.05193 | 1.19E-09 | 1.12E-08 | 11.71531 |
| ABHD9 | 1.899018 | 8.021949 | 7.051378 | 1.19E-09 | 1.12E-08 | 11.71308 |
| ZYX | -0.8112 | 7.31214 | -7.05129 | 1.19E-09 | 1.12E-08 | 11.71271 |
| TMSB10 | -0.78078 | 7.158534 | -7.05111 | 1.20E-09 | 1.12E-08 | 11.71199 |
| TCP10 | 0.741625 | 6.855257 | 7.050253 | 1.20E-09 | 1.12E-08 | 11.70852 |
| ROMO1 | -1.4752 | 8.250047 | -7.04927 | 1.20E-09 | 1.12E-08 | 11.70455 |
| MAF | -1.33486 | 7.888481 | -7.0491 | 1.21E-09 | 1.12E-08 | 11.70386 |
| SIGLECP3 | 0.741721 | 6.955862 | 7.048182 | 1.21E-09 | 1.13E-08 | 11.70013 |
| GCN1L1 | -1.01244 | 7.838295 | -7.04796 | 1.21E-09 | 1.13E-08 | 11.69923 |
| BRUNOL4 | 1.079884 | 7.006488 | 7.047091 | 1.22E-09 | 1.13E-08 | 11.6957 |
| PHC1 | 1.276378 | 7.891008 | 7.046794 | 1.22E-09 | 1.13E-08 | 11.6945 |
| ANGPT4 | 1.896439 | 7.625146 | 7.046606 | 1.22E-09 | 1.13E-08 | 11.69374 |
| CHD1L | -0.93188 | 7.745524 | -7.04633 | 1.22E-09 | 1.13E-08 | 11.69263 |
| UBD | -2.13358 | 9.788285 | -7.04466 | 1.23E-09 | 1.14E-08 | 11.68583 |
| EIF4A1 | -1.28388 | 8.892377 | -7.04307 | 1.24E-09 | 1.15E-08 | 11.67938 |
| SNORA5A | -1.59681 | 8.663261 | -7.04272 | 1.24E-09 | 1.15E-08 | 11.67798 |
| C14orf166 | -0.81392 | 7.405812 | -7.04209 | 1.24E-09 | 1.15E-08 | 11.67543 |
| IL1B | 1.37555 | 7.645716 | 7.04133 | 1.24E-09 | 1.15E-08 | 11.67234 |
| ADRM1 | -1.04129 | 8.422199 | -7.04049 | 1.25E-09 | 1.16E-08 | 11.66893 |
| ETFA | -0.72 | 7.441505 | -7.04036 | 1.25E-09 | 1.16E-08 | 11.66842 |
| CDC16 | -1.14395 | 7.727608 | -7.04014 | 1.25E-09 | 1.16E-08 | 11.66752 |
| ZNF511 | -0.84772 | 7.618669 | -7.03774 | 1.26E-09 | 1.17E-08 | 11.65781 |
| UBXN6 | -0.90809 | 7.512468 | -7.03757 | 1.26E-09 | 1.17E-08 | 11.65709 |
| SNORD36 | -1.8554 | 8.883368 | -7.03716 | 1.27E-09 | 1.17E-08 | 11.65544 |
| RGR | 0.830871 | 6.960222 | 7.036636 | 1.27E-09 | 1.17E-08 | 11.65331 |
| C1orf123 | -1.03656 | 7.605073 | -7.03645 | 1.27E-09 | 1.17E-08 | 11.65257 |
| TM4SF1 | -1.7624 | 8.775766 | -7.0364 | 1.27E-09 | 1.17E-08 | 11.65234 |
| MSX1 | 1.234779 | 7.381569 | 7.036042 | 1.27E-09 | 1.18E-08 | 11.6509 |
| FBXO24 | 1.194381 | 7.341997 | 7.035674 | 1.27E-09 | 1.18E-08 | 11.64941 |
| OR4C6 | 1.276729 | 7.011383 | 7.034979 | 1.28E-09 | 1.18E-08 | 11.64659 |
| FOXD4L3 | 1.152374 | 7.09152 | 7.034026 | 1.28E-09 | 1.18E-08 | 11.64273 |
| CITED1 | 1.207756 | 7.293206 | 7.033689 | 1.28E-09 | 1.18E-08 | 11.64136 |
| KRT78 | 0.845166 | 6.925423 | 7.033584 | 1.29E-09 | 1.18E-08 | 11.64094 |
| DGAT1 | -1.28669 | 7.67548 | -7.03219 | 1.29E-09 | 1.19E-08 | 11.63528 |
| TMEM75 | 1.073836 | 7.045362 | 7.032125 | 1.29E-09 | 1.19E-08 | 11.63502 |
| UPF2 | -0.95687 | 7.843313 | -7.03153 | 1.30E-09 | 1.19E-08 | 11.63259 |
| ODF3 | 2.065884 | 7.563149 | 7.031269 | 1.30E-09 | 1.19E-08 | 11.63155 |
| MRGPRX4 | 1.448211 | 7.266796 | 7.029801 | 1.31E-09 | 1.20E-08 | 11.6256 |
| ZNF385C | 1.463746 | 7.466931 | 7.029718 | 1.31E-09 | 1.20E-08 | 11.62527 |
| CHD4 | -0.9946 | 7.599398 | -7.02909 | 1.31E-09 | 1.20E-08 | 11.62271 |
| STK33 | 1.112046 | 7.216318 | 7.02851 | 1.31E-09 | 1.21E-08 | 11.62037 |
| LOC40089 | 1.201889 | 7.102893 | 7.026186 | 1.33E-09 | 1.22E-08 | 11.61095 |
| HTR3C | 1.719067 | 7.603902 | 7.024962 | 1.33E-09 | 1.22E-08 | 11.60599 |
| CHST13 | -1.54138 | 8.958929 | -7.02469 | 1.33E-09 | 1.22E-08 | 11.60489 |
| RNF7 | -1.33867 | 8.736833 | -7.02238 | 1.35E-09 | 1.23E-08 | 11.59552 |
| PPP1R2P9 | 0.732834 | 6.82304 | 7.02232 | 1.35E-09 | 1.23E-08 | 11.59528 |
| ASGR2 | -1.38762 | 8.952199 | -7.01937 | 1.36E-09 | 1.25E-08 | 11.58334 |
| AMAC1L2 | 0.972099 | 6.93692 | 7.01884 | 1.37E-09 | 1.25E-08 | 11.58118 |
| HTR4 | 0.731139 | 6.946605 | 7.018648 | 1.37E-09 | 1.25E-08 | 11.5804 |
| SELPLG | 0.956324 | 7.066774 | 7.014984 | 1.39E-09 | 1.27E-08 | 11.56555 |
| POM121L4 | 2.147383 | 7.908907 | 7.014584 | 1.39E-09 | 1.27E-08 | 11.56393 |
| BEST2 | 1.825709 | 7.312474 | 7.013363 | 1.40E-09 | 1.28E-08 | 11.55898 |
| SDHA | -1.42926 | 8.594286 | -7.0133 | 1.40E-09 | 1.28E-08 | 11.55871 |
| KCNA1 | 0.873469 | 6.78398 | 7.012416 | 1.40E-09 | 1.28E-08 | 11.55514 |
| C6orf114 | -0.76361 | 7.257832 | -7.00998 | 1.42E-09 | 1.29E-08 | 11.54528 |
| SEC61B | -1.09673 | 7.737274 | -7.00995 | 1.42E-09 | 1.29E-08 | 11.54516 |

| | | | | | | |
|---|---|---|---|---|---|---|
| AIG1 | -1.06265 | 7.973873 | -7.00839 | 1.43E-09 | 1.30E-08 | 11.53885 |
| MSX2P1 | 1.044731 | 6.975466 | 7.0072 | 1.43E-09 | 1.30E-08 | 11.53401 |
| SNORA50 | -1.84225 | 9.337998 | -7.00662 | 1.44E-09 | 1.31E-08 | 11.53164 |
| SCGB1D4 | 1.098438 | 6.928159 | 7.005836 | 1.44E-09 | 1.31E-08 | 11.52848 |
| IFI6 | -1.10274 | 9.459762 | -7.00564 | 1.44E-09 | 1.31E-08 | 11.52769 |
| OR5H15 | 0.72102 | 6.709464 | 7.0032 | 1.46E-09 | 1.32E-08 | 11.5178 |
| EMD | -1.35188 | 8.069262 | -7.00067 | 1.47E-09 | 1.34E-08 | 11.50756 |
| GJD3 | 1.560759 | 8.017006 | 6.999772 | 1.48E-09 | 1.34E-08 | 11.50391 |
| RAB2A | -0.72652 | 7.55505 | -6.999 | 1.48E-09 | 1.34E-08 | 11.5008 |
| CHST10 | 1.853635 | 7.699436 | 6.997035 | 1.49E-09 | 1.35E-08 | 11.49283 |
| IDE | -1.10813 | 7.410001 | -6.99689 | 1.50E-09 | 1.35E-08 | 11.49226 |
| ZNF585A | 0.79754 | 7.093807 | 6.994937 | 1.51E-09 | 1.36E-08 | 11.48433 |
| PPYR1 | 1.13676 | 6.92415 | 6.994064 | 1.51E-09 | 1.37E-08 | 11.48079 |
| MIR1978 | 1.542821 | 10.89869 | 6.994034 | 1.51E-09 | 1.37E-08 | 11.48067 |
| C3orf43 | 0.841073 | 6.945359 | 6.99121 | 1.53E-09 | 1.38E-08 | 11.46923 |
| HSP90B3P | -0.87327 | 7.563647 | -6.98902 | 1.55E-09 | 1.39E-08 | 11.46037 |
| SPDEF | 1.189284 | 7.178656 | 6.988457 | 1.55E-09 | 1.40E-08 | 11.45808 |
| OR9K2 | 1.363983 | 7.092451 | 6.987609 | 1.55E-09 | 1.40E-08 | 11.45465 |
| SLC2A2 | -1.85004 | 8.819798 | -6.98579 | 1.57E-09 | 1.41E-08 | 11.44728 |
| OR52K2 | 1.039663 | 7.030427 | 6.984418 | 1.57E-09 | 1.42E-08 | 11.44172 |
| APOA1 | -2.83129 | 11.02461 | -6.98364 | 1.58E-09 | 1.42E-08 | 11.43858 |
| TSEN34 | -0.79966 | 7.382266 | -6.98293 | 1.58E-09 | 1.43E-08 | 11.43568 |
| XRN2 | -0.74665 | 7.171495 | -6.98232 | 1.59E-09 | 1.43E-08 | 11.43321 |
| ABCC8 | 2.334852 | 8.061141 | 6.980132 | 1.60E-09 | 1.44E-08 | 11.42437 |
| SFT2D2 | -0.71165 | 7.406132 | -6.97924 | 1.61E-09 | 1.45E-08 | 11.42076 |
| ATP8A2 | 1.629853 | 7.67129 | 6.976318 | 1.63E-09 | 1.46E-08 | 11.40892 |
| GFI1B | 0.784178 | 6.969604 | 6.973101 | 1.65E-09 | 1.48E-08 | 11.3959 |
| MIR1208 | 1.520532 | 7.23191 | 6.97294 | 1.65E-09 | 1.48E-08 | 11.39525 |
| TFF1 | 1.593108 | 7.201805 | 6.972639 | 1.65E-09 | 1.48E-08 | 11.39403 |
| TNFRSF12 | -1.1041 | 7.837819 | -6.97183 | 1.66E-09 | 1.49E-08 | 11.39075 |
| FKBP5 | -1.20346 | 8.184312 | -6.96985 | 1.67E-09 | 1.50E-08 | 11.38272 |
| YME1L1 | -0.83867 | 7.49839 | -6.96795 | 1.69E-09 | 1.51E-08 | 11.37504 |
| TBC1D3G | 1.249574 | 7.972602 | 6.967625 | 1.69E-09 | 1.51E-08 | 11.37373 |
| OR1M1 | 1.815793 | 7.289875 | 6.96733 | 1.69E-09 | 1.51E-08 | 11.37253 |
| HCFC1R1 | -0.87036 | 7.743254 | -6.96699 | 1.69E-09 | 1.51E-08 | 11.37116 |
| GPT2 | -2.25542 | 9.268337 | -6.9668 | 1.69E-09 | 1.51E-08 | 11.37039 |
| MRPL33 | -1.06026 | 8.229443 | -6.96655 | 1.70E-09 | 1.51E-08 | 11.36936 |
| STAT2 | -1.85671 | 8.905523 | -6.96592 | 1.70E-09 | 1.52E-08 | 11.36681 |
| SOX2OT | 0.712376 | 6.926198 | 6.965525 | 1.70E-09 | 1.52E-08 | 11.36523 |
| MIR196B | 1.064554 | 6.992665 | 6.965107 | 1.71E-09 | 1.52E-08 | 11.36353 |
| OR6K2 | 1.16092 | 7.114477 | 6.963633 | 1.72E-09 | 1.53E-08 | 11.35757 |
| MYL6B | -1.04861 | 7.405635 | -6.96347 | 1.72E-09 | 1.53E-08 | 11.35692 |
| TRPT1 | -0.79542 | 7.638821 | -6.96311 | 1.72E-09 | 1.53E-08 | 11.35547 |
| GAK | -2.21704 | 9.593192 | -6.96266 | 1.72E-09 | 1.53E-08 | 11.35364 |
| SAT1 | -1.04737 | 8.232174 | -6.96241 | 1.72E-09 | 1.53E-08 | 11.35261 |
| OR4D10 | 0.777136 | 6.860781 | 6.962171 | 1.73E-09 | 1.53E-08 | 11.35165 |
| OR6C3 | 1.748476 | 7.392355 | 6.961004 | 1.73E-09 | 1.54E-08 | 11.34693 |
| CABP2 | 0.99064 | 6.988574 | 6.960651 | 1.74E-09 | 1.54E-08 | 11.3455 |
| KHNYN | 1.287652 | 9.240055 | 6.960446 | 1.74E-09 | 1.54E-08 | 11.34467 |
| LOC44031 | 1.424061 | 7.19857 | 6.959669 | 1.74E-09 | 1.55E-08 | 11.34152 |
| C19orf56 | -1.07747 | 7.823612 | -6.95748 | 1.76E-09 | 1.56E-08 | 11.33266 |
| PHACTR3 | 1.132626 | 7.296731 | 6.956619 | 1.77E-09 | 1.56E-08 | 11.32918 |
| PRODH2 | -1.36159 | 7.896266 | -6.9561 | 1.77E-09 | 1.57E-08 | 11.32708 |
| TGOLN2 | -0.86251 | 7.544833 | -6.9551 | 1.78E-09 | 1.57E-08 | 11.32304 |
| CAMK2B | 1.301461 | 7.362421 | 6.953599 | 1.79E-09 | 1.58E-08 | 11.31695 |
| ADH1A | -1.84498 | 8.928991 | -6.95061 | 1.81E-09 | 1.60E-08 | 11.30487 |
| CPA4 | 1.121364 | 7.267768 | 6.950088 | 1.81E-09 | 1.60E-08 | 11.30274 |
| LCE5A | 1.090581 | 7.03692 | 6.950027 | 1.82E-09 | 1.60E-08 | 11.3025 |

| | | | | | | |
|---|---|---|---|---|---|---|
| RPS29 | -0.83714 | 10.28612 | -6.94498 | 1.85E-09 | 1.63E-08 | 11.28208 |
| FGG | -1.71962 | 9.369802 | -6.94493 | 1.85E-09 | 1.63E-08 | 11.28188 |
| SC65 | 0.754972 | 7.078496 | 6.943497 | 1.86E-09 | 1.64E-08 | 11.27607 |
| CES4 | -1.78664 | 9.977738 | -6.94318 | 1.87E-09 | 1.64E-08 | 11.27478 |
| NHLH1 | 1.611283 | 7.446252 | 6.941976 | 1.88E-09 | 1.65E-08 | 11.26992 |
| COPS5 | -1.19516 | 8.179736 | -6.93996 | 1.89E-09 | 1.66E-08 | 11.26178 |
| RPL23AP5 | 0.757969 | 7.252896 | 6.939159 | 1.90E-09 | 1.67E-08 | 11.25852 |
| UGT2B15 | -1.78496 | 8.398426 | -6.93811 | 1.91E-09 | 1.67E-08 | 11.25427 |
| TEX14 | 1.223535 | 7.181359 | 6.937892 | 1.91E-09 | 1.67E-08 | 11.2534 |
| KIAA1045 | 1.105182 | 7.042872 | 6.937329 | 1.91E-09 | 1.68E-08 | 11.25112 |
| IQCF2 | 1.713848 | 7.262575 | 6.937323 | 1.91E-09 | 1.68E-08 | 11.2511 |
| WBSCR28 | 0.923928 | 6.846274 | 6.935264 | 1.93E-09 | 1.69E-08 | 11.24277 |
| HPN | -0.8533 | 7.379613 | -6.93125 | 1.96E-09 | 1.71E-08 | 11.22653 |
| CASC2 | 0.70249 | 6.881755 | 6.930338 | 1.97E-09 | 1.72E-08 | 11.22284 |
| TBC1D3H | 0.858372 | 7.601864 | 6.928149 | 1.99E-09 | 1.73E-08 | 11.21398 |
| DIAPH1 | -0.93078 | 8.038781 | -6.92678 | 2.00E-09 | 1.74E-08 | 11.20845 |
| PIP5K1B | 0.904642 | 7.092956 | 6.925432 | 2.01E-09 | 1.75E-08 | 11.203 |
| SLC26A9 | 0.796189 | 6.923107 | 6.920852 | 2.05E-09 | 1.78E-08 | 11.18447 |
| SIX3 | 1.336624 | 7.22178 | 6.919865 | 2.06E-09 | 1.79E-08 | 11.18048 |
| WDR49 | 0.985943 | 7.052053 | 6.911807 | 2.13E-09 | 1.85E-08 | 11.1479 |
| FOXD4 | 1.551476 | 7.764254 | 6.911291 | 2.13E-09 | 1.85E-08 | 11.14581 |
| HOXA9 | 1.385403 | 7.240223 | 6.910208 | 2.14E-09 | 1.86E-08 | 11.14143 |
| KRTAP6-3 | 1.222447 | 7.074757 | 6.908684 | 2.15E-09 | 1.87E-08 | 11.13527 |
| AADAC | -1.05832 | 7.587492 | -6.90833 | 2.16E-09 | 1.87E-08 | 11.13383 |
| CYTSA | -0.73358 | 7.560793 | -6.90697 | 2.17E-09 | 1.88E-08 | 11.12834 |
| UBE2E1 | -1.28262 | 8.223864 | -6.90566 | 2.18E-09 | 1.89E-08 | 11.12305 |
| SLC40A1 | -1.0317 | 7.725592 | -6.90269 | 2.21E-09 | 1.91E-08 | 11.11103 |
| CNIH | -0.92508 | 8.138263 | -6.90165 | 2.22E-09 | 1.92E-08 | 11.10682 |
| RCC1 | -0.92221 | 7.261847 | -6.901 | 2.22E-09 | 1.92E-08 | 11.10419 |
| LIX1 | 1.214362 | 7.301225 | 6.898383 | 2.25E-09 | 1.94E-08 | 11.09363 |
| TNMD | 0.862904 | 6.955136 | 6.897881 | 2.25E-09 | 1.94E-08 | 11.0916 |
| TMEM132 | 1.714075 | 7.626248 | 6.893901 | 2.29E-09 | 1.97E-08 | 11.07551 |
| RPL3 | -1.04882 | 9.264753 | -6.89333 | 2.29E-09 | 1.98E-08 | 11.07322 |
| ILK | -0.73531 | 7.809506 | -6.89267 | 2.30E-09 | 1.98E-08 | 11.07052 |
| TMEM66 | -1.17445 | 7.767453 | -6.88988 | 2.33E-09 | 2.00E-08 | 11.05925 |
| HRK | 0.947405 | 6.811627 | 6.889702 | 2.33E-09 | 2.00E-08 | 11.05854 |
| HIST1H4B | -0.7942 | 7.530146 | -6.88925 | 2.33E-09 | 2.01E-08 | 11.0567 |
| FAM105B | -0.87603 | 7.542313 | -6.88613 | 2.36E-09 | 2.03E-08 | 11.04412 |
| TTTY6B | 0.730894 | 6.808745 | 6.882963 | 2.39E-09 | 2.05E-08 | 11.03131 |
| LOC3999( | 1.59725 | 9.904153 | 6.882855 | 2.39E-09 | 2.05E-08 | 11.03087 |
| SRC | 1.141384 | 8.85051 | 6.880642 | 2.42E-09 | 2.07E-08 | 11.02193 |
| BLOC1S2 | 1.048377 | 7.662067 | 6.879359 | 2.43E-09 | 2.08E-08 | 11.01675 |
| FOXO4 | -1.28471 | 7.834222 | -6.879 | 2.43E-09 | 2.08E-08 | 11.01528 |
| LOC33924 | 1.250936 | 7.378493 | 6.878867 | 2.43E-09 | 2.08E-08 | 11.01476 |
| MYCBP | 0.894598 | 7.078231 | 6.876201 | 2.46E-09 | 2.10E-08 | 11.00399 |
| SNORA74. | -2.24563 | 10.2308 | -6.87505 | 2.47E-09 | 2.11E-08 | 10.99934 |
| OR6P1 | 0.722909 | 6.762795 | 6.874471 | 2.48E-09 | 2.12E-08 | 10.997 |
| CLRN1OS | 1.335524 | 7.213241 | 6.873799 | 2.49E-09 | 2.12E-08 | 10.99429 |
| KRTDAP | 0.773633 | 6.750541 | 6.870583 | 2.52E-09 | 2.15E-08 | 10.98129 |
| TNN | 1.946049 | 8.445504 | 6.87017 | 2.52E-09 | 2.15E-08 | 10.97963 |
| NDUFB5 | -1.26554 | 8.605668 | -6.86756 | 2.55E-09 | 2.17E-08 | 10.96909 |
| STAT3 | -0.72476 | 7.904424 | -6.86644 | 2.56E-09 | 2.18E-08 | 10.96457 |
| RAG1AP1 | -0.8692 | 7.85759 | -6.86068 | 2.62E-09 | 2.23E-08 | 10.94128 |
| FAM107B | -1.09425 | 8.206053 | -6.85952 | 2.64E-09 | 2.24E-08 | 10.93661 |
| SNORD57 | -1.6907 | 8.54777 | -6.85949 | 2.64E-09 | 2.24E-08 | 10.93649 |
| C8B | -1.40838 | 7.791654 | -6.85916 | 2.64E-09 | 2.24E-08 | 10.93516 |
| C3orf56 | 1.46039 | 7.261231 | 6.857915 | 2.65E-09 | 2.26E-08 | 10.93013 |
| ZNF471 | 1.369542 | 7.687729 | 6.855521 | 2.68E-09 | 2.28E-08 | 10.92046 |

| | | | | | | |
|---|---|---|---|---|---|---|
| PTCHD1 | 0.738245 | 6.786603 | 6.855001 | 2.69E-09 | 2.28E-08 | 10.91836 |
| SPATS2L | -0.87964 | 7.672363 | -6.85498 | 2.69E-09 | 2.28E-08 | 10.91828 |
| OR4C13 | 0.875121 | 6.932501 | 6.853295 | 2.70E-09 | 2.30E-08 | 10.91147 |
| ELL2 | -1.03949 | 7.788664 | -6.85194 | 2.72E-09 | 2.31E-08 | 10.90601 |
| SNORD41 | -1.42717 | 8.517564 | -6.85191 | 2.72E-09 | 2.31E-08 | 10.9059 |
| RPL35 | -1.53272 | 8.622682 | -6.85127 | 2.73E-09 | 2.31E-08 | 10.90329 |
| KRTHA5 | 0.819195 | 6.884544 | 6.851239 | 2.73E-09 | 2.31E-08 | 10.90317 |
| HSP90B1 | -1.14845 | 7.529307 | -6.84926 | 2.75E-09 | 2.33E-08 | 10.89518 |
| KRTAP23- | 1.68629 | 7.297702 | 6.848126 | 2.76E-09 | 2.34E-08 | 10.8906 |
| SNORD15 | -2.05218 | 10.52699 | -6.84756 | 2.77E-09 | 2.34E-08 | 10.88832 |
| SPRYD3 | -1.14646 | 7.718342 | -6.84742 | 2.77E-09 | 2.34E-08 | 10.88775 |
| DTNBP1 | 0.789935 | 7.621554 | 6.847097 | 2.77E-09 | 2.34E-08 | 10.88645 |
| OR6C4 | 0.827323 | 6.837105 | 6.84673 | 2.78E-09 | 2.35E-08 | 10.88497 |
| PIP4K2B | -0.75362 | 7.498696 | -6.84607 | 2.79E-09 | 2.35E-08 | 10.88232 |
| ITCH | -1.30455 | 7.896388 | -6.84097 | 2.85E-09 | 2.40E-08 | 10.8617 |
| TCTN2 | 0.976432 | 7.498999 | 6.838938 | 2.87E-09 | 2.42E-08 | 10.85351 |
| DNAJC3 | -1.22329 | 8.382987 | -6.83829 | 2.88E-09 | 2.42E-08 | 10.85089 |
| CCND1 | -1.76338 | 8.35491 | -6.83809 | 2.88E-09 | 2.42E-08 | 10.85008 |
| PLD3 | -0.9746 | 7.790401 | -6.83776 | 2.88E-09 | 2.42E-08 | 10.84874 |
| PNPO | -1.02517 | 7.584695 | -6.83689 | 2.89E-09 | 2.43E-08 | 10.84523 |
| CEACAM6 | 1.278695 | 7.276946 | 6.836425 | 2.90E-09 | 2.44E-08 | 10.84337 |
| RAET1K | 1.077436 | 7.232323 | 6.836089 | 2.90E-09 | 2.44E-08 | 10.84202 |
| NCOR2 | -0.70552 | 7.48166 | -6.83591 | 2.91E-09 | 2.44E-08 | 10.8413 |
| BCL2L15 | 0.800625 | 6.987545 | 6.835507 | 2.91E-09 | 2.44E-08 | 10.83967 |
| SDK2 | 2.18164 | 8.902537 | 6.834638 | 2.92E-09 | 2.45E-08 | 10.83616 |
| CSH2 | 1.698578 | 7.397818 | 6.833747 | 2.93E-09 | 2.45E-08 | 10.83256 |
| AAA1 | 1.001987 | 7.191564 | 6.832072 | 2.95E-09 | 2.47E-08 | 10.8258 |
| RPL11 | -1.47933 | 8.233535 | -6.83036 | 2.97E-09 | 2.49E-08 | 10.81887 |
| STAT6 | -1.28369 | 9.286531 | -6.8301 | 2.98E-09 | 2.49E-08 | 10.81785 |
| BRP44L | -1.31669 | 8.442945 | -6.82719 | 3.01E-09 | 2.52E-08 | 10.80609 |
| KPNB1 | -0.80515 | 8.192031 | -6.82702 | 3.01E-09 | 2.52E-08 | 10.8054 |
| UTP11L | -0.95309 | 7.946155 | -6.82649 | 3.02E-09 | 2.52E-08 | 10.8033 |
| LAMP1 | -1.1068 | 7.44975 | -6.8253 | 3.04E-09 | 2.53E-08 | 10.79847 |
| C11orf60 | 0.817641 | 7.075924 | 6.824546 | 3.04E-09 | 2.54E-08 | 10.79544 |
| OR2AG2 | 1.0579 | 7.093678 | 6.822842 | 3.07E-09 | 2.56E-08 | 10.78856 |
| KCNJ6 | 0.888958 | 6.953782 | 6.821939 | 3.08E-09 | 2.57E-08 | 10.78492 |
| STAG1 | -0.70954 | 7.219361 | -6.82194 | 3.08E-09 | 2.57E-08 | 10.7849 |
| LBP | -0.75623 | 6.976258 | -6.82183 | 3.08E-09 | 2.57E-08 | 10.78447 |
| PIGU | -1.5182 | 9.190471 | -6.8202 | 3.10E-09 | 2.58E-08 | 10.7779 |
| C4orf44 | 1.148796 | 7.295934 | 6.819796 | 3.10E-09 | 2.58E-08 | 10.77627 |
| CMC1 | -1.03872 | 7.355738 | -6.81856 | 3.12E-09 | 2.59E-08 | 10.77128 |
| PRIM2 | 1.085325 | 7.456142 | 6.818377 | 3.12E-09 | 2.60E-08 | 10.77054 |
| CSN3 | 2.369235 | 7.856861 | 6.818099 | 3.13E-09 | 2.60E-08 | 10.76942 |
| EEF1D | -0.70999 | 7.289339 | -6.81711 | 3.14E-09 | 2.60E-08 | 10.76544 |
| TMED2 | -1.51873 | 8.250578 | -6.81707 | 3.14E-09 | 2.60E-08 | 10.76527 |
| C9orf106 | 1.163374 | 7.373537 | 6.816844 | 3.14E-09 | 2.61E-08 | 10.76436 |
| LOC38951 | 1.245189 | 8.407771 | 6.813267 | 3.19E-09 | 2.64E-08 | 10.74993 |
| GDI1 | -0.72442 | 7.201765 | -6.81018 | 3.23E-09 | 2.67E-08 | 10.73749 |
| DEFB125 | 1.535531 | 7.468239 | 6.81006 | 3.23E-09 | 2.67E-08 | 10.73699 |
| AKR1C3 | -2.53617 | 9.102505 | -6.80843 | 3.25E-09 | 2.69E-08 | 10.73043 |
| BLVRB | -1.39548 | 8.197905 | -6.80731 | 3.27E-09 | 2.70E-08 | 10.72591 |
| CACNA2D | 1.182561 | 7.481001 | 6.807017 | 3.27E-09 | 2.70E-08 | 10.72472 |
| TM4SF19 | 0.82973 | 7.05095 | 6.806527 | 3.28E-09 | 2.70E-08 | 10.72275 |
| FLJ36701 | 0.725446 | 6.821776 | 6.805271 | 3.30E-09 | 2.72E-08 | 10.71768 |
| C4BPB | -0.88884 | 7.62508 | -6.80512 | 3.30E-09 | 2.72E-08 | 10.71706 |
| NDUFA1 | -1.19942 | 8.342129 | -6.80503 | 3.30E-09 | 2.72E-08 | 10.71671 |
| ADAR | -0.95932 | 8.308892 | -6.80459 | 3.31E-09 | 2.72E-08 | 10.71492 |
| PLB1 | 1.326088 | 7.648435 | 6.804303 | 3.31E-09 | 2.72E-08 | 10.71377 |

| | | | | | | |
|---|---|---|---|---|---|---|
| TCF23 | 0.800549 | 6.99648 | 6.803874 | 3.31E-09 | 2.73E-08 | 10.71205 |
| MPHOSPH | -1.1145 | 7.718612 | -6.80166 | 3.35E-09 | 2.75E-08 | 10.70312 |
| FLJ42102 | 1.321219 | 7.01242 | 6.80134 | 3.35E-09 | 2.75E-08 | 10.70183 |
| POLR2J2 | -0.94237 | 7.40386 | -6.79817 | 3.39E-09 | 2.78E-08 | 10.68904 |
| IKBKG | -1.53275 | 8.258406 | -6.79329 | 3.46E-09 | 2.84E-08 | 10.66937 |
| ATOX1 | -1.83796 | 10.48022 | -6.79304 | 3.47E-09 | 2.84E-08 | 10.66837 |
| CACNA2D | 1.335621 | 7.662755 | 6.792331 | 3.48E-09 | 2.85E-08 | 10.6655 |
| DYNLL2 | -1.08116 | 9.012371 | -6.7915 | 3.49E-09 | 2.85E-08 | 10.66217 |
| PRICKLE4 | -0.83273 | 7.535994 | -6.79021 | 3.51E-09 | 2.87E-08 | 10.65695 |
| SSTR2 | 0.814306 | 7.175646 | 6.789387 | 3.52E-09 | 2.88E-08 | 10.65363 |
| CCDC92 | -1.16246 | 8.263554 | -6.78756 | 3.55E-09 | 2.90E-08 | 10.64627 |
| PIGM | -0.81098 | 7.552332 | -6.78611 | 3.57E-09 | 2.91E-08 | 10.6404 |
| OVOL2 | 0.802094 | 6.86507 | 6.784957 | 3.58E-09 | 2.92E-08 | 10.63578 |
| SNHG8 | -1.30557 | 7.766311 | -6.78485 | 3.58E-09 | 2.92E-08 | 10.63535 |
| NMBR | 0.792131 | 6.865459 | 6.784666 | 3.59E-09 | 2.92E-08 | 10.6346 |
| SLAMF6 | 0.835239 | 7.492091 | 6.78374 | 3.60E-09 | 2.93E-08 | 10.63087 |
| LOC10012 | 1.034766 | 7.253918 | 6.782431 | 3.62E-09 | 2.95E-08 | 10.6256 |
| MXD4 | -1.90191 | 9.617629 | -6.78077 | 3.65E-09 | 2.97E-08 | 10.61891 |
| GPX4 | -1.69115 | 9.608441 | -6.7799 | 3.66E-09 | 2.98E-08 | 10.61539 |
| FLJ35816 | 0.945693 | 6.936234 | 6.779425 | 3.67E-09 | 2.98E-08 | 10.61348 |
| KCTD3 | -0.72804 | 7.496729 | -6.77906 | 3.67E-09 | 2.98E-08 | 10.61201 |
| C20orf26 | 1.450531 | 7.249491 | 6.777135 | 3.70E-09 | 3.00E-08 | 10.60425 |
| C16orf3 | 1.71073 | 7.576247 | 6.775818 | 3.72E-09 | 3.02E-08 | 10.59894 |
| PDXDC1 | -1.06457 | 7.963442 | -6.77546 | 3.73E-09 | 3.02E-08 | 10.59752 |
| MIR604 | 1.204482 | 8.718714 | 6.775134 | 3.73E-09 | 3.02E-08 | 10.59619 |
| UGDH | -1.07564 | 7.406225 | -6.77417 | 3.75E-09 | 3.03E-08 | 10.59229 |
| GK2 | 0.855585 | 6.817272 | 6.773939 | 3.75E-09 | 3.03E-08 | 10.59137 |
| NIPBL | -0.7216 | 7.716583 | -6.77342 | 3.76E-09 | 3.04E-08 | 10.58928 |
| PGRMC2 | -1.46656 | 7.867285 | -6.77228 | 3.77E-09 | 3.05E-08 | 10.5847 |
| NOTCH3 | -1.53887 | 8.833601 | -6.77157 | 3.79E-09 | 3.06E-08 | 10.58181 |
| CES1 | -1.72396 | 8.788145 | -6.77015 | 3.81E-09 | 3.07E-08 | 10.57612 |
| METAP2 | -1.40017 | 7.845701 | -6.76851 | 3.83E-09 | 3.09E-08 | 10.5695 |
| SUMF1 | -0.84203 | 7.342679 | -6.76793 | 3.84E-09 | 3.10E-08 | 10.56714 |
| RBM14 | -1.07064 | 7.591 | -6.76758 | 3.85E-09 | 3.10E-08 | 10.56574 |
| CNNM1 | 2.079692 | 8.374232 | 6.765401 | 3.88E-09 | 3.13E-08 | 10.55697 |
| ACRV1 | 0.880456 | 6.877458 | 6.765075 | 3.89E-09 | 3.13E-08 | 10.55565 |
| OR4D6 | 1.871984 | 7.603267 | 6.764806 | 3.89E-09 | 3.13E-08 | 10.55457 |
| RPH3A | 0.881298 | 7.009012 | 6.764004 | 3.91E-09 | 3.14E-08 | 10.55134 |
| PTGES2 | -1.60722 | 8.974742 | -6.76373 | 3.91E-09 | 3.14E-08 | 10.55023 |
| FAM116B | -1.30601 | 8.03143 | -6.76307 | 3.92E-09 | 3.15E-08 | 10.54757 |
| TEX261 | -0.72885 | 7.520293 | -6.76192 | 3.94E-09 | 3.16E-08 | 10.54296 |
| SSR4 | -0.70736 | 7.250402 | -6.76119 | 3.95E-09 | 3.17E-08 | 10.54001 |
| PSMB4 | -1.20009 | 8.298229 | -6.76066 | 3.96E-09 | 3.18E-08 | 10.53787 |
| CDKL2 | 0.789022 | 6.826186 | 6.75796 | 4.00E-09 | 3.21E-08 | 10.527 |
| POLR2H | -1.04556 | 9.419641 | -6.75705 | 4.02E-09 | 3.22E-08 | 10.52335 |
| STARD10 | -0.71864 | 7.111326 | -6.75617 | 4.03E-09 | 3.23E-08 | 10.5198 |
| CASC1 | 0.968841 | 6.926857 | 6.753705 | 4.07E-09 | 3.26E-08 | 10.50986 |
| SNORD97 | -1.98108 | 9.171298 | -6.75264 | 4.09E-09 | 3.27E-08 | 10.50556 |
| PPY | 1.300047 | 7.328971 | 6.752565 | 4.09E-09 | 3.27E-08 | 10.50526 |
| C11orf10 | -1.33366 | 10.18937 | -6.75163 | 4.11E-09 | 3.28E-08 | 10.50152 |
| CEBPB | -1.86458 | 9.989659 | -6.75094 | 4.12E-09 | 3.29E-08 | 10.49874 |
| SNORA39 | -1.92069 | 9.166451 | -6.74825 | 4.17E-09 | 3.33E-08 | 10.4879 |
| ASB18 | 1.46335 | 7.357586 | 6.747754 | 4.18E-09 | 3.33E-08 | 10.48589 |
| LHX9 | 1.069757 | 7.124538 | 6.74739 | 4.18E-09 | 3.34E-08 | 10.48442 |
| STAT1 | -1.25536 | 9.291998 | -6.74704 | 4.19E-09 | 3.34E-08 | 10.48303 |
| FANCD2 | 1.048192 | 7.437829 | 6.745382 | 4.22E-09 | 3.36E-08 | 10.47634 |
| COL4A2 | -0.90534 | 7.72017 | -6.74432 | 4.23E-09 | 3.37E-08 | 10.47208 |
| TH | 1.727272 | 7.305226 | 6.743552 | 4.25E-09 | 3.38E-08 | 10.46897 |

| | | | | | | |
|---|---|---|---|---|---|---|
| SCARNA2 | -1.51162 | 8.728162 | -6.74344 | 4.25E-09 | 3.38E-08 | 10.46854 |
| PHB2 | -0.73194 | 7.669407 | -6.74252 | 4.27E-09 | 3.39E-08 | 10.4648 |
| KRTAP10- | 1.231676 | 7.044617 | 6.742357 | 4.27E-09 | 3.39E-08 | 10.46416 |
| RGS6 | 0.72788 | 6.916218 | 6.742268 | 4.27E-09 | 3.39E-08 | 10.4638 |
| TIMP3 | -1.43693 | 8.23037 | -6.74217 | 4.27E-09 | 3.39E-08 | 10.46342 |
| WDR13 | -1.25379 | 8.36984 | -6.74158 | 4.28E-09 | 3.40E-08 | 10.46104 |
| TMEM163 | 1.14919 | 7.138491 | 6.73992 | 4.31E-09 | 3.42E-08 | 10.45435 |
| SMC1B | 1.000944 | 7.029154 | 6.738295 | 4.34E-09 | 3.44E-08 | 10.4478 |
| LRRTM1 | 0.741769 | 6.777671 | 6.736681 | 4.37E-09 | 3.46E-08 | 10.44131 |
| HSPA1A | -1.69603 | 8.09447 | -6.73495 | 4.40E-09 | 3.48E-08 | 10.43433 |
| ZNF626 | 1.011338 | 7.496381 | 6.733307 | 4.43E-09 | 3.51E-08 | 10.42772 |
| OTP | 2.851386 | 8.658451 | 6.732784 | 4.44E-09 | 3.51E-08 | 10.42562 |
| PTPLB | -1.57287 | 8.300145 | -6.73165 | 4.46E-09 | 3.53E-08 | 10.42105 |
| GK | -1.13332 | 8.306934 | -6.73093 | 4.47E-09 | 3.54E-08 | 10.41816 |
| PLOD1 | -1.28098 | 8.669493 | -6.72877 | 4.51E-09 | 3.56E-08 | 10.40948 |
| RIMS1 | 2.024328 | 7.675446 | 6.72836 | 4.52E-09 | 3.57E-08 | 10.40781 |
| SNORA57 | -2.18983 | 11.68846 | -6.72707 | 4.55E-09 | 3.59E-08 | 10.40262 |
| DDOST | -0.72506 | 7.518515 | -6.72695 | 4.55E-09 | 3.59E-08 | 10.40216 |
| CHP | -0.77407 | 7.531829 | -6.72678 | 4.55E-09 | 3.59E-08 | 10.40146 |
| EIF5A2 | 1.204525 | 7.354834 | 6.718355 | 4.71E-09 | 3.71E-08 | 10.36755 |
| C8orf55 | -1.71947 | 9.196901 | -6.71641 | 4.75E-09 | 3.73E-08 | 10.35973 |
| AURKC | 1.312584 | 7.469407 | 6.71324 | 4.81E-09 | 3.78E-08 | 10.34697 |
| C6orf108 | -1.12047 | 8.336211 | -6.71303 | 4.82E-09 | 3.78E-08 | 10.34615 |
| LOC72916 | 1.007628 | 7.11515 | 6.712963 | 4.82E-09 | 3.78E-08 | 10.34586 |
| SLC38A2 | -2.03045 | 8.642485 | -6.71223 | 4.83E-09 | 3.79E-08 | 10.34292 |
| NACA | -0.80742 | 7.379086 | -6.71183 | 4.84E-09 | 3.80E-08 | 10.3413 |
| METTL7A | -1.59527 | 8.410645 | -6.71098 | 4.86E-09 | 3.81E-08 | 10.33789 |
| ZBTB4 | -0.80296 | 8.043892 | -6.70962 | 4.88E-09 | 3.83E-08 | 10.33242 |
| PHB | -1.41471 | 8.047301 | -6.70896 | 4.90E-09 | 3.84E-08 | 10.32976 |
| ELA3A | 1.401507 | 7.121 | 6.707636 | 4.92E-09 | 3.86E-08 | 10.32443 |
| CBWD1 | -0.82949 | 7.292719 | -6.70723 | 4.93E-09 | 3.86E-08 | 10.32281 |
| KLHDC2 | -0.94064 | 7.474316 | -6.70492 | 4.98E-09 | 3.89E-08 | 10.31352 |
| SOX13 | -0.70504 | 7.710993 | -6.70373 | 5.00E-09 | 3.91E-08 | 10.30871 |
| CACNB1 | 0.776751 | 7.135183 | 6.703615 | 5.01E-09 | 3.91E-08 | 10.30826 |
| H1FNT | 1.252196 | 7.418819 | 6.703439 | 5.01E-09 | 3.91E-08 | 10.30755 |
| AKR7A2 | -1.02664 | 7.911369 | -6.70236 | 5.03E-09 | 3.93E-08 | 10.30322 |
| MYO1B | -0.84659 | 7.390185 | -6.70219 | 5.03E-09 | 3.93E-08 | 10.30252 |
| MST1R | 1.641962 | 7.517157 | 6.700903 | 5.06E-09 | 3.95E-08 | 10.29735 |
| INSL3 | 0.952469 | 6.901142 | 6.700598 | 5.07E-09 | 3.95E-08 | 10.29612 |
| XRCC6 | -1.19282 | 8.452682 | -6.70022 | 5.08E-09 | 3.95E-08 | 10.2946 |
| ILVBL | -1.20262 | 9.068966 | -6.699 | 5.10E-09 | 3.97E-08 | 10.28968 |
| SLC15A3 | -1.02789 | 7.801364 | -6.69856 | 5.11E-09 | 3.97E-08 | 10.28794 |
| C9orf114 | -1.02799 | 7.947005 | -6.6968 | 5.15E-09 | 4.00E-08 | 10.28084 |
| SF3A2 | -1.08103 | 8.120628 | -6.69603 | 5.16E-09 | 4.01E-08 | 10.27775 |
| KIAA0773 | 1.022505 | 7.132339 | 6.695057 | 5.18E-09 | 4.02E-08 | 10.27384 |
| FAM125A | -1.02408 | 7.618531 | -6.69498 | 5.19E-09 | 4.02E-08 | 10.27353 |
| MGC4432 | 0.990887 | 6.968734 | 6.69494 | 5.19E-09 | 4.02E-08 | 10.27337 |
| CCDC125 | 1.233322 | 7.592138 | 6.694864 | 5.19E-09 | 4.02E-08 | 10.27306 |
| IFITM1 | -1.67087 | 7.970491 | -6.69467 | 5.19E-09 | 4.03E-08 | 10.27227 |
| ABCB7 | -0.84228 | 7.755781 | -6.69264 | 5.24E-09 | 4.06E-08 | 10.2641 |
| SNORD84 | -1.92471 | 9.459477 | -6.69212 | 5.25E-09 | 4.07E-08 | 10.26204 |
| TPSG1 | 2.261821 | 7.81857 | 6.689296 | 5.31E-09 | 4.11E-08 | 10.25068 |
| PRKAB2 | -1.19323 | 8.030626 | -6.68843 | 5.33E-09 | 4.12E-08 | 10.2472 |
| FLJ12684 | 0.904798 | 6.83076 | 6.688293 | 5.33E-09 | 4.12E-08 | 10.24664 |
| SLC47A1 | -1.89388 | 9.296619 | -6.68767 | 5.34E-09 | 4.13E-08 | 10.24414 |
| SETDB2 | -0.81189 | 8.656587 | -6.6862 | 5.38E-09 | 4.15E-08 | 10.23822 |
| SFRS2 | -0.73705 | 7.590201 | -6.68618 | 5.38E-09 | 4.15E-08 | 10.23815 |
| ISL1 | 0.893675 | 6.918532 | 6.685824 | 5.38E-09 | 4.16E-08 | 10.23672 |

| | | | | | | |
|---|---|---|---|---|---|---|
| DNAJC8 | -2.26061 | 9.861429 | -6.68572 | 5.39E-09 | 4.16E-08 | 10.2363 |
| RFPL1S | 1.394672 | 7.482956 | 6.685534 | 5.39E-09 | 4.16E-08 | 10.23555 |
| CCDC37 | 0.879934 | 6.873608 | 6.684003 | 5.42E-09 | 4.18E-08 | 10.2294 |
| SCRIB | -1.29236 | 8.950274 | -6.68292 | 5.45E-09 | 4.20E-08 | 10.22506 |
| VRK2 | -0.78547 | 7.49709 | -6.68216 | 5.47E-09 | 4.21E-08 | 10.22199 |
| OR51S1 | 1.90273 | 7.38271 | 6.680731 | 5.50E-09 | 4.23E-08 | 10.21624 |
| HBA2 | -1.79354 | 9.58285 | -6.68064 | 5.50E-09 | 4.23E-08 | 10.21587 |
| WFIKKN2 | 1.338532 | 7.240972 | 6.679453 | 5.53E-09 | 4.25E-08 | 10.21111 |
| CHST6 | 1.959574 | 7.668637 | 6.679277 | 5.53E-09 | 4.25E-08 | 10.2104 |
| SCARB2 | -1.13795 | 7.607882 | -6.67863 | 5.55E-09 | 4.26E-08 | 10.2078 |
| AARS | -1.70916 | 10.27908 | -6.67694 | 5.58E-09 | 4.29E-08 | 10.201 |
| TXNIP | -1.40266 | 8.7903 | -6.6751 | 5.63E-09 | 4.32E-08 | 10.19363 |
| SERPINA7 | -1.54589 | 8.338437 | -6.6745 | 5.64E-09 | 4.33E-08 | 10.19119 |
| UBIAD1 | -0.8809 | 7.438143 | -6.67404 | 5.65E-09 | 4.33E-08 | 10.18937 |
| PPP1R14C | 0.80742 | 6.767531 | 6.673342 | 5.67E-09 | 4.34E-08 | 10.18655 |
| RGPD2 | 1.003514 | 7.821765 | 6.672899 | 5.68E-09 | 4.35E-08 | 10.18477 |
| MYH7B | 0.819371 | 7.043432 | 6.671935 | 5.70E-09 | 4.37E-08 | 10.1809 |
| TROVE2 | -0.75382 | 8.316316 | -6.67104 | 5.72E-09 | 4.38E-08 | 10.17729 |
| MYO18A | -1.2041 | 7.944328 | -6.66931 | 5.76E-09 | 4.41E-08 | 10.17037 |
| C17orf77 | 1.075413 | 6.870411 | 6.668014 | 5.79E-09 | 4.43E-08 | 10.16514 |
| PLSCR2 | 1.308246 | 7.216356 | 6.666422 | 5.83E-09 | 4.46E-08 | 10.15874 |
| PSPC1 | -0.79296 | 7.684076 | -6.66623 | 5.84E-09 | 4.46E-08 | 10.15796 |
| FAM83E | 1.325278 | 7.007656 | 6.663108 | 5.91E-09 | 4.51E-08 | 10.14543 |
| FOSL2 | -1.13853 | 8.175431 | -6.66308 | 5.91E-09 | 4.51E-08 | 10.1453 |
| ADIG | 1.130221 | 7.117684 | 6.662851 | 5.92E-09 | 4.51E-08 | 10.1444 |
| CD302 | -0.77338 | 7.429063 | -6.66228 | 5.93E-09 | 4.52E-08 | 10.1421 |
| SCN3B | 0.81183 | 6.870687 | 6.661635 | 5.95E-09 | 4.53E-08 | 10.13951 |
| SNRPC | -0.92854 | 8.552895 | -6.66153 | 5.95E-09 | 4.53E-08 | 10.13909 |
| NDUFA8 | -1.26849 | 8.981936 | -6.66148 | 5.95E-09 | 4.53E-08 | 10.13889 |
| MBD4 | 1.21157 | 8.990029 | 6.659656 | 5.99E-09 | 4.56E-08 | 10.13157 |
| SNX26 | -0.71027 | 7.344298 | -6.65789 | 6.04E-09 | 4.59E-08 | 10.12447 |
| FLJ43860 | 0.840173 | 6.966428 | 6.657108 | 6.06E-09 | 4.61E-08 | 10.12133 |
| ALS2CL | 1.025245 | 8.367386 | 6.652377 | 6.18E-09 | 4.69E-08 | 10.10233 |
| LOC39068 | 0.815982 | 6.762506 | 6.646854 | 6.32E-09 | 4.79E-08 | 10.08015 |
| METTL11E | 0.905854 | 6.962544 | 6.645764 | 6.35E-09 | 4.81E-08 | 10.07577 |
| DCAKD | -1.56622 | 8.669111 | -6.64547 | 6.35E-09 | 4.82E-08 | 10.07459 |
| POU4F3 | 2.372871 | 7.750833 | 6.645176 | 6.36E-09 | 4.82E-08 | 10.07341 |
| SPTAN1 | -1.12177 | 9.665955 | -6.64345 | 6.41E-09 | 4.85E-08 | 10.06646 |
| SNORD10 | -0.97729 | 7.578618 | -6.64249 | 6.43E-09 | 4.87E-08 | 10.06264 |
| UGT3A2 | 2.209047 | 7.920852 | 6.641975 | 6.45E-09 | 4.88E-08 | 10.06056 |
| GPR137 | -0.78566 | 10.83315 | -6.63901 | 6.52E-09 | 4.93E-08 | 10.04865 |
| BCL11A | 0.997708 | 7.42169 | 6.638859 | 6.53E-09 | 4.93E-08 | 10.04805 |
| OR8S1 | 1.309434 | 7.098152 | 6.63883 | 6.53E-09 | 4.93E-08 | 10.04793 |
| C17orf82 | -0.77573 | 7.412437 | -6.63726 | 6.57E-09 | 4.96E-08 | 10.04164 |
| SND1 | -0.71038 | 7.859047 | -6.63656 | 6.59E-09 | 4.97E-08 | 10.03883 |
| TUBA3E | 0.813367 | 6.937064 | 6.633924 | 6.66E-09 | 5.02E-08 | 10.02824 |
| PRB4 | 1.093834 | 7.037774 | 6.633709 | 6.67E-09 | 5.02E-08 | 10.02738 |
| P2RY11 | -0.92652 | 8.66952 | -6.63318 | 6.68E-09 | 5.03E-08 | 10.02527 |
| KRT8 | -1.04356 | 7.639115 | -6.63274 | 6.69E-09 | 5.03E-08 | 10.02347 |
| PPP1R16A | -1.6923 | 9.067577 | -6.63056 | 6.75E-09 | 5.08E-08 | 10.01472 |
| CNTD2 | 1.610159 | 7.839272 | 6.628146 | 6.82E-09 | 5.12E-08 | 10.00505 |
| IFI30 | -1.29976 | 8.152023 | -6.62625 | 6.87E-09 | 5.16E-08 | 9.997429 |
| PRNT | 0.737924 | 6.822339 | 6.625779 | 6.89E-09 | 5.16E-08 | 9.995552 |
| RPRC1 | -1.81992 | 8.69245 | -6.62553 | 6.89E-09 | 5.17E-08 | 9.994539 |
| RAP1GAP | -1.65207 | 8.449533 | -6.62341 | 6.95E-09 | 5.21E-08 | 9.986052 |
| QARS | -1.48162 | 8.948904 | -6.62297 | 6.97E-09 | 5.22E-08 | 9.984272 |
| MORF4L2 | -1.37369 | 9.028203 | -6.62251 | 6.98E-09 | 5.22E-08 | 9.982444 |
| RHPN2 | -1.08058 | 7.563632 | -6.6222 | 6.99E-09 | 5.23E-08 | 9.981174 |

| | | | | | | |
|---|---|---|---|---|---|---|
| DEFB109 | 0.775246 | 6.768189 | 6.62166 | 7.00E-09 | 5.24E-08 | 9.979022 |
| KRTAP10- | 1.505612 | 7.415521 | 6.620768 | 7.03E-09 | 5.26E-08 | 9.975446 |
| GAGE10 | 1.047436 | 7.077755 | 6.616096 | 7.17E-09 | 5.35E-08 | 9.956699 |
| TMEM40 | 1.141058 | 6.893437 | 6.615748 | 7.18E-09 | 5.36E-08 | 9.955302 |
| FBRS | -0.87433 | 8.245168 | -6.61567 | 7.18E-09 | 5.36E-08 | 9.954995 |
| CSAG3B | 1.14129 | 7.370079 | 6.615429 | 7.19E-09 | 5.36E-08 | 9.954022 |
| ARTN | 0.74796 | 6.946802 | 6.614645 | 7.21E-09 | 5.38E-08 | 9.950879 |
| SCUBE3 | 0.732651 | 6.85265 | 6.614151 | 7.22E-09 | 5.39E-08 | 9.948898 |
| AKAP6 | 0.817006 | 6.943113 | 6.612206 | 7.28E-09 | 5.43E-08 | 9.941094 |
| PHLDB1 | -0.76111 | 7.522645 | -6.61205 | 7.29E-09 | 5.43E-08 | 9.940457 |
| KRTAP5-7 | 1.020981 | 7.217458 | 6.611925 | 7.29E-09 | 5.43E-08 | 9.93997 |
| SERINC4 | 1.167636 | 7.322278 | 6.610014 | 7.35E-09 | 5.47E-08 | 9.932302 |
| UBTFL2 | 1.493138 | 7.34253 | 6.609891 | 7.35E-09 | 5.47E-08 | 9.93181 |
| GPT | -1.52397 | 8.376437 | -6.60893 | 7.38E-09 | 5.49E-08 | 9.927939 |
| KRTAP17- | 1.435918 | 7.283401 | 6.60824 | 7.40E-09 | 5.50E-08 | 9.925186 |
| LOC38843 | 1.367996 | 7.307404 | 6.60648 | 7.45E-09 | 5.54E-08 | 9.91813 |
| SCD5 | 1.395364 | 7.975466 | 6.606362 | 7.46E-09 | 5.54E-08 | 9.917654 |
| ISG20 | -1.52405 | 8.349706 | -6.60593 | 7.47E-09 | 5.55E-08 | 9.915942 |
| SNORD54 | -0.94577 | 7.331977 | -6.60459 | 7.51E-09 | 5.57E-08 | 9.910541 |
| KAT2A | -1.13868 | 7.955564 | -6.60402 | 7.53E-09 | 5.58E-08 | 9.908256 |
| UBE2T | 1.692596 | 8.356129 | 6.600345 | 7.64E-09 | 5.66E-08 | 9.893526 |
| ARL6IP5 | -1.29996 | 7.813409 | -6.59843 | 7.70E-09 | 5.70E-08 | 9.88584 |
| GMFG | -0.73555 | 7.241621 | -6.59669 | 7.76E-09 | 5.74E-08 | 9.87889 |
| TOMM22 | -1.29134 | 9.043534 | -6.59629 | 7.77E-09 | 5.75E-08 | 9.877269 |
| ANKRD9 | -1.14223 | 8.924888 | -6.59503 | 7.81E-09 | 5.78E-08 | 9.87223 |
| MAPK3 | -1.34504 | 8.97303 | -6.59366 | 7.86E-09 | 5.81E-08 | 9.866732 |
| GLYCTK | -1.06032 | 7.843974 | -6.59256 | 7.89E-09 | 5.83E-08 | 9.862324 |
| BTF3 | -1.38588 | 8.674519 | -6.59166 | 7.92E-09 | 5.85E-08 | 9.858703 |
| YWHAB | -0.83292 | 7.753891 | -6.59148 | 7.93E-09 | 5.85E-08 | 9.857998 |
| HEXB | -1.86153 | 8.750247 | -6.59086 | 7.95E-09 | 5.86E-08 | 9.855492 |
| SOX3 | 1.238301 | 7.010537 | 6.588196 | 8.03E-09 | 5.93E-08 | 9.844822 |
| C11orf76 | 0.73172 | 6.758038 | 6.586739 | 8.08E-09 | 5.96E-08 | 9.838981 |
| RPL36AL | -1.17596 | 8.005282 | -6.58517 | 8.13E-09 | 5.99E-08 | 9.83269 |
| MRPS22 | -0.89783 | 7.434628 | -6.58364 | 8.18E-09 | 6.02E-08 | 9.82657 |
| ADH1B | -1.73936 | 8.099201 | -6.58185 | 8.25E-09 | 6.07E-08 | 9.819394 |
| ITPKB | 1.001187 | 7.945824 | 6.580936 | 8.28E-09 | 6.09E-08 | 9.815724 |
| C16orf42 | -1.02866 | 7.997531 | -6.58082 | 8.28E-09 | 6.09E-08 | 9.815243 |
| MTSS1 | -1.00601 | 7.758144 | -6.5807 | 8.28E-09 | 6.09E-08 | 9.814792 |
| ABCF1 | -0.98188 | 8.360314 | -6.57706 | 8.41E-09 | 6.17E-08 | 9.8002 |
| C5orf60 | 1.023949 | 7.047195 | 6.57463 | 8.49E-09 | 6.23E-08 | 9.790458 |
| FLJ41821 | 1.312905 | 7.177115 | 6.574456 | 8.50E-09 | 6.23E-08 | 9.78976 |
| NCRNA00 | 1.015138 | 7.169637 | 6.570868 | 8.62E-09 | 6.31E-08 | 9.775383 |
| SLC25A13 | -1.06239 | 7.628581 | -6.57066 | 8.63E-09 | 6.31E-08 | 9.77454 |
| ATP6V0E1 | -0.91841 | 9.505572 | -6.56951 | 8.67E-09 | 6.34E-08 | 9.769959 |
| PUF60 | -1.50811 | 10.6792 | -6.56944 | 8.67E-09 | 6.34E-08 | 9.769664 |
| DACT1 | 0.921413 | 7.536135 | 6.569208 | 8.68E-09 | 6.35E-08 | 9.768733 |
| MYOM3 | 1.319544 | 7.347379 | 6.566276 | 8.79E-09 | 6.41E-08 | 9.756989 |
| NSMCE1 | -0.85277 | 7.336926 | -6.56578 | 8.81E-09 | 6.42E-08 | 9.755009 |
| GAL3ST3 | 1.791216 | 7.566576 | 6.564621 | 8.85E-09 | 6.45E-08 | 9.750362 |
| SCNM1 | -0.74032 | 7.118257 | -6.56443 | 8.85E-09 | 6.45E-08 | 9.749579 |
| DNLZ | -0.98258 | 7.533795 | -6.56386 | 8.88E-09 | 6.47E-08 | 9.747294 |
| HIST1H2A | 1.867871 | 8.079389 | 6.56287 | 8.91E-09 | 6.49E-08 | 9.743348 |
| FAM3A | -1.23413 | 9.363741 | -6.56115 | 8.97E-09 | 6.53E-08 | 9.736442 |
| GRHL2 | 1.679184 | 7.49593 | 6.560119 | 9.01E-09 | 6.56E-08 | 9.73233 |
| GHRL | 1.381582 | 7.738103 | 6.559616 | 9.03E-09 | 6.57E-08 | 9.730315 |
| TACO1 | -1.11371 | 7.658651 | -6.5591 | 9.05E-09 | 6.58E-08 | 9.728238 |
| F13B | -0.89152 | 7.189522 | -6.55793 | 9.09E-09 | 6.61E-08 | 9.723583 |
| IRF9 | -0.88728 | 7.767564 | -6.55542 | 9.19E-09 | 6.67E-08 | 9.71351 |

| | | | | | | |
|---|---|---|---|---|---|---|
| OSBPL5 | 1.280907 | 8.033738 | 6.55485 | 9.21E-09 | 6.68E-08 | 9.711232 |
| STX5 | -1.10341 | 7.90869 | -6.55358 | 9.26E-09 | 6.71E-08 | 9.706147 |
| PHRF1 | -1.4014 | 9.334995 | -6.55329 | 9.27E-09 | 6.71E-08 | 9.704986 |
| OBFC1 | 1.308619 | 8.095633 | 6.55305 | 9.28E-09 | 6.71E-08 | 9.704026 |
| CLDN6 | 2.257371 | 8.201436 | 6.552909 | 9.28E-09 | 6.71E-08 | 9.70346 |
| HULC | -1.65771 | 9.626097 | -6.55286 | 9.28E-09 | 6.71E-08 | 9.703254 |
| RNU4-1 | -2.72387 | 11.8172 | -6.55189 | 9.32E-09 | 6.74E-08 | 9.699373 |
| RAB21 | -0.75995 | 7.692668 | -6.55134 | 9.34E-09 | 6.75E-08 | 9.697197 |
| NDUFS5 | -1.10439 | 7.836785 | -6.55113 | 9.35E-09 | 6.75E-08 | 9.696337 |
| IGFN1 | 1.11086 | 7.131092 | 6.550967 | 9.36E-09 | 6.76E-08 | 9.695686 |
| FAM177A1 | 1.216118 | 9.968405 | 6.550562 | 9.37E-09 | 6.76E-08 | 9.694063 |
| ZBTB7C | 0.71243 | 7.125845 | 6.550322 | 9.38E-09 | 6.77E-08 | 9.693102 |
| PRKCQ | 1.065292 | 7.77846 | 6.548831 | 9.44E-09 | 6.81E-08 | 9.687134 |
| CNTNAP5 | 0.756699 | 6.912818 | 6.548693 | 9.44E-09 | 6.81E-08 | 9.686582 |
| ACSL5 | -1.13033 | 7.906919 | -6.54685 | 9.51E-09 | 6.85E-08 | 9.679195 |
| NPHP3 | 0.938619 | 7.64806 | 6.546631 | 9.52E-09 | 6.86E-08 | 9.678326 |
| RC3H2 | -0.73386 | 7.980843 | -6.54488 | 9.59E-09 | 6.90E-08 | 9.671328 |
| NDRG1 | -1.1062 | 7.795846 | -6.54178 | 9.71E-09 | 6.99E-08 | 9.658917 |
| TM4SF5 | -2.05412 | 10.01518 | -6.54154 | 9.72E-09 | 6.99E-08 | 9.657944 |
| LOC39989 | 1.2057 | 7.079424 | 6.540211 | 9.78E-09 | 7.03E-08 | 9.65263 |
| KIR2DS3 | 1.209804 | 7.040962 | 6.539513 | 9.80E-09 | 7.05E-08 | 9.649837 |
| HYAL1 | -1.15913 | 9.608464 | -6.5376 | 9.88E-09 | 7.10E-08 | 9.642188 |
| SNORA27 | -1.35462 | 7.892471 | -6.53727 | 9.89E-09 | 7.11E-08 | 9.640869 |
| KRT75 | 1.590211 | 7.227418 | 6.537136 | 9.90E-09 | 7.11E-08 | 9.640324 |
| ITGA8 | 0.844265 | 7.410711 | 6.534306 | 1.00E-08 | 7.18E-08 | 9.629001 |
| UBE2L6 | -0.87098 | 8.14518 | -6.53405 | 1.00E-08 | 7.19E-08 | 9.627976 |
| ULBP2 | 1.749051 | 7.692324 | 6.533713 | 1.00E-08 | 7.20E-08 | 9.626628 |
| SLC38A3 | -0.98874 | 7.467477 | -6.53137 | 1.01E-08 | 7.26E-08 | 9.617259 |
| RPL10A | -1.13838 | 8.718919 | -6.52942 | 1.02E-08 | 7.32E-08 | 9.609455 |
| FBXO38 | -0.83354 | 8.301057 | -6.52825 | 1.03E-08 | 7.35E-08 | 9.604782 |
| SERPIND1 | -1.50245 | 9.824411 | -6.52424 | 1.04E-08 | 7.47E-08 | 9.588722 |
| C3orf58 | -0.82828 | 7.400527 | -6.52385 | 1.05E-08 | 7.47E-08 | 9.587174 |
| REXO4 | -0.83164 | 7.755149 | -6.52317 | 1.05E-08 | 7.49E-08 | 9.584442 |
| OCIAD2 | -0.78413 | 7.440684 | -6.52259 | 1.05E-08 | 7.50E-08 | 9.582137 |
| C7orf30 | -1.72792 | 8.068758 | -6.52091 | 1.06E-08 | 7.55E-08 | 9.575393 |
| CCDC53 | -1.34019 | 7.971444 | -6.52075 | 1.06E-08 | 7.55E-08 | 9.574758 |
| MYCL1 | 0.707261 | 7.260017 | 6.519117 | 1.07E-08 | 7.59E-08 | 9.56824 |
| TUBA8 | 1.098683 | 7.312833 | 6.518972 | 1.07E-08 | 7.60E-08 | 9.567657 |
| GK5 | -1.77319 | 8.42351 | -6.51857 | 1.07E-08 | 7.61E-08 | 9.566032 |
| LOC15112 | 1.758075 | 7.75746 | 6.51622 | 1.08E-08 | 7.67E-08 | 9.556653 |
| OR6N1 | 1.47803 | 7.178121 | 6.516023 | 1.08E-08 | 7.68E-08 | 9.555866 |
| C16orf48 | -1.75498 | 9.013213 | -6.51428 | 1.09E-08 | 7.72E-08 | 9.548904 |
| CALM3 | -1.58074 | 9.052682 | -6.51354 | 1.09E-08 | 7.74E-08 | 9.545919 |
| DNAJC15 | -0.98941 | 7.526083 | -6.51301 | 1.09E-08 | 7.75E-08 | 9.543804 |
| SAR1B | 0.809047 | 8.874662 | 6.512486 | 1.09E-08 | 7.77E-08 | 9.541719 |
| DNAI1 | 0.929178 | 7.186001 | 6.511991 | 1.10E-08 | 7.78E-08 | 9.539741 |
| C1QB | -1.05195 | 8.243651 | -6.5118 | 1.10E-08 | 7.78E-08 | 9.538997 |
| FCRL1 | 0.866824 | 7.171476 | 6.509663 | 1.11E-08 | 7.84E-08 | 9.530435 |
| SUSD2 | -1.57403 | 8.233718 | -6.50869 | 1.11E-08 | 7.87E-08 | 9.526553 |
| PGA3 | 0.723535 | 6.805487 | 6.508673 | 1.11E-08 | 7.87E-08 | 9.526474 |
| SNORD10 | -1.72356 | 9.664906 | -6.50667 | 1.12E-08 | 7.93E-08 | 9.518462 |
| C12orf53 | 2.072212 | 7.856492 | 6.5058 | 1.13E-08 | 7.95E-08 | 9.514991 |
| FUT5 | 0.94218 | 7.010952 | 6.505083 | 1.13E-08 | 7.97E-08 | 9.512125 |
| ST7 | 0.73351 | 8.653567 | 6.505035 | 1.13E-08 | 7.97E-08 | 9.511931 |
| TAF10 | -1.35437 | 8.06203 | -6.50424 | 1.13E-08 | 7.99E-08 | 9.508771 |
| CCDC149 | 0.967186 | 7.232477 | 6.503705 | 1.13E-08 | 8.01E-08 | 9.506615 |
| IMP3 | -0.72153 | 7.688077 | -6.50361 | 1.14E-08 | 8.01E-08 | 9.506254 |
| IRAK1 | -1.14312 | 8.104455 | -6.50344 | 1.14E-08 | 8.01E-08 | 9.505545 |

| | | | | | | |
|---|---|---|---|---|---|---|
| HLA-DRB3 | -1.1903 | 7.593848 | -6.50242 | 1.14E-08 | 8.04E-08 | 9.501486 |
| PLEKHG6 | 0.842151 | 7.309103 | 6.501993 | 1.14E-08 | 8.05E-08 | 9.499771 |
| ZCCHC5 | 0.914453 | 6.957662 | 6.500757 | 1.15E-08 | 8.09E-08 | 9.494832 |
| ASB16 | 1.08693 | 7.324529 | 6.500379 | 1.15E-08 | 8.10E-08 | 9.493319 |
| GNPAT | -1.35131 | 9.169864 | -6.49976 | 1.15E-08 | 8.11E-08 | 9.490828 |
| FOXI3 | 0.84757 | 7.085567 | 6.499746 | 1.15E-08 | 8.11E-08 | 9.490792 |
| NRIP3 | 0.833649 | 6.856208 | 6.497027 | 1.17E-08 | 8.19E-08 | 9.479925 |
| RPS20 | -0.90517 | 7.500376 | -6.49702 | 1.17E-08 | 8.19E-08 | 9.479882 |
| GJB3 | 1.082934 | 7.0935 | 6.496354 | 1.17E-08 | 8.21E-08 | 9.477232 |
| ZFAND5 | -1.86516 | 9.752177 | -6.49508 | 1.18E-08 | 8.25E-08 | 9.47216 |
| TMEFF2 | 1.648557 | 7.390965 | 6.494258 | 1.18E-08 | 8.28E-08 | 9.468856 |
| ACAA1 | -1.70384 | 8.439367 | -6.49356 | 1.18E-08 | 8.30E-08 | 9.466081 |
| ITPR2 | -0.96444 | 7.867525 | -6.49316 | 1.18E-08 | 8.31E-08 | 9.464474 |
| CXCL2 | -1.18611 | 7.719904 | -6.48994 | 1.20E-08 | 8.41E-08 | 9.451593 |
| HNRNPK | -1.47689 | 8.980214 | -6.48821 | 1.21E-08 | 8.47E-08 | 9.444703 |
| LRRIQ1 | 0.779544 | 6.790871 | 6.485372 | 1.22E-08 | 8.55E-08 | 9.433354 |
| FLAD1 | -0.7083 | 8.274407 | -6.48516 | 1.22E-08 | 8.56E-08 | 9.432494 |
| APC2 | 2.484672 | 8.700353 | 6.484764 | 1.23E-08 | 8.57E-08 | 9.430925 |
| POLR2J3 | -0.84305 | 7.5123 | -6.48065 | 1.25E-08 | 8.71E-08 | 9.414492 |
| LCN2 | -1.76646 | 7.930683 | -6.48005 | 1.25E-08 | 8.73E-08 | 9.412108 |
| C1orf69 | 0.94068 | 7.369019 | 6.478695 | 1.26E-08 | 8.77E-08 | 9.406685 |
| LYZ | -2.00313 | 8.70906 | -6.47865 | 1.26E-08 | 8.77E-08 | 9.406487 |
| TMCO2 | 0.720725 | 6.836689 | 6.477067 | 1.27E-08 | 8.83E-08 | 9.400184 |
| ZFP91 | -0.82085 | 7.336055 | -6.47706 | 1.27E-08 | 8.83E-08 | 9.400139 |
| DEK | -0.82042 | 7.725471 | -6.47625 | 1.27E-08 | 8.85E-08 | 9.396926 |
| PTPN13 | 0.755667 | 7.073643 | 6.476168 | 1.27E-08 | 8.85E-08 | 9.396593 |
| GDAP1L1 | 1.667115 | 7.800517 | 6.473329 | 1.28E-08 | 8.94E-08 | 9.385252 |
| SULT2B1 | 1.016854 | 7.171419 | 6.473301 | 1.28E-08 | 8.94E-08 | 9.385143 |
| CRISPLD1 | 0.848073 | 6.871913 | 6.472351 | 1.29E-08 | 8.97E-08 | 9.38135 |
| IFI35 | -1.25435 | 8.500507 | -6.47136 | 1.30E-08 | 9.00E-08 | 9.377402 |
| SEPHS2 | -1.23797 | 7.900346 | -6.4704 | 1.30E-08 | 9.03E-08 | 9.373563 |
| EIF3B | -1.07303 | 8.865924 | -6.4695 | 1.30E-08 | 9.06E-08 | 9.369959 |
| RBM35A | 1.236781 | 7.274265 | 6.469422 | 1.31E-08 | 9.06E-08 | 9.369655 |
| MTPAP | 0.873107 | 7.293804 | 6.468382 | 1.31E-08 | 9.09E-08 | 9.3655 |
| SGOL1 | 0.805207 | 7.507402 | 6.467865 | 1.31E-08 | 9.11E-08 | 9.363438 |
| SNORD93 | -1.69539 | 8.892894 | -6.46606 | 1.32E-08 | 9.17E-08 | 9.356239 |
| RNU6ATA | -1.19536 | 8.608293 | -6.46438 | 1.33E-08 | 9.23E-08 | 9.34953 |
| VAMP8 | -0.87005 | 7.639945 | -6.46354 | 1.34E-08 | 9.26E-08 | 9.346163 |
| CER1 | 0.8954 | 6.961396 | 6.462831 | 1.34E-08 | 9.28E-08 | 9.343342 |
| TRAF3IP3 | 1.695763 | 7.916454 | 6.462011 | 1.35E-08 | 9.30E-08 | 9.340069 |
| SNORA71 | -1.0616 | 7.555358 | -6.45998 | 1.36E-08 | 9.37E-08 | 9.331964 |
| GBA | -1.25397 | 7.771871 | -6.45851 | 1.36E-08 | 9.43E-08 | 9.32609 |
| LY6D | 1.138946 | 7.001941 | 6.457903 | 1.37E-08 | 9.45E-08 | 9.323672 |
| CBS | -2.26696 | 10.647 | -6.45724 | 1.37E-08 | 9.47E-08 | 9.321029 |
| DHRS3 | -0.7842 | 7.543163 | -6.45669 | 1.37E-08 | 9.48E-08 | 9.318813 |
| SPRYD5 | 1.477841 | 7.342252 | 6.454213 | 1.39E-08 | 9.57E-08 | 9.308946 |
| TCTE3 | 1.326898 | 7.236092 | 6.453612 | 1.39E-08 | 9.59E-08 | 9.306546 |
| APEX2 | -0.71562 | 7.840765 | -6.4529 | 1.40E-08 | 9.62E-08 | 9.303715 |
| APLP2 | -0.93438 | 9.709845 | -6.4506 | 1.41E-08 | 9.70E-08 | 9.294523 |
| SLC25A1 | -0.95987 | 8.376257 | -6.44989 | 1.41E-08 | 9.72E-08 | 9.291682 |
| PLG | -1.98714 | 9.441698 | -6.44977 | 1.41E-08 | 9.72E-08 | 9.291197 |
| TMEM38B | -0.81158 | 7.651468 | -6.44908 | 1.42E-08 | 9.74E-08 | 9.28845 |
| CART1 | 1.021206 | 7.015444 | 6.448901 | 1.42E-08 | 9.75E-08 | 9.287748 |
| GSTK1 | -1.19624 | 8.533116 | -6.44882 | 1.42E-08 | 9.75E-08 | 9.287406 |
| RPA2 | -0.75698 | 7.516019 | -6.44805 | 1.42E-08 | 9.77E-08 | 9.284373 |
| SCOC | -0.71542 | 7.193443 | -6.44555 | 1.44E-08 | 9.86E-08 | 9.274363 |
| ETS1 | -0.79436 | 7.396507 | -6.44504 | 1.44E-08 | 9.88E-08 | 9.272344 |
| RNU5A | -2.40695 | 10.59162 | -6.44493 | 1.44E-08 | 9.88E-08 | 9.27189 |

| | | | | | | |
|---|---|---|---|---|---|---|
| C8G | -1.20658 | 8.800169 | -6.44358 | 1.45E-08 | 9.93E-08 | 9.266511 |
| TDO2 | -1.13254 | 7.566997 | -6.44346 | 1.45E-08 | 9.93E-08 | 9.266049 |
| HIST1H2A | -1.00331 | 8.04852 | -6.4423 | 1.46E-08 | 9.97E-08 | 9.261424 |
| RPL27 | -1.11171 | 11.8044 | -6.4422 | 1.46E-08 | 9.97E-08 | 9.261025 |
| FRG1 | -1.23526 | 8.257541 | -6.44158 | 1.46E-08 | 9.99E-08 | 9.258531 |
| GARNL4 | 0.770634 | 7.23166 | 6.441161 | 1.46E-08 | 1.00E-07 | 9.256871 |
| ALKBH5 | -1.31045 | 10.08834 | -6.43975 | 1.47E-08 | 1.01E-07 | 9.251228 |
| BRF2 | 0.963197 | 7.533533 | 6.437832 | 1.48E-08 | 1.01E-07 | 9.243594 |
| CDC40 | -0.83303 | 7.623058 | -6.43686 | 1.49E-08 | 1.02E-07 | 9.239719 |
| OR4D11 | 0.943766 | 6.922162 | 6.435486 | 1.50E-08 | 1.02E-07 | 9.234238 |
| TMEM14A | -0.80504 | 7.201132 | -6.43364 | 1.51E-08 | 1.03E-07 | 9.226875 |
| SNORD91 | -1.93082 | 9.314183 | -6.43256 | 1.52E-08 | 1.03E-07 | 9.222575 |
| CSN2 | 1.133615 | 7.016459 | 6.431485 | 1.52E-08 | 1.04E-07 | 9.218282 |
| TTYH2 | 0.709404 | 7.359969 | 6.430351 | 1.53E-08 | 1.04E-07 | 9.213762 |
| ANGPTL3 | -1.81716 | 8.867752 | -6.42807 | 1.55E-08 | 1.05E-07 | 9.204669 |
| C19orf6 | -1.11717 | 8.605296 | -6.427 | 1.55E-08 | 1.05E-07 | 9.200413 |
| SNORD31 | -1.89206 | 9.256717 | -6.42391 | 1.57E-08 | 1.07E-07 | 9.188068 |
| MICALCL | 0.955883 | 7.566532 | 6.423338 | 1.58E-08 | 1.07E-07 | 9.185802 |
| ZHX1 | -1.10795 | 8.364527 | -6.42133 | 1.59E-08 | 1.08E-07 | 9.177808 |
| SAFB2 | -0.77916 | 8.14112 | -6.4209 | 1.59E-08 | 1.08E-07 | 9.17609 |
| SPINK5 | 1.489766 | 7.907181 | 6.420824 | 1.59E-08 | 1.08E-07 | 9.17578 |
| SCARNA1( | -0.77807 | 7.444538 | -6.41965 | 1.60E-08 | 1.08E-07 | 9.171096 |
| FGF17 | 1.217917 | 7.546145 | 6.419467 | 1.60E-08 | 1.08E-07 | 9.170373 |
| HIF1A | -0.7797 | 7.578848 | -6.41938 | 1.60E-08 | 1.08E-07 | 9.170013 |
| ELOVL5 | -0.76969 | 7.28832 | -6.41779 | 1.61E-08 | 1.09E-07 | 9.163702 |
| LOC72827 | 1.45866 | 7.221257 | 6.417222 | 1.61E-08 | 1.09E-07 | 9.161424 |
| MRFAP1 | -1.53248 | 8.970815 | -6.41319 | 1.64E-08 | 1.11E-07 | 9.14537 |
| DUSP21 | 1.125296 | 6.989368 | 6.412351 | 1.65E-08 | 1.11E-07 | 9.142015 |
| GGCX | -0.87472 | 7.518467 | -6.41231 | 1.65E-08 | 1.11E-07 | 9.141847 |
| SNTB1 | -1.00405 | 7.576189 | -6.41074 | 1.66E-08 | 1.12E-07 | 9.135581 |
| ERVWE1 | 0.879323 | 6.968888 | 6.410177 | 1.66E-08 | 1.12E-07 | 9.133351 |
| CAB39 | -0.891 | 8.081322 | -6.41009 | 1.66E-08 | 1.12E-07 | 9.132992 |
| JMJD1C | -0.75148 | 7.555673 | -6.40919 | 1.67E-08 | 1.12E-07 | 9.129436 |
| RGS7 | 0.768387 | 6.884193 | 6.408001 | 1.68E-08 | 1.13E-07 | 9.124681 |
| HOTAIR | 1.287055 | 7.15481 | 6.405367 | 1.69E-08 | 1.14E-07 | 9.11419 |
| PUM1 | -0.85064 | 9.110007 | -6.40501 | 1.70E-08 | 1.14E-07 | 9.112775 |
| DTX2 | -1.19775 | 7.968915 | -6.40434 | 1.70E-08 | 1.14E-07 | 9.110088 |
| OTOL1 | 2.226656 | 7.670241 | 6.403692 | 1.71E-08 | 1.15E-07 | 9.107518 |
| KRTAP4-4 | 0.851982 | 6.830429 | 6.403342 | 1.71E-08 | 1.15E-07 | 9.106124 |
| EIF4A2 | -1.66027 | 8.347635 | -6.40261 | 1.71E-08 | 1.15E-07 | 9.103187 |
| NCBP1 | -0.90923 | 8.342829 | -6.40193 | 1.72E-08 | 1.15E-07 | 9.100493 |
| YIPF7 | 1.107948 | 6.953255 | 6.40098 | 1.73E-08 | 1.16E-07 | 9.096713 |
| LOC38785 | 0.953289 | 6.954821 | 6.400641 | 1.73E-08 | 1.16E-07 | 9.095363 |
| NFKBIA | -1.2628 | 10.15267 | -6.39984 | 1.73E-08 | 1.16E-07 | 9.092156 |
| SQSTM1 | -1.61041 | 10.26242 | -6.39958 | 1.74E-08 | 1.16E-07 | 9.091139 |
| IQCE | 0.995471 | 8.005102 | 6.398546 | 1.74E-08 | 1.17E-07 | 9.087018 |
| PDIA6 | -1.06721 | 8.099685 | -6.39789 | 1.75E-08 | 1.17E-07 | 9.08439 |
| PRM2 | 0.978973 | 6.91959 | 6.397817 | 1.75E-08 | 1.17E-07 | 9.084117 |
| NTF4 | 0.771862 | 6.959206 | 6.396291 | 1.76E-08 | 1.17E-07 | 9.078039 |
| FBN2 | 0.765838 | 7.007359 | 6.394838 | 1.77E-08 | 1.18E-07 | 9.072253 |
| LOC64219 | -0.79586 | 7.62205 | -6.39474 | 1.77E-08 | 1.18E-07 | 9.071862 |
| LOC64620 | -1.16703 | 9.223709 | -6.3945 | 1.77E-08 | 1.18E-07 | 9.070896 |
| RAD23A | -0.89534 | 7.620388 | -6.39362 | 1.78E-08 | 1.18E-07 | 9.067384 |
| C8orf33 | 1.271636 | 8.412353 | 6.3936 | 1.78E-08 | 1.18E-07 | 9.067322 |
| CAPN11 | 1.766946 | 8.784179 | 6.392209 | 1.79E-08 | 1.19E-07 | 9.061786 |
| SEZ6L | 2.256114 | 8.366403 | 6.391164 | 1.80E-08 | 1.19E-07 | 9.057626 |
| MPP2 | 0.984735 | 7.231831 | 6.388731 | 1.81E-08 | 1.20E-07 | 9.047936 |
| FAM65C | 2.0236 | 8.956759 | 6.388673 | 1.81E-08 | 1.20E-07 | 9.047708 |

| | | | | | | |
|---|---|---|---|---|---|---|
| HMGCS1 | -1.56308 | 8.10138 | -6.38862 | 1.81E-08 | 1.20E-07 | 9.047479 |
| LPCAT3 | -0.7981 | 8.23953 | -6.38837 | 1.82E-08 | 1.21E-07 | 9.046515 |
| MYL6 | -1.51694 | 10.66809 | -6.38758 | 1.82E-08 | 1.21E-07 | 9.043357 |
| LOC38869 | 0.750597 | 7.166136 | 6.387562 | 1.82E-08 | 1.21E-07 | 9.043283 |
| APCDD1L | 1.001615 | 7.438671 | 6.387477 | 1.82E-08 | 1.21E-07 | 9.042943 |
| LOC39239 | 1.194004 | 7.207203 | 6.384385 | 1.85E-08 | 1.22E-07 | 9.030635 |
| IL8RB | 0.824216 | 7.267779 | 6.382786 | 1.86E-08 | 1.23E-07 | 9.024272 |
| GIYD2 | -0.97722 | 7.461812 | -6.38272 | 1.86E-08 | 1.23E-07 | 9.024007 |
| LPAR4 | 0.980548 | 7.178078 | 6.382696 | 1.86E-08 | 1.23E-07 | 9.023915 |
| RFX2 | 1.517593 | 8.102147 | 6.378831 | 1.89E-08 | 1.25E-07 | 9.00853 |
| ASB14 | 0.792021 | 6.866491 | 6.378774 | 1.89E-08 | 1.25E-07 | 9.008302 |
| UGT1A1 | -1.46729 | 8.965594 | -6.37785 | 1.90E-08 | 1.25E-07 | 9.004644 |
| TST | -1.6215 | 7.997745 | -6.37755 | 1.90E-08 | 1.25E-07 | 9.003415 |
| IL6ST | -0.83977 | 7.695857 | -6.37585 | 1.91E-08 | 1.26E-07 | 8.996686 |
| ACAP2 | -0.84935 | 7.68418 | -6.37464 | 1.92E-08 | 1.27E-07 | 8.991866 |
| TNNI3K | 0.890202 | 6.932819 | 6.374363 | 1.92E-08 | 1.27E-07 | 8.99075 |
| RPS12 | -1.29072 | 9.853593 | -6.37319 | 1.93E-08 | 1.27E-07 | 8.986094 |
| SPHKAP | 0.78207 | 6.849026 | 6.372193 | 1.94E-08 | 1.28E-07 | 8.982117 |
| TJAP1 | -1.60194 | 8.163309 | -6.37168 | 1.94E-08 | 1.28E-07 | 8.980082 |
| MAP4K3 | -0.81537 | 7.415662 | -6.37062 | 1.95E-08 | 1.28E-07 | 8.975861 |
| GPR81 | 0.864545 | 7.001274 | 6.370479 | 1.95E-08 | 1.28E-07 | 8.975295 |
| MCCC1 | -0.91583 | 8.031002 | -6.36805 | 1.97E-08 | 1.30E-07 | 8.96562 |
| PDGFRB | -0.98202 | 7.925964 | -6.36796 | 1.97E-08 | 1.30E-07 | 8.96527 |
| LRRN2 | 0.903008 | 7.492256 | 6.36716 | 1.98E-08 | 1.30E-07 | 8.962092 |
| ERO1L | -0.82311 | 7.250345 | -6.36692 | 1.98E-08 | 1.30E-07 | 8.961147 |
| TEKT2 | 1.400313 | 7.330927 | 6.364005 | 2.01E-08 | 1.32E-07 | 8.949542 |
| SDCBP | -1.11203 | 8.421434 | -6.36123 | 2.03E-08 | 1.33E-07 | 8.938511 |
| LSM4 | -1.31408 | 8.68588 | -6.36088 | 2.03E-08 | 1.33E-07 | 8.937097 |
| C12orf52 | -0.70562 | 7.269836 | -6.36033 | 2.04E-08 | 1.33E-07 | 8.934939 |
| AHSG | -2.01591 | 9.33653 | -6.3603 | 2.04E-08 | 1.33E-07 | 8.934806 |
| P2RX2 | 0.911139 | 7.090943 | 6.359711 | 2.04E-08 | 1.33E-07 | 8.932464 |
| C10orf10 | -1.0103 | 7.744229 | -6.35952 | 2.04E-08 | 1.33E-07 | 8.931711 |
| ACTN4 | -0.8161 | 10.4886 | -6.35755 | 2.06E-08 | 1.34E-07 | 8.923864 |
| HAP1 | 1.058772 | 7.166109 | 6.357449 | 2.06E-08 | 1.34E-07 | 8.92347 |
| RNASE9 | 0.765783 | 6.801422 | 6.356121 | 2.07E-08 | 1.35E-07 | 8.918187 |
| TAT | -2.01542 | 9.124842 | -6.35579 | 2.07E-08 | 1.35E-07 | 8.916875 |
| PPP2R4 | -0.8728 | 8.652805 | -6.35536 | 2.08E-08 | 1.35E-07 | 8.915156 |
| RNU105A | -1.71252 | 11.50747 | -6.35522 | 2.08E-08 | 1.35E-07 | 8.914608 |
| LDHA | -1.29093 | 8.3948 | -6.35462 | 2.08E-08 | 1.36E-07 | 8.912236 |
| MYOZ1 | 1.641719 | 8.059242 | 6.354402 | 2.09E-08 | 1.36E-07 | 8.911353 |
| SNORA71 | -1.3884 | 8.269913 | -6.3541 | 2.09E-08 | 1.36E-07 | 8.910145 |
| NFX1 | -0.86703 | 8.170542 | -6.35395 | 2.09E-08 | 1.36E-07 | 8.909568 |
| LTBR | -0.90255 | 7.665098 | -6.35291 | 2.10E-08 | 1.36E-07 | 8.905417 |
| TMEM143 | 0.923395 | 8.464988 | 6.352595 | 2.10E-08 | 1.36E-07 | 8.904167 |
| OR6W1P | 0.876691 | 6.928512 | 6.352469 | 2.10E-08 | 1.37E-07 | 8.903667 |
| PTTG1IP | -0.99156 | 7.860604 | -6.34942 | 2.13E-08 | 1.38E-07 | 8.89156 |
| PGAM5 | 1.357838 | 7.944468 | 6.348271 | 2.14E-08 | 1.39E-07 | 8.886978 |
| PYHIN1 | 0.980103 | 7.584966 | 6.347246 | 2.15E-08 | 1.39E-07 | 8.882904 |
| SNORD62 | -1.73607 | 9.351275 | -6.34636 | 2.15E-08 | 1.40E-07 | 8.879391 |
| ENOSF1 | -0.77657 | 7.349139 | -6.34599 | 2.16E-08 | 1.40E-07 | 8.877928 |
| CLEC4G | 1.822809 | 8.601013 | 6.345507 | 2.16E-08 | 1.40E-07 | 8.875991 |
| ATP5C1 | -0.79923 | 8.096991 | -6.34517 | 2.16E-08 | 1.40E-07 | 8.874636 |
| SNORA79 | -0.80448 | 7.340055 | -6.34505 | 2.17E-08 | 1.40E-07 | 8.874181 |
| C14orf112 | -1.08103 | 8.561849 | -6.34367 | 2.18E-08 | 1.41E-07 | 8.868689 |
| ZNF358 | -1.10587 | 7.941759 | -6.34345 | 2.18E-08 | 1.41E-07 | 8.867801 |
| DEGS1 | -1.30981 | 8.030323 | -6.34088 | 2.20E-08 | 1.42E-07 | 8.857618 |
| NDUFAB1 | -1.54512 | 8.901917 | -6.33698 | 2.24E-08 | 1.45E-07 | 8.842122 |
| C19orf30 | 1.389862 | 7.305424 | 6.335935 | 2.25E-08 | 1.45E-07 | 8.83795 |

| | | | | | | |
|---|---|---|---|---|---|---|
| CTLA4 | 0.884106 | 7.540918 | 6.335245 | 2.25E-08 | 1.45E-07 | 8.83521 |
| MYOCD | 1.862354 | 8.110486 | 6.331284 | 2.29E-08 | 1.48E-07 | 8.819474 |
| PTGR1 | -1.2357 | 8.795328 | -6.32973 | 2.31E-08 | 1.49E-07 | 8.813287 |
| CCT8 | -1.5738 | 9.13461 | -6.32837 | 2.32E-08 | 1.49E-07 | 8.807892 |
| KIR2DL4 | 0.882065 | 6.905246 | 6.32643 | 2.34E-08 | 1.50E-07 | 8.80019 |
| SLC35B1 | -0.72424 | 7.704071 | -6.32577 | 2.34E-08 | 1.51E-07 | 8.797551 |
| KRT84 | 1.077244 | 7.04678 | 6.325681 | 2.34E-08 | 1.51E-07 | 8.797218 |
| C14orf119 | 0.74192 | 7.072515 | 6.319951 | 2.40E-08 | 1.54E-07 | 8.774462 |
| VCAM1 | -1.17441 | 7.716131 | -6.31899 | 2.41E-08 | 1.55E-07 | 8.770632 |
| MPHOSPH | -0.91875 | 8.282696 | -6.31852 | 2.41E-08 | 1.55E-07 | 8.768771 |
| MIR614 | 1.069893 | 6.983648 | 6.317955 | 2.42E-08 | 1.55E-07 | 8.766533 |
| RGS20 | 1.216141 | 7.101462 | 6.317793 | 2.42E-08 | 1.55E-07 | 8.765893 |
| SEMA4F | 1.41313 | 7.869968 | 6.317433 | 2.42E-08 | 1.55E-07 | 8.764461 |
| CAMK2A | 0.991365 | 6.972747 | 6.317387 | 2.42E-08 | 1.55E-07 | 8.764282 |
| CCNL1 | -0.75411 | 8.084844 | -6.31737 | 2.42E-08 | 1.55E-07 | 8.764214 |
| SNORA11 | -1.63833 | 8.588824 | -6.31733 | 2.42E-08 | 1.55E-07 | 8.764059 |
| NT5C2 | -0.96863 | 7.447264 | -6.31695 | 2.43E-08 | 1.55E-07 | 8.762561 |
| TSPAN9 | -1.71279 | 9.677393 | -6.31666 | 2.43E-08 | 1.55E-07 | 8.761403 |
| LRRC3B | 1.141343 | 7.273913 | 6.316625 | 2.43E-08 | 1.55E-07 | 8.761254 |
| TPM1 | -1.20734 | 8.136221 | -6.31646 | 2.43E-08 | 1.56E-07 | 8.760587 |
| CCDC87 | 1.401415 | 7.57533 | 6.31502 | 2.45E-08 | 1.56E-07 | 8.75488 |
| ZMAT3 | 0.703865 | 7.970027 | 6.314325 | 2.45E-08 | 1.57E-07 | 8.752125 |
| ITIH1 | -1.38684 | 8.480931 | -6.31257 | 2.47E-08 | 1.58E-07 | 8.745168 |
| C20orf114 | 0.760293 | 6.876118 | 6.312082 | 2.48E-08 | 1.58E-07 | 8.74322 |
| NRBP1 | -0.8204 | 7.591474 | -6.31171 | 2.48E-08 | 1.58E-07 | 8.741745 |
| CRY1 | -0.7868 | 7.71539 | -6.31167 | 2.48E-08 | 1.58E-07 | 8.74158 |
| ATP9A | -1.41624 | 9.551486 | -6.31135 | 2.48E-08 | 1.58E-07 | 8.740299 |
| OR8D1 | 1.372531 | 7.187209 | 6.310675 | 2.49E-08 | 1.59E-07 | 8.737634 |
| PPP1R14A | -2.04098 | 10.72971 | -6.31033 | 2.49E-08 | 1.59E-07 | 8.736283 |
| PRKACA | -0.77066 | 7.818141 | -6.30971 | 2.50E-08 | 1.59E-07 | 8.73382 |
| SLC9A3R1 | -0.70355 | 7.23049 | -6.30782 | 2.52E-08 | 1.60E-07 | 8.726299 |
| PNLIPRP3 | 0.730072 | 6.779582 | 6.307584 | 2.52E-08 | 1.60E-07 | 8.725364 |
| GSDMD | -1.82831 | 9.959214 | -6.30686 | 2.53E-08 | 1.61E-07 | 8.722486 |
| SLC39A1 | -1.05201 | 8.205754 | -6.30633 | 2.53E-08 | 1.61E-07 | 8.720385 |
| PLA2G2F | 0.77687 | 6.843896 | 6.305552 | 2.54E-08 | 1.62E-07 | 8.717299 |
| ZBTB20 | -0.9935 | 8.221148 | -6.30546 | 2.54E-08 | 1.62E-07 | 8.71693 |
| SEPP1 | -1.95022 | 8.628281 | -6.3038 | 2.56E-08 | 1.63E-07 | 8.710335 |
| SNORA41 | -1.76349 | 9.57332 | -6.30339 | 2.57E-08 | 1.63E-07 | 8.708729 |
| ADH1C | -1.67966 | 8.436691 | -6.30329 | 2.57E-08 | 1.63E-07 | 8.708342 |
| PIWIL2 | 0.892844 | 7.098772 | 6.301431 | 2.59E-08 | 1.64E-07 | 8.700947 |
| RNF168 | -0.84577 | 7.882834 | -6.30054 | 2.60E-08 | 1.64E-07 | 8.697415 |
| SAA4 | -1.12357 | 7.325101 | -6.29954 | 2.61E-08 | 1.65E-07 | 8.693462 |
| CPZ | 0.758303 | 7.023329 | 6.299008 | 2.61E-08 | 1.65E-07 | 8.691331 |
| C10orf113 | 1.857093 | 7.498984 | 6.295965 | 2.64E-08 | 1.67E-07 | 8.67926 |
| CTSL1 | -0.86335 | 8.120107 | -6.29585 | 2.64E-08 | 1.67E-07 | 8.678817 |
| GPRC5A | 1.219919 | 7.577079 | 6.294129 | 2.66E-08 | 1.68E-07 | 8.671974 |
| OR4K2 | 1.18102 | 7.137444 | 6.293819 | 2.67E-08 | 1.68E-07 | 8.670747 |
| DDX17 | -1.139 | 9.177142 | -6.29195 | 2.69E-08 | 1.69E-07 | 8.663324 |
| HNRPUL1 | -1.1372 | 8.443906 | -6.29191 | 2.69E-08 | 1.69E-07 | 8.663156 |
| RTN4 | -0.99027 | 8.555883 | -6.29134 | 2.69E-08 | 1.70E-07 | 8.660918 |
| GPR63 | 1.622706 | 7.445654 | 6.291242 | 2.69E-08 | 1.70E-07 | 8.660526 |
| LYG2 | 0.748942 | 6.912054 | 6.289787 | 2.71E-08 | 1.71E-07 | 8.654755 |
| OR4D2 | 0.84671 | 6.960899 | 6.288118 | 2.73E-08 | 1.72E-07 | 8.648135 |
| OR13J1 | 2.237483 | 7.87086 | 6.287985 | 2.73E-08 | 1.72E-07 | 8.647606 |
| FCRLA | 0.856293 | 7.14695 | 6.287297 | 2.74E-08 | 1.72E-07 | 8.644878 |
| HNRPH1 | -0.84554 | 8.499495 | -6.28694 | 2.74E-08 | 1.72E-07 | 8.643456 |
| BRS3 | 1.476898 | 7.265466 | 6.286675 | 2.75E-08 | 1.73E-07 | 8.64241 |
| LIPA | -0.72739 | 7.365726 | -6.28655 | 2.75E-08 | 1.73E-07 | 8.641912 |

| | | | | | | |
|---|---|---|---|---|---|---|
| TBR1 | 1.177896 | 7.146765 | 6.2854 | 2.76E-08 | 1.73E-07 | 8.637353 |
| CYP4F11 | -0.94019 | 9.10156 | -6.28412 | 2.77E-08 | 1.74E-07 | 8.632262 |
| ACAD11 | -1.11958 | 7.823218 | -6.2838 | 2.78E-08 | 1.74E-07 | 8.631016 |
| GNAI2 | -1.08645 | 8.169967 | -6.28265 | 2.79E-08 | 1.75E-07 | 8.626444 |
| FLJ22536 | 1.220121 | 7.511027 | 6.281729 | 2.80E-08 | 1.76E-07 | 8.622799 |
| ZNF391 | 1.065014 | 7.023712 | 6.281499 | 2.80E-08 | 1.76E-07 | 8.621886 |
| ZSCAN2 | 0.788919 | 8.027956 | 6.281054 | 2.81E-08 | 1.76E-07 | 8.620121 |
| MTCH1 | -1.23948 | 8.08775 | -6.28072 | 2.81E-08 | 1.76E-07 | 8.618803 |
| WT1 | 0.726339 | 7.043792 | 6.278466 | 2.84E-08 | 1.78E-07 | 8.609861 |
| IDH3G | -0.83096 | 7.438273 | -6.27778 | 2.85E-08 | 1.78E-07 | 8.607143 |
| SCAMP2 | -0.70602 | 7.464037 | -6.27765 | 2.85E-08 | 1.78E-07 | 8.606642 |
| OXR1 | -0.9371 | 7.233697 | -6.27758 | 2.85E-08 | 1.78E-07 | 8.606353 |
| IAH1 | -0.83978 | 7.237011 | -6.27563 | 2.87E-08 | 1.79E-07 | 8.598615 |
| DOC2A | 1.125578 | 7.307446 | 6.275508 | 2.87E-08 | 1.79E-07 | 8.598138 |
| GPR142 | 1.455391 | 7.400216 | 6.274471 | 2.88E-08 | 1.80E-07 | 8.594025 |
| MSL3L1 | -0.87615 | 7.38332 | -6.27332 | 2.90E-08 | 1.81E-07 | 8.589473 |
| VTRNA1-1 | -1.94125 | 8.680941 | -6.27272 | 2.91E-08 | 1.81E-07 | 8.587079 |
| CHCHD10 | -1.20493 | 7.950156 | -6.26933 | 2.95E-08 | 1.83E-07 | 8.573642 |
| LOC40128 | 1.518699 | 7.781224 | 6.268775 | 2.95E-08 | 1.84E-07 | 8.571451 |
| SLC25A38 | 1.054267 | 8.947913 | 6.266477 | 2.98E-08 | 1.85E-07 | 8.562346 |
| SPATA3 | 1.180464 | 7.255106 | 6.265294 | 2.99E-08 | 1.86E-07 | 8.557656 |
| XAB2 | -0.87641 | 9.242327 | -6.26406 | 3.01E-08 | 1.87E-07 | 8.552782 |
| TMEM97 | -1.40702 | 8.091103 | -6.26236 | 3.03E-08 | 1.88E-07 | 8.546037 |
| PARP10 | -1.41928 | 10.07249 | -6.26227 | 3.03E-08 | 1.88E-07 | 8.545691 |
| PEBP1 | -1.01979 | 8.014014 | -6.25902 | 3.07E-08 | 1.90E-07 | 8.532782 |
| TMEM56 | -1.23358 | 8.319906 | -6.25796 | 3.08E-08 | 1.91E-07 | 8.528621 |
| RHOA | -1.00126 | 7.727048 | -6.2574 | 3.09E-08 | 1.91E-07 | 8.526366 |
| MIR181B1 | 0.771445 | 6.926658 | 6.256232 | 3.11E-08 | 1.92E-07 | 8.521759 |
| RP9 | -1.37023 | 7.972709 | -6.25622 | 3.11E-08 | 1.92E-07 | 8.521704 |
| COL1A1 | -1.6971 | 8.592835 | -6.25573 | 3.11E-08 | 1.92E-07 | 8.51975 |
| KRT73 | 0.718867 | 6.924123 | 6.254215 | 3.13E-08 | 1.93E-07 | 8.51377 |
| ANKRD20A | -0.82679 | 7.164088 | -6.25417 | 3.13E-08 | 1.93E-07 | 8.513593 |
| ASGR1 | -1.24043 | 7.922044 | -6.25356 | 3.14E-08 | 1.94E-07 | 8.511159 |
| C5orf34 | 1.349342 | 7.580436 | 6.253307 | 3.14E-08 | 1.94E-07 | 8.510174 |
| MIR554 | 1.147704 | 7.521684 | 6.252927 | 3.15E-08 | 1.94E-07 | 8.508667 |
| CNDP2 | -1.16301 | 8.023574 | -6.25237 | 3.15E-08 | 1.94E-07 | 8.506456 |
| ERGIC1 | 0.935371 | 8.254711 | 6.250356 | 3.18E-08 | 1.96E-07 | 8.498488 |
| FLJ38723 | 0.857482 | 6.900461 | 6.250232 | 3.18E-08 | 1.96E-07 | 8.497997 |
| ZNF622 | -0.74777 | 7.195672 | -6.24812 | 3.21E-08 | 1.98E-07 | 8.489624 |
| PPM1G | -1.39938 | 9.430124 | -6.24803 | 3.21E-08 | 1.98E-07 | 8.489273 |
| C18orf34 | 0.752548 | 6.877416 | 6.246858 | 3.23E-08 | 1.98E-07 | 8.484635 |
| LAMA5 | -1.17506 | 8.678634 | -6.2466 | 3.23E-08 | 1.99E-07 | 8.483602 |
| SNORA2A | -0.90283 | 7.441385 | -6.24643 | 3.23E-08 | 1.99E-07 | 8.482923 |
| EIF2A | -1.27498 | 9.510253 | -6.24516 | 3.25E-08 | 2.00E-07 | 8.477913 |
| MIR152 | 0.936788 | 6.855428 | 6.241861 | 3.29E-08 | 2.02E-07 | 8.464852 |
| HARS2 | -0.97852 | 7.953936 | -6.24157 | 3.30E-08 | 2.02E-07 | 8.463682 |
| IDS | -1.03843 | 7.934029 | -6.24047 | 3.31E-08 | 2.03E-07 | 8.459338 |
| UBA1 | -1.05877 | 9.405727 | -6.24035 | 3.31E-08 | 2.03E-07 | 8.458881 |
| ADAMTS1 | 1.691286 | 7.476783 | 6.2398 | 3.32E-08 | 2.04E-07 | 8.456694 |
| RFTN1 | -0.93788 | 7.841887 | -6.23896 | 3.33E-08 | 2.04E-07 | 8.453377 |
| MRPL12 | -1.51882 | 8.152581 | -6.23881 | 3.33E-08 | 2.04E-07 | 8.452772 |
| SNORD3A | -2.15243 | 10.61475 | -6.23859 | 3.34E-08 | 2.04E-07 | 8.451921 |
| JUN | -1.55384 | 9.126501 | -6.23853 | 3.34E-08 | 2.04E-07 | 8.451674 |
| PLOD3 | -1.15077 | 9.323012 | -6.23849 | 3.34E-08 | 2.04E-07 | 8.451517 |
| HNRNPA2 | -0.78313 | 7.327461 | -6.23721 | 3.35E-08 | 2.05E-07 | 8.446436 |
| CCDC74B | 1.443942 | 7.42367 | 6.237153 | 3.36E-08 | 2.05E-07 | 8.446219 |
| MIR298 | 0.946117 | 6.936835 | 6.236296 | 3.37E-08 | 2.06E-07 | 8.442828 |
| AMAC1 | 1.407716 | 7.223532 | 6.236236 | 3.37E-08 | 2.06E-07 | 8.442589 |

| | | | | | | |
|---|---|---|---|---|---|---|
| CLK3 | -0.78314 | 7.760302 | -6.23506 | 3.38E-08 | 2.07E-07 | 8.437928 |
| ZFP28 | 0.911366 | 6.831828 | 6.234767 | 3.39E-08 | 2.07E-07 | 8.436774 |
| OR5B12 | 1.282996 | 7.136672 | 6.234624 | 3.39E-08 | 2.07E-07 | 8.43621 |
| C4orf17 | 0.823438 | 6.815625 | 6.234544 | 3.39E-08 | 2.07E-07 | 8.435894 |
| SLC35E4 | 1.32864 | 7.375949 | 6.234467 | 3.39E-08 | 2.07E-07 | 8.435588 |
| DARS2 | -1.24704 | 8.015498 | -6.23411 | 3.40E-08 | 2.07E-07 | 8.434188 |
| PEBP4 | 1.0101 | 7.219674 | 6.23293 | 3.41E-08 | 2.08E-07 | 8.429504 |
| RARA | -0.96815 | 8.840124 | -6.23201 | 3.43E-08 | 2.09E-07 | 8.425855 |
| C10orf110 | 0.908141 | 7.055347 | 6.231493 | 3.43E-08 | 2.09E-07 | 8.423819 |
| ASS1 | -1.02773 | 8.316024 | -6.23136 | 3.43E-08 | 2.09E-07 | 8.423303 |
| LOC10019 | 0.732738 | 6.882741 | 6.231301 | 3.44E-08 | 2.09E-07 | 8.423061 |
| UBE2D3 | -0.98329 | 8.736954 | -6.23083 | 3.44E-08 | 2.09E-07 | 8.421191 |
| PSMB1 | -0.94693 | 8.050688 | -6.22987 | 3.46E-08 | 2.10E-07 | 8.417404 |
| MAPK14 | -0.79298 | 8.034879 | -6.22943 | 3.46E-08 | 2.10E-07 | 8.415666 |
| FCER2 | 0.959482 | 7.114412 | 6.229282 | 3.46E-08 | 2.10E-07 | 8.415073 |
| MIR769 | 1.040006 | 7.042971 | 6.227141 | 3.49E-08 | 2.12E-07 | 8.406601 |
| IFNA21 | 0.739014 | 6.951074 | 6.227085 | 3.49E-08 | 2.12E-07 | 8.406379 |
| SNORA47 | -2.10833 | 9.073497 | -6.22523 | 3.52E-08 | 2.14E-07 | 8.399054 |
| REM1 | 1.175768 | 7.405741 | 6.224293 | 3.53E-08 | 2.14E-07 | 8.395337 |
| OR2W3 | 0.70832 | 7.040798 | 6.224241 | 3.54E-08 | 2.14E-07 | 8.395131 |
| CST9 | 1.082251 | 7.002838 | 6.223988 | 3.54E-08 | 2.14E-07 | 8.394131 |
| PAX9 | 0.751597 | 6.868435 | 6.223837 | 3.54E-08 | 2.15E-07 | 8.393532 |
| SNORA71 | -1.13531 | 8.177041 | -6.22232 | 3.56E-08 | 2.16E-07 | 8.387524 |
| AHNAK | -0.90325 | 8.330205 | -6.22145 | 3.58E-08 | 2.16E-07 | 8.384086 |
| PGLYRP3 | 0.994275 | 7.158772 | 6.220079 | 3.60E-08 | 2.18E-07 | 8.37867 |
| PSMC3 | -1.06435 | 7.658802 | -6.21986 | 3.60E-08 | 2.18E-07 | 8.377799 |
| CYB5R3 | -1.26587 | 9.008363 | -6.21828 | 3.62E-08 | 2.19E-07 | 8.37156 |
| LOC14474 | 0.912378 | 6.8964 | 6.217914 | 3.63E-08 | 2.19E-07 | 8.370106 |
| MITD1 | -0.78109 | 7.220807 | -6.21698 | 3.64E-08 | 2.20E-07 | 8.366414 |
| MDH1 | -0.94718 | 7.758901 | -6.21575 | 3.66E-08 | 2.21E-07 | 8.361541 |
| MIR567 | 0.806358 | 6.837011 | 6.21551 | 3.66E-08 | 2.21E-07 | 8.360601 |
| DPM1 | -0.78305 | 7.716642 | -6.21535 | 3.66E-08 | 2.21E-07 | 8.359957 |
| KRTAP3-1 | 1.312357 | 7.315274 | 6.215324 | 3.67E-08 | 2.21E-07 | 8.359863 |
| AKT1 | -1.07613 | 10.39473 | -6.21516 | 3.67E-08 | 2.21E-07 | 8.359221 |
| NSUN2 | -0.71924 | 7.167281 | -6.21424 | 3.68E-08 | 2.22E-07 | 8.355581 |
| CD207 | 2.177126 | 8.377557 | 6.213702 | 3.69E-08 | 2.22E-07 | 8.353451 |
| SLC22A8 | 1.436145 | 7.319201 | 6.213435 | 3.69E-08 | 2.22E-07 | 8.352396 |
| C2orf28 | -0.75428 | 7.783384 | -6.21337 | 3.69E-08 | 2.22E-07 | 8.352137 |
| RNY1 | -1.2496 | 11.21531 | -6.21281 | 3.70E-08 | 2.23E-07 | 8.349917 |
| FAM195A | -1.07804 | 8.263096 | -6.20915 | 3.76E-08 | 2.26E-07 | 8.335443 |
| FBXO40 | 0.807302 | 6.819684 | 6.208651 | 3.77E-08 | 2.26E-07 | 8.333481 |
| RNF181 | -1.08138 | 7.940362 | -6.20826 | 3.77E-08 | 2.26E-07 | 8.331916 |
| ALAS1 | -0.789 | 7.212351 | -6.20784 | 3.78E-08 | 2.27E-07 | 8.330266 |
| C9orf80 | 0.826686 | 9.052642 | 6.207409 | 3.78E-08 | 2.27E-07 | 8.328572 |
| MAP4 | -0.85243 | 8.39633 | -6.20676 | 3.79E-08 | 2.28E-07 | 8.325992 |
| ABCC3 | -0.90258 | 7.352578 | -6.20667 | 3.80E-08 | 2.28E-07 | 8.325666 |
| MIR134 | 0.860221 | 7.006152 | 6.20587 | 3.81E-08 | 2.28E-07 | 8.322489 |
| LHX6 | 1.07566 | 7.353532 | 6.205806 | 3.81E-08 | 2.28E-07 | 8.322234 |
| SLC4A1 | 1.455432 | 7.366821 | 6.205356 | 3.82E-08 | 2.29E-07 | 8.320459 |
| SYT11 | 1.512977 | 8.897975 | 6.205232 | 3.82E-08 | 2.29E-07 | 8.319966 |
| STT3A | -1.33062 | 8.387807 | -6.20355 | 3.84E-08 | 2.30E-07 | 8.313325 |
| PDGFRL | 1.590673 | 8.267322 | 6.203165 | 3.85E-08 | 2.30E-07 | 8.311798 |
| PELP1 | -1.50667 | 8.099396 | -6.20273 | 3.86E-08 | 2.31E-07 | 8.310086 |
| PRPF8 | -1.09649 | 8.235788 | -6.20114 | 3.88E-08 | 2.32E-07 | 8.303794 |
| PYGM | 1.399246 | 7.358072 | 6.201016 | 3.88E-08 | 2.32E-07 | 8.303307 |
| BMP8B | 1.017904 | 7.738815 | 6.19853 | 3.92E-08 | 2.34E-07 | 8.293481 |
| FLJ45445 | 1.688371 | 9.473957 | 6.198125 | 3.93E-08 | 2.35E-07 | 8.291882 |
| SLC9A4 | 1.066771 | 11.70433 | 6.198081 | 3.93E-08 | 2.35E-07 | 8.291708 |

| | | | | | | |
|---|---|---|---|---|---|---|
| C7orf41 | -0.71359 | 7.250902 | -6.19779 | 3.93E-08 | 2.35E-07 | 8.290577 |
| NPEPL1 | -0.93194 | 8.058562 | -6.19712 | 3.95E-08 | 2.35E-07 | 8.287915 |
| HP | -2.26625 | 10.16649 | -6.19603 | 3.96E-08 | 2.36E-07 | 8.283615 |
| SLC30A4 | 1.227797 | 8.609203 | 6.19593 | 3.96E-08 | 2.36E-07 | 8.283208 |
| HOXB9 | 0.785781 | 6.919956 | 6.195338 | 3.97E-08 | 2.37E-07 | 8.280872 |
| GNL1 | -0.81418 | 9.26851 | -6.1947 | 3.98E-08 | 2.37E-07 | 8.278363 |
| OR51G1 | 0.847347 | 6.912277 | 6.193816 | 4.00E-08 | 2.38E-07 | 8.274859 |
| MAOA | -1.91365 | 8.453769 | -6.19363 | 4.00E-08 | 2.38E-07 | 8.27411 |
| SCARNA3 | -1.22515 | 9.665449 | -6.1925 | 4.02E-08 | 2.39E-07 | 8.269655 |
| YY1AP1 | -1.4637 | 9.892659 | -6.19195 | 4.03E-08 | 2.40E-07 | 8.267467 |
| RAB12 | -0.84648 | 7.32888 | -6.19042 | 4.05E-08 | 2.41E-07 | 8.261461 |
| BMP4 | 1.020742 | 7.750821 | 6.189087 | 4.08E-08 | 2.42E-07 | 8.256177 |
| SFRS4 | -0.71471 | 7.649132 | -6.18857 | 4.08E-08 | 2.43E-07 | 8.254133 |
| KCNMA1 | 0.936318 | 8.06588 | 6.187103 | 4.11E-08 | 2.44E-07 | 8.24834 |
| TAF13 | -1.82645 | 9.096502 | -6.18537 | 4.14E-08 | 2.45E-07 | 8.241484 |
| FAM178B | 1.705327 | 7.934329 | 6.185049 | 4.14E-08 | 2.46E-07 | 8.24023 |
| SPRED3 | 0.843066 | 6.97449 | 6.185029 | 4.14E-08 | 2.46E-07 | 8.24015 |
| GTF2H5 | -0.72446 | 7.477098 | -6.18463 | 4.15E-08 | 2.46E-07 | 8.238567 |
| IL19 | 0.812779 | 6.922194 | 6.184493 | 4.15E-08 | 2.46E-07 | 8.238033 |
| GALT | -0.70276 | 7.517478 | -6.18289 | 4.18E-08 | 2.47E-07 | 8.23172 |
| VPREB1 | 0.77953 | 6.778074 | 6.182101 | 4.19E-08 | 2.48E-07 | 8.228589 |
| GFM1 | -0.99033 | 7.476294 | -6.18203 | 4.19E-08 | 2.48E-07 | 8.228296 |
| COX7A2 | -1.70893 | 8.954913 | -6.18163 | 4.20E-08 | 2.48E-07 | 8.226734 |
| OR4P4 | 1.016419 | 6.908381 | 6.181242 | 4.21E-08 | 2.49E-07 | 8.225195 |
| PAQR4 | -1.28502 | 7.857345 | -6.18083 | 4.21E-08 | 2.49E-07 | 8.223551 |
| CHST3 | 1.399237 | 8.119306 | 6.179689 | 4.23E-08 | 2.50E-07 | 8.219063 |
| HCFC1 | -1.04972 | 7.497531 | -6.17916 | 4.24E-08 | 2.50E-07 | 8.216959 |
| CCDC72 | -1.37375 | 9.072852 | -6.17597 | 4.30E-08 | 2.54E-07 | 8.204386 |
| LOC44166 | 0.734416 | 6.987722 | 6.175537 | 4.30E-08 | 2.54E-07 | 8.202672 |
| SUMO2 | -1.35827 | 9.165665 | -6.17552 | 4.31E-08 | 2.54E-07 | 8.202591 |
| FLJ10661 | 0.73347 | 7.219479 | 6.174479 | 4.32E-08 | 2.55E-07 | 8.198497 |
| SNTG1 | 1.51365 | 7.462138 | 6.173358 | 4.34E-08 | 2.56E-07 | 8.194074 |
| CT47A6 | 1.133766 | 7.418096 | 6.171903 | 4.37E-08 | 2.57E-07 | 8.188329 |
| CHX10 | 1.094899 | 7.124978 | 6.171261 | 4.38E-08 | 2.58E-07 | 8.185795 |
| KIAA1688 | -1.36319 | 8.608187 | -6.17064 | 4.39E-08 | 2.58E-07 | 8.183335 |
| ACLY | -1.51908 | 8.458169 | -6.17064 | 4.39E-08 | 2.58E-07 | 8.183331 |
| PCCB | -1.06516 | 8.979185 | -6.16938 | 4.41E-08 | 2.60E-07 | 8.178388 |
| FAM118B | 1.121868 | 8.399963 | 6.168987 | 4.42E-08 | 2.60E-07 | 8.176822 |
| C5orf20 | 1.035548 | 7.511386 | 6.168774 | 4.42E-08 | 2.60E-07 | 8.17598 |
| DCDC2 | -1.21029 | 7.575283 | -6.16716 | 4.45E-08 | 2.62E-07 | 8.169598 |
| UFSP2 | -0.94126 | 7.592205 | -6.16627 | 4.47E-08 | 2.62E-07 | 8.166106 |
| PAQR8 | 0.882488 | 6.983413 | 6.166101 | 4.47E-08 | 2.62E-07 | 8.165431 |
| C21orf86 | 1.148701 | 7.120311 | 6.164883 | 4.49E-08 | 2.64E-07 | 8.160627 |
| PCM1 | -0.70956 | 7.222697 | -6.16432 | 4.50E-08 | 2.64E-07 | 8.158418 |
| EFNA5 | 1.491136 | 7.599833 | 6.161557 | 4.55E-08 | 2.67E-07 | 8.147506 |
| SYT14L | 1.025158 | 7.027025 | 6.161224 | 4.56E-08 | 2.67E-07 | 8.146192 |
| CGB5 | 1.510322 | 7.354693 | 6.160954 | 4.57E-08 | 2.67E-07 | 8.145128 |
| C4A | -0.992 | 7.602063 | -6.15971 | 4.59E-08 | 2.69E-07 | 8.14023 |
| SLC5A4 | 0.810796 | 6.832217 | 6.156829 | 4.64E-08 | 2.71E-07 | 8.128854 |
| GLUD1 | -0.97946 | 8.095654 | -6.15597 | 4.66E-08 | 2.72E-07 | 8.125481 |
| SNORA14 | -1.15672 | 8.150688 | -6.15423 | 4.69E-08 | 2.74E-07 | 8.118593 |
| C20orf12 | 1.304311 | 7.645976 | 6.153964 | 4.70E-08 | 2.74E-07 | 8.117556 |
| KLHL28 | 1.22973 | 9.027805 | 6.153733 | 4.70E-08 | 2.75E-07 | 8.116646 |
| EMILIN3 | 0.757507 | 6.79177 | 6.15173 | 4.74E-08 | 2.77E-07 | 8.108745 |
| TMEM175 | -1.49411 | 7.831848 | -6.15151 | 4.74E-08 | 2.77E-07 | 8.107861 |
| MTRF1L | 1.123045 | 7.915619 | 6.151378 | 4.75E-08 | 2.77E-07 | 8.107357 |
| MRPL13 | -0.74483 | 7.431518 | -6.15015 | 4.77E-08 | 2.78E-07 | 8.102521 |
| KRTAP6-2 | 0.803979 | 6.869639 | 6.147666 | 4.82E-08 | 2.81E-07 | 8.092723 |

| | | | | | | |
|---|---|---|---|---|---|---|
| ALDH9A1 | -1.76167 | 9.27466 | -6.14266 | 4.92E-08 | 2.86E-07 | 8.073002 |
| SLC25A42 | -1.24352 | 8.874924 | -6.14224 | 4.92E-08 | 2.87E-07 | 8.071325 |
| GPX2 | -1.2028 | 7.848156 | -6.14169 | 4.94E-08 | 2.87E-07 | 8.069178 |
| CCNA1 | 0.881324 | 6.778595 | 6.140702 | 4.95E-08 | 2.88E-07 | 8.065268 |
| UTX | -0.9571 | 8.404608 | -6.13976 | 4.97E-08 | 2.89E-07 | 8.061571 |
| OPN4 | 0.77799 | 6.908545 | 6.13914 | 4.99E-08 | 2.90E-07 | 8.059111 |
| JUNB | -1.26343 | 8.564636 | -6.13818 | 5.01E-08 | 2.91E-07 | 8.055316 |
| RNASE13 | 0.881105 | 7.055393 | 6.137459 | 5.02E-08 | 2.91E-07 | 8.05249 |
| C21orf42 | 0.851559 | 7.137548 | 6.137222 | 5.02E-08 | 2.92E-07 | 8.051555 |
| MYL12A | -1.93702 | 8.336705 | -6.1359 | 5.05E-08 | 2.93E-07 | 8.046363 |
| HMX1 | 0.769241 | 6.821757 | 6.132546 | 5.12E-08 | 2.97E-07 | 8.033129 |
| TTC39C | -1.22408 | 8.910436 | -6.1315 | 5.14E-08 | 2.98E-07 | 8.029025 |
| TTC9B | 0.990997 | 6.91011 | 6.12959 | 5.18E-08 | 3.00E-07 | 8.021482 |
| C16orf35 | -0.90164 | 7.367915 | -6.12955 | 5.18E-08 | 3.00E-07 | 8.021313 |
| C7orf66 | 0.834512 | 6.839927 | 6.129329 | 5.19E-08 | 3.00E-07 | 8.020454 |
| ARMC4 | 0.775929 | 6.82202 | 6.129327 | 5.19E-08 | 3.00E-07 | 8.020447 |
| PC | -0.74769 | 7.684875 | -6.12903 | 5.19E-08 | 3.00E-07 | 8.019259 |
| AOX1 | -1.01563 | 7.379644 | -6.12884 | 5.20E-08 | 3.00E-07 | 8.018537 |
| OR10Q1 | 1.511882 | 7.302468 | 6.126007 | 5.26E-08 | 3.03E-07 | 8.00737 |
| ZNF287 | 0.961112 | 7.150241 | 6.125322 | 5.27E-08 | 3.04E-07 | 8.00467 |
| USF2 | -0.78813 | 8.343306 | -6.12532 | 5.27E-08 | 3.04E-07 | 8.004658 |
| ATP6V1B1 | 1.580048 | 8.172308 | 6.125212 | 5.27E-08 | 3.04E-07 | 8.004237 |
| KIAA1407 | 1.219662 | 7.942815 | 6.12446 | 5.29E-08 | 3.05E-07 | 8.001277 |
| TLK1 | -0.8163 | 7.277381 | -6.12405 | 5.30E-08 | 3.05E-07 | 7.999656 |
| ABHD8 | -0.88448 | 7.410102 | -6.12272 | 5.33E-08 | 3.07E-07 | 7.994442 |
| HDGFL1 | 1.338796 | 7.233883 | 6.122446 | 5.33E-08 | 3.07E-07 | 7.993343 |
| SCARA5 | 0.966356 | 7.141114 | 6.122162 | 5.34E-08 | 3.07E-07 | 7.992225 |
| BEND7 | 0.785618 | 7.59371 | 6.121319 | 5.36E-08 | 3.08E-07 | 7.988904 |
| ANXA13 | 0.882271 | 7.328517 | 6.12025 | 5.38E-08 | 3.09E-07 | 7.984695 |
| GSTA5 | 1.023939 | 6.842729 | 6.119516 | 5.40E-08 | 3.10E-07 | 7.981807 |
| SNORD83 | -1.40184 | 8.033325 | -6.119 | 5.41E-08 | 3.11E-07 | 7.979779 |
| OR1F2P | 1.041942 | 7.091709 | 6.118635 | 5.42E-08 | 3.11E-07 | 7.978335 |
| YARS | -1.00475 | 7.601566 | -6.11718 | 5.45E-08 | 3.13E-07 | 7.972598 |
| HIPK2 | -0.87354 | 8.004245 | -6.11579 | 5.48E-08 | 3.15E-07 | 7.967147 |
| APBB3 | -0.92507 | 7.935315 | -6.11342 | 5.53E-08 | 3.17E-07 | 7.957787 |
| SBK2 | 1.924236 | 7.471638 | 6.11257 | 5.55E-08 | 3.18E-07 | 7.954458 |
| TUBA4A | -0.96116 | 7.356098 | -6.11188 | 5.57E-08 | 3.19E-07 | 7.951751 |
| OR4K13 | 1.066683 | 7.232137 | 6.111579 | 5.57E-08 | 3.19E-07 | 7.950557 |
| RPL24 | -1.42462 | 9.380337 | -6.11073 | 5.59E-08 | 3.20E-07 | 7.947217 |
| IMMT | -0.70113 | 7.477574 | -6.10958 | 5.62E-08 | 3.22E-07 | 7.942686 |
| PNLIP | 1.249156 | 7.12459 | 6.109494 | 5.62E-08 | 3.22E-07 | 7.942352 |
| NPTXR | 1.435902 | 7.746183 | 6.109019 | 5.63E-08 | 3.22E-07 | 7.940481 |
| OR1L3 | 0.709807 | 6.818256 | 6.108917 | 5.63E-08 | 3.22E-07 | 7.940079 |
| TMEM16D | 0.962334 | 7.006229 | 6.10718 | 5.67E-08 | 3.24E-07 | 7.933244 |
| PRUNE2 | 0.843605 | 6.987953 | 6.106486 | 5.69E-08 | 3.25E-07 | 7.930513 |
| GNB2L1 | -1.18396 | 10.65251 | -6.10642 | 5.69E-08 | 3.25E-07 | 7.930252 |
| DEFB126 | 0.766184 | 6.787502 | 6.105989 | 5.70E-08 | 3.26E-07 | 7.928557 |
| MBD3 | -0.79288 | 7.337081 | -6.10561 | 5.71E-08 | 3.26E-07 | 7.927081 |
| NPFFR1 | 1.642356 | 7.350088 | 6.105445 | 5.71E-08 | 3.26E-07 | 7.926417 |
| ARHGEF16 | -1.05857 | 7.569586 | -6.10415 | 5.74E-08 | 3.28E-07 | 7.921317 |
| MT1G | -1.66262 | 9.153082 | -6.10379 | 5.75E-08 | 3.28E-07 | 7.919917 |
| PNN | -1.0739 | 7.714583 | -6.10347 | 5.76E-08 | 3.28E-07 | 7.918644 |
| ATP6V0B | -1.25133 | 9.202876 | -6.10253 | 5.78E-08 | 3.29E-07 | 7.914944 |
| KLF15 | -1.05062 | 8.360529 | -6.10112 | 5.81E-08 | 3.31E-07 | 7.909415 |
| NTN4 | -0.72004 | 7.555748 | -6.10017 | 5.83E-08 | 3.32E-07 | 7.905654 |
| CYP2E1 | -1.89297 | 9.654357 | -6.09601 | 5.93E-08 | 3.38E-07 | 7.889296 |
| OR5AK2 | 0.790038 | 6.798709 | 6.095894 | 5.94E-08 | 3.38E-07 | 7.888842 |
| NACAP1 | 0.801857 | 6.807678 | 6.095871 | 5.94E-08 | 3.38E-07 | 7.888748 |

| | | | | | | |
|---|---|---|---|---|---|---|
| HS3ST5 | 0.72192 | 6.7575 | 6.095132 | 5.95E-08 | 3.38E-07 | 7.885841 |
| LOC6515C | 0.968535 | 6.869985 | 6.094779 | 5.96E-08 | 3.39E-07 | 7.884454 |
| KLRG2 | 1.384809 | 7.084565 | 6.093875 | 5.98E-08 | 3.40E-07 | 7.8809 |
| FLJ42953 | 1.372565 | 7.30431 | 6.092058 | 6.03E-08 | 3.42E-07 | 7.873754 |
| SCNN1B | 1.235673 | 7.297799 | 6.090792 | 6.06E-08 | 3.44E-07 | 7.868775 |
| DPYS | -0.95902 | 7.610616 | -6.08857 | 6.11E-08 | 3.47E-07 | 7.860017 |
| PDZD8 | -1.03669 | 7.864282 | -6.08836 | 6.12E-08 | 3.47E-07 | 7.859208 |
| SNORD1A | -1.47724 | 8.23697 | -6.08824 | 6.12E-08 | 3.47E-07 | 7.858738 |
| GPM6B | 0.804047 | 7.018021 | 6.087711 | 6.13E-08 | 3.48E-07 | 7.856659 |
| APOH | -1.34286 | 7.940071 | -6.0865 | 6.16E-08 | 3.49E-07 | 7.851908 |
| KLK6 | 0.99022 | 7.033965 | 6.084246 | 6.22E-08 | 3.52E-07 | 7.843034 |
| MMP16 | 0.714435 | 7.04691 | 6.083759 | 6.23E-08 | 3.53E-07 | 7.84112 |
| EPAS1 | -1.47967 | 8.883624 | -6.08348 | 6.24E-08 | 3.53E-07 | 7.840036 |
| SNORD90 | -1.23668 | 7.774649 | -6.08326 | 6.25E-08 | 3.53E-07 | 7.839143 |
| EFNB3 | 1.135932 | 7.141549 | 6.08275 | 6.26E-08 | 3.54E-07 | 7.837155 |
| C20orf77 | 1.092142 | 7.122068 | 6.081108 | 6.30E-08 | 3.56E-07 | 7.8307 |
| PCK2 | -1.02013 | 8.460738 | -6.08098 | 6.30E-08 | 3.56E-07 | 7.830188 |
| WNK1 | -0.76033 | 8.140957 | -6.08052 | 6.31E-08 | 3.57E-07 | 7.828388 |
| SNORD12 | -1.1268 | 7.55416 | -6.07996 | 6.33E-08 | 3.57E-07 | 7.826207 |
| TSHZ2 | -0.97598 | 8.395166 | -6.07956 | 6.34E-08 | 3.58E-07 | 7.824617 |
| BTN1A1 | 1.32159 | 7.288559 | 6.078607 | 6.36E-08 | 3.59E-07 | 7.820869 |
| PI4KB | -1.34664 | 8.308333 | -6.07857 | 6.36E-08 | 3.59E-07 | 7.820713 |
| IFI27 | -1.70799 | 8.434287 | -6.07728 | 6.40E-08 | 3.60E-07 | 7.815665 |
| ACMSD | -1.20702 | 8.085112 | -6.07728 | 6.40E-08 | 3.60E-07 | 7.815654 |
| C15orf37 | 0.778951 | 7.160107 | 6.076988 | 6.40E-08 | 3.61E-07 | 7.814508 |
| KRTAP10- | 0.837402 | 6.837755 | 6.076893 | 6.41E-08 | 3.61E-07 | 7.814133 |
| PROX1 | -0.83391 | 7.417428 | -6.07642 | 6.42E-08 | 3.61E-07 | 7.812262 |
| PRICKLE1 | 1.748216 | 9.25263 | 6.076403 | 6.42E-08 | 3.61E-07 | 7.812206 |
| CALD1 | -0.99938 | 8.038529 | -6.07363 | 6.49E-08 | 3.65E-07 | 7.801322 |
| C18orf21 | -0.84382 | 7.22289 | -6.07356 | 6.49E-08 | 3.65E-07 | 7.801022 |
| MCART2 | 0.722879 | 6.854664 | 6.072961 | 6.51E-08 | 3.66E-07 | 7.798682 |
| VPS36 | -0.89925 | 7.935488 | -6.07267 | 6.52E-08 | 3.66E-07 | 7.79755 |
| DDX1 | -0.93746 | 7.581712 | -6.07263 | 6.52E-08 | 3.66E-07 | 7.797365 |
| CDC42BPE | -0.92335 | 7.927518 | -6.07238 | 6.52E-08 | 3.66E-07 | 7.796404 |
| ACTRT2 | 1.215887 | 7.077411 | 6.072348 | 6.53E-08 | 3.66E-07 | 7.796272 |
| RHOU | -1.47364 | 7.829144 | -6.07058 | 6.57E-08 | 3.68E-07 | 7.78931 |
| SELM | -1.79519 | 9.196141 | -6.07043 | 6.58E-08 | 3.68E-07 | 7.788729 |
| GDF15 | -1.21492 | 7.650306 | -6.07027 | 6.58E-08 | 3.69E-07 | 7.788111 |
| OSBPL7 | 1.11955 | 7.59631 | 6.069874 | 6.59E-08 | 3.69E-07 | 7.786555 |
| LZTR1 | -1.08409 | 9.551137 | -6.06918 | 6.61E-08 | 3.70E-07 | 7.783826 |
| SMARCA1 | -0.72134 | 7.251131 | -6.0682 | 6.64E-08 | 3.71E-07 | 7.779978 |
| NDUFB7 | -1.96768 | 9.297602 | -6.06773 | 6.65E-08 | 3.72E-07 | 7.778134 |
| ESYT1 | -1.43294 | 9.253773 | -6.0672 | 6.66E-08 | 3.72E-07 | 7.776053 |
| LINGO1 | 1.407141 | 7.456515 | 6.066348 | 6.69E-08 | 3.73E-07 | 7.772702 |
| AP2S1 | -1.36225 | 9.78647 | -6.06498 | 6.72E-08 | 3.75E-07 | 7.767315 |
| ENTPD6 | -0.93563 | 7.861818 | -6.06407 | 6.75E-08 | 3.76E-07 | 7.763754 |
| RAD21 | -0.88468 | 7.564487 | -6.06269 | 6.78E-08 | 3.78E-07 | 7.758343 |
| PTCH2 | 1.197037 | 7.454096 | 6.060917 | 6.83E-08 | 3.81E-07 | 7.751369 |
| APOA2 | -2.10236 | 10.64716 | -6.05964 | 6.87E-08 | 3.83E-07 | 7.746347 |
| GABRB2 | 0.809643 | 6.924705 | 6.059258 | 6.88E-08 | 3.83E-07 | 7.744855 |
| RNF150 | 1.714362 | 8.133815 | 6.058987 | 6.89E-08 | 3.84E-07 | 7.743793 |
| ROBLD3 | -0.7049 | 7.321627 | -6.05771 | 6.92E-08 | 3.86E-07 | 7.738797 |
| AP3B2 | 1.288912 | 7.259637 | 6.057135 | 6.94E-08 | 3.86E-07 | 7.736519 |
| OR1C1 | 1.502223 | 7.441878 | 6.055128 | 6.99E-08 | 3.89E-07 | 7.728642 |
| CX3CL1 | -0.70146 | 7.22148 | -6.05462 | 7.01E-08 | 3.90E-07 | 7.726661 |
| PROM1 | 1.391645 | 7.708977 | 6.05215 | 7.08E-08 | 3.94E-07 | 7.716951 |
| EIF3L | -1.05702 | 8.574897 | -6.04899 | 7.17E-08 | 3.98E-07 | 7.704558 |
| MKI67 | 1.845176 | 9.31679 | 6.04831 | 7.19E-08 | 3.99E-07 | 7.701876 |

| | | | | | | |
|---|---|---|---|---|---|---|
| TASP1 | 0.961462 | 7.56301 | 6.048305 | 7.19E-08 | 3.99E-07 | 7.701856 |
| DEFB118 | 1.753863 | 7.513233 | 6.048176 | 7.19E-08 | 3.99E-07 | 7.70135 |
| CETN2 | -0.71868 | 7.307077 | -6.0472 | 7.22E-08 | 4.01E-07 | 7.697527 |
| LOC64653 | -0.74549 | 10.30709 | -6.04649 | 7.24E-08 | 4.02E-07 | 7.69474 |
| NPHS1 | 0.851333 | 6.970161 | 6.044492 | 7.30E-08 | 4.05E-07 | 7.686892 |
| PSMB11 | 1.528559 | 7.396199 | 6.042515 | 7.36E-08 | 4.08E-07 | 7.679135 |
| FAM55A | 0.997265 | 6.969024 | 6.041272 | 7.39E-08 | 4.10E-07 | 7.674259 |
| CES2 | -1.0796 | 9.904443 | -6.04105 | 7.40E-08 | 4.10E-07 | 7.673387 |
| OR52B2 | 0.875421 | 6.907442 | 6.040131 | 7.43E-08 | 4.11E-07 | 7.669784 |
| RPL31 | -1.66062 | 9.723053 | -6.03653 | 7.54E-08 | 4.17E-07 | 7.655652 |
| RTP2 | 0.840666 | 6.930461 | 6.036243 | 7.55E-08 | 4.17E-07 | 7.654532 |
| FRYL | -0.73828 | 8.319321 | -6.0361 | 7.55E-08 | 4.17E-07 | 7.653977 |
| BCAM | -0.88422 | 7.396055 | -6.03518 | 7.58E-08 | 4.19E-07 | 7.650379 |
| RUNDC3A | 0.82559 | 6.951701 | 6.03419 | 7.61E-08 | 4.20E-07 | 7.646477 |
| OR51B6 | 0.933878 | 6.926391 | 6.034049 | 7.61E-08 | 4.20E-07 | 7.645924 |
| XPNPEP3 | 0.842769 | 7.577921 | 6.03198 | 7.68E-08 | 4.23E-07 | 7.637811 |
| ZNHIT3 | 1.109215 | 8.574778 | 6.031158 | 7.70E-08 | 4.24E-07 | 7.634589 |
| PNMT | 1.900493 | 7.467819 | 6.028508 | 7.78E-08 | 4.29E-07 | 7.624198 |
| EBNA1BP2 | -1.12788 | 8.000996 | -6.02648 | 7.85E-08 | 4.32E-07 | 7.616227 |
| SNORD4A | -0.86401 | 7.544398 | -6.02477 | 7.90E-08 | 4.34E-07 | 7.609529 |
| TSP50 | 0.728182 | 7.023352 | 6.024502 | 7.91E-08 | 4.35E-07 | 7.608491 |
| TSR2 | -1.13247 | 8.511393 | -6.02429 | 7.92E-08 | 4.35E-07 | 7.607661 |
| TIMD4 | 0.795146 | 6.931348 | 6.023763 | 7.93E-08 | 4.36E-07 | 7.605592 |
| OR1L8 | 0.983937 | 7.115914 | 6.02354 | 7.94E-08 | 4.36E-07 | 7.604719 |
| LONP2 | -0.80164 | 8.365293 | -6.02097 | 8.02E-08 | 4.40E-07 | 7.594628 |
| NKX2-2 | 1.064968 | 7.209337 | 6.019911 | 8.06E-08 | 4.42E-07 | 7.590494 |
| KLF8 | 0.846117 | 7.334728 | 6.01643 | 8.17E-08 | 4.48E-07 | 7.576852 |
| LOC44055 | 1.394524 | 7.108676 | 6.015827 | 8.19E-08 | 4.49E-07 | 7.57449 |
| UGT2A2 | 0.778244 | 6.840365 | 6.015247 | 8.21E-08 | 4.50E-07 | 7.572216 |
| GPAA1 | -0.91114 | 7.978836 | -6.01412 | 8.25E-08 | 4.51E-07 | 7.567808 |
| C15orf60 | 1.665527 | 7.307571 | 6.013656 | 8.26E-08 | 4.52E-07 | 7.565982 |
| TMEM139 | 1.00504 | 7.807374 | 6.013331 | 8.27E-08 | 4.53E-07 | 7.564709 |
| SCGB1D2 | 0.82586 | 6.882789 | 6.012371 | 8.30E-08 | 4.54E-07 | 7.560946 |
| SCGB1C1 | 1.398814 | 7.267111 | 6.011901 | 8.32E-08 | 4.55E-07 | 7.559107 |
| SCRG1 | 1.159379 | 7.154406 | 6.009816 | 8.39E-08 | 4.58E-07 | 7.550938 |
| SIK1 | -0.77446 | 7.36159 | -6.00954 | 8.40E-08 | 4.59E-07 | 7.549862 |
| MAN2B1 | -1.25948 | 8.03881 | -6.00938 | 8.40E-08 | 4.59E-07 | 7.549218 |
| SIRPB1 | 1.286673 | 7.598993 | 6.007463 | 8.47E-08 | 4.62E-07 | 7.541721 |
| ACO1 | -1.08267 | 7.890989 | -6.00417 | 8.58E-08 | 4.68E-07 | 7.528836 |
| C9orf116 | 0.814224 | 7.243562 | 6.002559 | 8.64E-08 | 4.71E-07 | 7.522513 |
| EPS15L1 | 0.989305 | 7.49291 | 6.001821 | 8.66E-08 | 4.72E-07 | 7.519623 |
| SNRNP70 | -1.22697 | 9.531289 | -6.00124 | 8.68E-08 | 4.73E-07 | 7.517362 |
| FAM19A3 | 1.010367 | 7.099177 | 5.998996 | 8.76E-08 | 4.77E-07 | 7.508561 |
| RCN3 | -0.81755 | 7.668854 | -5.9981 | 8.79E-08 | 4.78E-07 | 7.505039 |
| ADCY10 | 1.137798 | 7.322673 | 5.996214 | 8.86E-08 | 4.82E-07 | 7.497671 |
| NAPG | -0.70069 | 7.495082 | -5.99604 | 8.87E-08 | 4.82E-07 | 7.496975 |
| EEF1G | -2.06928 | 10.21889 | -5.99523 | 8.90E-08 | 4.83E-07 | 7.493822 |
| ARHGDIB | -1.53025 | 9.124657 | -5.99498 | 8.90E-08 | 4.84E-07 | 7.492824 |
| TCP11 | 0.707173 | 6.908268 | 5.993837 | 8.95E-08 | 4.86E-07 | 7.488364 |
| MRPL45 | -0.77508 | 7.741194 | -5.99297 | 8.98E-08 | 4.87E-07 | 7.48499 |
| STK31 | 1.341694 | 7.435283 | 5.991791 | 9.02E-08 | 4.89E-07 | 7.480358 |
| FLJ32011 | 2.202278 | 8.403631 | 5.991203 | 9.04E-08 | 4.90E-07 | 7.478055 |
| RALA | -0.88221 | 7.966431 | -5.99067 | 9.06E-08 | 4.91E-07 | 7.475988 |
| USP8 | -0.7688 | 7.667585 | -5.98896 | 9.12E-08 | 4.94E-07 | 7.469285 |
| PTPLAD2 | 1.63827 | 10.80128 | 5.988118 | 9.15E-08 | 4.96E-07 | 7.46598 |
| SOX30 | 1.631489 | 7.634401 | 5.985004 | 9.27E-08 | 5.01E-07 | 7.453796 |
| SNORA21 | -1.49767 | 8.286871 | -5.98453 | 9.29E-08 | 5.02E-07 | 7.451939 |
| FAM22D | 1.088315 | 7.196175 | 5.984348 | 9.29E-08 | 5.02E-07 | 7.451229 |

| | | | | | | |
|---|---|---|---|---|---|---|
| LOC10012 | 0.851801 | 7.111555 | 5.983042 | 9.34E-08 | 5.04E-07 | 7.446119 |
| STIM1 | -0.78275 | 7.911049 | -5.98221 | 9.37E-08 | 5.06E-07 | 7.442879 |
| ACP6 | -1.2119 | 7.547159 | -5.98154 | 9.40E-08 | 5.07E-07 | 7.44025 |
| SLC27A3 | -0.94778 | 7.583218 | -5.98108 | 9.41E-08 | 5.08E-07 | 7.438457 |
| TNFRSF1A | -0.78605 | 7.163529 | -5.98057 | 9.43E-08 | 5.09E-07 | 7.436449 |
| UBA3 | -0.9012 | 7.283001 | -5.97773 | 9.54E-08 | 5.15E-07 | 7.425341 |
| SNORA81 | -1.12989 | 7.775805 | -5.97373 | 9.70E-08 | 5.22E-07 | 7.409699 |
| HLA-DQA | 1.239067 | 6.99212 | 5.973082 | 9.72E-08 | 5.23E-07 | 7.407162 |
| EIF4G1 | -0.86612 | 8.372957 | -5.97307 | 9.72E-08 | 5.23E-07 | 7.407131 |
| YIPF6 | -0.87786 | 7.581543 | -5.97274 | 9.73E-08 | 5.24E-07 | 7.405808 |
| TUFM | -1.13549 | 9.204899 | -5.97218 | 9.76E-08 | 5.25E-07 | 7.403618 |
| BCAP31 | -1.12269 | 9.716059 | -5.97062 | 9.82E-08 | 5.28E-07 | 7.397551 |
| TMEM126 | -1.00706 | 7.518207 | -5.96937 | 9.87E-08 | 5.30E-07 | 7.392638 |
| LOC10019 | -0.84472 | 7.525978 | -5.96911 | 9.88E-08 | 5.30E-07 | 7.39163 |
| KRTAP5-1 | 0.80651 | 6.939253 | 5.968918 | 9.88E-08 | 5.30E-07 | 7.39088 |
| CYP4F22 | 1.872451 | 8.715478 | 5.968702 | 9.89E-08 | 5.31E-07 | 7.390036 |
| SNF8 | -0.73105 | 7.965692 | -5.96759 | 9.94E-08 | 5.33E-07 | 7.38569 |
| SGK1 | -0.95433 | 7.709792 | -5.96527 | 1.00E-07 | 5.37E-07 | 7.376621 |
| PPP1R3B | -0.75291 | 7.574793 | -5.96469 | 1.01E-07 | 5.38E-07 | 7.374336 |
| SNRPB | -1.09277 | 10.33228 | -5.96133 | 1.02E-07 | 5.45E-07 | 7.361225 |
| YWHAH | -1.57885 | 8.711599 | -5.96015 | 1.02E-07 | 5.47E-07 | 7.356592 |
| HPCAL1 | -0.82603 | 8.474913 | -5.95977 | 1.03E-07 | 5.48E-07 | 7.355129 |
| GADD45G | -1.4438 | 8.194769 | -5.95919 | 1.03E-07 | 5.49E-07 | 7.352845 |
| CNPY2 | -0.71118 | 7.274726 | -5.95912 | 1.03E-07 | 5.49E-07 | 7.352588 |
| HMGB4 | 0.710617 | 6.882398 | 5.959002 | 1.03E-07 | 5.49E-07 | 7.352124 |
| PMPCB | -0.85883 | 7.265069 | -5.95826 | 1.03E-07 | 5.50E-07 | 7.349225 |
| AKR7A3 | -1.32423 | 8.023406 | -5.95588 | 1.04E-07 | 5.55E-07 | 7.339922 |
| LRFN2 | 1.66196 | 7.692034 | 5.955262 | 1.04E-07 | 5.56E-07 | 7.337512 |
| C15orf51 | 0.714439 | 7.028179 | 5.952082 | 1.06E-07 | 5.63E-07 | 7.325088 |
| FAM43A | -0.79921 | 7.3565 | -5.95127 | 1.06E-07 | 5.65E-07 | 7.321931 |
| KIAA0895I | 1.104816 | 8.579385 | 5.95123 | 1.06E-07 | 5.65E-07 | 7.321762 |
| ZDHHC16 | -0.93372 | 8.3776 | -5.94937 | 1.07E-07 | 5.69E-07 | 7.314478 |
| ALDH1L1 | -0.92341 | 7.373532 | -5.94669 | 1.08E-07 | 5.74E-07 | 7.304032 |
| ITGB1BP3 | 0.835189 | 6.807142 | 5.945283 | 1.09E-07 | 5.77E-07 | 7.298536 |
| HOXA5 | 0.831439 | 7.306677 | 5.944456 | 1.09E-07 | 5.79E-07 | 7.295303 |
| HNRPM | -0.93229 | 8.423696 | -5.94216 | 1.10E-07 | 5.84E-07 | 7.286334 |
| TAS2R1 | 0.909477 | 6.950264 | 5.941775 | 1.10E-07 | 5.84E-07 | 7.284837 |
| UBR4 | -0.71781 | 9.040387 | -5.94176 | 1.10E-07 | 5.84E-07 | 7.284795 |
| NKX2-5 | 0.976212 | 6.99728 | 5.940502 | 1.11E-07 | 5.87E-07 | 7.279868 |
| OR5B21 | 1.675776 | 7.522255 | 5.940247 | 1.11E-07 | 5.87E-07 | 7.278873 |
| FRK | -0.84341 | 7.749321 | -5.94025 | 1.11E-07 | 5.87E-07 | 7.278872 |
| E2F4 | -0.76104 | 7.503476 | -5.93994 | 1.11E-07 | 5.88E-07 | 7.277669 |
| NLRP6 | 1.479315 | 7.68457 | 5.939696 | 1.11E-07 | 5.88E-07 | 7.27672 |
| FILIP1 | 0.723954 | 7.483495 | 5.939695 | 1.11E-07 | 5.88E-07 | 7.276718 |
| FMO2 | 1.322127 | 8.101304 | 5.938978 | 1.11E-07 | 5.89E-07 | 7.273916 |
| ZNF653 | -0.99285 | 8.101507 | -5.93769 | 1.12E-07 | 5.92E-07 | 7.268908 |
| FLJ23834 | 1.52087 | 8.006417 | 5.93618 | 1.13E-07 | 5.96E-07 | 7.262996 |
| FAM48B2 | 0.979796 | 6.992117 | 5.935913 | 1.13E-07 | 5.96E-07 | 7.261954 |
| CRYBB1 | 1.148063 | 7.479844 | 5.935258 | 1.13E-07 | 5.97E-07 | 7.259398 |
| ERCC2 | -1.03991 | 7.373339 | -5.9336 | 1.14E-07 | 6.01E-07 | 7.252932 |
| CRYL1 | -0.91364 | 7.826629 | -5.93347 | 1.14E-07 | 6.01E-07 | 7.252407 |
| AES | -0.92817 | 9.550116 | -5.93302 | 1.14E-07 | 6.02E-07 | 7.250645 |
| LOC10019 | 0.755857 | 6.828103 | 5.931966 | 1.15E-07 | 6.04E-07 | 7.246548 |
| PLCD4 | 1.588698 | 8.634177 | 5.930226 | 1.15E-07 | 6.08E-07 | 7.23976 |
| C22orf28 | -0.92058 | 8.264131 | -5.92817 | 1.16E-07 | 6.13E-07 | 7.231744 |
| LRRC16B | 1.410456 | 7.777173 | 5.927083 | 1.17E-07 | 6.15E-07 | 7.227499 |
| MRPL17 | -0.73236 | 7.178808 | -5.92589 | 1.17E-07 | 6.18E-07 | 7.222831 |
| HCP5 | -1.09168 | 7.893936 | -5.92418 | 1.18E-07 | 6.22E-07 | 7.216181 |

| | | | | | | |
|---|---|---|---|---|---|---|
| IGLL3 | 0.993993 | 7.314037 | 5.923746 | 1.18E-07 | 6.22E-07 | 7.214479 |
| USF1 | 0.990298 | 8.10976 | 5.923122 | 1.19E-07 | 6.24E-07 | 7.212048 |
| THBS1 | -0.79076 | 7.121993 | -5.92147 | 1.20E-07 | 6.27E-07 | 7.205623 |
| PSD4 | -1.10106 | 7.590451 | -5.91979 | 1.20E-07 | 6.31E-07 | 7.199063 |
| FKBP9L | 0.975162 | 7.875073 | 5.91919 | 1.21E-07 | 6.33E-07 | 7.19671 |
| CNTD1 | 0.754862 | 6.950952 | 5.918587 | 1.21E-07 | 6.34E-07 | 7.194359 |
| PTCD1 | -1.17886 | 8.151055 | -5.9172 | 1.22E-07 | 6.37E-07 | 7.188964 |
| SLC2A5 | 1.165857 | 7.607129 | 5.917095 | 1.22E-07 | 6.37E-07 | 7.188543 |
| C18orf56 | -1.25249 | 7.984333 | -5.9167 | 1.22E-07 | 6.38E-07 | 7.187018 |
| CYP4B1 | 0.982726 | 6.979573 | 5.916541 | 1.22E-07 | 6.38E-07 | 7.186383 |
| NCSTN | -1.12521 | 9.107412 | -5.91578 | 1.22E-07 | 6.40E-07 | 7.183402 |
| ZNF142 | -0.85646 | 7.82613 | -5.91155 | 1.24E-07 | 6.50E-07 | 7.166941 |
| GOLT1A | -1.78596 | 8.43709 | -5.91148 | 1.24E-07 | 6.50E-07 | 7.166651 |
| SEC23A | -0.87366 | 7.292295 | -5.91126 | 1.24E-07 | 6.51E-07 | 7.16578 |
| LOC55423 | -1.78234 | 10.9123 | -5.91095 | 1.25E-07 | 6.51E-07 | 7.164579 |
| RNF144B | 1.203683 | 8.29616 | 5.910236 | 1.25E-07 | 6.53E-07 | 7.161802 |
| NDUFV2 | -0.9944 | 7.458157 | -5.90693 | 1.27E-07 | 6.61E-07 | 7.148924 |
| RPL7L1 | 1.079251 | 11.84613 | 5.906687 | 1.27E-07 | 6.62E-07 | 7.147969 |
| PAOX | -0.81426 | 7.680288 | -5.90521 | 1.28E-07 | 6.65E-07 | 7.142209 |
| AQP10 | 1.561644 | 7.790599 | 5.904633 | 1.28E-07 | 6.66E-07 | 7.139964 |
| ZSWIM1 | -0.82025 | 7.796007 | -5.90444 | 1.28E-07 | 6.67E-07 | 7.139224 |
| OR14J1 | 1.11493 | 7.069191 | 5.903273 | 1.29E-07 | 6.69E-07 | 7.134666 |
| NYNRIN | 1.77456 | 8.95906 | 5.902375 | 1.29E-07 | 6.71E-07 | 7.131167 |
| TMEM9B | -0.84212 | 7.632963 | -5.90153 | 1.29E-07 | 6.73E-07 | 7.127867 |
| CD300LB | 1.32253 | 7.388152 | 5.898497 | 1.31E-07 | 6.81E-07 | 7.116059 |
| KRTAP5-1 | 1.646847 | 7.72269 | 5.897553 | 1.32E-07 | 6.83E-07 | 7.11238 |
| COL6A1 | -0.88305 | 8.221421 | -5.89692 | 1.32E-07 | 6.85E-07 | 7.109914 |
| FAM78A | 0.828745 | 7.639652 | 5.893183 | 1.34E-07 | 6.94E-07 | 7.095363 |
| CGB | 0.940506 | 7.113627 | 5.892868 | 1.34E-07 | 6.94E-07 | 7.094135 |
| ALB | -2.18363 | 10.90246 | -5.8927 | 1.34E-07 | 6.95E-07 | 7.093474 |
| CAPZA2 | -0.93459 | 10.34367 | -5.89266 | 1.34E-07 | 6.95E-07 | 7.093323 |
| ACTG1 | -1.29426 | 8.991337 | -5.89172 | 1.35E-07 | 6.97E-07 | 7.089654 |
| LOC15153 | 0.953176 | 7.935971 | 5.891355 | 1.35E-07 | 6.97E-07 | 7.088244 |
| C9orf167 | 1.023299 | 8.223926 | 5.891195 | 1.35E-07 | 6.98E-07 | 7.08762 |
| FUT3 | 0.729819 | 6.997097 | 5.890864 | 1.35E-07 | 6.98E-07 | 7.08633 |
| KCNJ5 | 0.700228 | 7.246728 | 5.888533 | 1.36E-07 | 7.05E-07 | 7.077254 |
| NEK7 | -0.70711 | 7.532525 | -5.88815 | 1.37E-07 | 7.05E-07 | 7.075773 |
| NOS2 | 0.702736 | 7.120702 | 5.888114 | 1.37E-07 | 7.05E-07 | 7.075624 |
| FCER1G | -1.29914 | 8.844749 | -5.8879 | 1.37E-07 | 7.06E-07 | 7.074799 |
| RHBDL2 | 0.806269 | 7.360088 | 5.886702 | 1.37E-07 | 7.09E-07 | 7.070128 |
| GGH | -0.86649 | 7.450598 | -5.88635 | 1.38E-07 | 7.10E-07 | 7.068751 |
| LYRM1 | -1.32767 | 7.994515 | -5.88565 | 1.38E-07 | 7.11E-07 | 7.066035 |
| GADD45A | -0.9456 | 7.689947 | -5.88558 | 1.38E-07 | 7.11E-07 | 7.065752 |
| HIST1H1C | -0.75735 | 7.753427 | -5.88479 | 1.38E-07 | 7.13E-07 | 7.062675 |
| CCDC103 | 0.766262 | 7.063667 | 5.884048 | 1.39E-07 | 7.15E-07 | 7.059793 |
| TEX101 | 0.706331 | 6.803967 | 5.882605 | 1.40E-07 | 7.19E-07 | 7.054176 |
| TF | -1.72763 | 9.571647 | -5.88223 | 1.40E-07 | 7.20E-07 | 7.052734 |
| CPNE6 | 1.036476 | 7.258452 | 5.881815 | 1.40E-07 | 7.21E-07 | 7.051103 |
| TREML1 | 1.631009 | 7.703089 | 5.881538 | 1.40E-07 | 7.22E-07 | 7.050025 |
| CLDN3 | -0.95022 | 7.506739 | -5.88112 | 1.40E-07 | 7.23E-07 | 7.048387 |
| C7orf55 | -0.79603 | 7.312945 | -5.88101 | 1.40E-07 | 7.23E-07 | 7.047968 |
| GSTA1 | -1.36082 | 9.014131 | -5.8796 | 1.41E-07 | 7.26E-07 | 7.04247 |
| C11orf55 | 0.829688 | 7.156471 | 5.877469 | 1.42E-07 | 7.32E-07 | 7.034191 |
| ACCN1 | 1.384408 | 7.243468 | 5.875908 | 1.43E-07 | 7.36E-07 | 7.028116 |
| SFRS5 | -0.94422 | 9.724447 | -5.87524 | 1.44E-07 | 7.38E-07 | 7.025508 |
| PROS1 | -1.37823 | 8.638711 | -5.87379 | 1.45E-07 | 7.42E-07 | 7.019885 |
| PRLHR | 1.577875 | 7.287135 | 5.872864 | 1.45E-07 | 7.44E-07 | 7.016274 |
| GOT1 | -1.38925 | 8.785082 | -5.8725 | 1.45E-07 | 7.45E-07 | 7.014839 |

| | | | | | | |
|---|---|---|---|---|---|---|
| DUOXA1 | 1.030582 | 7.220325 | 5.871844 | 1.46E-07 | 7.46E-07 | 7.012308 |
| UGT2B17 | -1.03918 | 7.523163 | -5.8666 | 1.49E-07 | 7.61E-07 | 6.991925 |
| ANKFN1 | 1.116251 | 7.018496 | 5.865759 | 1.49E-07 | 7.63E-07 | 6.988639 |
| ZCCHC6 | -1.23085 | 8.633962 | -5.86563 | 1.49E-07 | 7.63E-07 | 6.98815 |
| OAF | -1.75381 | 8.978267 | -5.86367 | 1.51E-07 | 7.69E-07 | 6.980521 |
| CDS1 | 1.56789 | 8.322185 | 5.862969 | 1.51E-07 | 7.71E-07 | 6.977792 |
| CALY | 1.571805 | 7.631233 | 5.861744 | 1.52E-07 | 7.74E-07 | 6.973028 |
| UFC1 | -0.72351 | 7.719324 | -5.8616 | 1.52E-07 | 7.74E-07 | 6.972471 |
| PRPF6 | -0.76955 | 8.033505 | -5.86035 | 1.53E-07 | 7.78E-07 | 6.967604 |
| SNORA52 | -1.09181 | 9.151219 | -5.86011 | 1.53E-07 | 7.79E-07 | 6.96669 |
| OR5B3 | 1.276846 | 7.035217 | 5.859897 | 1.53E-07 | 7.79E-07 | 6.965846 |
| FAM74A3 | 1.077844 | 7.166656 | 5.858383 | 1.54E-07 | 7.83E-07 | 6.959962 |
| KIAA1310 | -1.54901 | 9.219663 | -5.8583 | 1.54E-07 | 7.83E-07 | 6.959644 |
| TMED10 | -0.82735 | 7.567588 | -5.85504 | 1.56E-07 | 7.93E-07 | 6.946952 |
| CTF1 | 2.096557 | 8.85143 | 5.854394 | 1.56E-07 | 7.95E-07 | 6.94446 |
| OR10P1 | 0.800431 | 6.858248 | 5.854107 | 1.56E-07 | 7.95E-07 | 6.943342 |
| DAZ3 | 1.439474 | 7.317094 | 5.851685 | 1.58E-07 | 8.03E-07 | 6.933931 |
| RIN1 | 0.998424 | 7.585131 | 5.85134 | 1.58E-07 | 8.03E-07 | 6.93259 |
| NANOS2 | 2.045765 | 8.07587 | 5.847914 | 1.60E-07 | 8.14E-07 | 6.919279 |
| OR4X1 | 0.725832 | 6.859203 | 5.84416 | 1.63E-07 | 8.26E-07 | 6.904696 |
| SNORA2B | -1.46554 | 9.58877 | -5.84397 | 1.63E-07 | 8.26E-07 | 6.903977 |
| ABL1 | -0.73847 | 7.586605 | -5.84376 | 1.63E-07 | 8.26E-07 | 6.903153 |
| SLC25A22 | -1.38305 | 8.5438 | -5.84321 | 1.63E-07 | 8.28E-07 | 6.901022 |
| COX15 | 0.8078 | 8.30604 | 5.841625 | 1.64E-07 | 8.33E-07 | 6.894852 |
| GCK | 1.353616 | 7.52978 | 5.841246 | 1.65E-07 | 8.34E-07 | 6.893378 |
| EXOC2 | 1.009728 | 8.095038 | 5.839409 | 1.66E-07 | 8.40E-07 | 6.886244 |
| CCL24 | 1.671765 | 7.711608 | 5.838329 | 1.67E-07 | 8.43E-07 | 6.882054 |
| ZDBF2 | 0.935771 | 7.323741 | 5.837446 | 1.67E-07 | 8.46E-07 | 6.878624 |
| LEMD1 | 0.835956 | 6.887604 | 5.837323 | 1.67E-07 | 8.46E-07 | 6.878145 |
| HCN2 | 2.031092 | 8.548501 | 5.83715 | 1.67E-07 | 8.46E-07 | 6.877475 |
| IL18BP | -1.15916 | 8.155157 | -5.83704 | 1.67E-07 | 8.47E-07 | 6.877052 |
| C1orf198 | -0.94866 | 8.284514 | -5.83525 | 1.69E-07 | 8.52E-07 | 6.870105 |
| WNT1 | 1.088343 | 7.067151 | 5.834567 | 1.69E-07 | 8.54E-07 | 6.867445 |
| SCO2 | -0.75513 | 7.816661 | -5.83337 | 1.70E-07 | 8.58E-07 | 6.86281 |
| C17orf45 | -1.23736 | 8.732341 | -5.83255 | 1.70E-07 | 8.60E-07 | 6.859607 |
| CSF1R | -1.31782 | 8.120782 | -5.83253 | 1.70E-07 | 8.60E-07 | 6.85954 |
| COL4A1 | -1.54762 | 8.349076 | -5.8306 | 1.72E-07 | 8.66E-07 | 6.852058 |
| ACPT | 1.727663 | 7.995723 | 5.830085 | 1.72E-07 | 8.67E-07 | 6.850049 |
| POLR3B | -1.10025 | 8.169418 | -5.82916 | 1.73E-07 | 8.70E-07 | 6.846456 |
| UBE3A | -0.81528 | 7.704231 | -5.82851 | 1.73E-07 | 8.72E-07 | 6.843954 |
| FAM184B | 0.996914 | 7.169458 | 5.826744 | 1.74E-07 | 8.77E-07 | 6.837082 |
| C3orf27 | 1.035074 | 7.057553 | 5.825322 | 1.75E-07 | 8.82E-07 | 6.831564 |
| SLC6A5 | 1.425719 | 7.375181 | 5.823999 | 1.76E-07 | 8.86E-07 | 6.826433 |
| PFKFB1 | -1.02076 | 8.01988 | -5.82345 | 1.77E-07 | 8.88E-07 | 6.824287 |
| FSCN3 | 1.132927 | 7.130755 | 5.82241 | 1.77E-07 | 8.91E-07 | 6.820268 |
| SCRT1 | 1.318529 | 7.146434 | 5.821469 | 1.78E-07 | 8.94E-07 | 6.816615 |
| C19orf44 | -1.11135 | 8.786244 | -5.82073 | 1.79E-07 | 8.97E-07 | 6.813748 |
| DCAF6 | -1.44214 | 9.007008 | -5.81931 | 1.80E-07 | 9.01E-07 | 6.808243 |
| MRPS15 | -1.00089 | 8.341349 | -5.81811 | 1.80E-07 | 9.05E-07 | 6.803568 |
| RNASE7 | 1.617452 | 7.720225 | 5.817117 | 1.81E-07 | 9.08E-07 | 6.799734 |
| UNC93A | 1.564942 | 8.464887 | 5.817102 | 1.81E-07 | 9.08E-07 | 6.799678 |
| BUB3 | -0.80586 | 7.415015 | -5.81681 | 1.81E-07 | 9.09E-07 | 6.798562 |
| SLC25A12 | 0.920816 | 7.552376 | 5.815876 | 1.82E-07 | 9.12E-07 | 6.794923 |
| GSDMC | 0.938074 | 7.107612 | 5.814836 | 1.83E-07 | 9.15E-07 | 6.790888 |
| ZNF473 | 0.886559 | 7.687249 | 5.813163 | 1.84E-07 | 9.21E-07 | 6.784401 |
| NDUFA4 | -1.50141 | 8.429784 | -5.81048 | 1.86E-07 | 9.30E-07 | 6.774016 |
| LOC44092 | -0.9382 | 7.666952 | -5.80818 | 1.88E-07 | 9.38E-07 | 6.765067 |
| YPEL1 | 1.016407 | 7.640909 | 5.806206 | 1.89E-07 | 9.44E-07 | 6.757431 |

| | | | | | | |
|---|---|---|---|---|---|---|
| UROD | -0.99804 | 8.079431 | -5.80585 | 1.90E-07 | 9.46E-07 | 6.756048 |
| EXOSC7 | -0.76373 | 7.132521 | -5.80411 | 1.91E-07 | 9.51E-07 | 6.749317 |
| SLC22A6 | 1.15545 | 7.143673 | 5.802055 | 1.92E-07 | 9.58E-07 | 6.741343 |
| MYH13 | 0.962324 | 7.005265 | 5.802016 | 1.92E-07 | 9.58E-07 | 6.741191 |
| BEND6 | 0.84611 | 7.037222 | 5.80159 | 1.93E-07 | 9.59E-07 | 6.739542 |
| C9orf163 | 1.06492 | 7.226242 | 5.801204 | 1.93E-07 | 9.60E-07 | 6.738046 |
| SPRN | 0.83321 | 7.033712 | 5.801044 | 1.93E-07 | 9.61E-07 | 6.737426 |
| USH1G | 0.8149 | 7.326822 | 5.799955 | 1.94E-07 | 9.64E-07 | 6.733208 |
| MICA | -0.86899 | 7.160781 | -5.79951 | 1.94E-07 | 9.66E-07 | 6.731471 |
| OPCML | 1.077809 | 7.058189 | 5.798312 | 1.95E-07 | 9.70E-07 | 6.726842 |
| LOC64734 | -0.78437 | 7.976561 | -5.79775 | 1.96E-07 | 9.71E-07 | 6.724649 |
| KIRREL3 | 1.687337 | 7.741445 | 5.795605 | 1.97E-07 | 9.79E-07 | 6.716354 |
| THEM4 | 1.187706 | 7.660638 | 5.795521 | 1.97E-07 | 9.79E-07 | 6.716029 |
| DDX5 | -0.84415 | 7.496018 | -5.79431 | 1.98E-07 | 9.83E-07 | 6.711329 |
| HLA-DPA1 | -1.19023 | 9.159395 | -5.79317 | 1.99E-07 | 9.87E-07 | 6.70693 |
| GAGE12C | 2.423439 | 8.546851 | 5.792566 | 2.00E-07 | 9.89E-07 | 6.704582 |
| ZNF519 | 0.787293 | 7.246599 | 5.790713 | 2.01E-07 | 9.95E-07 | 6.697407 |
| DAP | -1.17507 | 8.735184 | -5.79064 | 2.01E-07 | 9.95E-07 | 6.697136 |
| PSMB10 | -1.50262 | 8.974897 | -5.78898 | 2.03E-07 | 1.00E-06 | 6.69068 |
| CREB1 | 0.795875 | 7.705749 | 5.788475 | 2.03E-07 | 1.00E-06 | 6.688738 |
| OR4F15 | 0.741013 | 6.827871 | 5.787811 | 2.04E-07 | 1.01E-06 | 6.686167 |
| BRMS1 | -0.95877 | 9.42559 | -5.78536 | 2.06E-07 | 1.01E-06 | 6.676661 |
| POU4F2 | 1.400192 | 7.221312 | 5.78497 | 2.06E-07 | 1.02E-06 | 6.67517 |
| FMO3 | -1.06584 | 8.364705 | -5.7846 | 2.06E-07 | 1.02E-06 | 6.673728 |
| HSD17B10 | -1.20005 | 8.962368 | -5.78232 | 2.08E-07 | 1.03E-06 | 6.664904 |
| RALB | -0.80076 | 8.269439 | -5.78226 | 2.08E-07 | 1.03E-06 | 6.664683 |
| TNKS | -0.75085 | 7.764885 | -5.78207 | 2.08E-07 | 1.03E-06 | 6.663924 |
| TTC38 | -0.76366 | 7.193129 | -5.7815 | 2.09E-07 | 1.03E-06 | 6.661717 |
| RPS8 | -0.88339 | 7.670853 | -5.78103 | 2.09E-07 | 1.03E-06 | 6.659898 |
| POLD2 | -0.74507 | 7.302963 | -5.77676 | 2.13E-07 | 1.05E-06 | 6.643376 |
| FGF3 | 1.269548 | 7.042527 | 5.776529 | 2.13E-07 | 1.05E-06 | 6.642497 |
| FLJ46010 | 0.903898 | 6.976501 | 5.775742 | 2.14E-07 | 1.05E-06 | 6.639453 |
| CMBL | -1.35208 | 8.70307 | -5.77516 | 2.14E-07 | 1.05E-06 | 6.637186 |
| OR4A5 | 1.006177 | 6.978939 | 5.774769 | 2.14E-07 | 1.05E-06 | 6.635687 |
| RNF190 | 1.036117 | 6.926795 | 5.773888 | 2.15E-07 | 1.06E-06 | 6.632281 |
| BCAR4 | 0.776344 | 6.896758 | 5.773725 | 2.15E-07 | 1.06E-06 | 6.631648 |
| DUOX1 | 0.761944 | 7.130267 | 5.773328 | 2.16E-07 | 1.06E-06 | 6.630112 |
| CACNG3 | 1.029608 | 7.003126 | 5.770291 | 2.18E-07 | 1.07E-06 | 6.618363 |
| MIR886 | -1.31181 | 9.743153 | -5.77022 | 2.18E-07 | 1.07E-06 | 6.618098 |
| RPL18A | -0.92347 | 11.88737 | -5.76897 | 2.19E-07 | 1.08E-06 | 6.61327 |
| LOC38852 | -0.87666 | 8.718783 | -5.76894 | 2.19E-07 | 1.08E-06 | 6.613139 |
| CCDC39 | 1.037821 | 7.026711 | 5.768443 | 2.20E-07 | 1.08E-06 | 6.611217 |
| FAM113B | -0.98708 | 7.933431 | -5.76667 | 2.21E-07 | 1.08E-06 | 6.604353 |
| ZFAND2A | -1.11766 | 9.866641 | -5.76551 | 2.22E-07 | 1.09E-06 | 6.599865 |
| BCLAF1 | -0.90478 | 8.42118 | -5.76541 | 2.23E-07 | 1.09E-06 | 6.599489 |
| DDX54 | 1.668301 | 8.462843 | 5.764562 | 2.23E-07 | 1.09E-06 | 6.596208 |
| CLDN17 | 0.955313 | 6.910021 | 5.764314 | 2.23E-07 | 1.09E-06 | 6.595249 |
| IL3 | 1.461435 | 7.295077 | 5.764281 | 2.24E-07 | 1.09E-06 | 6.59512 |
| FBXL6 | -1.13403 | 8.73846 | -5.76378 | 2.24E-07 | 1.10E-06 | 6.59318 |
| HECTD3 | -1.08477 | 9.131893 | -5.76341 | 2.24E-07 | 1.10E-06 | 6.591767 |
| GUCY1B2 | 1.152991 | 7.296343 | 5.763387 | 2.24E-07 | 1.10E-06 | 6.591663 |
| HIST1H4H | -1.2657 | 9.020653 | -5.76152 | 2.26E-07 | 1.10E-06 | 6.584457 |
| SULT2A1 | -0.88384 | 7.434112 | -5.76121 | 2.26E-07 | 1.11E-06 | 6.583258 |
| S100A10 | -0.97969 | 8.104462 | -5.75922 | 2.28E-07 | 1.11E-06 | 6.575547 |
| ARHGAP1 | 0.836922 | 7.474852 | 5.758739 | 2.28E-07 | 1.12E-06 | 6.573692 |
| ZNF324 | 1.401706 | 7.9071 | 5.758108 | 2.29E-07 | 1.12E-06 | 6.571255 |
| EIF3D | -1.1721 | 9.100177 | -5.75721 | 2.30E-07 | 1.12E-06 | 6.567773 |
| LOXL4 | -1.56542 | 8.339478 | -5.75512 | 2.32E-07 | 1.13E-06 | 6.559718 |

| | | | | | | |
|---|---|---|---|---|---|---|
| SNORD12 | -0.84926 | 7.534182 | -5.75505 | 2.32E-07 | 1.13E-06 | 6.559425 |
| KRT9 | 1.028851 | 7.417511 | 5.751605 | 2.35E-07 | 1.14E-06 | 6.546123 |
| MRGPRX1 | 0.71491 | 6.706277 | 5.749707 | 2.37E-07 | 1.15E-06 | 6.538787 |
| C5orf15 | -1.09217 | 7.910509 | -5.7485 | 2.38E-07 | 1.16E-06 | 6.534113 |
| ERP29 | -1.27388 | 8.88735 | -5.74785 | 2.39E-07 | 1.16E-06 | 6.531629 |
| CYBA | -1.46557 | 7.99549 | -5.74647 | 2.40E-07 | 1.17E-06 | 6.526269 |
| F12 | -1.83381 | 9.429038 | -5.74636 | 2.40E-07 | 1.17E-06 | 6.525861 |
| IMP4 | -1.21393 | 8.974619 | -5.74565 | 2.41E-07 | 1.17E-06 | 6.523128 |
| FKBP11 | -1.01546 | 7.583492 | -5.74451 | 2.42E-07 | 1.17E-06 | 6.518714 |
| DDI2 | -0.78072 | 7.509353 | -5.7426 | 2.44E-07 | 1.18E-06 | 6.511334 |
| GPR18 | 0.747696 | 6.993279 | 5.740966 | 2.45E-07 | 1.19E-06 | 6.505026 |
| PARK7 | -1.17642 | 8.786096 | -5.74011 | 2.46E-07 | 1.19E-06 | 6.501711 |
| AFM | -1.11127 | 7.461165 | -5.73862 | 2.47E-07 | 1.20E-06 | 6.495981 |
| PLXNB1 | -1.23314 | 9.247571 | -5.73844 | 2.48E-07 | 1.20E-06 | 6.495264 |
| PLAC8 | 0.787969 | 7.395033 | 5.738083 | 2.48E-07 | 1.20E-06 | 6.493893 |
| MLL2 | -0.97607 | 8.179597 | -5.73734 | 2.49E-07 | 1.20E-06 | 6.49104 |
| DIO2 | 0.777627 | 7.167534 | 5.736535 | 2.50E-07 | 1.21E-06 | 6.487919 |
| SNHG11 | -1.03733 | 8.349457 | -5.73514 | 2.51E-07 | 1.21E-06 | 6.48252 |
| APOE | -2.06163 | 11.38695 | -5.73261 | 2.53E-07 | 1.22E-06 | 6.472761 |
| BDH1 | -0.89176 | 7.67601 | -5.7323 | 2.54E-07 | 1.22E-06 | 6.471574 |
| OR1D4 | 0.709366 | 6.842625 | 5.731523 | 2.55E-07 | 1.23E-06 | 6.468572 |
| ZBTB10 | -0.73768 | 7.248453 | -5.73118 | 2.55E-07 | 1.23E-06 | 6.46724 |
| ARPC3 | -0.88765 | 8.486536 | -5.7304 | 2.56E-07 | 1.23E-06 | 6.46425 |
| CAPN5 | -1.56339 | 9.395206 | -5.73032 | 2.56E-07 | 1.23E-06 | 6.463939 |
| RBM42 | -1.21153 | 9.813668 | -5.73013 | 2.56E-07 | 1.23E-06 | 6.46321 |
| SDS | -1.8246 | 8.708482 | -5.72823 | 2.58E-07 | 1.24E-06 | 6.455876 |
| PSMC2 | -1.14854 | 8.608196 | -5.7281 | 2.58E-07 | 1.24E-06 | 6.455358 |
| LYPLA1 | -0.77522 | 7.708022 | -5.72801 | 2.58E-07 | 1.24E-06 | 6.455021 |
| ARHGEF18 | -0.99434 | 7.746769 | -5.72794 | 2.58E-07 | 1.24E-06 | 6.454737 |
| SNORD15 | -1.06589 | 7.728559 | -5.72709 | 2.59E-07 | 1.24E-06 | 6.451476 |
| FLRT1 | 0.927516 | 7.036845 | 5.726204 | 2.60E-07 | 1.25E-06 | 6.448045 |
| ACSM2B | -1.45471 | 8.847994 | -5.72598 | 2.60E-07 | 1.25E-06 | 6.447195 |
| LRP4 | 1.71699 | 8.097979 | 5.725005 | 2.61E-07 | 1.25E-06 | 6.443421 |
| BIRC7 | 0.712075 | 6.850538 | 5.721898 | 2.64E-07 | 1.27E-06 | 6.431435 |
| IRS1 | -0.79037 | 7.704238 | -5.72016 | 2.66E-07 | 1.27E-06 | 6.424748 |
| EFS | 0.713277 | 7.104377 | 5.713606 | 2.73E-07 | 1.30E-06 | 6.399457 |
| FAM153A | 0.901232 | 6.994567 | 5.713237 | 2.74E-07 | 1.31E-06 | 6.398034 |
| CLNS1A | -0.73587 | 7.686109 | -5.71261 | 2.74E-07 | 1.31E-06 | 6.395626 |
| DEFA3 | 1.005438 | 7.109299 | 5.711633 | 2.75E-07 | 1.31E-06 | 6.391848 |
| SLC47A2 | 0.874956 | 6.906086 | 5.711327 | 2.76E-07 | 1.31E-06 | 6.390669 |
| ZFHX2 | 1.411486 | 7.651722 | 5.710949 | 2.76E-07 | 1.32E-06 | 6.389212 |
| LCE3A | 1.094338 | 7.096127 | 5.710377 | 2.77E-07 | 1.32E-06 | 6.387008 |
| GAGE2B | 1.425164 | 7.639706 | 5.709406 | 2.78E-07 | 1.32E-06 | 6.383264 |
| VPS28 | -0.94569 | 9.145573 | -5.70842 | 2.79E-07 | 1.33E-06 | 6.37947 |
| OR4D9 | 1.553855 | 7.316305 | 5.707622 | 2.80E-07 | 1.33E-06 | 6.376389 |
| MRLC2 | -1.36769 | 8.426992 | -5.70691 | 2.81E-07 | 1.33E-06 | 6.373631 |
| MYCN | 0.743645 | 7.072939 | 5.70553 | 2.82E-07 | 1.34E-06 | 6.368326 |
| CCDC114 | 2.031792 | 8.065315 | 5.705187 | 2.82E-07 | 1.34E-06 | 6.367005 |
| HFE2 | -1.29919 | 8.696435 | -5.70448 | 2.83E-07 | 1.35E-06 | 6.364292 |
| FCF1 | 0.756083 | 8.486451 | 5.70314 | 2.85E-07 | 1.35E-06 | 6.359116 |
| C9orf100 | 0.726813 | 7.167303 | 5.703037 | 2.85E-07 | 1.35E-06 | 6.358721 |
| FAM54A | 0.938777 | 7.614449 | 5.702502 | 2.86E-07 | 1.36E-06 | 6.35666 |
| SF4 | 1.261565 | 8.4209 | 5.70175 | 2.86E-07 | 1.36E-06 | 6.353761 |
| SLC6A18 | 0.806783 | 6.827368 | 5.698894 | 2.90E-07 | 1.37E-06 | 6.342758 |
| SNORA33 | -1.07789 | 7.835471 | -5.69767 | 2.91E-07 | 1.38E-06 | 6.338034 |
| ZBTB32 | 0.843878 | 7.17552 | 5.696731 | 2.92E-07 | 1.38E-06 | 6.33443 |
| CUZD1 | 0.717215 | 6.937707 | 5.69593 | 2.93E-07 | 1.39E-06 | 6.331345 |
| ZNF395 | -0.87188 | 7.878838 | -5.69357 | 2.96E-07 | 1.40E-06 | 6.322263 |

| | | | | | | |
|---|---|---|---|---|---|---|
| OSBPL6 | 0.933582 | 7.275123 | 5.693007 | 2.96E-07 | 1.40E-06 | 6.320087 |
| TM7SF3 | -0.78096 | 7.998983 | -5.69272 | 2.97E-07 | 1.40E-06 | 6.318986 |
| TAP1 | -1.36106 | 8.606499 | -5.68854 | 3.02E-07 | 1.43E-06 | 6.302904 |
| LILRB2 | 0.998908 | 8.202613 | 5.685953 | 3.05E-07 | 1.44E-06 | 6.292931 |
| FLJ14816 | 1.156081 | 7.074509 | 5.684017 | 3.07E-07 | 1.45E-06 | 6.285479 |
| ATP5J | -1.08777 | 9.232152 | -5.68311 | 3.08E-07 | 1.45E-06 | 6.281988 |
| FOXP4 | 1.521292 | 7.933833 | 5.682895 | 3.09E-07 | 1.45E-06 | 6.281161 |
| EVL | -1.69163 | 9.461721 | -5.68264 | 3.09E-07 | 1.46E-06 | 6.280182 |
| SPRY2 | -0.74499 | 7.202869 | -5.68256 | 3.09E-07 | 1.46E-06 | 6.279878 |
| TUBB2B | 1.606843 | 8.660202 | 5.68143 | 3.10E-07 | 1.46E-06 | 6.275522 |
| RPS3 | -1.15637 | 9.271685 | -5.68082 | 3.11E-07 | 1.46E-06 | 6.273193 |
| CCDC107 | -1.42078 | 7.917035 | -5.68014 | 3.12E-07 | 1.47E-06 | 6.270572 |
| LENG8 | -0.98114 | 9.436917 | -5.67947 | 3.13E-07 | 1.47E-06 | 6.267982 |
| ATL2 | -0.79261 | 7.738641 | -5.67804 | 3.15E-07 | 1.48E-06 | 6.262467 |
| TAPBPL | -0.73336 | 7.353554 | -5.67699 | 3.16E-07 | 1.48E-06 | 6.258446 |
| TMTC3 | 0.906388 | 6.960685 | 5.676667 | 3.16E-07 | 1.49E-06 | 6.257197 |
| AMDHD1 | -1.11496 | 7.922614 | -5.6749 | 3.18E-07 | 1.50E-06 | 6.250404 |
| MDH2 | -1.45855 | 9.531998 | -5.67058 | 3.24E-07 | 1.52E-06 | 6.233777 |
| GUSB | -0.88185 | 7.829885 | -5.66574 | 3.30E-07 | 1.54E-06 | 6.215167 |
| NGEF | -0.83946 | 7.518763 | -5.66572 | 3.30E-07 | 1.54E-06 | 6.215093 |
| MYO1A | 0.987985 | 7.713324 | 5.665564 | 3.30E-07 | 1.54E-06 | 6.214499 |
| U2AF1L2 | -0.92647 | 7.581594 | -5.66395 | 3.32E-07 | 1.55E-06 | 6.208286 |
| CHSY1 | -0.88772 | 7.25572 | -5.66388 | 3.33E-07 | 1.55E-06 | 6.208027 |
| DLX1 | 0.783492 | 7.051803 | 5.662682 | 3.34E-07 | 1.56E-06 | 6.203423 |
| ATG7 | -1.03372 | 8.413162 | -5.66268 | 3.34E-07 | 1.56E-06 | 6.203411 |
| FSCN1 | -1.20111 | 7.905047 | -5.66178 | 3.35E-07 | 1.56E-06 | 6.199963 |
| SPTBN1 | -1.0422 | 9.656443 | -5.66134 | 3.36E-07 | 1.57E-06 | 6.198252 |
| RAB11FIP3 | -0.73005 | 7.210399 | -5.66122 | 3.36E-07 | 1.57E-06 | 6.197802 |
| TNFRSF13 | 0.880455 | 7.402002 | 5.659103 | 3.39E-07 | 1.58E-06 | 6.189668 |
| SLC13A5 | -2.20946 | 9.921301 | -5.65902 | 3.39E-07 | 1.58E-06 | 6.189361 |
| DLK1 | 0.838261 | 6.883517 | 5.658097 | 3.40E-07 | 1.58E-06 | 6.1858 |
| PLA2G4F | 0.870578 | 6.835575 | 5.657524 | 3.41E-07 | 1.59E-06 | 6.183601 |
| TP53INP1 | -0.91448 | 7.702864 | -5.65748 | 3.41E-07 | 1.59E-06 | 6.183414 |
| ADCY3 | -0.75932 | 7.045441 | -5.65666 | 3.42E-07 | 1.59E-06 | 6.18027 |
| FNDC8 | 1.480877 | 7.522887 | 5.655795 | 3.43E-07 | 1.60E-06 | 6.176957 |
| NFIL3 | -0.97314 | 8.147887 | -5.65542 | 3.44E-07 | 1.60E-06 | 6.175517 |
| C6orf138 | 0.996719 | 7.780357 | 5.654569 | 3.45E-07 | 1.60E-06 | 6.172246 |
| NAT5 | -0.85264 | 9.431566 | -5.65427 | 3.45E-07 | 1.60E-06 | 6.171098 |
| NMRAL1 | -0.81035 | 8.061833 | -5.65426 | 3.45E-07 | 1.60E-06 | 6.171044 |
| C1QL4 | 2.064061 | 8.050598 | 5.654012 | 3.46E-07 | 1.60E-06 | 6.170105 |
| MFGE8 | -1.74246 | 9.62487 | -5.65307 | 3.47E-07 | 1.61E-06 | 6.16649 |
| NTNG2 | 1.904615 | 8.345789 | 5.653036 | 3.47E-07 | 1.61E-06 | 6.16636 |
| IMMP2L | -0.89039 | 8.255855 | -5.6527 | 3.48E-07 | 1.61E-06 | 6.165059 |
| FRMPD4 | 1.162837 | 7.09792 | 5.652016 | 3.49E-07 | 1.61E-06 | 6.16244 |
| GNB3 | 1.021069 | 7.188218 | 5.650392 | 3.51E-07 | 1.62E-06 | 6.156201 |
| DYTN | 0.937165 | 6.971347 | 5.648948 | 3.53E-07 | 1.63E-06 | 6.150657 |
| RPS28 | -1.37155 | 9.899228 | -5.64855 | 3.53E-07 | 1.63E-06 | 6.149137 |
| MFSD2B | 0.855554 | 6.950359 | 5.642861 | 3.61E-07 | 1.67E-06 | 6.127285 |
| DDX11 | 0.863507 | 7.76449 | 5.642846 | 3.61E-07 | 1.67E-06 | 6.127226 |
| AZI1 | -0.94429 | 8.633223 | -5.64043 | 3.65E-07 | 1.68E-06 | 6.117969 |
| PRSS42 | 1.178596 | 7.867186 | 5.640115 | 3.65E-07 | 1.68E-06 | 6.116744 |
| CPLX3 | 1.701891 | 7.71567 | 5.639222 | 3.67E-07 | 1.69E-06 | 6.113315 |
| LRRC42 | -0.93855 | 8.623119 | -5.63698 | 3.70E-07 | 1.70E-06 | 6.104727 |
| HBG1 | 1.027972 | 7.097348 | 5.634795 | 3.73E-07 | 1.72E-06 | 6.096328 |
| GFRA3 | 1.090979 | 7.153812 | 5.634354 | 3.74E-07 | 1.72E-06 | 6.094636 |
| SLC35F5 | -0.73553 | 7.376529 | -5.6337 | 3.75E-07 | 1.72E-06 | 6.092118 |
| PLEKHB1 | 1.351744 | 8.260535 | 5.633615 | 3.75E-07 | 1.72E-06 | 6.091799 |
| TOX | 0.798728 | 6.981046 | 5.633085 | 3.76E-07 | 1.72E-06 | 6.089766 |

| | | | | | | |
|---|---|---|---|---|---|---|
| GATA1 | 0.74153 | 6.817159 | 5.630908 | 3.79E-07 | 1.74E-06 | 6.081416 |
| PCBD1 | -0.85183 | 8.159981 | -5.63087 | 3.79E-07 | 1.74E-06 | 6.081251 |
| SLC24A6 | -0.81583 | 7.774656 | -5.63079 | 3.79E-07 | 1.74E-06 | 6.080957 |
| CELSR1 | 1.073099 | 7.176717 | 5.630422 | 3.79E-07 | 1.74E-06 | 6.079552 |
| SLC25A27 | -0.71675 | 7.486213 | -5.62899 | 3.82E-07 | 1.75E-06 | 6.07406 |
| FHOD3 | 1.049999 | 7.451476 | 5.627777 | 3.83E-07 | 1.76E-06 | 6.069407 |
| ITIH5 | 0.886361 | 10.16676 | 5.627714 | 3.84E-07 | 1.76E-06 | 6.069166 |
| LOC72811 | 0.786411 | 6.818171 | 5.626443 | 3.85E-07 | 1.77E-06 | 6.06429 |
| ITGB5 | -0.71696 | 7.975346 | -5.6258 | 3.86E-07 | 1.77E-06 | 6.061811 |
| CRLF3 | -0.72989 | 7.502676 | -5.6238 | 3.90E-07 | 1.78E-06 | 6.054165 |
| OTC | -1.58969 | 9.247716 | -5.62212 | 3.92E-07 | 1.79E-06 | 6.047699 |
| C2CD3 | 1.205442 | 8.8983 | 5.620803 | 3.94E-07 | 1.80E-06 | 6.042665 |
| MYL3 | 1.738279 | 8.263531 | 5.619023 | 3.97E-07 | 1.81E-06 | 6.03584 |
| NID1 | -0.83962 | 8.431344 | -5.61407 | 4.05E-07 | 1.85E-06 | 6.016867 |
| SDHB | -0.76777 | 10.57282 | -5.61215 | 4.08E-07 | 1.86E-06 | 6.009498 |
| GPX8 | 1.400097 | 9.649732 | 5.611685 | 4.09E-07 | 1.86E-06 | 6.00772 |
| PRTG | 1.143226 | 7.240641 | 5.610897 | 4.10E-07 | 1.87E-06 | 6.004702 |
| ARHGEF17 | 1.222478 | 9.407871 | 5.608324 | 4.14E-07 | 1.88E-06 | 5.994844 |
| APP | -0.97111 | 8.259105 | -5.60771 | 4.15E-07 | 1.89E-06 | 5.992499 |
| PAFAH2 | 0.838663 | 8.825991 | 5.605156 | 4.19E-07 | 1.91E-06 | 5.982709 |
| DDIT4 | -0.96403 | 7.697061 | -5.60472 | 4.20E-07 | 1.91E-06 | 5.981051 |
| SYT6 | 1.107469 | 7.169686 | 5.602095 | 4.24E-07 | 1.93E-06 | 5.970985 |
| C22orf13 | -1.11915 | 8.767979 | -5.60204 | 4.24E-07 | 1.93E-06 | 5.970759 |
| SLC13A2 | 1.637853 | 8.033558 | 5.601131 | 4.26E-07 | 1.94E-06 | 5.967295 |
| DBH | 1.717679 | 8.585924 | 5.601039 | 4.26E-07 | 1.94E-06 | 5.966944 |
| APBA2 | 0.902579 | 7.023012 | 5.600963 | 4.26E-07 | 1.94E-06 | 5.966651 |
| C7orf52 | 1.77045 | 7.725871 | 5.599987 | 4.28E-07 | 1.94E-06 | 5.962915 |
| RILPL1 | -1.04827 | 8.289029 | -5.59767 | 4.32E-07 | 1.96E-06 | 5.954049 |
| LOC15476 | 1.266424 | 8.283282 | 5.596757 | 4.33E-07 | 1.96E-06 | 5.950549 |
| KCNT1 | 0.91967 | 7.119448 | 5.596093 | 4.34E-07 | 1.97E-06 | 5.948009 |
| OTOS | 0.978566 | 6.920121 | 5.594721 | 4.37E-07 | 1.98E-06 | 5.942756 |
| SPCS1 | -1.47686 | 10.47998 | -5.59343 | 4.39E-07 | 1.99E-06 | 5.937812 |
| NXT2 | -0.74394 | 7.220689 | -5.58876 | 4.47E-07 | 2.02E-06 | 5.919941 |
| SRP72 | -0.758 | 8.601673 | -5.58859 | 4.47E-07 | 2.02E-06 | 5.919291 |
| ZNF334 | 0.721874 | 7.121266 | 5.58778 | 4.49E-07 | 2.03E-06 | 5.916197 |
| DGUOK | -0.9772 | 8.327504 | -5.58726 | 4.50E-07 | 2.03E-06 | 5.914206 |
| AUTS2 | -0.85435 | 7.51805 | -5.58404 | 4.55E-07 | 2.05E-06 | 5.901906 |
| E4F1 | -1.57587 | 8.156706 | -5.5828 | 4.58E-07 | 2.06E-06 | 5.897167 |
| TXN | -1.25194 | 8.568756 | -5.58231 | 4.59E-07 | 2.07E-06 | 5.89529 |
| HIST2H2B | 0.839606 | 7.400037 | 5.581054 | 4.61E-07 | 2.08E-06 | 5.890473 |
| SERP2 | 1.230368 | 7.582071 | 5.580024 | 4.63E-07 | 2.09E-06 | 5.886533 |
| FLJ41423 | 0.718136 | 6.778287 | 5.579009 | 4.65E-07 | 2.09E-06 | 5.882654 |
| ATP5L | -0.84892 | 9.028062 | -5.57498 | 4.72E-07 | 2.12E-06 | 5.867258 |
| SLC26A5 | 0.762179 | 6.959438 | 5.574578 | 4.73E-07 | 2.13E-06 | 5.865715 |
| A3GALT2 | 0.708433 | 6.862031 | 5.572351 | 4.77E-07 | 2.14E-06 | 5.857204 |
| FGL2 | -0.71618 | 7.700546 | -5.57173 | 4.78E-07 | 2.15E-06 | 5.854823 |
| ZC3HC1 | -1.19999 | 7.796813 | -5.57123 | 4.79E-07 | 2.15E-06 | 5.85293 |
| RASSF4 | -0.95601 | 8.129312 | -5.57094 | 4.80E-07 | 2.15E-06 | 5.851827 |
| SMR3B | 1.156905 | 7.278055 | 5.569736 | 4.82E-07 | 2.16E-06 | 5.847208 |
| LEPR | -0.93977 | 7.66719 | -5.56922 | 4.83E-07 | 2.17E-06 | 5.845241 |
| TOMM7 | -2.52441 | 10.58148 | -5.56826 | 4.85E-07 | 2.17E-06 | 5.841588 |
| LEAP2 | -1.24502 | 7.964925 | -5.56805 | 4.85E-07 | 2.18E-06 | 5.840777 |
| ASAH2B | -0.81033 | 7.072533 | -5.56794 | 4.85E-07 | 2.18E-06 | 5.840329 |
| ADRBK1 | -0.80963 | 8.102696 | -5.56547 | 4.90E-07 | 2.20E-06 | 5.830892 |
| AKAP8L | -1.36617 | 8.106757 | -5.56445 | 4.92E-07 | 2.20E-06 | 5.827017 |
| PROKR1 | 1.552447 | 7.23752 | 5.563924 | 4.93E-07 | 2.21E-06 | 5.825006 |
| TMC7 | 1.000935 | 7.437514 | 5.563586 | 4.94E-07 | 2.21E-06 | 5.823714 |
| AZU1 | 1.107193 | 7.090859 | 5.562118 | 4.96E-07 | 2.22E-06 | 5.818108 |

| | | | | | | |
|---|---|---|---|---|---|---|
| UGCG | -0.75759 | 7.800441 | -5.56162 | 4.97E-07 | 2.22E-06 | 5.816222 |
| MIR196A2 | 0.878739 | 6.915209 | 5.561157 | 4.98E-07 | 2.23E-06 | 5.814435 |
| NCR1 | 0.805654 | 7.061933 | 5.55986 | 5.01E-07 | 2.24E-06 | 5.809483 |
| MRPL48 | -0.95787 | 7.488636 | -5.55969 | 5.01E-07 | 2.24E-06 | 5.808821 |
| C16orf61 | -1.04792 | 7.812142 | -5.55914 | 5.02E-07 | 2.24E-06 | 5.806747 |
| ZNF232 | 0.799501 | 7.599874 | 5.556117 | 5.08E-07 | 2.27E-06 | 5.795193 |
| TSPYL1 | -0.96222 | 8.378372 | -5.55601 | 5.08E-07 | 2.27E-06 | 5.794784 |
| COMMD1 | -0.9597 | 9.009438 | -5.55509 | 5.10E-07 | 2.28E-06 | 5.791283 |
| GORASP2 | -1.35323 | 8.132036 | -5.55358 | 5.13E-07 | 2.29E-06 | 5.785497 |
| PLEKHA9 | 0.915182 | 7.657895 | 5.553121 | 5.14E-07 | 2.29E-06 | 5.783754 |
| SOX1 | 1.070403 | 6.944443 | 5.552788 | 5.15E-07 | 2.29E-06 | 5.782486 |
| GPR27 | 1.452906 | 7.4864 | 5.552551 | 5.15E-07 | 2.30E-06 | 5.781581 |
| MIR933 | 1.534111 | 8.077222 | 5.55067 | 5.19E-07 | 2.31E-06 | 5.774402 |
| SAT2 | -0.95487 | 8.096926 | -5.54885 | 5.23E-07 | 2.33E-06 | 5.767462 |
| GPN2 | -1.3167 | 8.58116 | -5.54771 | 5.25E-07 | 2.34E-06 | 5.7631 |
| IL28A | 1.699403 | 7.650199 | 5.545996 | 5.29E-07 | 2.35E-06 | 5.756565 |
| FERMT2 | -0.93008 | 7.350435 | -5.5457 | 5.29E-07 | 2.35E-06 | 5.755449 |
| RIMBP3 | 1.172944 | 7.194704 | 5.544843 | 5.31E-07 | 2.36E-06 | 5.752168 |
| COL3A1 | -0.85545 | 7.343749 | -5.54378 | 5.33E-07 | 2.37E-06 | 5.748111 |
| NUP210L | 1.744109 | 7.296071 | 5.543644 | 5.34E-07 | 2.37E-06 | 5.747592 |
| PAQR6 | 1.452079 | 8.38965 | 5.541529 | 5.38E-07 | 2.39E-06 | 5.739526 |
| CNN3 | -1.52751 | 8.396859 | -5.54083 | 5.40E-07 | 2.39E-06 | 5.736843 |
| OR6K3 | 0.716542 | 6.871604 | 5.53847 | 5.45E-07 | 2.41E-06 | 5.727858 |
| SNORD35 | -1.87335 | 9.45518 | -5.53642 | 5.49E-07 | 2.43E-06 | 5.720036 |
| PSMC5 | -0.80039 | 7.31473 | -5.53481 | 5.53E-07 | 2.44E-06 | 5.713904 |
| C3orf32 | 0.736915 | 7.080339 | 5.534746 | 5.53E-07 | 2.44E-06 | 5.713659 |
| FBXO15 | 1.115581 | 7.380723 | 5.534559 | 5.53E-07 | 2.44E-06 | 5.712948 |
| RGAG1 | 1.156951 | 7.147929 | 5.53411 | 5.54E-07 | 2.45E-06 | 5.711236 |
| TUSC4 | -0.75761 | 7.420245 | -5.52895 | 5.65E-07 | 2.50E-06 | 5.691581 |
| EHHADH | -0.93557 | 7.893446 | -5.52798 | 5.68E-07 | 2.50E-06 | 5.687866 |
| MAS1 | 1.032079 | 7.0425 | 5.526314 | 5.71E-07 | 2.52E-06 | 5.681523 |
| FOSL1 | 0.793056 | 7.445268 | 5.524377 | 5.76E-07 | 2.54E-06 | 5.674143 |
| SRMS | 1.484438 | 7.4057 | 5.523672 | 5.77E-07 | 2.54E-06 | 5.671459 |
| LGI4 | 1.254331 | 7.543295 | 5.523619 | 5.77E-07 | 2.54E-06 | 5.671254 |
| VARS2 | -0.9924 | 10.54877 | -5.5229 | 5.79E-07 | 2.55E-06 | 5.668518 |
| P8 | -1.27595 | 8.448763 | -5.52228 | 5.80E-07 | 2.55E-06 | 5.666156 |
| NCRNA00 | -0.77152 | 7.078709 | -5.52055 | 5.84E-07 | 2.57E-06 | 5.659579 |
| SNORD1C | -1.44831 | 8.470356 | -5.51958 | 5.87E-07 | 2.58E-06 | 5.655862 |
| ORM1 | -1.79128 | 10.33344 | -5.51945 | 5.87E-07 | 2.58E-06 | 5.655362 |
| FTCD | -1.33856 | 10.0004 | -5.5189 | 5.88E-07 | 2.58E-06 | 5.653289 |
| SLC22A25 | -0.78965 | 7.57198 | -5.51871 | 5.89E-07 | 2.59E-06 | 5.652561 |
| SNORA28 | -0.80804 | 7.348775 | -5.51865 | 5.89E-07 | 2.59E-06 | 5.652335 |
| TNXB | 1.142564 | 7.953897 | 5.517979 | 5.90E-07 | 2.59E-06 | 5.649774 |
| ZNF616 | 0.846226 | 7.541401 | 5.517896 | 5.90E-07 | 2.59E-06 | 5.649456 |
| ZNF385D | 1.516416 | 8.056538 | 5.516735 | 5.93E-07 | 2.60E-06 | 5.645035 |
| MIR338 | 0.894147 | 7.019889 | 5.516624 | 5.93E-07 | 2.60E-06 | 5.644615 |
| C14orf183 | 0.81127 | 6.943334 | 5.516187 | 5.94E-07 | 2.61E-06 | 5.642952 |
| MRPL35 | -0.70624 | 7.68351 | -5.51313 | 6.02E-07 | 2.64E-06 | 5.631294 |
| MIR7-3 | 0.982238 | 6.981115 | 5.512504 | 6.03E-07 | 2.64E-06 | 5.628927 |
| SNORA74 | -1.4949 | 8.838737 | -5.51182 | 6.05E-07 | 2.65E-06 | 5.626343 |
| GUCA2B | 0.96123 | 7.310844 | 5.510686 | 6.07E-07 | 2.66E-06 | 5.622007 |
| TOP2B | -0.85272 | 8.513498 | -5.50847 | 6.13E-07 | 2.68E-06 | 5.613563 |
| OR5AY1 | 1.371989 | 7.418489 | 5.507551 | 6.15E-07 | 2.69E-06 | 5.610075 |
| MUL1 | -0.87497 | 7.526419 | -5.50661 | 6.17E-07 | 2.70E-06 | 5.606501 |
| CA9 | 1.851467 | 7.759643 | 5.506291 | 6.18E-07 | 2.70E-06 | 5.605283 |
| KRTAP10- | 0.783218 | 6.885891 | 5.503599 | 6.24E-07 | 2.73E-06 | 5.595038 |
| C19orf48 | -0.73847 | 7.839464 | -5.50239 | 6.27E-07 | 2.74E-06 | 5.590454 |
| RBMY1J | 1.005081 | 7.185044 | 5.500938 | 6.31E-07 | 2.75E-06 | 5.584918 |

| | | | | | | |
|---|---|---|---|---|---|---|
| RDH5 | -1.33662 | 8.473882 | -5.49988 | 6.34E-07 | 2.76E-06 | 5.580899 |
| CCM2 | -1.06197 | 7.8486 | -5.49878 | 6.36E-07 | 2.77E-06 | 5.576704 |
| INTS3 | -0.76444 | 7.577167 | -5.49826 | 6.38E-07 | 2.77E-06 | 5.57472 |
| RUFY4 | 1.281748 | 7.508952 | 5.493955 | 6.48E-07 | 2.82E-06 | 5.558361 |
| RBM22 | -1.30268 | 8.40703 | -5.49052 | 6.57E-07 | 2.86E-06 | 5.545314 |
| SPTA1 | 0.761585 | 7.095223 | 5.490136 | 6.58E-07 | 2.86E-06 | 5.543843 |
| SV2A | 0.985234 | 7.581307 | 5.487021 | 6.66E-07 | 2.89E-06 | 5.532003 |
| TMEM57 | -0.92825 | 8.056972 | -5.48618 | 6.68E-07 | 2.90E-06 | 5.528815 |
| WAS | -1.00579 | 8.509472 | -5.48562 | 6.70E-07 | 2.91E-06 | 5.526677 |
| SNORD76 | -1.03411 | 7.553473 | -5.48424 | 6.74E-07 | 2.92E-06 | 5.521434 |
| SNORA43 | -2.15421 | 10.4624 | -5.48304 | 6.77E-07 | 2.93E-06 | 5.516895 |
| CHN2 | -0.72622 | 7.679756 | -5.48281 | 6.77E-07 | 2.93E-06 | 5.51599 |
| LHX1 | 0.78117 | 6.923632 | 5.482486 | 6.78E-07 | 2.94E-06 | 5.514775 |
| OBFC2B | 0.711734 | 7.633961 | 5.482352 | 6.78E-07 | 2.94E-06 | 5.514266 |
| WISP2 | 1.043028 | 7.781012 | 5.48162 | 6.80E-07 | 2.95E-06 | 5.511483 |
| BEST3 | 0.895566 | 6.909862 | 5.481122 | 6.82E-07 | 2.95E-06 | 5.509591 |
| FLJ43752 | 0.804068 | 6.899404 | 5.478568 | 6.89E-07 | 2.98E-06 | 5.49989 |
| MLXIPL | -1.45443 | 9.569141 | -5.47382 | 7.01E-07 | 3.03E-06 | 5.48188 |
| HELT | 1.396603 | 7.384233 | 5.473188 | 7.03E-07 | 3.03E-06 | 5.47946 |
| FEM1B | -0.75392 | 7.992348 | -5.47307 | 7.04E-07 | 3.03E-06 | 5.479013 |
| FAF2 | -0.96098 | 7.702901 | -5.47306 | 7.04E-07 | 3.03E-06 | 5.478958 |
| BPIL2 | 0.788524 | 6.817138 | 5.47021 | 7.11E-07 | 3.06E-06 | 5.468159 |
| VIL2 | -1.45546 | 8.64962 | -5.46997 | 7.12E-07 | 3.07E-06 | 5.467233 |
| HSD17B6 | -1.25114 | 10.39534 | -5.46894 | 7.15E-07 | 3.08E-06 | 5.463324 |
| FER1L4 | 0.899246 | 7.753224 | 5.467229 | 7.20E-07 | 3.09E-06 | 5.456842 |
| RCE1 | 0.851884 | 9.180121 | 5.465975 | 7.23E-07 | 3.11E-06 | 5.452085 |
| SLC25A44 | -0.70247 | 7.407604 | -5.4656 | 7.24E-07 | 3.11E-06 | 5.450659 |
| CTSS | -0.70599 | 7.870939 | -5.46554 | 7.25E-07 | 3.11E-06 | 5.450452 |
| MAP3K2 | -1.06315 | 8.00902 | -5.46465 | 7.27E-07 | 3.12E-06 | 5.447057 |
| SLC25A30 | -1.00217 | 7.980316 | -5.46108 | 7.37E-07 | 3.16E-06 | 5.433511 |
| RNF152 | -0.75637 | 7.589634 | -5.46091 | 7.38E-07 | 3.16E-06 | 5.432857 |
| PPP2R1A | -0.72273 | 7.967534 | -5.46082 | 7.38E-07 | 3.16E-06 | 5.432544 |
| UQCRH | -1.2223 | 8.453978 | -5.46041 | 7.39E-07 | 3.17E-06 | 5.430979 |
| EPHX2 | -1.13382 | 7.84864 | -5.4601 | 7.40E-07 | 3.17E-06 | 5.429805 |
| SNORD37 | -1.012 | 7.720549 | -5.45902 | 7.43E-07 | 3.18E-06 | 5.425684 |
| ADAMTSL | -1.82621 | 9.429922 | -5.45892 | 7.43E-07 | 3.18E-06 | 5.425327 |
| TNKS1BP1 | -1.09623 | 8.354613 | -5.45798 | 7.46E-07 | 3.19E-06 | 5.421769 |
| GLG1 | -1.38656 | 9.170214 | -5.45698 | 7.49E-07 | 3.20E-06 | 5.417946 |
| HADH2 | -1.2389 | 9.133273 | -5.45678 | 7.50E-07 | 3.20E-06 | 5.417202 |
| POFUT2 | 0.756033 | 7.692813 | 5.456224 | 7.51E-07 | 3.21E-06 | 5.415094 |
| SLC7A14 | 0.742266 | 7.07793 | 5.45463 | 7.56E-07 | 3.23E-06 | 5.409051 |
| CAMKV | 0.703983 | 6.960811 | 5.454496 | 7.56E-07 | 3.23E-06 | 5.408541 |
| GPRIN2 | 0.870513 | 6.951727 | 5.453766 | 7.59E-07 | 3.24E-06 | 5.405776 |
| DAD1 | -0.77538 | 9.901665 | -5.4536 | 7.59E-07 | 3.24E-06 | 5.40513 |
| SLC11A2 | -1.42219 | 8.094387 | -5.45052 | 7.68E-07 | 3.27E-06 | 5.393478 |
| TSPAN3 | -0.72635 | 7.429933 | -5.45009 | 7.70E-07 | 3.28E-06 | 5.391845 |
| RBMY1A1 | 1.029603 | 7.208162 | 5.448525 | 7.74E-07 | 3.29E-06 | 5.385906 |
| LOC38891 | 0.943185 | 6.78015 | 5.44847 | 7.74E-07 | 3.29E-06 | 5.385699 |
| AK3 | -1.06279 | 8.194785 | -5.4477 | 7.77E-07 | 3.30E-06 | 5.38278 |
| MPG | 0.740931 | 6.938768 | 5.447398 | 7.78E-07 | 3.30E-06 | 5.381637 |
| BAIAP2L2 | -1.5379 | 8.293365 | -5.44702 | 7.79E-07 | 3.31E-06 | 5.380205 |
| SCARB1 | -0.94477 | 10.27008 | -5.44487 | 7.85E-07 | 3.34E-06 | 5.372069 |
| REXO2 | -1.03939 | 8.144585 | -5.44436 | 7.87E-07 | 3.34E-06 | 5.37014 |
| MSH4 | 1.087408 | 7.251599 | 5.443021 | 7.91E-07 | 3.36E-06 | 5.365052 |
| MECR | 0.848346 | 7.929564 | 5.442541 | 7.93E-07 | 3.36E-06 | 5.363232 |
| CDK5RAP2 | -0.7245 | 7.581558 | -5.44113 | 7.97E-07 | 3.38E-06 | 5.357893 |
| TSSK4 | 0.765919 | 7.009916 | 5.440209 | 8.00E-07 | 3.39E-06 | 5.3544 |
| FBXL21 | 1.007737 | 7.171753 | 5.439783 | 8.01E-07 | 3.39E-06 | 5.352786 |

| | | | | | | |
|---|---|---|---|---|---|---|
| UBC | -1.11208 | 9.360268 | -5.43973 | 8.01E-07 | 3.39E-06 | 5.352577 |
| APH1A | -0.98569 | 8.172406 | -5.43189 | 8.26E-07 | 3.49E-06 | 5.322883 |
| VDAC1 | -0.76081 | 7.782365 | -5.43129 | 8.28E-07 | 3.50E-06 | 5.320611 |
| FLJ16734 | 1.682594 | 8.223224 | 5.43012 | 8.32E-07 | 3.51E-06 | 5.316196 |
| SAFB | -0.76829 | 8.794342 | -5.43008 | 8.32E-07 | 3.51E-06 | 5.316043 |
| SNORA67 | -1.20676 | 8.249114 | -5.42933 | 8.34E-07 | 3.52E-06 | 5.313193 |
| KLHDC10 | -0.76238 | 7.521867 | -5.42868 | 8.37E-07 | 3.53E-06 | 5.310748 |
| RUVBL2 | -0.77521 | 7.708789 | -5.42739 | 8.41E-07 | 3.54E-06 | 5.305852 |
| ARF5 | -0.89532 | 8.056674 | -5.42709 | 8.42E-07 | 3.55E-06 | 5.304728 |
| TNFRSF18 | 1.510162 | 7.78301 | 5.426294 | 8.44E-07 | 3.55E-06 | 5.301717 |
| SNORA6 | -1.07636 | 8.548528 | -5.42627 | 8.44E-07 | 3.55E-06 | 5.301618 |
| ELOVL6 | -0.84622 | 7.637375 | -5.42393 | 8.52E-07 | 3.58E-06 | 5.292767 |
| NCRNA00 | 1.376924 | 7.614397 | 5.421828 | 8.59E-07 | 3.61E-06 | 5.284817 |
| UGT1A6 | -0.78535 | 8.096747 | -5.41839 | 8.71E-07 | 3.65E-06 | 5.271815 |
| IMPDH2 | -0.77428 | 7.762906 | -5.41838 | 8.71E-07 | 3.65E-06 | 5.271785 |
| BPIL1 | 0.725888 | 6.903023 | 5.417541 | 8.74E-07 | 3.67E-06 | 5.268605 |
| ITGA2B | 1.292656 | 7.455188 | 5.417384 | 8.74E-07 | 3.67E-06 | 5.268009 |
| AQP7 | 1.123162 | 10.04431 | 5.414694 | 8.83E-07 | 3.70E-06 | 5.257839 |
| KRT14 | 1.121394 | 7.179383 | 5.414203 | 8.85E-07 | 3.71E-06 | 5.255981 |
| LRRC52 | 1.031477 | 7.235815 | 5.410518 | 8.98E-07 | 3.76E-06 | 5.242052 |
| C19orf35 | 0.829793 | 6.8282 | 5.410139 | 8.99E-07 | 3.76E-06 | 5.240619 |
| CYP4A11 | -1.69593 | 7.99786 | -5.40856 | 9.05E-07 | 3.78E-06 | 5.234652 |
| VPS37D | -0.72872 | 7.656581 | -5.40763 | 9.08E-07 | 3.80E-06 | 5.231144 |
| C18orf10 | 1.004166 | 8.629181 | 5.406518 | 9.12E-07 | 3.81E-06 | 5.226934 |
| RP2 | -0.81424 | 7.259181 | -5.40573 | 9.15E-07 | 3.82E-06 | 5.223944 |
| COX8C | 0.807478 | 7.028193 | 5.404633 | 9.19E-07 | 3.84E-06 | 5.219811 |
| DYDC2 | 1.280573 | 7.910117 | 5.40426 | 9.20E-07 | 3.84E-06 | 5.218402 |
| HBG2 | 1.146894 | 7.430612 | 5.403843 | 9.21E-07 | 3.85E-06 | 5.216824 |
| VWA2 | 0.85713 | 6.877813 | 5.402336 | 9.27E-07 | 3.87E-06 | 5.211132 |
| PILRA | 0.919982 | 8.205348 | 5.402263 | 9.27E-07 | 3.87E-06 | 5.210857 |
| PLEKHG4 | 0.993283 | 7.281787 | 5.402096 | 9.28E-07 | 3.87E-06 | 5.210226 |
| PMM1 | -1.4348 | 9.181828 | -5.40065 | 9.33E-07 | 3.89E-06 | 5.20476 |
| OPTC | 1.249843 | 7.143564 | 5.400395 | 9.34E-07 | 3.89E-06 | 5.203802 |
| DPP4 | -0.73173 | 7.46941 | -5.39942 | 9.37E-07 | 3.90E-06 | 5.200101 |
| C6orf127 | 0.703133 | 6.841535 | 5.399248 | 9.38E-07 | 3.90E-06 | 5.199467 |
| C11orf59 | -1.32982 | 8.214712 | -5.39884 | 9.39E-07 | 3.91E-06 | 5.197937 |
| DHRS12 | 0.77983 | 7.612281 | 5.396618 | 9.48E-07 | 3.94E-06 | 5.189537 |
| CAMTA1 | 1.143248 | 7.379535 | 5.395034 | 9.53E-07 | 3.96E-06 | 5.183555 |
| SLC25A3 | -1.0756 | 8.968855 | -5.39439 | 9.56E-07 | 3.97E-06 | 5.181114 |
| SNORD60 | -1.7296 | 9.383869 | -5.39411 | 9.57E-07 | 3.97E-06 | 5.180057 |
| TRPC7 | 0.762162 | 6.890222 | 5.393234 | 9.60E-07 | 3.99E-06 | 5.17676 |
| UCHL1 | 0.970249 | 7.790907 | 5.39179 | 9.66E-07 | 4.01E-06 | 5.171306 |
| LOC72961 | 0.90572 | 8.718425 | 5.391739 | 9.66E-07 | 4.01E-06 | 5.171115 |
| FLJ32569 | 0.806896 | 7.06913 | 5.391099 | 9.68E-07 | 4.02E-06 | 5.1687 |
| TNS3 | -1.19937 | 8.898994 | -5.39009 | 9.72E-07 | 4.03E-06 | 5.164888 |
| CLDN1 | -1.31594 | 8.136294 | -5.38905 | 9.76E-07 | 4.05E-06 | 5.160968 |
| CPS1 | -1.35464 | 8.087846 | -5.387 | 9.84E-07 | 4.08E-06 | 5.153234 |
| C19orf39 | 1.076349 | 7.306004 | 5.386977 | 9.84E-07 | 4.08E-06 | 5.153144 |
| HBQ1 | 1.11331 | 7.137781 | 5.386123 | 9.87E-07 | 4.09E-06 | 5.14992 |
| COL18A1 | -1.03563 | 8.706734 | -5.38583 | 9.88E-07 | 4.09E-06 | 5.148817 |
| CYR61 | -1.2197 | 8.379503 | -5.38517 | 9.91E-07 | 4.10E-06 | 5.146338 |
| UBAC1 | -0.93571 | 8.745405 | -5.3846 | 9.93E-07 | 4.11E-06 | 5.144158 |
| CALM2 | -0.98135 | 7.687008 | -5.38061 | 1.01E-06 | 4.17E-06 | 5.129133 |
| TESSP5 | 0.790183 | 7.231048 | 5.377005 | 1.02E-06 | 4.22E-06 | 5.115528 |
| WDR7 | 0.85171 | 7.916321 | 5.375227 | 1.03E-06 | 4.25E-06 | 5.108822 |
| MIR219-2 | 0.764286 | 6.828275 | 5.37375 | 1.04E-06 | 4.27E-06 | 5.103254 |
| USP42 | 0.832554 | 8.572988 | 5.372911 | 1.04E-06 | 4.28E-06 | 5.100091 |
| C17orf102 | 0.766978 | 7.043954 | 5.369653 | 1.05E-06 | 4.33E-06 | 5.087812 |

| | | | | | | |
|---|---|---|---|---|---|---|
| DDR2 | 1.110341 | 8.194319 | 5.36815 | 1.06E-06 | 4.36E-06 | 5.082147 |
| DEFA1 | 1.298253 | 7.326312 | 5.367858 | 1.06E-06 | 4.36E-06 | 5.081049 |
| DGCR10 | 0.874609 | 7.379413 | 5.367715 | 1.06E-06 | 4.36E-06 | 5.08051 |
| FFAR2 | 1.270418 | 7.171812 | 5.364595 | 1.07E-06 | 4.41E-06 | 5.068754 |
| HSPA1B | -1.40432 | 8.790478 | -5.36434 | 1.07E-06 | 4.42E-06 | 5.06778 |
| SEC61G | -1.07385 | 8.237956 | -5.36406 | 1.08E-06 | 4.42E-06 | 5.066719 |
| WDR74 | -0.85454 | 8.472209 | -5.36379 | 1.08E-06 | 4.42E-06 | 5.065716 |
| VEGFB | -0.91342 | 8.051174 | -5.36164 | 1.09E-06 | 4.46E-06 | 5.057612 |
| C20orf196 | 0.838086 | 7.415183 | 5.361429 | 1.09E-06 | 4.46E-06 | 5.056828 |
| C2orf15 | 0.887101 | 7.301219 | 5.357442 | 1.10E-06 | 4.52E-06 | 5.041813 |
| OR10G3 | 1.143648 | 7.225182 | 5.356276 | 1.11E-06 | 4.54E-06 | 5.03742 |
| TRHDE | 0.978456 | 7.060642 | 5.356194 | 1.11E-06 | 4.54E-06 | 5.037114 |
| MT1E | -0.97878 | 8.6052 | -5.35596 | 1.11E-06 | 4.55E-06 | 5.036236 |
| CD300LF | -0.71055 | 7.496928 | -5.35486 | 1.11E-06 | 4.56E-06 | 5.032087 |
| DNAJC5G | 1.46958 | 7.644722 | 5.353365 | 1.12E-06 | 4.59E-06 | 5.026464 |
| GDF5 | 1.136808 | 7.169012 | 5.350062 | 1.14E-06 | 4.64E-06 | 5.01403 |
| TMEM59 | -1.1707 | 8.017392 | -5.34833 | 1.14E-06 | 4.67E-06 | 5.007508 |
| SILV | 1.186522 | 8.148491 | 5.348269 | 1.14E-06 | 4.67E-06 | 5.007286 |
| SH3BGRL2 | -0.85519 | 7.516236 | -5.34818 | 1.14E-06 | 4.67E-06 | 5.006961 |
| TMED10P | -0.78454 | 8.021135 | -5.34784 | 1.15E-06 | 4.68E-06 | 5.005676 |
| OR5V1 | 1.125274 | 7.158181 | 5.345498 | 1.16E-06 | 4.72E-06 | 4.996861 |
| PLXNA4B | 0.913487 | 7.127614 | 5.343994 | 1.16E-06 | 4.74E-06 | 4.991201 |
| SLC41A3 | 0.779537 | 7.774078 | 5.343436 | 1.16E-06 | 4.75E-06 | 4.989101 |
| NRSN1 | 0.826002 | 6.958317 | 5.343122 | 1.17E-06 | 4.75E-06 | 4.98792 |
| PITX2 | 0.943228 | 7.161569 | 5.342935 | 1.17E-06 | 4.76E-06 | 4.987219 |
| ATP5EP2 | 1.189104 | 7.990193 | 5.342511 | 1.17E-06 | 4.76E-06 | 4.985624 |
| TNFRSF21 | -0.72547 | 7.560021 | -5.34243 | 1.17E-06 | 4.76E-06 | 4.985315 |
| ANXA2P1 | -0.99071 | 7.465901 | -5.3415 | 1.17E-06 | 4.78E-06 | 4.981831 |
| PIWIL3 | 0.993847 | 6.939887 | 5.341121 | 1.18E-06 | 4.78E-06 | 4.980396 |
| ANKRD5 | 0.794545 | 7.893221 | 5.339702 | 1.18E-06 | 4.81E-06 | 4.97506 |
| PDCD6 | -0.84417 | 8.092121 | -5.33842 | 1.19E-06 | 4.83E-06 | 4.970228 |
| NEK3 | 1.244641 | 7.666495 | 5.338317 | 1.19E-06 | 4.83E-06 | 4.969852 |
| DCDC1 | 0.857396 | 6.960311 | 5.336835 | 1.19E-06 | 4.86E-06 | 4.964281 |
| AGBL2 | 0.767167 | 7.147047 | 5.333741 | 1.21E-06 | 4.91E-06 | 4.952648 |
| GALR3 | 1.499917 | 7.735939 | 5.333112 | 1.21E-06 | 4.92E-06 | 4.950282 |
| CHRD | -0.71161 | 7.637774 | -5.3324 | 1.22E-06 | 4.93E-06 | 4.947615 |
| CDRT15P | 0.822186 | 6.807136 | 5.331382 | 1.22E-06 | 4.95E-06 | 4.943782 |
| NOS1AP | 1.024978 | 8.357614 | 5.330479 | 1.22E-06 | 4.96E-06 | 4.940388 |
| HEYL | -0.9104 | 7.699266 | -5.32914 | 1.23E-06 | 4.99E-06 | 4.935351 |
| AKR1C4 | -1.39539 | 8.937724 | -5.32881 | 1.23E-06 | 4.99E-06 | 4.934114 |
| NDUFS8 | -0.70035 | 10.4851 | -5.3287 | 1.23E-06 | 4.99E-06 | 4.93372 |
| SNORD18 | -1.18433 | 7.87845 | -5.32843 | 1.23E-06 | 5.00E-06 | 4.932676 |
| CHRNA4 | 1.464854 | 7.82228 | 5.327913 | 1.24E-06 | 5.00E-06 | 4.930747 |
| UCN3 | 1.011304 | 7.124213 | 5.327468 | 1.24E-06 | 5.01E-06 | 4.929074 |
| SNORD87 | -0.84943 | 7.259951 | -5.32718 | 1.24E-06 | 5.01E-06 | 4.927994 |
| PAEP | 0.865161 | 7.136274 | 5.32439 | 1.25E-06 | 5.06E-06 | 4.91751 |
| DNAJB1 | -0.99288 | 7.815847 | -5.32167 | 1.27E-06 | 5.11E-06 | 4.907312 |
| VHLL | 1.022632 | 7.07176 | 5.320662 | 1.27E-06 | 5.13E-06 | 4.903512 |
| SORD | -0.94701 | 7.918158 | -5.31787 | 1.29E-06 | 5.18E-06 | 4.893035 |
| C1QL2 | 1.580458 | 7.610675 | 5.317694 | 1.29E-06 | 5.18E-06 | 4.892368 |
| LOC64384 | 1.231804 | 7.014895 | 5.317274 | 1.29E-06 | 5.19E-06 | 4.890789 |
| CD164L2 | 1.14969 | 7.210514 | 5.317131 | 1.29E-06 | 5.19E-06 | 4.890253 |
| BSG | -0.89976 | 9.608228 | -5.31259 | 1.31E-06 | 5.28E-06 | 4.873214 |
| HRG | -1.32219 | 9.77992 | -5.31121 | 1.32E-06 | 5.30E-06 | 4.868014 |
| CRIPAK | 0.743813 | 8.615561 | 5.310432 | 1.32E-06 | 5.32E-06 | 4.865112 |
| IGFL1 | 0.709482 | 6.875512 | 5.308269 | 1.33E-06 | 5.36E-06 | 4.856997 |
| FLJ20699 | -1.21667 | 8.510724 | -5.30152 | 1.37E-06 | 5.49E-06 | 4.831693 |
| LEPROTL1 | -0.77877 | 8.019188 | -5.29805 | 1.39E-06 | 5.56E-06 | 4.818677 |

| | | | | | | |
|---|---|---|---|---|---|---|
| ITGAL | -1.0547 | 8.357405 | -5.29699 | 1.39E-06 | 5.58E-06 | 4.81472 |
| INSIG1 | -1.16197 | 8.486214 | -5.29585 | 1.40E-06 | 5.60E-06 | 4.810424 |
| SERPINA1 | -0.71201 | 7.639351 | -5.2954 | 1.40E-06 | 5.61E-06 | 4.808736 |
| ABCA6 | -0.74544 | 7.773123 | -5.29508 | 1.40E-06 | 5.61E-06 | 4.807527 |
| RNY5 | -1.87617 | 11.039 | -5.29492 | 1.41E-06 | 5.61E-06 | 4.806934 |
| SMOX | 0.959559 | 7.626915 | 5.294764 | 1.41E-06 | 5.62E-06 | 4.80636 |
| DKFZp686 | 0.806272 | 7.459272 | 5.294696 | 1.41E-06 | 5.62E-06 | 4.806104 |
| PPAN | -0.74863 | 7.770619 | -5.29447 | 1.41E-06 | 5.62E-06 | 4.805249 |
| LILRA3 | 1.183919 | 7.283257 | 5.289943 | 1.43E-06 | 5.72E-06 | 4.788293 |
| C1orf210 | 0.873795 | 7.949298 | 5.28885 | 1.44E-06 | 5.74E-06 | 4.7842 |
| CCDC116 | 1.011298 | 7.136852 | 5.287996 | 1.44E-06 | 5.76E-06 | 4.781001 |
| GALNTL1 | 0.713583 | 7.28594 | 5.287915 | 1.44E-06 | 5.76E-06 | 4.780697 |
| C6orf156 | 0.961088 | 6.853724 | 5.287889 | 1.44E-06 | 5.76E-06 | 4.7806 |
| KRTAP4-1 | 0.822475 | 6.894634 | 5.287104 | 1.45E-06 | 5.77E-06 | 4.77766 |
| NTN5 | 0.784546 | 7.152515 | 5.286461 | 1.45E-06 | 5.78E-06 | 4.775252 |
| IGF2R | -1.12864 | 9.360953 | -5.28642 | 1.45E-06 | 5.78E-06 | 4.775103 |
| MCM6 | -0.8269 | 7.599495 | -5.28553 | 1.46E-06 | 5.80E-06 | 4.771755 |
| FTHL17 | 0.705461 | 6.818739 | 5.284561 | 1.46E-06 | 5.82E-06 | 4.768134 |
| HCST | -0.84805 | 8.292438 | -5.28448 | 1.46E-06 | 5.82E-06 | 4.767817 |
| UNC50 | -0.70313 | 8.528784 | -5.28405 | 1.47E-06 | 5.83E-06 | 4.766231 |
| FRMPD1 | 1.271207 | 7.407812 | 5.282975 | 1.47E-06 | 5.85E-06 | 4.762198 |
| GPR89A | 0.972464 | 8.921542 | 5.28283 | 1.47E-06 | 5.85E-06 | 4.761656 |
| CADM4 | 0.844925 | 7.863667 | 5.282207 | 1.48E-06 | 5.86E-06 | 4.759321 |
| PRR19 | 0.836763 | 7.282697 | 5.282155 | 1.48E-06 | 5.86E-06 | 4.759125 |
| OR4A15 | 0.998072 | 7.068363 | 5.281665 | 1.48E-06 | 5.87E-06 | 4.757293 |
| ZNF540 | 0.848656 | 7.536187 | 5.280926 | 1.48E-06 | 5.89E-06 | 4.754527 |
| INHBC | -0.7278 | 7.719451 | -5.28082 | 1.48E-06 | 5.89E-06 | 4.754126 |
| WDR12 | -0.77529 | 8.064468 | -5.28004 | 1.49E-06 | 5.90E-06 | 4.751197 |
| ANKRD1 | 1.570685 | 8.136425 | 5.272926 | 1.53E-06 | 6.06E-06 | 4.724585 |
| OR6Q1 | 1.247643 | 7.029444 | 5.270775 | 1.54E-06 | 6.11E-06 | 4.71654 |
| STAU1 | -0.88037 | 7.94481 | -5.27032 | 1.55E-06 | 6.12E-06 | 4.714819 |
| LOC37532 | 1.020254 | 7.019447 | 5.269096 | 1.55E-06 | 6.15E-06 | 4.710257 |
| CDH16 | 1.168664 | 7.286688 | 5.268344 | 1.56E-06 | 6.16E-06 | 4.707445 |
| TEX13A | 0.944946 | 7.030142 | 5.267265 | 1.56E-06 | 6.19E-06 | 4.70341 |
| AHRR | 0.769023 | 6.962776 | 5.266624 | 1.57E-06 | 6.20E-06 | 4.701013 |
| FAM19A4 | 1.205641 | 7.367349 | 5.265377 | 1.57E-06 | 6.23E-06 | 4.696349 |
| PPM1F | -1.13045 | 8.215799 | -5.26467 | 1.58E-06 | 6.24E-06 | 4.693713 |
| ACCS | -1.07824 | 8.648194 | -5.26298 | 1.59E-06 | 6.28E-06 | 4.687375 |
| APITD1 | 1.027379 | 8.603583 | 5.26206 | 1.60E-06 | 6.30E-06 | 4.683949 |
| TAGLN | -0.93205 | 7.891284 | -5.25972 | 1.61E-06 | 6.36E-06 | 4.675211 |
| GRM4 | 1.213465 | 7.332736 | 5.257456 | 1.62E-06 | 6.41E-06 | 4.666739 |
| C20orf11 | -0.83953 | 7.284306 | -5.25679 | 1.63E-06 | 6.42E-06 | 4.664234 |
| SNORA31 | -0.95372 | 7.498464 | -5.2554 | 1.64E-06 | 6.46E-06 | 4.659075 |
| GRN | -0.91838 | 7.825127 | -5.25536 | 1.64E-06 | 6.46E-06 | 4.658914 |
| MAT2A | -1.11554 | 8.484496 | -5.25299 | 1.65E-06 | 6.51E-06 | 4.650045 |
| FALZ | 1.293025 | 8.301968 | 5.251436 | 1.66E-06 | 6.55E-06 | 4.644249 |
| C13orf30 | 1.249289 | 7.184833 | 5.250233 | 1.67E-06 | 6.58E-06 | 4.639757 |
| LPIN1 | -0.82558 | 7.799723 | -5.24995 | 1.67E-06 | 6.58E-06 | 4.638691 |
| SHC4 | 0.73724 | 6.93267 | 5.249218 | 1.68E-06 | 6.60E-06 | 4.635967 |
| MS4A8B | 0.908841 | 7.007445 | 5.246619 | 1.69E-06 | 6.66E-06 | 4.626262 |
| SCP2 | -0.87844 | 8.566461 | -5.24611 | 1.70E-06 | 6.67E-06 | 4.624379 |
| SEC14L2 | -0.82957 | 7.415822 | -5.24181 | 1.72E-06 | 6.77E-06 | 4.608318 |
| PMPCA | -1.13807 | 7.877119 | -5.24174 | 1.72E-06 | 6.78E-06 | 4.608048 |
| TATDN3 | 1.098542 | 9.109837 | 5.241541 | 1.73E-06 | 6.78E-06 | 4.607306 |
| MIR1203 | 1.166192 | 7.943132 | 5.24141 | 1.73E-06 | 6.78E-06 | 4.606816 |
| REG1B | 1.028132 | 7.244091 | 5.238642 | 1.75E-06 | 6.85E-06 | 4.596487 |
| NSDHL | -1.4775 | 10.02423 | -5.23711 | 1.76E-06 | 6.88E-06 | 4.590771 |
| DECR2 | -0.96477 | 7.292106 | -5.23658 | 1.76E-06 | 6.90E-06 | 4.588786 |

| | | | | | | |
|---|---|---|---|---|---|---|
| AVP | 1.01762 | 7.243918 | 5.235862 | 1.76E-06 | 6.91E-06 | 4.586114 |
| KRT33A | 0.775697 | 6.798273 | 5.23471 | 1.77E-06 | 6.94E-06 | 4.581817 |
| LRCH3 | -0.91101 | 8.094163 | -5.23429 | 1.78E-06 | 6.95E-06 | 4.580261 |
| PSTK | 0.838359 | 8.329414 | 5.233203 | 1.78E-06 | 6.98E-06 | 4.576198 |
| BHLHE22 | 1.070488 | 7.786373 | 5.232312 | 1.79E-06 | 7.00E-06 | 4.572875 |
| CTAGE5 | 0.757079 | 7.643951 | 5.231896 | 1.79E-06 | 7.01E-06 | 4.571321 |
| SLC17A5 | 0.778566 | 7.69619 | 5.230048 | 1.80E-06 | 7.06E-06 | 4.564431 |
| CTBP1 | -0.7652 | 8.137409 | -5.22913 | 1.81E-06 | 7.08E-06 | 4.561005 |
| PDXK | -1.29836 | 8.558159 | -5.22859 | 1.81E-06 | 7.10E-06 | 4.559007 |
| FKSG30 | 0.733143 | 6.924494 | 5.228066 | 1.82E-06 | 7.11E-06 | 4.55704 |
| MIR146B | 0.998216 | 7.136944 | 5.226815 | 1.83E-06 | 7.14E-06 | 4.552378 |
| XPNPEP1 | -0.915 | 8.125977 | -5.22645 | 1.83E-06 | 7.15E-06 | 4.551023 |
| PTTG3P | 1.278677 | 8.047597 | 5.225154 | 1.84E-06 | 7.18E-06 | 4.546186 |
| PDE7B | 1.393609 | 7.591041 | 5.225118 | 1.84E-06 | 7.18E-06 | 4.54605 |
| SLC35A4 | -0.89269 | 8.69707 | -5.21876 | 1.88E-06 | 7.35E-06 | 4.522349 |
| H1FX | -1.0592 | 8.825333 | -5.21863 | 1.89E-06 | 7.35E-06 | 4.521885 |
| TBC1D29 | 1.17068 | 7.094962 | 5.218493 | 1.89E-06 | 7.35E-06 | 4.521363 |
| SRY | 1.226767 | 7.272821 | 5.217228 | 1.90E-06 | 7.39E-06 | 4.516653 |
| TTC39A | 0.854968 | 6.990394 | 5.216597 | 1.90E-06 | 7.40E-06 | 4.514302 |
| SGK3 | 0.735112 | 9.033228 | 5.216563 | 1.90E-06 | 7.40E-06 | 4.514174 |
| MGAT3 | 0.885643 | 7.039728 | 5.214491 | 1.92E-06 | 7.46E-06 | 4.506456 |
| PP8961 | 0.888444 | 11.86918 | 5.213365 | 1.92E-06 | 7.48E-06 | 4.502264 |
| DSC3 | 0.707439 | 7.035201 | 5.213307 | 1.92E-06 | 7.49E-06 | 4.50205 |
| AQP9 | -1.03902 | 7.602491 | -5.21294 | 1.93E-06 | 7.49E-06 | 4.500699 |
| TCEB3C | 1.494497 | 7.182873 | 5.210623 | 1.94E-06 | 7.56E-06 | 4.492053 |
| MID1IP1 | -0.70124 | 7.710607 | -5.21022 | 1.95E-06 | 7.57E-06 | 4.490553 |
| SEMA3A | 1.148791 | 7.666698 | 5.209988 | 1.95E-06 | 7.57E-06 | 4.489689 |
| FDFT1 | -1.17603 | 7.996238 | -5.20875 | 1.96E-06 | 7.60E-06 | 4.485074 |
| MYH7 | 0.756421 | 6.855046 | 5.208354 | 1.96E-06 | 7.61E-06 | 4.483607 |
| RPL9 | -1.32305 | 9.289718 | -5.20768 | 1.97E-06 | 7.63E-06 | 4.48109 |
| C20orf132 | 0.747969 | 7.083317 | 5.207126 | 1.97E-06 | 7.64E-06 | 4.479038 |
| FGGY | -0.70322 | 7.299636 | -5.20608 | 1.98E-06 | 7.67E-06 | 4.475136 |
| LEMD2 | -0.81247 | 8.0238 | -5.20543 | 1.98E-06 | 7.69E-06 | 4.472727 |
| RAB3IL1 | -1.35667 | 8.921712 | -5.204 | 1.99E-06 | 7.73E-06 | 4.467403 |
| SLC25A11 | -0.82427 | 7.582278 | -5.20371 | 2.00E-06 | 7.73E-06 | 4.466307 |
| BAG1 | 1.022037 | 7.70175 | 5.199447 | 2.03E-06 | 7.85E-06 | 4.450466 |
| RAX | 0.865721 | 6.99954 | 5.19698 | 2.05E-06 | 7.92E-06 | 4.441289 |
| C20orf160 | 0.731652 | 7.512357 | 5.196338 | 2.05E-06 | 7.94E-06 | 4.438903 |
| PBX1 | 1.449267 | 9.594377 | 5.194723 | 2.07E-06 | 7.98E-06 | 4.432895 |
| NPAS1 | -0.77585 | 7.455667 | -5.19402 | 2.07E-06 | 8.00E-06 | 4.430271 |
| HSPB7 | 1.358576 | 7.664493 | 5.192206 | 2.09E-06 | 8.05E-06 | 4.423537 |
| CHST12 | 1.151841 | 8.313394 | 5.191759 | 2.09E-06 | 8.06E-06 | 4.421876 |
| ZNF621 | 1.374747 | 9.062308 | 5.191143 | 2.09E-06 | 8.07E-06 | 4.419585 |
| ACAP3 | -0.71184 | 7.401672 | -5.19023 | 2.10E-06 | 8.10E-06 | 4.416192 |
| YIF1A | -1.16144 | 11.74167 | -5.19009 | 2.10E-06 | 8.10E-06 | 4.415674 |
| RPS27A | -1.27868 | 8.235769 | -5.18876 | 2.11E-06 | 8.14E-06 | 4.410718 |
| F9 | -1.38735 | 8.609641 | -5.18833 | 2.12E-06 | 8.15E-06 | 4.409128 |
| ATIC | -1.39585 | 8.525558 | -5.18677 | 2.13E-06 | 8.19E-06 | 4.403348 |
| BRUNOL5 | 0.963157 | 7.275402 | 5.185973 | 2.14E-06 | 8.21E-06 | 4.400373 |
| LSM1 | -1.04308 | 7.577102 | -5.18551 | 2.14E-06 | 8.23E-06 | 4.398654 |
| MYC | -0.79393 | 7.42698 | -5.1842 | 2.15E-06 | 8.26E-06 | 4.393789 |
| COL1A2 | -1.03715 | 8.269146 | -5.18381 | 2.15E-06 | 8.27E-06 | 4.392321 |
| HSPE1 | -0.70365 | 7.555255 | -5.18289 | 2.16E-06 | 8.30E-06 | 4.388937 |
| MSTP9 | 1.130925 | 7.756788 | 5.182661 | 2.16E-06 | 8.31E-06 | 4.38807 |
| TYMP | -1.03477 | 7.899432 | -5.1824 | 2.17E-06 | 8.31E-06 | 4.3871 |
| RPL27A | -0.99282 | 8.136032 | -5.182 | 2.17E-06 | 8.33E-06 | 4.385603 |
| BHMT2 | -1.063 | 8.256633 | -5.18089 | 2.18E-06 | 8.36E-06 | 4.381488 |
| LOC38915 | 1.24027 | 7.119097 | 5.180306 | 2.18E-06 | 8.38E-06 | 4.379323 |

| | | | | | | |
|---|---|---|---|---|---|---|
| AP1S2 | -1.21488 | 7.813093 | -5.18 | 2.19E-06 | 8.38E-06 | 4.378201 |
| ZC3H12D | 0.996202 | 7.960602 | 5.176951 | 2.21E-06 | 8.48E-06 | 4.366863 |
| FCAMR | 0.735452 | 7.593612 | 5.172411 | 2.25E-06 | 8.62E-06 | 4.350009 |
| HPX | -1.66462 | 9.93756 | -5.17229 | 2.25E-06 | 8.62E-06 | 4.349575 |
| TSLP | 1.229809 | 8.538901 | 5.170809 | 2.26E-06 | 8.66E-06 | 4.344063 |
| SIDT2 | -1.11036 | 8.23889 | -5.16917 | 2.28E-06 | 8.71E-06 | 4.337976 |
| ELFN2 | 1.24231 | 7.732573 | 5.168897 | 2.28E-06 | 8.72E-06 | 4.336971 |
| GBP5 | -0.73602 | 6.991837 | -5.1686 | 2.28E-06 | 8.72E-06 | 4.335869 |
| ABO | 1.46912 | 7.886362 | 5.167979 | 2.29E-06 | 8.74E-06 | 4.333563 |
| TCEAL4 | -0.84396 | 7.809332 | -5.16716 | 2.30E-06 | 8.77E-06 | 4.33052 |
| SNORA34 | -1.87878 | 10.32439 | -5.16404 | 2.32E-06 | 8.86E-06 | 4.318945 |
| UQCRFS1 | -0.78332 | 7.612509 | -5.16294 | 2.33E-06 | 8.90E-06 | 4.314886 |
| GRAMD4 | -1.14712 | 8.578478 | -5.16271 | 2.34E-06 | 8.90E-06 | 4.314004 |
| PPP1R15A | -1.40864 | 8.591737 | -5.16174 | 2.34E-06 | 8.93E-06 | 4.310415 |
| MYOM1 | -1.0533 | 8.532688 | -5.16138 | 2.35E-06 | 8.94E-06 | 4.309094 |
| ALX4 | 0.82708 | 6.838293 | 5.160238 | 2.36E-06 | 8.98E-06 | 4.304852 |
| SNORA68 | -1.09672 | 8.431621 | -5.15973 | 2.36E-06 | 8.99E-06 | 4.302967 |
| EMP1 | -0.79849 | 7.554884 | -5.15742 | 2.38E-06 | 9.06E-06 | 4.294414 |
| PTGES3 | -0.7502 | 7.445864 | -5.15714 | 2.39E-06 | 9.07E-06 | 4.293376 |
| LOC79112 | -1.34355 | 9.048363 | -5.15608 | 2.40E-06 | 9.11E-06 | 4.289433 |
| JAKMIP3 | 1.293971 | 7.496656 | 5.153045 | 2.42E-06 | 9.20E-06 | 4.278189 |
| UCHL3 | -0.8198 | 7.725824 | -5.14969 | 2.46E-06 | 9.31E-06 | 4.265749 |
| SLBP | -0.71684 | 7.781294 | -5.1484 | 2.47E-06 | 9.36E-06 | 4.260978 |
| HIST1H2B | -0.75619 | 7.689799 | -5.14648 | 2.49E-06 | 9.42E-06 | 4.253867 |
| LOC73009 | 1.308096 | 7.677841 | 5.146362 | 2.49E-06 | 9.42E-06 | 4.253432 |
| CENPF | 1.26211 | 7.773844 | 5.145356 | 2.50E-06 | 9.45E-06 | 4.249707 |
| LOC72893 | 0.944014 | 10.05556 | 5.141827 | 2.53E-06 | 9.57E-06 | 4.23664 |
| PRKDC | -0.9923 | 8.085491 | -5.14174 | 2.53E-06 | 9.57E-06 | 4.236326 |
| NMUR2 | 1.053665 | 7.127568 | 5.140452 | 2.54E-06 | 9.61E-06 | 4.231551 |
| IL6 | 1.122742 | 7.40767 | 5.139216 | 2.56E-06 | 9.65E-06 | 4.226976 |
| DCAF10 | -0.88372 | 7.500628 | -5.13911 | 2.56E-06 | 9.65E-06 | 4.22659 |
| IL17C | 1.0352 | 7.336393 | 5.13712 | 2.58E-06 | 9.72E-06 | 4.219219 |
| STARD8 | -0.85584 | 7.863251 | -5.13524 | 2.59E-06 | 9.78E-06 | 4.212249 |
| MAGEA2 | 0.722297 | 6.891023 | 5.133533 | 2.61E-06 | 9.84E-06 | 4.205946 |
| FBXO36 | 0.702812 | 7.494539 | 5.13269 | 2.62E-06 | 9.87E-06 | 4.202827 |
| SNORD3D | -1.63932 | 11.58237 | -5.13253 | 2.62E-06 | 9.88E-06 | 4.202246 |
| SLC39A9 | -0.77853 | 7.771146 | -5.13233 | 2.62E-06 | 9.88E-06 | 4.20149 |
| SAA2 | -1.66909 | 8.05254 | -5.13065 | 2.64E-06 | 9.94E-06 | 4.195269 |
| GAA | -0.76192 | 8.357889 | -5.12833 | 2.66E-06 | 1.00E-05 | 4.186704 |
| C7 | -0.94018 | 7.295842 | -5.12716 | 2.68E-06 | 1.01E-05 | 4.182361 |
| ACOT12 | -0.9697 | 8.189746 | -5.12665 | 2.68E-06 | 1.01E-05 | 4.180483 |
| C16orf7 | -0.73457 | 7.490856 | -5.12373 | 2.71E-06 | 1.02E-05 | 4.169712 |
| PPM1M | -1.08442 | 10.04541 | -5.1234 | 2.71E-06 | 1.02E-05 | 4.168459 |
| UGT1A4 | -1.00045 | 7.830309 | -5.1225 | 2.72E-06 | 1.02E-05 | 4.165146 |
| FOSB | -1.49794 | 8.153512 | -5.12247 | 2.72E-06 | 1.02E-05 | 4.165021 |
| IDUA | -0.93658 | 7.657115 | -5.12129 | 2.74E-06 | 1.03E-05 | 4.160687 |
| C22orf32 | 1.330339 | 8.559117 | 5.120745 | 2.74E-06 | 1.03E-05 | 4.158661 |
| SLN | 1.181681 | 7.190379 | 5.118746 | 2.76E-06 | 1.04E-05 | 4.151277 |
| HLF | -1.07992 | 8.513687 | -5.11752 | 2.78E-06 | 1.04E-05 | 4.146751 |
| FTL | -1.46352 | 10.80448 | -5.11588 | 2.79E-06 | 1.05E-05 | 4.140698 |
| KIAA1324 | 1.263502 | 7.462671 | 5.108465 | 2.87E-06 | 1.07E-05 | 4.113305 |
| RDH12 | 0.95332 | 7.421388 | 5.104255 | 2.92E-06 | 1.09E-05 | 4.097765 |
| HSPH1 | -0.72506 | 7.75419 | -5.10281 | 2.94E-06 | 1.09E-05 | 4.092417 |
| SPRR4 | 1.280183 | 7.528194 | 5.10067 | 2.96E-06 | 1.10E-05 | 4.084536 |
| CCL16 | -0.87499 | 7.505999 | -5.09979 | 2.97E-06 | 1.11E-05 | 4.081296 |
| TP53BP2 | 0.808475 | 11.16344 | 5.09929 | 2.98E-06 | 1.11E-05 | 4.079446 |
| RASGEF1C | 0.754545 | 6.940419 | 5.097792 | 2.99E-06 | 1.11E-05 | 4.07392 |
| C20orf24 | -0.78848 | 8.162559 | -5.09727 | 3.00E-06 | 1.11E-05 | 4.071992 |

| | | | | | | |
|---|---|---|---|---|---|---|
| GUSBL2 | 0.964009 | 7.4703 | 5.097178 | 3.00E-06 | 1.11E-05 | 4.071656 |
| DAZ2 | 0.909305 | 7.084768 | 5.095384 | 3.02E-06 | 1.12E-05 | 4.065041 |
| SNPH | 0.893926 | 6.94992 | 5.094874 | 3.03E-06 | 1.12E-05 | 4.06316 |
| SH3GL3 | 1.707303 | 7.876712 | 5.091745 | 3.06E-06 | 1.13E-05 | 4.051623 |
| TAS1R3 | 1.324751 | 7.390199 | 5.091036 | 3.07E-06 | 1.14E-05 | 4.049009 |
| TERC | -1.0092 | 9.034294 | -5.09073 | 3.07E-06 | 1.14E-05 | 4.047892 |
| DLST | -1.01909 | 8.406288 | -5.08576 | 3.13E-06 | 1.16E-05 | 4.029577 |
| SSTR5 | 1.165315 | 7.166961 | 5.080422 | 3.20E-06 | 1.18E-05 | 4.009903 |
| AVPI1 | -1.01899 | 9.165058 | -5.08016 | 3.20E-06 | 1.18E-05 | 4.008929 |
| AHR | -0.77522 | 7.645919 | -5.07995 | 3.20E-06 | 1.18E-05 | 4.008163 |
| PPP4C | -0.97897 | 7.961286 | -5.07764 | 3.23E-06 | 1.19E-05 | 3.999644 |
| FGF12 | 0.822921 | 7.261318 | 5.076306 | 3.25E-06 | 1.20E-05 | 3.994747 |
| ZBTB7B | -0.74209 | 7.741839 | -5.07395 | 3.28E-06 | 1.21E-05 | 3.986076 |
| FBXO16 | 0.844688 | 7.149803 | 5.073655 | 3.28E-06 | 1.21E-05 | 3.984987 |
| SNORA53 | -0.73478 | 7.225884 | -5.07242 | 3.30E-06 | 1.21E-05 | 3.980453 |
| TTBK1 | 1.34676 | 7.544045 | 5.070248 | 3.32E-06 | 1.22E-05 | 3.972449 |
| KATNB1 | -0.73294 | 8.013671 | -5.0699 | 3.33E-06 | 1.22E-05 | 3.971153 |
| ZCCHC16 | 1.125089 | 7.451821 | 5.06942 | 3.33E-06 | 1.23E-05 | 3.969404 |
| FOXD3 | 1.358808 | 7.183865 | 5.067543 | 3.36E-06 | 1.23E-05 | 3.962496 |
| WDFY1 | -0.94013 | 7.689084 | -5.06629 | 3.37E-06 | 1.24E-05 | 3.957897 |
| PPP1R7 | -0.84507 | 7.528781 | -5.06582 | 3.38E-06 | 1.24E-05 | 3.956146 |
| C7orf28A | 1.329025 | 8.586923 | 5.065024 | 3.39E-06 | 1.25E-05 | 3.953232 |
| FUT6 | 0.773267 | 7.408199 | 5.064355 | 3.40E-06 | 1.25E-05 | 3.950771 |
| RNU2-1 | -1.23225 | 9.79308 | -5.06365 | 3.41E-06 | 1.25E-05 | 3.948169 |
| VGF | 1.070191 | 7.148122 | 5.061253 | 3.44E-06 | 1.26E-05 | 3.939363 |
| LAIR1 | 0.88215 | 10.96426 | 5.061173 | 3.44E-06 | 1.26E-05 | 3.939071 |
| MFN2 | -0.73171 | 7.125327 | -5.05947 | 3.46E-06 | 1.27E-05 | 3.932816 |
| COX8A | -1.11664 | 7.961016 | -5.05522 | 3.52E-06 | 1.29E-05 | 3.917173 |
| FOXA3 | -0.94393 | 7.786956 | -5.05509 | 3.52E-06 | 1.29E-05 | 3.916708 |
| ADAM11 | 1.447654 | 7.60286 | 5.054392 | 3.53E-06 | 1.29E-05 | 3.914144 |
| CLEC4F | 1.650806 | 8.206297 | 5.054275 | 3.53E-06 | 1.29E-05 | 3.913715 |
| ANKRD45 | 0.756872 | 7.01699 | 5.053824 | 3.54E-06 | 1.29E-05 | 3.912057 |
| CDCA7 | 0.714046 | 7.313669 | 5.053422 | 3.54E-06 | 1.30E-05 | 3.910581 |
| CFHR5 | -0.96866 | 7.536046 | -5.05113 | 3.57E-06 | 1.31E-05 | 3.902167 |
| ECHS1 | -1.60005 | 9.519553 | -5.0511 | 3.57E-06 | 1.31E-05 | 3.902062 |
| MAB21L2 | 1.263841 | 8.060108 | 5.050799 | 3.58E-06 | 1.31E-05 | 3.900944 |
| SSX1 | 1.089608 | 7.574879 | 5.049463 | 3.60E-06 | 1.31E-05 | 3.896037 |
| OR56B4 | 1.460743 | 7.453316 | 5.047265 | 3.63E-06 | 1.32E-05 | 3.887964 |
| FLJ43763 | 1.051552 | 7.018537 | 5.0457 | 3.65E-06 | 1.33E-05 | 3.882221 |
| CNTF | 0.747974 | 7.137577 | 5.045328 | 3.65E-06 | 1.33E-05 | 3.880854 |
| MIR210 | 1.415065 | 8.121614 | 5.044237 | 3.67E-06 | 1.34E-05 | 3.876847 |
| PRDM16 | 0.761231 | 7.189923 | 5.043481 | 3.68E-06 | 1.34E-05 | 3.874073 |
| PLD6 | -0.75766 | 7.867759 | -5.04282 | 3.69E-06 | 1.34E-05 | 3.871646 |
| OR2V2 | 1.058806 | 7.063123 | 5.042413 | 3.69E-06 | 1.35E-05 | 3.870152 |
| SNORD46 | -0.98496 | 11.50575 | -5.0415 | 3.71E-06 | 1.35E-05 | 3.866806 |
| PCDHA13 | 1.298892 | 7.126544 | 5.038525 | 3.75E-06 | 1.36E-05 | 3.855884 |
| RBMS1 | 0.740187 | 7.967887 | 5.035544 | 3.79E-06 | 1.38E-05 | 3.844945 |
| PSME4 | -0.7919 | 7.859119 | -5.0341 | 3.81E-06 | 1.39E-05 | 3.839651 |
| LGALS7B | 0.997434 | 6.995742 | 5.033799 | 3.82E-06 | 1.39E-05 | 3.838545 |
| SH3GL1 | -1.22086 | 8.770398 | -5.03117 | 3.85E-06 | 1.40E-05 | 3.828917 |
| TRAPPC5 | -0.76569 | 8.246978 | -5.02967 | 3.88E-06 | 1.41E-05 | 3.823406 |
| GAGE12B | 1.628063 | 8.03278 | 5.026316 | 3.93E-06 | 1.43E-05 | 3.811108 |
| C13orf16 | 0.802256 | 7.080656 | 5.024157 | 3.96E-06 | 1.44E-05 | 3.803195 |
| KRBA1 | -1.10628 | 8.551265 | -5.02348 | 3.97E-06 | 1.44E-05 | 3.800721 |
| PANX3 | 1.106044 | 7.327795 | 5.021325 | 4.00E-06 | 1.45E-05 | 3.79282 |
| CCDC105 | 0.877076 | 6.913187 | 5.021007 | 4.01E-06 | 1.45E-05 | 3.791653 |
| CELSR3 | -0.72036 | 7.686393 | -5.01915 | 4.03E-06 | 1.46E-05 | 3.784844 |
| CHD2 | -0.73267 | 7.415711 | -5.01345 | 4.12E-06 | 1.49E-05 | 3.763992 |

| | | | | | | |
|---|---|---|---|---|---|---|
| MT2A | -1.14224 | 10.77495 | -5.01303 | 4.13E-06 | 1.49E-05 | 3.762441 |
| SNORD65 | -1.5054 | 9.104328 | -5.01205 | 4.14E-06 | 1.50E-05 | 3.758862 |
| MIR596 | 0.724986 | 6.759743 | 5.004003 | 4.27E-06 | 1.54E-05 | 3.729404 |
| VTRNA1-2 | -1.51108 | 9.478186 | -5.00209 | 4.30E-06 | 1.55E-05 | 3.722391 |
| SCARNA2( | -1.25255 | 9.258585 | -5.00186 | 4.31E-06 | 1.55E-05 | 3.721549 |
| UPK2 | 1.101719 | 7.291959 | 5.001604 | 4.31E-06 | 1.55E-05 | 3.720631 |
| LHX5 | 0.789398 | 7.041654 | 4.99874 | 4.36E-06 | 1.57E-05 | 3.710156 |
| MAT2B | -0.74193 | 8.384007 | -4.99822 | 4.37E-06 | 1.57E-05 | 3.708243 |
| LYL1 | -0.90671 | 7.967566 | -4.99785 | 4.37E-06 | 1.57E-05 | 3.706916 |
| C2orf62 | 1.240898 | 7.181448 | 4.996989 | 4.39E-06 | 1.58E-05 | 3.703756 |
| TREX1 | 0.798253 | 7.469186 | 4.995559 | 4.41E-06 | 1.59E-05 | 3.698529 |
| TYRO3 | 1.349531 | 8.140677 | 4.995484 | 4.41E-06 | 1.59E-05 | 3.698252 |
| STMN1 | 0.849449 | 7.199741 | 4.993468 | 4.45E-06 | 1.60E-05 | 3.690887 |
| SNORA62 | -0.79841 | 7.79128 | -4.99215 | 4.47E-06 | 1.60E-05 | 3.686082 |
| CACNG7 | 0.865398 | 6.939875 | 4.991252 | 4.48E-06 | 1.61E-05 | 3.682787 |
| BRSK1 | 1.540963 | 9.374812 | 4.990104 | 4.50E-06 | 1.62E-05 | 3.678592 |
| LY6G6D | 0.983405 | 6.911216 | 4.98927 | 4.52E-06 | 1.62E-05 | 3.675546 |
| ETNK2 | -0.74862 | 7.817229 | -4.98922 | 4.52E-06 | 1.62E-05 | 3.675357 |
| RNF20 | -0.75905 | 7.610883 | -4.98691 | 4.56E-06 | 1.63E-05 | 3.666909 |
| USE1 | -1.40538 | 8.154355 | -4.9859 | 4.57E-06 | 1.64E-05 | 3.663226 |
| LOC92017 | 0.875691 | 7.6411 | 4.984621 | 4.60E-06 | 1.65E-05 | 3.658567 |
| ITIH2 | -1.36266 | 9.059943 | -4.98143 | 4.65E-06 | 1.66E-05 | 3.646922 |
| KIAA1609 | 0.81784 | 7.230474 | 4.979239 | 4.69E-06 | 1.68E-05 | 3.638918 |
| EIF2B5 | -0.81234 | 7.365385 | -4.97842 | 4.71E-06 | 1.68E-05 | 3.635925 |
| PRIMA1 | 0.848159 | 7.019042 | 4.975684 | 4.75E-06 | 1.70E-05 | 3.625945 |
| RRP7B | 1.021966 | 11.37157 | 4.975208 | 4.76E-06 | 1.70E-05 | 3.624208 |
| PYY | 1.225866 | 7.3271 | 4.975004 | 4.77E-06 | 1.70E-05 | 3.623465 |
| DNASE1L3 | -1.05867 | 7.333099 | -4.97486 | 4.77E-06 | 1.70E-05 | 3.622956 |
| C1orf65 | 0.751295 | 6.876417 | 4.974485 | 4.78E-06 | 1.70E-05 | 3.62157 |
| UMOD | 0.779057 | 6.899175 | 4.974403 | 4.78E-06 | 1.70E-05 | 3.621272 |
| NDUFS2 | -1.00971 | 9.307715 | -4.97271 | 4.81E-06 | 1.71E-05 | 3.615091 |
| CYP8B1 | -1.81538 | 10.47254 | -4.96993 | 4.86E-06 | 1.73E-05 | 3.604963 |
| FLJ90650 | 0.80152 | 7.099207 | 4.96973 | 4.86E-06 | 1.73E-05 | 3.604227 |
| LOC92659 | 1.289924 | 8.152786 | 4.969285 | 4.87E-06 | 1.73E-05 | 3.602603 |
| GFOD1 | 1.138753 | 8.642251 | 4.968524 | 4.88E-06 | 1.74E-05 | 3.599828 |
| DEGS2 | 0.722377 | 7.006575 | 4.967154 | 4.91E-06 | 1.75E-05 | 3.594835 |
| SMYD1 | 0.74578 | 6.829021 | 4.965429 | 4.94E-06 | 1.76E-05 | 3.588546 |
| C14orf148 | 0.703211 | 7.013847 | 4.962741 | 4.99E-06 | 1.77E-05 | 3.578748 |
| LSMD1 | -1.14465 | 8.158667 | -4.9613 | 5.02E-06 | 1.78E-05 | 3.573508 |
| ATP1B1 | -0.75185 | 8.73997 | -4.96115 | 5.02E-06 | 1.78E-05 | 3.572959 |
| CNN1 | 1.576551 | 8.549651 | 4.957936 | 5.08E-06 | 1.80E-05 | 3.561243 |
| ATG2A | -0.83797 | 7.654158 | -4.95688 | 5.10E-06 | 1.81E-05 | 3.557386 |
| ITGAV | -0.9772 | 7.75811 | -4.954 | 5.16E-06 | 1.83E-05 | 3.546916 |
| PLA2G10 | 0.773041 | 7.150216 | 4.953391 | 5.17E-06 | 1.83E-05 | 3.54469 |
| HEBP2 | -0.87715 | 7.507242 | -4.95285 | 5.18E-06 | 1.84E-05 | 3.542734 |
| LAMC3 | -1.24456 | 8.106171 | -4.95106 | 5.22E-06 | 1.85E-05 | 3.536216 |
| CDC42EP4 | -0.87198 | 8.609833 | -4.94659 | 5.31E-06 | 1.87E-05 | 3.519921 |
| TMUB1 | -1.46924 | 8.915002 | -4.94511 | 5.33E-06 | 1.88E-05 | 3.514533 |
| KEL | 0.900496 | 7.369352 | 4.943719 | 5.36E-06 | 1.89E-05 | 3.509489 |
| UBAP2L | -0.75087 | 8.028077 | -4.94362 | 5.36E-06 | 1.89E-05 | 3.509113 |
| SNORD35 | -1.73154 | 9.169943 | -4.94275 | 5.38E-06 | 1.90E-05 | 3.505949 |
| GLTSCR2 | -1.15248 | 8.535506 | -4.94159 | 5.41E-06 | 1.91E-05 | 3.501757 |
| TEAD4 | 0.765721 | 8.324592 | 4.940898 | 5.42E-06 | 1.91E-05 | 3.499227 |
| DPYSL2 | -0.80252 | 7.510728 | -4.93975 | 5.44E-06 | 1.92E-05 | 3.495052 |
| SNORD94 | -1.48772 | 8.784617 | -4.93944 | 5.45E-06 | 1.92E-05 | 3.493932 |
| LOC73182 | 0.759386 | 6.914489 | 4.938969 | 5.46E-06 | 1.92E-05 | 3.49221 |
| WNT11 | 1.01442 | 7.742636 | 4.937573 | 5.49E-06 | 1.93E-05 | 3.487137 |
| KCNK4 | 1.079989 | 7.269098 | 4.937029 | 5.50E-06 | 1.93E-05 | 3.485159 |

| | | | | | | |
|---|---|---|---|---|---|---|
| ITLN1 | 0.810953 | 6.987887 | 4.932753 | 5.59E-06 | 1.96E-05 | 3.469617 |
| GNG13 | 0.836154 | 7.023261 | 4.932741 | 5.59E-06 | 1.96E-05 | 3.469573 |
| RCL1 | -0.79712 | 7.474843 | -4.93232 | 5.60E-06 | 1.97E-05 | 3.468031 |
| SPARC | -0.70022 | 7.277103 | -4.92962 | 5.65E-06 | 1.98E-05 | 3.458248 |
| TNFRSF1B | -0.92346 | 8.891737 | -4.92876 | 5.67E-06 | 1.99E-05 | 3.455106 |
| C20orf46 | -0.95512 | 7.66828 | -4.92657 | 5.72E-06 | 2.00E-05 | 3.44717 |
| IKZF4 | 0.880698 | 7.729686 | 4.922037 | 5.82E-06 | 2.03E-05 | 3.430692 |
| PAPOLA | -0.9321 | 7.510276 | -4.91993 | 5.86E-06 | 2.05E-05 | 3.423039 |
| HNF4A | -0.71378 | 7.822685 | -4.919 | 5.88E-06 | 2.05E-05 | 3.419673 |
| KRTAP10- | 1.534715 | 7.586711 | 4.917844 | 5.91E-06 | 2.06E-05 | 3.415473 |
| BRDT | 1.101644 | 7.112052 | 4.917014 | 5.93E-06 | 2.07E-05 | 3.412462 |
| HHIPL1 | 0.717203 | 6.970375 | 4.915814 | 5.96E-06 | 2.08E-05 | 3.408106 |
| CCDC123 | 0.907255 | 7.37927 | 4.911487 | 6.05E-06 | 2.11E-05 | 3.392409 |
| SYT1 | 0.801641 | 7.400552 | 4.906165 | 6.18E-06 | 2.15E-05 | 3.373113 |
| CABP7 | 0.709552 | 6.976366 | 4.905974 | 6.18E-06 | 2.15E-05 | 3.372421 |
| SUOX | -0.92221 | 8.810363 | -4.90571 | 6.19E-06 | 2.15E-05 | 3.371476 |
| ABCG4 | 0.734976 | 7.17061 | 4.905651 | 6.19E-06 | 2.15E-05 | 3.37125 |
| ASB12 | 0.786493 | 6.916262 | 4.905397 | 6.19E-06 | 2.16E-05 | 3.37033 |
| MADCAM | 0.914367 | 7.130041 | 4.904372 | 6.22E-06 | 2.16E-05 | 3.366615 |
| NUP133 | 0.943995 | 8.871067 | 4.900574 | 6.31E-06 | 2.19E-05 | 3.35285 |
| JTB | -1.15797 | 9.262453 | -4.89899 | 6.34E-06 | 2.20E-05 | 3.347117 |
| AASS | -0.96867 | 7.430977 | -4.89792 | 6.37E-06 | 2.21E-05 | 3.343254 |
| GABRA3 | 0.982808 | 7.106597 | 4.896696 | 6.40E-06 | 2.22E-05 | 3.338804 |
| ID3 | -0.86523 | 7.843966 | -4.8948 | 6.44E-06 | 2.23E-05 | 3.331922 |
| VEPH1 | 0.973875 | 7.265414 | 4.894225 | 6.46E-06 | 2.24E-05 | 3.329855 |
| C6 | -0.98456 | 9.16761 | -4.89359 | 6.47E-06 | 2.24E-05 | 3.327539 |
| TSPY2 | 1.739362 | 7.822406 | 4.893568 | 6.47E-06 | 2.24E-05 | 3.327475 |
| FAM98C | -1.05607 | 8.486073 | -4.89229 | 6.50E-06 | 2.25E-05 | 3.322857 |
| RARRES3 | -0.73503 | 7.685276 | -4.88874 | 6.59E-06 | 2.28E-05 | 3.310014 |
| CYP2C8 | -0.85866 | 7.547836 | -4.88832 | 6.60E-06 | 2.28E-05 | 3.308466 |
| CREG1 | -1.02753 | 9.019433 | -4.88821 | 6.61E-06 | 2.28E-05 | 3.308066 |
| SNORD45 | -0.79478 | 7.467357 | -4.88573 | 6.67E-06 | 2.30E-05 | 3.299091 |
| CORO2B | 0.704167 | 7.034071 | 4.884927 | 6.69E-06 | 2.31E-05 | 3.296202 |
| HSP90AB1 | -1.1804 | 8.011167 | -4.87743 | 6.88E-06 | 2.37E-05 | 3.269091 |
| RPS4Y1 | -0.83797 | 7.56142 | -4.87742 | 6.88E-06 | 2.37E-05 | 3.269047 |
| FOXRED1 | -1.06506 | 8.436111 | -4.87626 | 6.91E-06 | 2.38E-05 | 3.264865 |
| HSZFP36 | -1.08178 | 7.737457 | -4.87603 | 6.91E-06 | 2.38E-05 | 3.264047 |
| RNF44 | -0.7472 | 7.909851 | -4.8757 | 6.92E-06 | 2.38E-05 | 3.262845 |
| THBD | 0.713044 | 7.103781 | 4.873489 | 6.98E-06 | 2.40E-05 | 3.254848 |
| AKR1B10 | -0.98796 | 7.26802 | -4.87333 | 6.98E-06 | 2.40E-05 | 3.25427 |
| CENTA1 | -1.09339 | 8.217093 | -4.87045 | 7.06E-06 | 2.42E-05 | 3.243874 |
| TCTE1 | 1.03221 | 7.069528 | 4.863147 | 7.25E-06 | 2.48E-05 | 3.217493 |
| RGS9BP | 0.761059 | 6.872168 | 4.860893 | 7.32E-06 | 2.50E-05 | 3.209358 |
| C2orf51 | 1.147274 | 7.278074 | 4.8587 | 7.38E-06 | 2.52E-05 | 3.201445 |
| ZNF687 | -0.72745 | 7.55332 | -4.85755 | 7.41E-06 | 2.53E-05 | 3.19729 |
| CHCHD1 | -0.71335 | 7.209351 | -4.85728 | 7.42E-06 | 2.53E-05 | 3.196327 |
| DHRS7C | 1.237673 | 7.323734 | 4.856319 | 7.44E-06 | 2.54E-05 | 3.192853 |
| MFAP2 | 0.872308 | 7.511547 | 4.854797 | 7.48E-06 | 2.55E-05 | 3.187364 |
| BCAT2 | -0.70472 | 8.132851 | -4.85275 | 7.54E-06 | 2.57E-05 | 3.179982 |
| MSC | 1.509983 | 8.748601 | 4.852677 | 7.54E-06 | 2.57E-05 | 3.179718 |
| GABRP | 0.731238 | 7.184424 | 4.852396 | 7.55E-06 | 2.57E-05 | 3.178702 |
| TCEA3 | -0.7817 | 7.413284 | -4.85171 | 7.57E-06 | 2.58E-05 | 3.176242 |
| FMO5 | -0.75954 | 7.658678 | -4.84964 | 7.63E-06 | 2.60E-05 | 3.168752 |
| PIGP | -0.7212 | 8.054009 | -4.84917 | 7.64E-06 | 2.60E-05 | 3.167078 |
| CEND1 | 0.779215 | 7.062491 | 4.847791 | 7.68E-06 | 2.61E-05 | 3.162101 |
| C10orf65 | -0.93123 | 7.427065 | -4.84666 | 7.72E-06 | 2.62E-05 | 3.158022 |
| MPV17L2 | 1.035589 | 7.790911 | 4.845275 | 7.76E-06 | 2.64E-05 | 3.15303 |
| RPUSD2 | -0.86948 | 8.054664 | -4.84354 | 7.81E-06 | 2.65E-05 | 3.146791 |

| | | | | | | |
|---|---|---|---|---|---|---|
| ACTN1 | -1.1393 | 11.90504 | -4.84012 | 7.91E-06 | 2.69E-05 | 3.13446 |
| RBMX | -1.06338 | 8.186029 | -4.83904 | 7.94E-06 | 2.70E-05 | 3.130583 |
| CLCN5 | -0.70863 | 8.131443 | -4.83624 | 8.02E-06 | 2.72E-05 | 3.120472 |
| TRIM7 | 0.877347 | 7.234581 | 4.834689 | 8.07E-06 | 2.74E-05 | 3.114904 |
| FAH | -1.02346 | 8.11344 | -4.83463 | 8.07E-06 | 2.74E-05 | 3.114683 |
| PLEKHM1 | 0.736633 | 8.090251 | 4.833158 | 8.11E-06 | 2.75E-05 | 3.109391 |
| MRGPRD | 1.147626 | 7.229015 | 4.832347 | 8.14E-06 | 2.76E-05 | 3.106473 |
| LY96 | -1.22211 | 8.350933 | -4.832 | 8.15E-06 | 2.76E-05 | 3.105242 |
| GHITM | -1.16071 | 9.467921 | -4.8319 | 8.15E-06 | 2.76E-05 | 3.104852 |
| SNRPF | -0.93471 | 7.483244 | -4.82891 | 8.24E-06 | 2.79E-05 | 3.094092 |
| RBP1 | -0.93345 | 7.92326 | -4.82875 | 8.25E-06 | 2.79E-05 | 3.093533 |
| RNU1A3 | -1.3048 | 10.09127 | -4.82655 | 8.32E-06 | 2.81E-05 | 3.08562 |
| NAPSA | 0.754739 | 7.081023 | 4.82571 | 8.34E-06 | 2.82E-05 | 3.082596 |
| FAM176A | -0.73379 | 7.823439 | -4.82544 | 8.35E-06 | 2.82E-05 | 3.081624 |
| FAM71A | 1.087411 | 7.22197 | 4.824811 | 8.37E-06 | 2.83E-05 | 3.079361 |
| CAPN13 | 0.934233 | 7.072838 | 4.821715 | 8.47E-06 | 2.86E-05 | 3.068229 |
| EFNA2 | -1.01343 | 7.469492 | -4.82133 | 8.48E-06 | 2.86E-05 | 3.066852 |
| PPFIBP2 | -0.90133 | 7.893745 | -4.82103 | 8.49E-06 | 2.87E-05 | 3.065751 |
| MYT1 | 0.85655 | 7.018071 | 4.82038 | 8.51E-06 | 2.87E-05 | 3.063427 |
| P2RXL1 | 0.729699 | 6.886441 | 4.81946 | 8.54E-06 | 2.88E-05 | 3.06012 |
| ANGEL2 | -0.9298 | 8.185221 | -4.81637 | 8.64E-06 | 2.91E-05 | 3.049008 |
| UBXN2A | 0.737606 | 8.843232 | 4.816268 | 8.64E-06 | 2.91E-05 | 3.048651 |
| IBTK | -0.7264 | 7.288828 | -4.81515 | 8.68E-06 | 2.92E-05 | 3.044623 |
| PSMA6 | -1.17251 | 8.155125 | -4.81348 | 8.73E-06 | 2.94E-05 | 3.038643 |
| DLC1 | 0.952733 | 8.981416 | 4.813403 | 8.73E-06 | 2.94E-05 | 3.038357 |
| SNHG3 | 0.994071 | 11.51774 | 4.812678 | 8.76E-06 | 2.95E-05 | 3.035753 |
| FAM64A | 1.098312 | 7.285172 | 4.812223 | 8.77E-06 | 2.95E-05 | 3.034117 |
| LRP2BP | 1.234234 | 7.472516 | 4.811874 | 8.78E-06 | 2.95E-05 | 3.032865 |
| CYP2J2 | -1.11596 | 9.284796 | -4.80915 | 8.87E-06 | 2.98E-05 | 3.023072 |
| NUP93 | -1.03597 | 7.645469 | -4.80823 | 8.90E-06 | 2.99E-05 | 3.019779 |
| TMEM173 | -0.80023 | 8.218349 | -4.80748 | 8.93E-06 | 3.00E-05 | 3.017103 |
| ADM | -0.84095 | 7.793346 | -4.80424 | 9.04E-06 | 3.03E-05 | 3.005471 |
| SLC39A5 | -0.95994 | 7.693661 | -4.80093 | 9.15E-06 | 3.06E-05 | 2.993561 |
| FLJ10916 | -1.22241 | 8.498786 | -4.80049 | 9.16E-06 | 3.07E-05 | 2.992007 |
| JSRP1 | -1.0342 | 7.597046 | -4.80003 | 9.18E-06 | 3.07E-05 | 2.990362 |
| COL22A1 | 0.736176 | 7.098138 | 4.799103 | 9.21E-06 | 3.08E-05 | 2.987025 |
| SPRED1 | -0.9629 | 7.542364 | -4.79873 | 9.22E-06 | 3.09E-05 | 2.9857 |
| PIN1 | -1.1934 | 9.198509 | -4.79506 | 9.35E-06 | 3.13E-05 | 2.972512 |
| TCEB3 | -0.7506 | 7.674246 | -4.79191 | 9.46E-06 | 3.16E-05 | 2.961241 |
| PPIL6 | 0.984096 | 7.461206 | 4.790903 | 9.50E-06 | 3.17E-05 | 2.957621 |
| CDY2A | 0.800713 | 6.813795 | 4.785732 | 9.68E-06 | 3.23E-05 | 2.939092 |
| CWF19L1 | -0.79866 | 7.421778 | -4.7764 | 1.00E-05 | 3.33E-05 | 2.905696 |
| PARD6G | 0.957643 | 7.980354 | 4.776319 | 1.00E-05 | 3.33E-05 | 2.905389 |
| LOC38850 | -1.23496 | 8.840952 | -4.77126 | 1.02E-05 | 3.39E-05 | 2.887274 |
| B3GAT1 | 0.773285 | 7.219604 | 4.769512 | 1.03E-05 | 3.41E-05 | 2.881037 |
| HBB | -0.74211 | 7.29958 | -4.769 | 1.03E-05 | 3.42E-05 | 2.879197 |
| SCARNA1 | -1.17537 | 8.956117 | -4.76752 | 1.04E-05 | 3.44E-05 | 2.873898 |
| C7orf28B | -0.70975 | 7.415022 | -4.76303 | 1.05E-05 | 3.49E-05 | 2.857844 |
| REEP5 | -1.04824 | 9.652722 | -4.76187 | 1.06E-05 | 3.51E-05 | 2.853715 |
| COL9A2 | 0.94577 | 7.354778 | 4.755503 | 1.08E-05 | 3.59E-05 | 2.830972 |
| NARS | -0.80819 | 7.366023 | -4.75517 | 1.08E-05 | 3.59E-05 | 2.829771 |
| UBB | -0.86657 | 9.013173 | -4.75437 | 1.09E-05 | 3.60E-05 | 2.826918 |
| MFSD1 | -0.90819 | 8.338281 | -4.75113 | 1.10E-05 | 3.64E-05 | 2.815345 |
| LOC28317 | 0.702717 | 6.979654 | 4.74994 | 1.11E-05 | 3.65E-05 | 2.811111 |
| LOC28400 | 1.033221 | 7.527996 | 4.748375 | 1.11E-05 | 3.67E-05 | 2.805526 |
| ARL10 | 0.747581 | 7.391135 | 4.745888 | 1.12E-05 | 3.70E-05 | 2.796652 |
| LECT2 | -1.18254 | 8.036922 | -4.74516 | 1.13E-05 | 3.71E-05 | 2.794046 |
| EPO | 0.884958 | 6.976725 | 4.740857 | 1.14E-05 | 3.76E-05 | 2.778706 |

| | | | | | | |
|---|---|---|---|---|---|---|
| EDF1 | -0.80044 | 9.154389 | -4.74052 | 1.14E-05 | 3.77E-05 | 2.777492 |
| LOC28501 | 1.010443 | 7.535889 | 4.739888 | 1.15E-05 | 3.78E-05 | 2.775253 |
| CASS4 | 0.93887 | 7.777124 | 4.739318 | 1.15E-05 | 3.78E-05 | 2.77322 |
| CCDC58 | -0.83931 | 8.002449 | -4.73821 | 1.15E-05 | 3.80E-05 | 2.769265 |
| JPH1 | 1.007846 | 7.671612 | 4.735831 | 1.16E-05 | 3.82E-05 | 2.760791 |
| DCTN6 | -0.90651 | 7.903756 | -4.73266 | 1.18E-05 | 3.87E-05 | 2.749503 |
| KIAA0430 | -0.92605 | 8.058068 | -4.73207 | 1.18E-05 | 3.87E-05 | 2.747403 |
| TADA2B | 0.896657 | 8.987865 | 4.730495 | 1.19E-05 | 3.89E-05 | 2.741778 |
| NPAS4 | 0.821949 | 6.968014 | 4.724259 | 1.22E-05 | 3.98E-05 | 2.719573 |
| TXNDC5 | -0.92121 | 8.958252 | -4.72205 | 1.23E-05 | 4.01E-05 | 2.711711 |
| GMFB | -0.8385 | 7.509766 | -4.72202 | 1.23E-05 | 4.01E-05 | 2.711622 |
| TPRX1 | 1.029524 | 6.960487 | 4.719619 | 1.24E-05 | 4.04E-05 | 2.703062 |
| PPP2R2D | -0.74334 | 8.357618 | -4.71854 | 1.24E-05 | 4.05E-05 | 2.699224 |
| GPRIN3 | -0.71398 | 7.311171 | -4.71822 | 1.24E-05 | 4.06E-05 | 2.698099 |
| COL4A3 | 1.207382 | 7.595609 | 4.713836 | 1.26E-05 | 4.12E-05 | 2.682494 |
| UPK1A | 1.078163 | 7.7862 | 4.713392 | 1.27E-05 | 4.12E-05 | 2.680913 |
| HGS | -1.02039 | 11.7203 | -4.71302 | 1.27E-05 | 4.13E-05 | 2.679593 |
| ZNF385A | 0.967648 | 9.189352 | 4.712887 | 1.27E-05 | 4.13E-05 | 2.679118 |
| LUZP2 | 0.750427 | 7.110201 | 4.711464 | 1.27E-05 | 4.15E-05 | 2.67406 |
| NUDT5 | -0.70036 | 7.333521 | -4.70788 | 1.29E-05 | 4.20E-05 | 2.66132 |
| DRP2 | 0.964009 | 7.049197 | 4.707509 | 1.29E-05 | 4.21E-05 | 2.660002 |
| TMEM140 | -0.77095 | 8.449946 | -4.70657 | 1.30E-05 | 4.22E-05 | 2.656668 |
| HSPD1 | -0.71767 | 8.569054 | -4.7056 | 1.30E-05 | 4.23E-05 | 2.653212 |
| ACAN | 1.216 | 7.141379 | 4.703982 | 1.31E-05 | 4.25E-05 | 2.647472 |
| ZNF28 | 0.763729 | 8.251089 | 4.702021 | 1.32E-05 | 4.28E-05 | 2.640507 |
| PSAT1 | -0.81684 | 7.473024 | -4.69894 | 1.33E-05 | 4.33E-05 | 2.62957 |
| CDKN2D | 0.791428 | 7.68752 | 4.698655 | 1.34E-05 | 4.33E-05 | 2.628556 |
| PPP1R9B | -0.72478 | 7.494184 | -4.69829 | 1.34E-05 | 4.33E-05 | 2.627257 |
| PFKFB3 | -0.90614 | 7.70333 | -4.69705 | 1.34E-05 | 4.35E-05 | 2.62287 |
| POLD4 | -0.93474 | 8.068145 | -4.69617 | 1.35E-05 | 4.36E-05 | 2.619747 |
| CDK2AP2 | -0.82465 | 10.15569 | -4.69564 | 1.35E-05 | 4.37E-05 | 2.617841 |
| FAM44B | -1.18958 | 8.580114 | -4.69492 | 1.35E-05 | 4.38E-05 | 2.615285 |
| SNX20 | 0.878871 | 7.495037 | 4.694596 | 1.36E-05 | 4.38E-05 | 2.614153 |
| LHPP | -1.11963 | 8.685456 | -4.69342 | 1.36E-05 | 4.40E-05 | 2.609976 |
| UPLP | -0.93651 | 8.226534 | -4.69337 | 1.36E-05 | 4.40E-05 | 2.609784 |
| PIM3 | -0.75608 | 7.588547 | -4.69314 | 1.36E-05 | 4.40E-05 | 2.608987 |
| EPHX1 | -1.08622 | 8.198439 | -4.69153 | 1.37E-05 | 4.43E-05 | 2.603275 |
| C3orf26 | -0.8098 | 8.063884 | -4.68915 | 1.38E-05 | 4.46E-05 | 2.594838 |
| MYH16 | 1.342587 | 7.339488 | 4.688099 | 1.39E-05 | 4.48E-05 | 2.591106 |
| HIGD1A | -0.76483 | 7.765935 | -4.68636 | 1.40E-05 | 4.50E-05 | 2.584929 |
| CYP27A1 | -0.72616 | 7.596127 | -4.68537 | 1.40E-05 | 4.52E-05 | 2.581429 |
| ONECUT1 | -0.767 | 8.508032 | -4.68452 | 1.41E-05 | 4.53E-05 | 2.578432 |
| LOC14772 | 0.961986 | 8.617361 | 4.684176 | 1.41E-05 | 4.54E-05 | 2.577196 |
| ICOSLG | 0.894614 | 9.628658 | 4.683261 | 1.41E-05 | 4.55E-05 | 2.573955 |
| KRI1 | 0.922618 | 8.919123 | 4.682104 | 1.42E-05 | 4.57E-05 | 2.569854 |
| SLC16A9 | 1.67961 | 8.31793 | 4.681802 | 1.42E-05 | 4.57E-05 | 2.568784 |
| PEMT | -0.76293 | 8.88857 | -4.67996 | 1.43E-05 | 4.60E-05 | 2.562263 |
| ELP4 | 1.271241 | 8.663845 | 4.679675 | 1.43E-05 | 4.60E-05 | 2.561246 |
| RBP4 | -1.43846 | 9.570494 | -4.67872 | 1.44E-05 | 4.62E-05 | 2.557855 |
| SLPI | -1.18901 | 7.745956 | -4.67516 | 1.46E-05 | 4.67E-05 | 2.545259 |
| NDUFB6 | -0.85196 | 7.859911 | -4.67413 | 1.46E-05 | 4.69E-05 | 2.541605 |
| PROL1 | 0.829068 | 7.231439 | 4.673768 | 1.46E-05 | 4.69E-05 | 2.540326 |
| ADRB3 | 0.810439 | 7.126869 | 4.673126 | 1.47E-05 | 4.71E-05 | 2.538053 |
| LCE1E | 0.705684 | 6.900047 | 4.6646 | 1.51E-05 | 4.84E-05 | 2.507886 |
| DNAH1 | -1.43276 | 9.725134 | -4.66362 | 1.52E-05 | 4.86E-05 | 2.504431 |
| LOC65036 | 1.269607 | 7.677028 | 4.662877 | 1.52E-05 | 4.87E-05 | 2.50179 |
| ATP6V1G1 | -0.72386 | 7.315311 | -4.66244 | 1.53E-05 | 4.88E-05 | 2.500238 |
| NXF3 | 0.772868 | 7.242676 | 4.661779 | 1.53E-05 | 4.89E-05 | 2.497909 |

| | | | | | | |
|---|---|---|---|---|---|---|
| UGT1A3 | -0.83482 | 7.960751 | -4.66144 | 1.53E-05 | 4.89E-05 | 2.496725 |
| HCG27 | 1.302031 | 8.335615 | 4.660585 | 1.54E-05 | 4.91E-05 | 2.493685 |
| PLSCR3 | -0.71352 | 7.938128 | -4.66033 | 1.54E-05 | 4.91E-05 | 2.492774 |
| CCDC56 | -1.28816 | 9.836614 | -4.65679 | 1.56E-05 | 4.97E-05 | 2.480268 |
| PRDM4 | -0.7311 | 8.561251 | -4.65264 | 1.58E-05 | 5.04E-05 | 2.465593 |
| LASP1 | -0.79142 | 9.170103 | -4.65121 | 1.59E-05 | 5.06E-05 | 2.460546 |
| HDAC6 | -0.70014 | 7.365111 | -4.65103 | 1.59E-05 | 5.07E-05 | 2.459911 |
| NLRC4 | 0.968288 | 7.410135 | 4.648067 | 1.61E-05 | 5.12E-05 | 2.449462 |
| CYP1A2 | -1.51607 | 8.46498 | -4.6472 | 1.61E-05 | 5.13E-05 | 2.446401 |
| GTF2H2B | -0.71261 | 7.685982 | -4.64524 | 1.63E-05 | 5.16E-05 | 2.439498 |
| MRPS30 | -0.75586 | 8.169814 | -4.64315 | 1.64E-05 | 5.20E-05 | 2.432101 |
| RGS12 | -0.82431 | 8.478691 | -4.64158 | 1.65E-05 | 5.22E-05 | 2.426553 |
| WDR5B | 0.944424 | 7.437381 | 4.641026 | 1.65E-05 | 5.23E-05 | 2.424609 |
| F10 | -1.28201 | 9.305316 | -4.63839 | 1.67E-05 | 5.28E-05 | 2.415326 |
| NEUROG3 | 0.966726 | 7.100311 | 4.635617 | 1.68E-05 | 5.33E-05 | 2.405533 |
| SH2D4A | -0.73486 | 7.870474 | -4.63518 | 1.69E-05 | 5.34E-05 | 2.404011 |
| C1orf117 | 0.753567 | 6.883033 | 4.633867 | 1.69E-05 | 5.36E-05 | 2.399364 |
| CLPTM1 | -0.93927 | 10.07683 | -4.63355 | 1.70E-05 | 5.37E-05 | 2.398252 |
| TTC22 | 0.829621 | 7.662196 | 4.632941 | 1.70E-05 | 5.38E-05 | 2.396099 |
| BCL6 | -0.83819 | 7.895303 | -4.63285 | 1.70E-05 | 5.38E-05 | 2.395797 |
| PGLYRP2 | -1.29017 | 9.166582 | -4.62516 | 1.75E-05 | 5.52E-05 | 2.368674 |
| C9orf71 | 0.797299 | 7.013379 | 4.624795 | 1.75E-05 | 5.53E-05 | 2.3674 |
| KIF7 | 0.94509 | 7.902999 | 4.616424 | 1.81E-05 | 5.69E-05 | 2.337935 |
| PPID | 0.96084 | 11.68709 | 4.616248 | 1.81E-05 | 5.69E-05 | 2.337316 |
| BBS4 | -1.09014 | 8.782968 | -4.61498 | 1.82E-05 | 5.71E-05 | 2.332866 |
| RBPMS2 | -1.04144 | 9.327164 | -4.61321 | 1.83E-05 | 5.74E-05 | 2.326637 |
| SLC16A8 | 0.855582 | 7.050277 | 4.609803 | 1.85E-05 | 5.81E-05 | 2.314648 |
| MGC3340 | 0.719225 | 6.822006 | 4.607165 | 1.87E-05 | 5.86E-05 | 2.305376 |
| LOC7286C | 1.290891 | 7.423245 | 4.605083 | 1.88E-05 | 5.90E-05 | 2.298061 |
| UBXN1 | -1.16663 | 8.53718 | -4.60462 | 1.89E-05 | 5.91E-05 | 2.296423 |
| PAK2 | -0.84368 | 8.310526 | -4.60214 | 1.90E-05 | 5.96E-05 | 2.287725 |
| KIAA0513 | 0.796607 | 7.714231 | 4.601897 | 1.90E-05 | 5.96E-05 | 2.286868 |
| STK3 | -0.83503 | 7.548583 | -4.60106 | 1.91E-05 | 5.98E-05 | 2.283924 |
| RHOT2 | -0.83374 | 7.901691 | -4.59832 | 1.93E-05 | 6.03E-05 | 2.274306 |
| ATF2 | -0.9401 | 8.285614 | -4.59738 | 1.94E-05 | 6.05E-05 | 2.27102 |
| SH3BP4 | -0.70065 | 8.145617 | -4.59641 | 1.94E-05 | 6.07E-05 | 2.267598 |
| LOC92249 | -1.23124 | 8.440083 | -4.59267 | 1.97E-05 | 6.15E-05 | 2.254461 |
| UBE2A | -0.71808 | 7.636237 | -4.58923 | 1.99E-05 | 6.23E-05 | 2.2424 |
| SCD | -1.36238 | 9.127669 | -4.58914 | 2.00E-05 | 6.23E-05 | 2.242097 |
| C10orf76 | 0.847124 | 7.827328 | 4.5877 | 2.01E-05 | 6.25E-05 | 2.237038 |
| PIGT | -0.98314 | 8.543703 | -4.58426 | 2.03E-05 | 6.33E-05 | 2.224967 |
| GCGR | -1.55991 | 8.25957 | -4.58355 | 2.04E-05 | 6.34E-05 | 2.222498 |
| SLC6A11 | 1.727405 | 9.389381 | 4.58052 | 2.06E-05 | 6.41E-05 | 2.21187 |
| GPI | -0.7988 | 9.504183 | -4.57638 | 2.09E-05 | 6.50E-05 | 2.197379 |
| PKN3 | 0.77853 | 8.80537 | 4.575966 | 2.09E-05 | 6.50E-05 | 2.195916 |
| LOC73177 | 1.340105 | 7.252611 | 4.573026 | 2.12E-05 | 6.57E-05 | 2.185621 |
| SNRNP40 | 0.867338 | 8.723063 | 4.570039 | 2.14E-05 | 6.64E-05 | 2.175163 |
| RNU11 | -1.14202 | 9.116546 | -4.56917 | 2.15E-05 | 6.65E-05 | 2.172126 |
| DDIT4L | 1.156212 | 7.426 | 4.56764 | 2.16E-05 | 6.69E-05 | 2.16677 |
| HSPB2 | 0.763373 | 7.367888 | 4.567405 | 2.16E-05 | 6.69E-05 | 2.165945 |
| EGLN1 | -0.8004 | 7.938502 | -4.56658 | 2.17E-05 | 6.71E-05 | 2.163071 |
| BIRC2 | -0.96359 | 7.655117 | -4.56571 | 2.17E-05 | 6.72E-05 | 2.160015 |
| C21orf33 | -0.75598 | 8.078645 | -4.5641 | 2.19E-05 | 6.76E-05 | 2.154393 |
| RNU6-1 | -0.70841 | 12.44278 | -4.5603 | 2.22E-05 | 6.85E-05 | 2.141086 |
| LSM14A | -1.11795 | 9.677078 | -4.55988 | 2.22E-05 | 6.85E-05 | 2.139636 |
| FBXO10 | 0.710558 | 7.499777 | 4.559723 | 2.22E-05 | 6.86E-05 | 2.13908 |
| KCNN3 | 0.757895 | 7.61691 | 4.558748 | 2.23E-05 | 6.88E-05 | 2.13567 |
| C12orf47 | -0.71624 | 7.550791 | -4.55829 | 2.23E-05 | 6.89E-05 | 2.134087 |

| | | | | | | |
|---|---|---|---|---|---|---|
| TSGA10 | 0.74138 | 7.061899 | 4.551535 | 2.29E-05 | 7.04E-05 | 2.110468 |
| RASL12 | 1.371489 | 8.493896 | 4.551452 | 2.29E-05 | 7.04E-05 | 2.110179 |
| C1QC | -0.95335 | 9.779774 | -4.55113 | 2.29E-05 | 7.05E-05 | 2.109066 |
| UHMK1 | -0.84985 | 7.520884 | -4.55111 | 2.29E-05 | 7.05E-05 | 2.108997 |
| FITM2 | 0.920902 | 8.89044 | 4.54999 | 2.30E-05 | 7.07E-05 | 2.105075 |
| CCBL1 | -0.75655 | 7.537013 | -4.54884 | 2.31E-05 | 7.10E-05 | 2.101074 |
| IGFBP4 | -0.86893 | 11.29191 | -4.54741 | 2.32E-05 | 7.14E-05 | 2.09606 |
| ZNF764 | 0.812221 | 8.075994 | 4.542762 | 2.36E-05 | 7.25E-05 | 2.079844 |
| HRC | 0.714834 | 7.091568 | 4.542315 | 2.37E-05 | 7.26E-05 | 2.078286 |
| HMGB1 | -0.7202 | 7.585994 | -4.54166 | 2.37E-05 | 7.27E-05 | 2.076017 |
| S100A11 | -0.96723 | 9.797586 | -4.53691 | 2.41E-05 | 7.39E-05 | 2.059426 |
| TTYH3 | -0.74102 | 8.26711 | -4.53034 | 2.47E-05 | 7.55E-05 | 2.036546 |
| MYO9B | -0.70205 | 8.173241 | -4.52933 | 2.48E-05 | 7.57E-05 | 2.033026 |
| GSN | -0.72624 | 7.783796 | -4.52769 | 2.49E-05 | 7.62E-05 | 2.027318 |
| CSAG3A | 1.254677 | 7.847449 | 4.525721 | 2.51E-05 | 7.66E-05 | 2.020446 |
| SCGB3A1 | 0.745495 | 6.93242 | 4.523569 | 2.53E-05 | 7.72E-05 | 2.012954 |
| TMEM201 | 0.913736 | 8.02873 | 4.522449 | 2.54E-05 | 7.75E-05 | 2.009056 |
| C9orf140 | 0.925967 | 7.429171 | 4.519842 | 2.57E-05 | 7.81E-05 | 1.999983 |
| NDUFV1 | -1.06911 | 8.144288 | -4.5197 | 2.57E-05 | 7.81E-05 | 1.999478 |
| GHR | -1.0584 | 7.674503 | -4.51925 | 2.57E-05 | 7.82E-05 | 1.997932 |
| RNF19A | -0.95618 | 8.50745 | -4.51651 | 2.60E-05 | 7.89E-05 | 1.988379 |
| MTL5 | 0.772066 | 7.180838 | 4.515179 | 2.61E-05 | 7.93E-05 | 1.983762 |
| ARG1 | -1.16243 | 7.689527 | -4.51513 | 2.61E-05 | 7.93E-05 | 1.983577 |
| CASP9 | 0.842687 | 8.484067 | 4.513009 | 2.63E-05 | 7.98E-05 | 1.976217 |
| LOC15816 | -0.81421 | 7.697686 | -4.51155 | 2.64E-05 | 8.02E-05 | 1.971137 |
| CDKN2C | 0.761767 | 7.858466 | 4.510341 | 2.66E-05 | 8.06E-05 | 1.966942 |
| FBXO31 | -1.05177 | 9.18008 | -4.50952 | 2.66E-05 | 8.08E-05 | 1.964092 |
| CGNL1 | -0.94897 | 8.328956 | -4.50838 | 2.68E-05 | 8.11E-05 | 1.96011 |
| FIS1 | -0.73574 | 7.232966 | -4.50198 | 2.74E-05 | 8.28E-05 | 1.937886 |
| RDH16 | -1.17752 | 8.7186 | -4.49934 | 2.76E-05 | 8.36E-05 | 1.928742 |
| IL6R | -0.72836 | 7.650504 | -4.49866 | 2.77E-05 | 8.38E-05 | 1.926381 |
| NDRG4 | 0.77166 | 7.005161 | 4.496311 | 2.79E-05 | 8.44E-05 | 1.918215 |
| STMN2 | 1.159143 | 8.19882 | 4.493925 | 2.82E-05 | 8.50E-05 | 1.909938 |
| ABCG8 | -0.87602 | 8.02717 | -4.49322 | 2.83E-05 | 8.52E-05 | 1.907505 |
| DAPL1 | 0.83211 | 6.887095 | 4.487475 | 2.89E-05 | 8.68E-05 | 1.887572 |
| CSK | -0.86551 | 8.408104 | -4.48465 | 2.91E-05 | 8.76E-05 | 1.877781 |
| P2RX1 | 0.858757 | 7.354453 | 4.48389 | 2.92E-05 | 8.78E-05 | 1.875147 |
| INPPL1 | -0.89152 | 8.088759 | -4.48321 | 2.93E-05 | 8.80E-05 | 1.8728 |
| LOC10013 | 1.256848 | 8.206554 | 4.483033 | 2.93E-05 | 8.80E-05 | 1.872176 |
| MTMR2 | 0.721242 | 7.590164 | 4.481607 | 2.95E-05 | 8.84E-05 | 1.867236 |
| HERC2P2 | -0.97305 | 8.462957 | -4.48092 | 2.95E-05 | 8.86E-05 | 1.864865 |
| SNORA14. | -0.81908 | 7.854674 | -4.47977 | 2.97E-05 | 8.90E-05 | 1.860884 |
| INHBE | -0.80448 | 7.449879 | -4.47787 | 2.99E-05 | 8.95E-05 | 1.854289 |
| PAX5 | 0.868067 | 7.136501 | 4.471166 | 3.06E-05 | 9.15E-05 | 1.831093 |
| KCNIP3 | 0.736518 | 7.159355 | 4.470306 | 3.07E-05 | 9.18E-05 | 1.828117 |
| FAM153C | 0.844626 | 6.961914 | 4.469225 | 3.08E-05 | 9.21E-05 | 1.824376 |
| SGK | -1.18059 | 9.088182 | -4.4682 | 3.09E-05 | 9.24E-05 | 1.820822 |
| SNORD91 | -0.92956 | 8.079034 | -4.46753 | 3.10E-05 | 9.26E-05 | 1.818524 |
| BHLHB2 | -1.2052 | 10.1097 | -4.46748 | 3.10E-05 | 9.26E-05 | 1.818345 |
| SAPS1 | -0.81191 | 8.417884 | -4.46621 | 3.12E-05 | 9.29E-05 | 1.813941 |
| C20orf103 | 0.987211 | 7.316063 | 4.46578 | 3.12E-05 | 9.30E-05 | 1.812465 |
| GINS3 | 1.056795 | 7.767344 | 4.465068 | 3.13E-05 | 9.32E-05 | 1.810003 |
| SRGN | -1.04902 | 8.392688 | -4.46282 | 3.15E-05 | 9.39E-05 | 1.802245 |
| ETFDH | -0.81615 | 8.669569 | -4.46233 | 3.16E-05 | 9.40E-05 | 1.800541 |
| TTYH1 | 0.810379 | 7.137886 | 4.461821 | 3.16E-05 | 9.42E-05 | 1.798779 |
| LIPC | -0.81901 | 8.248301 | -4.46164 | 3.17E-05 | 9.42E-05 | 1.798144 |
| BRI3 | -0.89434 | 7.866283 | -4.45774 | 3.21E-05 | 9.55E-05 | 1.784684 |
| SCARNA6 | -1.14902 | 9.127313 | -4.45558 | 3.24E-05 | 9.62E-05 | 1.777208 |

| | | | | | | |
|---|---|---|---|---|---|---|
| C6orf105 | 0.921067 | 7.089992 | 4.451434 | 3.29E-05 | 9.75E-05 | 1.762906 |
| C6orf168 | 0.888164 | 6.964268 | 4.451073 | 3.29E-05 | 9.76E-05 | 1.761662 |
| NPIP | 0.712647 | 8.417586 | 4.450294 | 3.30E-05 | 9.79E-05 | 1.758973 |
| CIDEB | -0.8313 | 9.210948 | -4.44223 | 3.40E-05 | 0.000101 | 1.731165 |
| KRT33B | 0.72293 | 6.877774 | 4.440872 | 3.41E-05 | 0.000101 | 1.726476 |
| PGM1 | -0.82505 | 8.262535 | -4.43975 | 3.43E-05 | 0.000101 | 1.722604 |
| C16orf93 | 0.828924 | 7.91385 | 4.438861 | 3.44E-05 | 0.000102 | 1.719544 |
| NUBP1 | -0.74033 | 8.275576 | -4.43629 | 3.47E-05 | 0.000103 | 1.710691 |
| KCTD14 | 0.853766 | 7.40114 | 4.435879 | 3.47E-05 | 0.000103 | 1.709271 |
| KIF24 | 0.703874 | 6.927798 | 4.434493 | 3.49E-05 | 0.000103 | 1.704496 |
| UBE2M | -0.7661 | 9.375247 | -4.43226 | 3.52E-05 | 0.000104 | 1.69679 |
| ZNF70 | 0.802344 | 7.224439 | 4.432234 | 3.52E-05 | 0.000104 | 1.696718 |
| ATP5G1 | -0.7107 | 8.728964 | -4.42617 | 3.60E-05 | 0.000106 | 1.675828 |
| FAM155B | 0.959584 | 7.300206 | 4.425857 | 3.60E-05 | 0.000106 | 1.674769 |
| MYBPH | 1.289488 | 8.00878 | 4.424458 | 3.62E-05 | 0.000107 | 1.669954 |
| SPC24 | 0.907649 | 10.04046 | 4.418931 | 3.69E-05 | 0.000109 | 1.650949 |
| MRPL41 | -0.93065 | 8.959928 | -4.41786 | 3.71E-05 | 0.000109 | 1.647259 |
| APOC4 | -1.26299 | 8.781921 | -4.41706 | 3.72E-05 | 0.000109 | 1.644523 |
| TLX1 | 0.938685 | 7.955282 | 4.416541 | 3.72E-05 | 0.000109 | 1.642733 |
| GFPT2 | 0.859814 | 7.129005 | 4.411957 | 3.79E-05 | 0.000111 | 1.626982 |
| ZFP14 | 0.855501 | 8.47877 | 4.409459 | 3.82E-05 | 0.000112 | 1.618404 |
| FAM193B | -0.74648 | 8.68938 | -4.40285 | 3.91E-05 | 0.000114 | 1.595727 |
| SNAPC4 | -1.47289 | 9.12836 | -4.40166 | 3.93E-05 | 0.000115 | 1.591629 |
| C8orf4 | -1.02745 | 8.409585 | -4.39843 | 3.97E-05 | 0.000116 | 1.580549 |
| MAPK13 | 1.305965 | 8.425866 | 4.396606 | 4.00E-05 | 0.000117 | 1.574309 |
| ESPN | -1.10073 | 11.1085 | -4.39619 | 4.00E-05 | 0.000117 | 1.572877 |
| GTF3A | -0.98898 | 8.10084 | -4.38694 | 4.14E-05 | 0.000121 | 1.541187 |
| CRYAA | -0.81418 | 7.401787 | -4.38577 | 4.16E-05 | 0.000121 | 1.537182 |
| C20orf52 | -0.9595 | 7.923994 | -4.38256 | 4.20E-05 | 0.000122 | 1.526197 |
| ANKRD57 | -1.37589 | 8.479903 | -4.37883 | 4.26E-05 | 0.000124 | 1.513441 |
| SNORD83 | -1.53074 | 10.61077 | -4.3773 | 4.28E-05 | 0.000124 | 1.508197 |
| HNRNPH1 | -0.79044 | 8.323989 | -4.37684 | 4.29E-05 | 0.000125 | 1.506613 |
| SNAR-I | 1.049196 | 7.098675 | 4.37425 | 4.33E-05 | 0.000126 | 1.497772 |
| JAM3 | -0.73327 | 7.756076 | -4.37424 | 4.33E-05 | 0.000126 | 1.497748 |
| PSMB3 | -1.09579 | 9.829581 | -4.3699 | 4.40E-05 | 0.000127 | 1.482898 |
| C17orf65 | 0.987046 | 7.509785 | 4.368312 | 4.42E-05 | 0.000128 | 1.477483 |
| SGCA | 1.026503 | 7.878939 | 4.368295 | 4.42E-05 | 0.000128 | 1.477422 |
| FLJ44653 | 0.80594 | 6.986573 | 4.367133 | 4.44E-05 | 0.000129 | 1.473453 |
| PRDX6 | -0.99039 | 8.055075 | -4.36547 | 4.47E-05 | 0.000129 | 1.467779 |
| FH | -1.29841 | 9.739339 | -4.36429 | 4.49E-05 | 0.00013 | 1.463732 |
| KIF21B | 0.827344 | 7.708827 | 4.364181 | 4.49E-05 | 0.00013 | 1.463373 |
| PSPN | 0.792237 | 6.871379 | 4.364165 | 4.49E-05 | 0.00013 | 1.463319 |
| LOC55908 | -0.82426 | 7.994117 | -4.36208 | 4.52E-05 | 0.000131 | 1.456202 |
| CFL2 | -0.73409 | 7.760098 | -4.36187 | 4.53E-05 | 0.000131 | 1.455484 |
| FEM1A | -0.97733 | 8.774989 | -4.36161 | 4.53E-05 | 0.000131 | 1.454592 |
| AGMAT | -0.93277 | 7.836825 | -4.35984 | 4.56E-05 | 0.000132 | 1.448557 |
| CRP | -1.57157 | 8.689964 | -4.3597 | 4.56E-05 | 0.000132 | 1.448066 |
| AFMID | 0.756822 | 8.74799 | 4.359695 | 4.56E-05 | 0.000132 | 1.448061 |
| C1QTNF9I | 0.82911 | 7.1617 | 4.358753 | 4.58E-05 | 0.000132 | 1.444849 |
| RAB17 | -1.30668 | 9.078918 | -4.35842 | 4.58E-05 | 0.000132 | 1.443701 |
| STOML2 | -1.15634 | 9.912077 | -4.35282 | 4.67E-05 | 0.000135 | 1.424626 |
| UBE1DC1 | 0.728784 | 8.84876 | 4.352273 | 4.68E-05 | 0.000135 | 1.422749 |
| PCNP | -0.96773 | 9.161599 | -4.35212 | 4.69E-05 | 0.000135 | 1.422217 |
| HLA-DRB1 | -1.0535 | 7.304468 | -4.35188 | 4.69E-05 | 0.000135 | 1.42142 |
| PPP1R1B | 1.060324 | 7.476861 | 4.350109 | 4.72E-05 | 0.000136 | 1.415372 |
| MAFA | 1.23095 | 7.425133 | 4.349901 | 4.72E-05 | 0.000136 | 1.414664 |
| AMT | -1.00985 | 8.388028 | -4.34883 | 4.74E-05 | 0.000136 | 1.410999 |
| RUNX3 | 0.854802 | 7.840809 | 4.34868 | 4.74E-05 | 0.000136 | 1.410502 |

| | | | | | | |
|---|---|---|---|---|---|---|
| FMNL3 | 0.869162 | 8.716318 | 4.345828 | 4.79E-05 | 0.000137 | 1.400787 |
| EBP | -0.94628 | 8.482687 | -4.34547 | 4.80E-05 | 0.000138 | 1.39956 |
| SDC4 | -0.77032 | 7.494194 | -4.34431 | 4.82E-05 | 0.000138 | 1.395601 |
| C2orf89 | 1.071056 | 8.097742 | 4.343919 | 4.83E-05 | 0.000138 | 1.394282 |
| C1orf85 | -0.97295 | 7.796663 | -4.34078 | 4.88E-05 | 0.00014 | 1.38358 |
| CCBP2 | -1.0555 | 8.581402 | -4.3377 | 4.93E-05 | 0.000141 | 1.373119 |
| EIF2S2 | 0.787744 | 7.806521 | 4.337067 | 4.94E-05 | 0.000141 | 1.370959 |
| REEP6 | -1.0359 | 8.252825 | -4.33393 | 5.00E-05 | 0.000143 | 1.360274 |
| NECAB3 | -0.75097 | 7.856005 | -4.3324 | 5.03E-05 | 0.000143 | 1.355077 |
| TMBIM4 | -0.72453 | 7.752119 | -4.33215 | 5.03E-05 | 0.000144 | 1.354248 |
| SCML4 | 1.112266 | 7.884251 | 4.331344 | 5.05E-05 | 0.000144 | 1.351497 |
| HEY2 | -0.95473 | 7.935809 | -4.33099 | 5.05E-05 | 0.000144 | 1.350303 |
| C20orf54 | 1.108397 | 7.60512 | 4.327671 | 5.11E-05 | 0.000146 | 1.339009 |
| GALNT11 | -0.74472 | 7.590353 | -4.32708 | 5.12E-05 | 0.000146 | 1.336998 |
| RASL11B | 0.78239 | 7.545739 | 4.326803 | 5.13E-05 | 0.000146 | 1.33606 |
| ACR | 1.276715 | 8.203833 | 4.326081 | 5.14E-05 | 0.000146 | 1.333607 |
| TRAPPC3 | -1.53504 | 8.98824 | -4.32531 | 5.15E-05 | 0.000147 | 1.330973 |
| RUNDC3B | 1.231662 | 9.318157 | 4.323927 | 5.18E-05 | 0.000147 | 1.326291 |
| YIPF5 | 0.750439 | 7.369947 | 4.323733 | 5.18E-05 | 0.000148 | 1.325633 |
| ANP32B | -1.27115 | 8.364854 | -4.32123 | 5.23E-05 | 0.000149 | 1.317131 |
| TTTY23 | 0.930465 | 7.069722 | 4.315584 | 5.34E-05 | 0.000151 | 1.297967 |
| C21orf129 | 1.215019 | 7.625439 | 4.314389 | 5.36E-05 | 0.000152 | 1.293914 |
| SNORA11 | -0.919 | 7.898056 | -4.31386 | 5.37E-05 | 0.000152 | 1.292135 |
| ASPG | 0.968078 | 8.473526 | 4.312884 | 5.39E-05 | 0.000153 | 1.28881 |
| CACYBP | 0.773543 | 8.328166 | 4.31114 | 5.42E-05 | 0.000154 | 1.282894 |
| STK38 | -1.05814 | 8.520028 | -4.30206 | 5.60E-05 | 0.000158 | 1.252106 |
| Gcom1 | 0.723998 | 7.355909 | 4.300399 | 5.63E-05 | 0.000159 | 1.246496 |
| POLR1A | 0.744666 | 8.257359 | 4.300162 | 5.63E-05 | 0.000159 | 1.245694 |
| CBFA2T3 | 0.752549 | 7.538719 | 4.300156 | 5.63E-05 | 0.000159 | 1.245676 |
| TMEM176 | -1.49176 | 9.019065 | -4.29762 | 5.69E-05 | 0.000161 | 1.237105 |
| BAI2 | 0.756505 | 6.981683 | 4.296423 | 5.71E-05 | 0.000161 | 1.233037 |
| TRAIP | 0.82 | 7.404536 | 4.294837 | 5.74E-05 | 0.000162 | 1.227668 |
| MFAP4 | -0.99885 | 8.379237 | -4.29359 | 5.77E-05 | 0.000163 | 1.223458 |
| MIR612 | -1.14779 | 9.60676 | -4.28842 | 5.87E-05 | 0.000165 | 1.205954 |
| LOC44015 | 1.115857 | 10.11721 | 4.287001 | 5.90E-05 | 0.000166 | 1.201169 |
| MAF1 | -0.894 | 8.394843 | -4.2833 | 5.98E-05 | 0.000168 | 1.188671 |
| GSTT2 | 0.832555 | 7.338492 | 4.282766 | 5.99E-05 | 0.000168 | 1.186858 |
| CTH | -0.78051 | 7.461584 | -4.27819 | 6.09E-05 | 0.000171 | 1.17141 |
| RRN3P2 | 1.072483 | 7.881316 | 4.275672 | 6.14E-05 | 0.000172 | 1.162903 |
| SNAI3 | 0.78477 | 7.147792 | 4.26761 | 6.32E-05 | 0.000177 | 1.135707 |
| P15RS | -0.89004 | 7.609124 | -4.26724 | 6.33E-05 | 0.000177 | 1.134459 |
| CPSF4 | -1.28478 | 9.406361 | -4.26473 | 6.39E-05 | 0.000179 | 1.125985 |
| ALPK2 | -1.08122 | 8.376279 | -4.25957 | 6.50E-05 | 0.000182 | 1.108625 |
| TFR2 | -0.87718 | 10.4714 | -4.25867 | 6.52E-05 | 0.000182 | 1.105571 |
| SLC22A14 | 0.74466 | 7.070187 | 4.255702 | 6.59E-05 | 0.000184 | 1.095591 |
| CPLX1 | 1.041651 | 9.751269 | 4.252271 | 6.67E-05 | 0.000186 | 1.084042 |
| NDUFB11 | -1.11796 | 8.815935 | -4.25009 | 6.72E-05 | 0.000187 | 1.076702 |
| ADSSL1 | 0.733138 | 7.987098 | 4.248262 | 6.77E-05 | 0.000188 | 1.070556 |
| DUSP19 | 0.743905 | 7.645716 | 4.245303 | 6.84E-05 | 0.00019 | 1.06061 |
| ARIH2 | -0.7032 | 7.783322 | -4.24363 | 6.88E-05 | 0.000191 | 1.054982 |
| FAM39DP | 0.738239 | 9.219179 | 4.242778 | 6.90E-05 | 0.000191 | 1.052121 |
| FLNC | -1.02205 | 7.491205 | -4.23922 | 6.99E-05 | 0.000193 | 1.040166 |
| FLJ39827 | -0.73678 | 7.854908 | -4.23601 | 7.06E-05 | 0.000195 | 1.029377 |
| KIAA2010 | -0.84697 | 8.587952 | -4.2357 | 7.07E-05 | 0.000196 | 1.028337 |
| CYP2R1 | 0.703946 | 7.616326 | 4.234498 | 7.10E-05 | 0.000196 | 1.024316 |
| FOXC1 | 1.030338 | 8.314666 | 4.234043 | 7.11E-05 | 0.000197 | 1.022789 |
| C14orf178 | 0.816174 | 7.152771 | 4.233984 | 7.11E-05 | 0.000197 | 1.02259 |
| XYLT1 | -0.92936 | 7.356715 | -4.23223 | 7.16E-05 | 0.000198 | 1.016688 |

| | | | | | | |
|---|---|---|---|---|---|---|
| G6PC | -0.94262 | 9.844543 | -4.23086 | 7.19E-05 | 0.000199 | 1.012109 |
| KLHDC7A | 0.944772 | 7.364572 | 4.23013 | 7.21E-05 | 0.000199 | 1.009657 |
| LRRC32 | -1.06409 | 8.826096 | -4.22994 | 7.22E-05 | 0.000199 | 1.009017 |
| C19orf70 | -0.73901 | 8.567584 | -4.22821 | 7.26E-05 | 0.0002 | 1.003225 |
| GBP7 | -0.79999 | 7.720415 | -4.22424 | 7.36E-05 | 0.000203 | 0.989892 |
| KIF1B | -0.73805 | 8.405992 | -4.22394 | 7.37E-05 | 0.000203 | 0.988887 |
| C8A | -1.28759 | 8.738772 | -4.22321 | 7.39E-05 | 0.000204 | 0.986466 |
| LOC72803 | -0.82554 | 8.452729 | -4.22075 | 7.45E-05 | 0.000205 | 0.978196 |
| DNM1 | 0.980133 | 8.328394 | 4.219594 | 7.48E-05 | 0.000206 | 0.974341 |
| ETV6 | 0.925224 | 9.992885 | 4.212834 | 7.66E-05 | 0.000211 | 0.951705 |
| BEST4 | 0.783742 | 7.159929 | 4.208915 | 7.77E-05 | 0.000213 | 0.938593 |
| ITPKA | -0.72089 | 7.490285 | -4.20735 | 7.81E-05 | 0.000214 | 0.933358 |
| SNORA37 | -1.32507 | 9.468698 | -4.20696 | 7.82E-05 | 0.000215 | 0.93206 |
| CRYBB2 | 0.994617 | 8.180468 | 4.205902 | 7.85E-05 | 0.000215 | 0.928517 |
| LOC72923 | 0.964153 | 7.923324 | 4.205557 | 7.86E-05 | 0.000216 | 0.927363 |
| BLCAP | -0.78968 | 7.819493 | -4.20479 | 7.88E-05 | 0.000216 | 0.924795 |
| MIR1180 | 0.769678 | 6.865013 | 4.199018 | 8.04E-05 | 0.00022 | 0.905513 |
| CBWD3 | 1.417072 | 9.013869 | 4.196525 | 8.11E-05 | 0.000222 | 0.897186 |
| EP300 | -0.82987 | 8.521574 | -4.19461 | 8.17E-05 | 0.000223 | 0.890804 |
| SNORA42 | -1.25206 | 9.758843 | -4.19163 | 8.25E-05 | 0.000225 | 0.880834 |
| IGF2BP2 | 1.318542 | 9.921718 | 4.189825 | 8.30E-05 | 0.000227 | 0.874826 |
| LOC44092 | 1.04115 | 8.73192 | 4.18954 | 8.31E-05 | 0.000227 | 0.873875 |
| ADAMTS2 | -0.7628 | 7.966875 | -4.18953 | 8.31E-05 | 0.000227 | 0.873847 |
| MGRN1 | -0.70176 | 8.942579 | -4.17909 | 8.62E-05 | 0.000234 | 0.839049 |
| SPOCK1 | 0.867264 | 7.588824 | 4.178917 | 8.63E-05 | 0.000234 | 0.838464 |
| RAB33B | -0.70246 | 7.187338 | -4.17372 | 8.78E-05 | 0.000239 | 0.821158 |
| ODZ2 | 1.166654 | 7.798332 | 4.172959 | 8.81E-05 | 0.000239 | 0.818625 |
| CCT2 | -0.72675 | 8.511313 | -4.1705 | 8.88E-05 | 0.000241 | 0.81043 |
| C6orf154 | 0.764711 | 7.190074 | 4.166345 | 9.01E-05 | 0.000244 | 0.796624 |
| FLJ46347 | 0.86289 | 6.977839 | 4.163006 | 9.12E-05 | 0.000247 | 0.785523 |
| KIF3C | 0.743787 | 7.057737 | 4.162422 | 9.14E-05 | 0.000247 | 0.783584 |
| USP54 | 0.979155 | 8.053157 | 4.159876 | 9.22E-05 | 0.000249 | 0.775122 |
| MX1 | -1.2972 | 11.37516 | -4.15964 | 9.23E-05 | 0.000249 | 0.774334 |
| MRPL14 | -0.93838 | 8.594578 | -4.15927 | 9.24E-05 | 0.000249 | 0.77311 |
| KCNG2 | 0.916143 | 7.102032 | 4.158703 | 9.26E-05 | 0.00025 | 0.771226 |
| ATP6V1E2 | 0.881025 | 7.589041 | 4.157851 | 9.28E-05 | 0.00025 | 0.768396 |
| TUBB | -0.90957 | 8.276792 | -4.15162 | 9.49E-05 | 0.000256 | 0.74771 |
| KLHL35 | 0.730704 | 7.324744 | 4.150852 | 9.51E-05 | 0.000256 | 0.745162 |
| SH2D3A | 0.913352 | 7.245169 | 4.148713 | 9.58E-05 | 0.000258 | 0.738068 |
| UNC13A | 0.702563 | 7.169328 | 4.140405 | 9.86E-05 | 0.000265 | 0.710528 |
| CAPN12 | -0.83501 | 8.377519 | -4.13885 | 9.92E-05 | 0.000266 | 0.705381 |
| PGLS | -0.75143 | 8.521597 | -4.13835 | 9.93E-05 | 0.000266 | 0.703736 |
| ORM2 | -1.2377 | 9.730823 | -4.13729 | 9.97E-05 | 0.000267 | 0.700201 |
| GSTM2 | -1.31081 | 7.989682 | -4.13587 | 0.0001 | 0.000268 | 0.695517 |
| TMEM85 | -0.80991 | 7.67516 | -4.13561 | 0.0001 | 0.000268 | 0.694659 |
| MIR92B | 0.86856 | 7.147987 | 4.135295 | 0.0001 | 0.000269 | 0.693604 |
| ZYG11A | 1.263833 | 8.305851 | 4.134228 | 0.000101 | 0.00027 | 0.690074 |
| RNF31 | 1.037516 | 9.149339 | 4.126592 | 0.000103 | 0.000276 | 0.664812 |
| NUPR1 | -1.10449 | 10.02352 | -4.12578 | 0.000104 | 0.000277 | 0.662123 |
| DKFZp564 | 1.617789 | 7.939436 | 4.1219 | 0.000105 | 0.00028 | 0.649304 |
| BRD4 | -0.88012 | 8.529266 | -4.12137 | 0.000105 | 0.00028 | 0.647558 |
| FOXC2 | 0.928763 | 7.639953 | 4.121046 | 0.000105 | 0.000281 | 0.646482 |
| C20orf118 | 0.871515 | 7.178562 | 4.120704 | 0.000106 | 0.000281 | 0.645353 |
| ATP5A1 | -0.82638 | 9.041758 | -4.11979 | 0.000106 | 0.000282 | 0.642342 |
| TAOK1 | -0.73734 | 8.319897 | -4.1184 | 0.000106 | 0.000283 | 0.637735 |
| ADAMTS1 | -0.76792 | 7.17301 | -4.11646 | 0.000107 | 0.000285 | 0.631344 |
| LCN8 | 0.874126 | 6.829975 | 4.114755 | 0.000108 | 0.000286 | 0.625708 |
| TIMM10 | -1.15465 | 8.413158 | -4.11183 | 0.000109 | 0.000289 | 0.616055 |

| | | | | | | |
|---|---|---|---|---|---|---|
| NNMT | -1.28171 | 8.227177 | -4.10751 | 0.000111 | 0.000293 | 0.60181 |
| SFRP4 | 0.86493 | 8.01774 | 4.107022 | 0.000111 | 0.000293 | 0.600199 |
| ORC1L | 0.91527 | 7.17593 | 4.104385 | 0.000112 | 0.000296 | 0.591505 |
| ARPC1A | -0.87772 | 8.716132 | -4.10166 | 0.000113 | 0.000298 | 0.582539 |
| UGT2B10 | -0.82378 | 8.333734 | -4.09829 | 0.000114 | 0.000301 | 0.571416 |
| CXCR4 | -0.98976 | 8.531272 | -4.09698 | 0.000115 | 0.000303 | 0.567111 |
| LCNL1 | 0.721035 | 7.06695 | 4.096241 | 0.000115 | 0.000303 | 0.56468 |
| ITLN2 | 0.873666 | 7.720582 | 4.092253 | 0.000117 | 0.000307 | 0.551555 |
| TMSL3 | -0.82613 | 7.987279 | -4.09023 | 0.000117 | 0.000309 | 0.544903 |
| TXNDC12 | -0.77269 | 7.633402 | -4.08958 | 0.000118 | 0.00031 | 0.542755 |
| TRIP6 | -1.23449 | 8.627833 | -4.08779 | 0.000118 | 0.000311 | 0.536867 |
| SFTA1P | 0.77865 | 7.19138 | 4.085682 | 0.000119 | 0.000313 | 0.529947 |
| LPIN2 | -0.83742 | 8.293217 | -4.08527 | 0.000119 | 0.000314 | 0.528583 |
| RBM15 | -1.51639 | 9.63858 | -4.08434 | 0.00012 | 0.000314 | 0.525535 |
| SEMA6D | 0.804802 | 7.37973 | 4.08393 | 0.00012 | 0.000315 | 0.524189 |
| MIR874 | 1.39764 | 7.923218 | 4.082734 | 0.00012 | 0.000316 | 0.52026 |
| EFR3A | -1.04639 | 8.194365 | -4.0821 | 0.000121 | 0.000317 | 0.518191 |
| CD180 | 0.776409 | 7.851133 | 4.077531 | 0.000123 | 0.000321 | 0.503172 |
| WNT7A | 1.322632 | 7.552323 | 4.075486 | 0.000123 | 0.000323 | 0.496461 |
| F5 | -0.84651 | 7.85018 | -4.07383 | 0.000124 | 0.000325 | 0.491035 |
| RPL30 | -1.11068 | 9.5909 | -4.0738 | 0.000124 | 0.000325 | 0.490926 |
| COPE | -0.85249 | 7.805172 | -4.06725 | 0.000127 | 0.000332 | 0.469455 |
| MIR937 | 1.478099 | 8.83137 | 4.065557 | 0.000128 | 0.000333 | 0.463898 |
| LOC4923C | 0.801207 | 7.071541 | 4.065481 | 0.000128 | 0.000333 | 0.463649 |
| HMGCL | -1.11566 | 9.74499 | -4.06226 | 0.000129 | 0.000337 | 0.453089 |
| MX2 | 0.868486 | 8.002183 | 4.062054 | 0.000129 | 0.000337 | 0.452422 |
| YWHAQ | -0.73867 | 8.177897 | -4.05943 | 0.00013 | 0.00034 | 0.443821 |
| MTSS1L | -0.71106 | 8.79442 | -4.05785 | 0.000131 | 0.000341 | 0.438646 |
| CA4 | 0.983086 | 7.754872 | 4.056678 | 0.000132 | 0.000343 | 0.43482 |
| POMT2 | 0.745384 | 8.388193 | 4.054765 | 0.000133 | 0.000345 | 0.428561 |
| FBLN1 | 0.754023 | 8.237174 | 4.052228 | 0.000134 | 0.000348 | 0.42026 |
| SDCCAG1 | -0.74877 | 7.821203 | -4.04611 | 0.000137 | 0.000354 | 0.400273 |
| PPCDC | 0.717925 | 7.139108 | 4.045072 | 0.000137 | 0.000355 | 0.396869 |
| ERAL1 | -0.84308 | 10.74875 | -4.04237 | 0.000138 | 0.000359 | 0.388036 |
| ZFP3 | 0.986939 | 7.741641 | 4.038729 | 0.00014 | 0.000363 | 0.376155 |
| C1QTNF4 | 0.794516 | 6.843329 | 4.032871 | 0.000143 | 0.000369 | 0.357044 |
| ROD1 | -0.71411 | 8.849735 | -4.0291 | 0.000145 | 0.000374 | 0.344764 |
| FAM71E1 | 0.744417 | 7.203789 | 4.027874 | 0.000145 | 0.000375 | 0.340754 |
| RGS5 | -0.70233 | 8.507792 | -4.02474 | 0.000147 | 0.000379 | 0.330542 |
| LOC5951C | -0.97223 | 7.969631 | -4.01845 | 0.00015 | 0.000387 | 0.310051 |
| SNORD10 | -0.88203 | 7.564065 | -4.01836 | 0.00015 | 0.000387 | 0.309775 |
| LOC88523 | -0.8469 | 7.936658 | -4.01692 | 0.000151 | 0.000388 | 0.305095 |
| AHCTF1 | 0.737679 | 8.753614 | 4.013489 | 0.000153 | 0.000393 | 0.293928 |
| ZNF546 | 0.70163 | 7.154583 | 4.013199 | 0.000153 | 0.000393 | 0.292985 |
| PCDHGA2 | 1.006601 | 7.470603 | 4.013017 | 0.000153 | 0.000393 | 0.292392 |
| CYP26B1 | 1.116863 | 7.506382 | 4.012922 | 0.000153 | 0.000393 | 0.292085 |
| HEBP1 | -1.09029 | 8.771581 | -4.01242 | 0.000153 | 0.000394 | 0.29044 |
| FAM110B | 0.844678 | 8.000835 | 4.009868 | 0.000155 | 0.000397 | 0.282156 |
| SNORD88 | -0.77263 | 7.894455 | -4.00429 | 0.000158 | 0.000404 | 0.264047 |
| RFP | -0.73299 | 8.427379 | -4.00426 | 0.000158 | 0.000404 | 0.263927 |
| MYBPHL | 0.727912 | 7.116658 | 4.001154 | 0.000159 | 0.000408 | 0.253858 |
| PNKD | -0.73594 | 8.826801 | -4.00087 | 0.000159 | 0.000408 | 0.252922 |
| MAPKAPK | -1.09358 | 8.717964 | -4.00061 | 0.00016 | 0.000408 | 0.252079 |
| CLCN4 | 0.705918 | 7.514715 | 3.997283 | 0.000161 | 0.000413 | 0.241297 |
| C15orf42 | 0.708056 | 7.110895 | 3.995531 | 0.000162 | 0.000415 | 0.235617 |
| SURF4 | -0.88789 | 8.406413 | -3.99372 | 0.000163 | 0.000417 | 0.229728 |
| FLJ44005 | 0.700251 | 7.024029 | 3.991231 | 0.000165 | 0.00042 | 0.221676 |
| AMACR | -0.70552 | 7.777122 | -3.99006 | 0.000165 | 0.000422 | 0.21788 |

| | | | | | | |
|---|---|---|---|---|---|---|
| KIAA0251 | -0.84391 | 9.756589 | -3.98933 | 0.000166 | 0.000423 | 0.215518 |
| ZNF775 | 0.75431 | 7.475545 | 3.984439 | 0.000169 | 0.000429 | 0.199678 |
| SNORD55 | -1.3947 | 10.92476 | -3.98232 | 0.00017 | 0.000432 | 0.192807 |
| RNU1-5 | -0.87304 | 12.47211 | -3.98015 | 0.000171 | 0.000435 | 0.185787 |
| SNORA10 | -0.78312 | 11.40234 | -3.97954 | 0.000171 | 0.000436 | 0.183823 |
| HLA-A29.: | -1.62593 | 8.92829 | -3.97854 | 0.000172 | 0.000437 | 0.180576 |
| BTAF1 | -0.81734 | 7.634872 | -3.97503 | 0.000174 | 0.000442 | 0.169256 |
| C19orf23 | 1.060714 | 8.405246 | 3.973941 | 0.000175 | 0.000444 | 0.165721 |
| ISOC2 | -0.76968 | 7.820999 | -3.97274 | 0.000175 | 0.000445 | 0.161832 |
| C2orf34 | 0.980379 | 8.857888 | 3.972286 | 0.000176 | 0.000446 | 0.160373 |
| NUDT18 | 0.89119 | 8.014071 | 3.969173 | 0.000178 | 0.00045 | 0.150317 |
| TIMM17B | -0.94152 | 10.00736 | -3.96894 | 0.000178 | 0.00045 | 0.149577 |
| FSTL4 | 0.729655 | 7.078044 | 3.968369 | 0.000178 | 0.000451 | 0.147723 |
| DCAF12L2 | 0.937139 | 7.039929 | 3.966454 | 0.000179 | 0.000453 | 0.141538 |
| EIF1 | -0.73745 | 7.84875 | -3.96602 | 0.00018 | 0.000454 | 0.140122 |
| DYNC1LI2 | -0.8999 | 8.779555 | -3.96551 | 0.00018 | 0.000455 | 0.138498 |
| RALGPS1 | 0.725234 | 7.801576 | 3.965476 | 0.00018 | 0.000455 | 0.138382 |
| CEACAM2 | 0.708284 | 7.362152 | 3.961783 | 0.000182 | 0.00046 | 0.126465 |
| POU5F1P1 | 0.949288 | 7.173411 | 3.960634 | 0.000183 | 0.000462 | 0.12276 |
| ABCA2 | 0.745265 | 7.389957 | 3.959217 | 0.000184 | 0.000464 | 0.118189 |
| RGS2 | -0.72079 | 7.162055 | -3.95715 | 0.000185 | 0.000466 | 0.111518 |
| BHMT | -1.41911 | 10.06164 | -3.95366 | 0.000187 | 0.000472 | 0.100276 |
| MTHFSD | 0.706425 | 7.635553 | 3.951743 | 0.000188 | 0.000474 | 0.094102 |
| LOC73001 | 0.751614 | 7.075551 | 3.95154 | 0.000189 | 0.000475 | 0.09345 |
| EDNRA | -1.13468 | 8.454253 | -3.94985 | 0.00019 | 0.000477 | 0.088018 |
| HTRA1 | -0.85247 | 8.862374 | -3.94763 | 0.000191 | 0.00048 | 0.080853 |
| SELL | 0.98851 | 8.073745 | 3.946761 | 0.000192 | 0.000481 | 0.078064 |
| MT1F | -0.78837 | 7.493398 | -3.9442 | 0.000193 | 0.000485 | 0.069818 |
| HRIHFB21 | -1.00647 | 7.66009 | -3.94417 | 0.000193 | 0.000485 | 0.069738 |
| ISLR | 0.943811 | 8.049756 | 3.940455 | 0.000196 | 0.00049 | 0.057779 |
| IGSF21 | 1.165863 | 8.377316 | 3.936394 | 0.000198 | 0.000497 | 0.044729 |
| NDUFS3 | -1.13772 | 9.698488 | -3.93335 | 0.000201 | 0.000501 | 0.034945 |
| SNORA60 | -1.39453 | 9.410811 | -3.93211 | 0.000201 | 0.000503 | 0.03097 |
| DCTN2 | -1.17444 | 8.352376 | -3.92383 | 0.000207 | 0.000516 | 0.004403 |
| FNBP1 | -0.86239 | 8.229865 | -3.92173 | 0.000209 | 0.00052 | -0.00233 |
| GNG7 | 0.837307 | 8.161535 | 3.921228 | 0.000209 | 0.00052 | -0.00394 |
| GNAS | -0.71753 | 8.818084 | -3.92061 | 0.000209 | 0.000521 | -0.00593 |
| MRAP | 0.80675 | 7.420912 | 3.917365 | 0.000212 | 0.000527 | -0.01632 |
| C17orf97 | 0.745908 | 8.373296 | 3.916984 | 0.000212 | 0.000527 | -0.01754 |
| C6orf15 | 0.84528 | 7.160648 | 3.91598 | 0.000213 | 0.000529 | -0.02075 |
| OLFML2B | 0.866693 | 7.985805 | 3.914963 | 0.000213 | 0.00053 | -0.02401 |
| NAGLU | -0.77414 | 7.83731 | -3.91143 | 0.000216 | 0.000536 | -0.03533 |
| SARS2 | -1.02147 | 8.427385 | -3.9061 | 0.00022 | 0.000545 | -0.05237 |
| TMEM37 | -0.89817 | 8.468346 | -3.90055 | 0.000224 | 0.000555 | -0.0701 |
| ARL3 | 1.175705 | 7.903054 | 3.898368 | 0.000226 | 0.000558 | -0.07708 |
| GMDS | -0.82081 | 8.290439 | -3.89473 | 0.000228 | 0.000565 | -0.0887 |
| SCARNA7 | -1.09251 | 8.796165 | -3.89444 | 0.000229 | 0.000565 | -0.08963 |
| C21orf69 | 0.784053 | 7.858771 | 3.894065 | 0.000229 | 0.000566 | -0.09082 |
| UHRF1 | 0.915128 | 8.628943 | 3.893633 | 0.000229 | 0.000567 | -0.0922 |
| STK40 | -0.74911 | 7.757279 | -3.89304 | 0.00023 | 0.000568 | -0.09408 |
| RNMTL1 | -1.31514 | 9.036129 | -3.8926 | 0.00023 | 0.000568 | -0.09548 |
| HIST2H3A | -0.90441 | 8.102248 | -3.89008 | 0.000232 | 0.000572 | -0.10352 |
| LCMT2 | 0.975925 | 8.214434 | 3.889693 | 0.000232 | 0.000573 | -0.10476 |
| CGN | -1.01325 | 8.542587 | -3.88677 | 0.000235 | 0.000578 | -0.11408 |
| C9orf164 | 0.712329 | 7.184723 | 3.883194 | 0.000237 | 0.000584 | -0.12548 |
| ADAM33 | 0.779117 | 7.382253 | 3.882968 | 0.000238 | 0.000584 | -0.1262 |
| CXCL16 | -1.0617 | 8.889146 | -3.8827 | 0.000238 | 0.000585 | -0.12705 |
| FURIN | -0.93292 | 9.885113 | -3.87964 | 0.00024 | 0.00059 | -0.13679 |

| | | | | | | |
|---|---|---|---|---|---|---|
| CST1 | 0.913145 | 7.501587 | 3.874768 | 0.000244 | 0.000599 | -0.15231 |
| DNHD1 | 0.725033 | 7.729614 | 3.871906 | 0.000247 | 0.000605 | -0.16141 |
| NBPF1 | 0.988971 | 9.480523 | 3.870293 | 0.000248 | 0.000608 | -0.16654 |
| PARVG | 0.834773 | 10.47525 | 3.867007 | 0.000251 | 0.000614 | -0.17698 |
| MIR99B | 0.772958 | 7.135144 | 3.866192 | 0.000251 | 0.000615 | -0.17957 |
| MIR138-2 | 0.769659 | 6.900577 | 3.864631 | 0.000253 | 0.000618 | -0.18453 |
| FAM101B | 0.975159 | 8.207828 | 3.863271 | 0.000254 | 0.00062 | -0.18884 |
| TRIM28 | -0.82985 | 9.198907 | -3.86221 | 0.000255 | 0.000622 | -0.1922 |
| KRTAP10- | 0.887032 | 7.368482 | 3.86021 | 0.000256 | 0.000626 | -0.19856 |
| PNPLA2 | -0.87776 | 9.485339 | -3.8584 | 0.000258 | 0.000629 | -0.20431 |
| IDH3A | 0.860289 | 9.289237 | 3.855327 | 0.000261 | 0.000635 | -0.21405 |
| UTF1 | 1.102223 | 7.202751 | 3.85086 | 0.000265 | 0.000644 | -0.22822 |
| PIP4K2A | 0.716552 | 8.465945 | 3.85038 | 0.000265 | 0.000645 | -0.22974 |
| C16orf71 | 0.729415 | 6.969211 | 3.848724 | 0.000266 | 0.000648 | -0.23498 |
| IGFBP1 | -1.276 | 8.827893 | -3.83975 | 0.000275 | 0.000666 | -0.26338 |
| SRP14 | -0.70011 | 9.395464 | -3.83414 | 0.00028 | 0.000678 | -0.28112 |
| SNORA46 | -0.99097 | 9.580565 | -3.83074 | 0.000283 | 0.000685 | -0.29187 |
| SFRP5 | 1.083158 | 7.760129 | 3.830654 | 0.000283 | 0.000685 | -0.29214 |
| GLI2 | 0.997081 | 7.759595 | 3.828485 | 0.000285 | 0.000689 | -0.29899 |
| SLC22A18 | -0.73905 | 8.841635 | -3.82647 | 0.000287 | 0.000694 | -0.30534 |
| SCARNA1 | -1.06366 | 8.82647 | -3.81439 | 0.000299 | 0.00072 | -0.34345 |
| PIGZ | 0.866953 | 8.308872 | 3.814285 | 0.000299 | 0.00072 | -0.34377 |
| FAM38A | -1.34742 | 10.4887 | -3.81258 | 0.000301 | 0.000724 | -0.34912 |
| HOXB13 | 0.730489 | 7.048283 | 3.809989 | 0.000303 | 0.00073 | -0.35729 |
| ARSE | -0.84035 | 7.576206 | -3.80628 | 0.000307 | 0.000738 | -0.36895 |
| SHD | -0.72521 | 8.043397 | -3.80624 | 0.000307 | 0.000738 | -0.36909 |
| LUM | -0.8382 | 7.673801 | -3.80589 | 0.000307 | 0.000738 | -0.37019 |
| RNU1F1 | -0.89484 | 9.509063 | -3.79969 | 0.000314 | 0.000753 | -0.38969 |
| F11 | -0.75111 | 8.598665 | -3.79815 | 0.000315 | 0.000756 | -0.39451 |
| ANG | -1.00612 | 9.912404 | -3.79298 | 0.000321 | 0.000768 | -0.41075 |
| SNORA7A | -0.78105 | 7.819359 | -3.78703 | 0.000327 | 0.000782 | -0.42942 |
| LOC7296 | 0.704137 | 7.210078 | 3.785197 | 0.000329 | 0.000787 | -0.43516 |
| PET112L | -0.99577 | 8.876782 | -3.78237 | 0.000332 | 0.000794 | -0.44402 |
| C22orf29 | 0.941926 | 7.853499 | 3.782143 | 0.000333 | 0.000794 | -0.44473 |
| ODF4 | 0.98894 | 7.369443 | 3.777424 | 0.000338 | 0.000806 | -0.4595 |
| UTRN | -0.71369 | 8.126904 | -3.77491 | 0.000341 | 0.000812 | -0.46737 |
| PCDHA7 | 0.72446 | 7.013387 | 3.774835 | 0.000341 | 0.000812 | -0.46761 |
| MBL2 | -0.87699 | 7.481785 | -3.77323 | 0.000342 | 0.000816 | -0.47262 |
| VAMP2 | -0.72059 | 7.632186 | -3.77206 | 0.000344 | 0.000818 | -0.47628 |
| CLDN23 | -0.99469 | 7.79708 | -3.76861 | 0.000348 | 0.000827 | -0.48706 |
| EID2B | 0.864734 | 7.922532 | 3.76647 | 0.00035 | 0.000832 | -0.49376 |
| GPR98 | 0.731679 | 8.385478 | 3.765499 | 0.000351 | 0.000835 | -0.49679 |
| EFHD2 | -0.97569 | 10.30531 | -3.76504 | 0.000352 | 0.000836 | -0.49822 |
| C19orf51 | 0.814461 | 7.475542 | 3.763952 | 0.000353 | 0.000838 | -0.50163 |
| CALML3 | 1.466185 | 7.83859 | 3.762895 | 0.000354 | 0.000841 | -0.50493 |
| CHRDL2 | 1.008953 | 8.409009 | 3.760908 | 0.000357 | 0.000846 | -0.51113 |
| ATF6 | -0.72302 | 8.085294 | -3.75841 | 0.00036 | 0.000852 | -0.51893 |
| FAM84B | -0.89712 | 8.537944 | -3.75834 | 0.00036 | 0.000852 | -0.51915 |
| SERINC2 | -0.86501 | 9.172704 | -3.75644 | 0.000362 | 0.000857 | -0.52507 |
| RUNX2 | 1.069395 | 8.162079 | 3.754526 | 0.000364 | 0.000862 | -0.53104 |
| EVPL | 1.099099 | 8.326123 | 3.750173 | 0.00037 | 0.000874 | -0.5446 |
| TCEAL3 | 0.710205 | 7.967392 | 3.749735 | 0.00037 | 0.000875 | -0.54597 |
| GALNTL4 | 0.873572 | 7.842532 | 3.748542 | 0.000372 | 0.000878 | -0.54968 |
| NDUFA3 | -1.67745 | 10.37169 | -3.7416 | 0.00038 | 0.000895 | -0.57129 |
| SECISBP2 | -0.74473 | 9.145698 | -3.73578 | 0.000388 | 0.000912 | -0.58938 |
| TMEM102 | 1.019186 | 7.553528 | 3.735236 | 0.000388 | 0.000913 | -0.59108 |
| RPL10L | 0.783683 | 7.847665 | 3.733107 | 0.000391 | 0.000919 | -0.59769 |
| FCGBP | -0.70659 | 7.796628 | -3.73269 | 0.000391 | 0.00092 | -0.59898 |

| | | | | | | |
|---|---|---|---|---|---|---|
| NUDT4P1 | 1.060475 | 9.479223 | 3.724373 | 0.000402 | 0.000943 | -0.6248 |
| VAX2 | 0.716044 | 7.32219 | 3.718817 | 0.00041 | 0.000958 | -0.64202 |
| FABP1 | -0.99814 | 7.578772 | -3.71355 | 0.000417 | 0.000974 | -0.65835 |
| ZFP64 | 0.737987 | 8.076025 | 3.70495 | 0.000429 | 0.000999 | -0.68493 |
| WDR72 | -0.77837 | 7.684016 | -3.70177 | 0.000433 | 0.001009 | -0.69477 |
| RAB7L1 | -0.77049 | 9.320793 | -3.68284 | 0.000461 | 0.001068 | -0.75315 |
| MGP | -0.82841 | 8.53961 | -3.68108 | 0.000464 | 0.001073 | -0.75856 |
| GPC3 | -0.86074 | 7.570577 | -3.67911 | 0.000467 | 0.001079 | -0.76461 |
| PNPLA7 | -0.81618 | 8.867635 | -3.67772 | 0.000469 | 0.001083 | -0.76888 |
| TMEM120 | -0.84631 | 9.12759 | -3.67613 | 0.000471 | 0.001088 | -0.77377 |
| FAM19A5 | 1.01925 | 7.831291 | 3.674166 | 0.000474 | 0.001095 | -0.77981 |
| SETBP1 | 0.841974 | 8.211291 | 3.67393 | 0.000474 | 0.001096 | -0.78054 |
| TMEM219 | -1.09235 | 10.20348 | -3.67354 | 0.000475 | 0.001097 | -0.78174 |
| SPINK1 | -1.20709 | 7.605406 | -3.66995 | 0.000481 | 0.001109 | -0.79278 |
| MFSD10 | -1.29024 | 9.373713 | -3.66862 | 0.000483 | 0.001113 | -0.79685 |
| EPHA1 | 0.713673 | 8.452367 | 3.664513 | 0.000489 | 0.001127 | -0.80946 |
| CHAF1A | -0.78911 | 8.837931 | -3.66325 | 0.000491 | 0.001131 | -0.81335 |
| LOC72832 | 0.804382 | 8.571764 | 3.659873 | 0.000497 | 0.001142 | -0.82369 |
| SLC8A1 | 0.784269 | 8.036105 | 3.651989 | 0.00051 | 0.00117 | -0.84784 |
| MACROD1 | -0.77226 | 7.379511 | -3.64982 | 0.000513 | 0.001177 | -0.8545 |
| SMCR7L | -0.91628 | 8.714231 | -3.63776 | 0.000534 | 0.00122 | -0.89135 |
| OSTalpha | -0.8205 | 8.12123 | -3.63512 | 0.000538 | 0.00123 | -0.89941 |
| KRTAP13- | 0.726631 | 6.946304 | 3.632704 | 0.000542 | 0.001238 | -0.90678 |
| LOC38979 | 0.794776 | 6.996697 | 3.631675 | 0.000544 | 0.001242 | -0.90992 |
| PRNP | -0.82624 | 8.996474 | -3.62431 | 0.000557 | 0.001268 | -0.93237 |
| HOM-TES | -0.8666 | 8.105351 | -3.61686 | 0.000571 | 0.001296 | -0.95503 |
| CD19 | 0.715997 | 7.319598 | 3.615784 | 0.000573 | 0.0013 | -0.95832 |
| KCNJ3 | 0.739071 | 7.590215 | 3.611251 | 0.000581 | 0.001318 | -0.9721 |
| PABPC1 | -0.95129 | 9.565201 | -3.60995 | 0.000584 | 0.001323 | -0.97606 |
| NBLA0030 | 0.782281 | 7.682483 | 3.609632 | 0.000584 | 0.001324 | -0.97701 |
| FIG4 | 0.703004 | 8.128581 | 3.606132 | 0.000591 | 0.001339 | -0.98764 |
| ADA | 0.734202 | 7.84485 | 3.605974 | 0.000591 | 0.001339 | -0.98812 |
| SNORD53 | -0.91966 | 9.916757 | -3.60532 | 0.000593 | 0.001341 | -0.99011 |
| WASH3P | -0.97334 | 7.634505 | -3.60112 | 0.000601 | 0.001358 | -1.00285 |
| MYO7A | 0.814604 | 8.69745 | 3.600776 | 0.000601 | 0.001359 | -1.00389 |
| GALNTL6 | 0.865151 | 7.154042 | 3.597441 | 0.000608 | 0.001373 | -1.014 |
| POR | -0.99012 | 10.21202 | -3.59684 | 0.000609 | 0.001375 | -1.01583 |
| COL8A1 | 0.739313 | 7.873477 | 3.593134 | 0.000616 | 0.00139 | -1.02705 |
| SNORA64 | -1.33808 | 10.39637 | -3.58303 | 0.000637 | 0.001431 | -1.05762 |
| FLJ21986 | -0.85103 | 8.23582 | -3.58298 | 0.000637 | 0.001432 | -1.05777 |
| CYP3A7 | -0.95484 | 7.673257 | -3.58274 | 0.000637 | 0.001432 | -1.05848 |
| BATF3 | 0.813729 | 7.654126 | 3.568816 | 0.000666 | 0.001493 | -1.10052 |
| SNORD10 | -1.0096 | 11.7496 | -3.56794 | 0.000668 | 0.001497 | -1.10318 |
| CLK2 | -0.78134 | 8.17872 | -3.56777 | 0.000669 | 0.001498 | -1.10367 |
| RGPD6 | 0.794086 | 8.249805 | 3.564278 | 0.000676 | 0.001512 | -1.11419 |
| UNC5A | 0.757579 | 7.240324 | 3.56028 | 0.000685 | 0.00153 | -1.12623 |
| ITGB2 | -0.71684 | 8.717516 | -3.55397 | 0.000699 | 0.001558 | -1.14522 |
| SNORD2 | -1.30104 | 8.823082 | -3.55023 | 0.000707 | 0.001575 | -1.15645 |
| SNORD63 | -1.06999 | 8.27062 | -3.54843 | 0.000711 | 0.001584 | -1.16185 |
| LIF | 0.98182 | 8.506827 | 3.545441 | 0.000718 | 0.001598 | -1.17083 |
| G0S2 | -0.77021 | 8.050581 | -3.54193 | 0.000726 | 0.001613 | -1.18134 |
| ZNF600 | 0.773479 | 8.35104 | 3.538503 | 0.000734 | 0.00163 | -1.19163 |
| LMOD1 | 0.851183 | 8.322407 | 3.537645 | 0.000736 | 0.001633 | -1.1942 |
| ALDH3A1 | -1.27432 | 7.849514 | -3.53435 | 0.000744 | 0.001649 | -1.20407 |
| WNT10A | 0.811192 | 7.217819 | 3.533152 | 0.000747 | 0.001655 | -1.20766 |
| GLCCI1 | 0.731615 | 7.536482 | 3.528721 | 0.000757 | 0.001677 | -1.22092 |
| NPW | -1.00369 | 9.888087 | -3.51436 | 0.000793 | 0.001749 | -1.2638 |
| FLJ10986 | -1.13263 | 9.398255 | -3.49697 | 0.000838 | 0.001839 | -1.3156 |

| | | | | | | |
|---|---|---|---|---|---|---|
| RPS19BP1 | -0.75712 | 7.912516 | -3.4908 | 0.000854 | 0.001872 | -1.33393 |
| PNCK | 0.967577 | 7.558548 | 3.48388 | 0.000873 | 0.00191 | -1.35446 |
| ANKRD47 | 0.95441 | 8.28897 | 3.479748 | 0.000885 | 0.001934 | -1.36671 |
| GLIS2 | 0.87401 | 9.314751 | 3.474461 | 0.0009 | 0.001965 | -1.38236 |
| HIST1H4C | -0.79045 | 9.3663 | -3.47329 | 0.000903 | 0.001971 | -1.38583 |
| ROBO1 | -0.75896 | 8.396825 | -3.46598 | 0.000924 | 0.002011 | -1.40743 |
| LRRC14 | -0.80391 | 8.964963 | -3.46392 | 0.00093 | 0.002023 | -1.41354 |
| BHLHB3 | 0.894185 | 8.032188 | 3.463151 | 0.000932 | 0.002027 | -1.41579 |
| NSMCE4A | -0.79792 | 7.92828 | -3.45588 | 0.000954 | 0.002071 | -1.43725 |
| LOC2859( | 0.964418 | 9.018563 | 3.453133 | 0.000962 | 0.002087 | -1.44535 |
| CLDN9 | 0.954811 | 8.142774 | 3.445352 | 0.000986 | 0.002133 | -1.46826 |
| ZNF48 | 0.704394 | 7.666972 | 3.443573 | 0.000991 | 0.002143 | -1.47349 |
| CCL19 | -1.09566 | 7.972348 | -3.41989 | 0.001068 | 0.002292 | -1.54297 |
| GATAD2A | -0.74314 | 9.08469 | -3.40616 | 0.001115 | 0.002383 | -1.58311 |
| RNU1G2 | -0.97657 | 11.97402 | -3.40533 | 0.001118 | 0.002388 | -1.58552 |
| EEF1A2 | -1.34775 | 8.120405 | -3.40307 | 0.001125 | 0.002404 | -1.59212 |
| DNAJC9 | 0.704526 | 8.432242 | 3.40299 | 0.001126 | 0.002404 | -1.59236 |
| IPPK | 0.750491 | 7.02295 | 3.38789 | 0.00118 | 0.002511 | -1.63633 |
| NAGS | -0.77822 | 7.872526 | -3.38729 | 0.001182 | 0.002515 | -1.63807 |
| NRXN2 | 0.79079 | 8.299147 | 3.38433 | 0.001193 | 0.002536 | -1.64668 |
| SFRS9 | -0.80277 | 8.570552 | -3.37831 | 0.001216 | 0.00258 | -1.66415 |
| SCG5 | -0.72773 | 7.974465 | -3.36649 | 0.001261 | 0.00267 | -1.69841 |
| HIST1H2B | -0.78638 | 8.453236 | -3.36453 | 0.001269 | 0.002685 | -1.70409 |
| PXMP2 | -0.86065 | 9.538159 | -3.3601 | 0.001286 | 0.00272 | -1.71689 |
| GLTPD1 | -0.70685 | 7.687291 | -3.35592 | 0.001303 | 0.002755 | -1.72897 |
| KIF1A | 0.793129 | 7.69132 | 3.339178 | 0.001372 | 0.002888 | -1.77725 |
| MYPOP | -0.81377 | 8.042927 | -3.33469 | 0.001391 | 0.002925 | -1.79016 |
| LELP1 | 0.717597 | 7.280102 | 3.331547 | 0.001405 | 0.00295 | -1.79919 |
| B3GNT7 | 0.868461 | 7.752059 | 3.32847 | 0.001418 | 0.002977 | -1.80803 |
| LGR5 | 1.02248 | 8.219123 | 3.32816 | 0.001419 | 0.002979 | -1.80892 |
| C20orf43 | -0.90291 | 8.783222 | -3.32595 | 0.001429 | 0.002997 | -1.81528 |
| ZNF467 | -0.90195 | 9.567472 | -3.31683 | 0.00147 | 0.003074 | -1.84141 |
| PLA2G4C | -0.70783 | 7.801496 | -3.3123 | 0.00149 | 0.003113 | -1.85437 |
| CSAG1 | 0.74653 | 7.284593 | 3.306943 | 0.001515 | 0.00316 | -1.8697 |
| RENBP | -0.88916 | 8.066644 | -3.30233 | 0.001537 | 0.003202 | -1.88287 |
| SEMA6A | 0.726527 | 7.657057 | 3.293072 | 0.001581 | 0.003286 | -1.90928 |
| UGT2B7 | -0.78713 | 10.57642 | -3.2877 | 0.001607 | 0.003335 | -1.92459 |
| VIPR1 | -1.17101 | 9.723694 | -3.28421 | 0.001624 | 0.003366 | -1.93451 |
| ANXA5 | -0.78576 | 8.034199 | -3.28011 | 0.001645 | 0.003404 | -1.94617 |
| CCNI | -0.93778 | 8.640306 | -3.27068 | 0.001693 | 0.003493 | -1.97292 |
| CYP3A4 | -0.87473 | 7.557512 | -3.26341 | 0.001731 | 0.003562 | -1.99354 |
| C9orf16 | -0.75875 | 8.160007 | -3.24455 | 0.001833 | 0.003753 | -2.0468 |
| DTNB | -0.7952 | 7.736869 | -3.23928 | 0.001862 | 0.003808 | -2.06164 |
| COMMD3 | -0.74062 | 7.982589 | -3.22186 | 0.001963 | 0.003991 | -2.11061 |
| FGD3 | -0.78058 | 8.504582 | -3.22123 | 0.001967 | 0.003998 | -2.11236 |
| SLC5A2 | 0.780027 | 7.314369 | 3.219183 | 0.001979 | 0.004021 | -2.11811 |
| ICAM2 | 0.824576 | 8.338322 | 3.215213 | 0.002003 | 0.004064 | -2.12923 |
| ADORA2A | 0.817629 | 7.981586 | 3.204471 | 0.002068 | 0.004186 | -2.15927 |
| GNB2 | -0.70627 | 7.793165 | -3.19763 | 0.002111 | 0.004264 | -2.17838 |
| C2orf82 | -0.91418 | 8.593601 | -3.1968 | 0.002117 | 0.004273 | -2.1807 |
| HLA-DRB! | -0.77111 | 7.103102 | -3.19612 | 0.002121 | 0.004279 | -2.18259 |
| FGF22 | 0.81477 | 7.17562 | 3.19218 | 0.002146 | 0.004326 | -2.19356 |
| STOX2 | 0.918766 | 10.01164 | 3.183566 | 0.002202 | 0.004429 | -2.21753 |
| ONECUT2 | -0.77853 | 8.921689 | -3.16616 | 0.00232 | 0.004647 | -2.26582 |
| PSCD4 | 0.830509 | 8.974399 | 3.159752 | 0.002365 | 0.004726 | -2.28356 |
| NAT8B | -0.70548 | 7.703837 | -3.14212 | 0.002493 | 0.004955 | -2.33222 |
| IFITM4P | 0.95501 | 7.655918 | 3.139578 | 0.002512 | 0.00499 | -2.3392 |
| ZNF34 | 0.707693 | 7.779322 | 3.135733 | 0.00254 | 0.005045 | -2.34978 |

| | | | | | | |
|---|---|---|---|---|---|---|
| SLC17A4 | -0.83942 | 8.84437 | -3.13116 | 0.002575 | 0.005106 | -2.36235 |
| ANXA8L2 | 0.831224 | 7.967653 | 3.127792 | 0.002601 | 0.005149 | -2.37159 |
| SAE1 | -1.17104 | 9.120281 | -3.12316 | 0.002637 | 0.005214 | -2.38428 |
| FOXN2 | -0.74062 | 8.300422 | -3.11892 | 0.00267 | 0.005269 | -2.3959 |
| SPP1 | -0.91646 | 8.697572 | -3.10359 | 0.002794 | 0.005496 | -2.43782 |
| ERRFI1 | -0.89837 | 8.748957 | -3.1033 | 0.002796 | 0.005499 | -2.4386 |
| NDUFA12 | -0.80964 | 10.77103 | -3.1011 | 0.002815 | 0.005529 | -2.44459 |
| SERPINE2 | -1.23405 | 8.870304 | -3.09966 | 0.002827 | 0.005551 | -2.44851 |
| GSTM1 | -0.87849 | 7.835806 | -3.08683 | 0.002935 | 0.005747 | -2.48343 |
| TMED3 | -0.83601 | 8.739519 | -3.08596 | 0.002943 | 0.00576 | -2.4858 |
| NEU4 | -1.06582 | 9.018533 | -3.08383 | 0.002962 | 0.005791 | -2.4916 |
| IIP45 | 0.792848 | 7.614354 | 3.078557 | 0.003008 | 0.005874 | -2.50589 |
| NCRNA00 | 0.717824 | 7.290797 | 3.07751 | 0.003017 | 0.005889 | -2.50873 |
| RNU5D | -0.80047 | 7.894167 | -3.07695 | 0.003022 | 0.005898 | -2.51026 |
| CRLS1 | -0.84033 | 8.701108 | -3.0567 | 0.003207 | 0.006224 | -2.56502 |
| SNORD43 | -1.02741 | 8.731777 | -3.05538 | 0.003219 | 0.006244 | -2.56857 |
| RPS6KL1 | 0.809217 | 7.968476 | 3.049069 | 0.003279 | 0.006347 | -2.58557 |
| FLJ36070 | 0.837422 | 8.441802 | 3.034956 | 0.003417 | 0.006589 | -2.62349 |
| LOC6538C | 0.897737 | 8.517062 | 3.024352 | 0.003524 | 0.006779 | -2.6519 |
| NECAB2 | -0.72553 | 7.826374 | -3.01314 | 0.00364 | 0.006987 | -2.68187 |
| POU2AF1 | 0.74073 | 7.878955 | 3.005142 | 0.003726 | 0.007136 | -2.70317 |
| SNORA5C | -1.05979 | 9.063054 | -3.00467 | 0.003731 | 0.007145 | -2.70442 |
| AKT2 | -0.71901 | 9.053089 | -2.96875 | 0.004138 | 0.007839 | -2.79962 |
| PGF | 0.775974 | 8.586553 | 2.954717 | 0.004308 | 0.008136 | -2.83657 |
| CLIP3 | -0.79438 | 8.810927 | -2.93658 | 0.004537 | 0.008533 | -2.88413 |
| HPDL | 0.959416 | 7.678303 | 2.909397 | 0.004902 | 0.009153 | -2.95499 |
| CYP2B7P1 | 0.822319 | 8.368435 | 2.907141 | 0.004933 | 0.009204 | -2.96084 |
| C19orf47 | 0.810028 | 9.080404 | 2.898005 | 0.005063 | 0.009424 | -2.98453 |
| C9 | -0.76041 | 7.845718 | -2.87804 | 0.005356 | 0.009914 | -3.03608 |
| SNORD3C | -1.2768 | 8.271541 | -2.87295 | 0.005434 | 0.010044 | -3.04919 |
| SNORD30 | -1.15378 | 9.865838 | -2.86946 | 0.005487 | 0.010133 | -3.05815 |
| Magmas | -1.05704 | 9.122799 | -2.85745 | 0.005675 | 0.010447 | -3.08896 |
| RAMP1 | -0.75238 | 8.395164 | -2.84035 | 0.005954 | 0.010911 | -3.13267 |
| RAB6B | 0.72219 | 7.256012 | 2.822868 | 0.006251 | 0.011411 | -3.17711 |
| SLC2A6 | -0.7877 | 9.012948 | -2.82193 | 0.006268 | 0.01144 | -3.1795 |
| AP2M1 | -0.72002 | 9.362362 | -2.81944 | 0.006311 | 0.011512 | -3.18579 |
| ATOH8 | 0.719611 | 7.959843 | 2.818778 | 0.006323 | 0.01153 | -3.18748 |
| TTC36 | 0.905562 | 8.220226 | 2.815705 | 0.006377 | 0.011617 | -3.19526 |
| SHBG | -0.75745 | 9.021603 | -2.80973 | 0.006484 | 0.0118 | -3.21037 |
| IRGQ | 0.842368 | 8.938594 | 2.79464 | 0.006761 | 0.012259 | -3.24844 |
| IGFBP2 | -0.82952 | 8.50886 | -2.78749 | 0.006896 | 0.012473 | -3.26642 |
| H19 | -1.00032 | 8.753451 | -2.76435 | 0.007349 | 0.013228 | -3.32434 |
| C14orf133 | 0.713699 | 9.369655 | 2.75214 | 0.0076 | 0.013644 | -3.35475 |
| XAGE1A | 1.156608 | 8.510656 | 2.745831 | 0.007732 | 0.013865 | -3.37042 |
| GNG10 | 0.707006 | 8.954184 | 2.740328 | 0.007849 | 0.014058 | -3.38407 |
| RABAC1 | -0.7002 | 8.609131 | -2.73994 | 0.007858 | 0.014069 | -3.38502 |
| NOXA1 | 0.726255 | 8.459287 | 2.704334 | 0.008657 | 0.015358 | -3.47279 |
| HNRNPL | -0.98354 | 8.686643 | -2.68598 | 0.009098 | 0.016068 | -3.51767 |
| ZNF668 | 0.875233 | 8.862661 | 2.651485 | 0.009981 | 0.017463 | -3.60134 |
| MGST2 | -0.73652 | 9.712077 | -2.65 | 0.010021 | 0.017531 | -3.60491 |
| FAM46A | 0.80759 | 9.634162 | 2.543068 | 0.01329 | 0.022666 | -3.85859 |
| TRIB1 | -0.75437 | 9.342632 | -2.5354 | 0.013558 | 0.023079 | -3.87645 |
| LOC6449C | 0.781596 | 10.0565 | 2.498136 | 0.014931 | 0.025188 | -3.96261 |
| RSPRY1 | 0.805705 | 9.429461 | 2.465243 | 0.016246 | 0.027169 | -4.03779 |
| CITED4 | -0.7061 | 9.931331 | -2.44525 | 0.017095 | 0.028446 | -4.08308 |
| ACTG2 | -0.71051 | 8.482373 | -2.44241 | 0.017218 | 0.028634 | -4.08948 |
| CDA | 0.740892 | 9.249691 | 2.424054 | 0.018038 | 0.029877 | -4.13076 |
| MRPL21 | -0.75975 | 8.666433 | -2.35919 | 0.021221 | 0.034636 | -4.27449 |

| | | | | | | |
|---|---|---|---|---|---|---|
| RXRA | -0.74204 | 9.836244 | -2.32223 | 0.02325 | 0.037628 | -4.35492 |
| MT1X | -0.9159 | 10.04525 | -2.29875 | 0.024627 | 0.039599 | -4.40544 |

supplementary Table 2

| ONTOLOGY | ID | Description of term | GeneRatio | BgRatio | pvalue | p.adjust | qvalue |
|---|---|---|---|---|---|---|---|
| BP | GO:0016999 | antibiotic metabolic process | 62/3673 | 151/18670 | 1.196E-09 | 6.33483E-07 | 5.49637E-07 |
| BP | GO:0046034 | ATP metabolic process | 89/3673 | 305/18670 | 3.87271E-05 | 0.002675535 | 0.002321409 |
| BP | GO:0016101 | diterpenoid metabolic process | 44/3673 | 110/18670 | 6.98063E-07 | 0.000113766 | 9.87085E-05 |
| BP | GO:0006631 | fatty acid metabolic process | 115/3673 | 383/18670 | 6.68861E-07 | 0.000113766 | 9.87085E-05 |
| BP | GO:0042445 | hormone metabolic process | 73/3673 | 232/18670 | 1.21286E-05 | 0.001117241 | 0.000969366 |
| BP | GO:0042743 | hydrogen peroxide metabolic | 25/3673 | 57/18670 | 2.73309E-05 | 0.002052295 | 0.001780658 |
| BP | GO:0006720 | isoprenoid metabolic process | 52/3673 | 139/18670 | 8.5132E-07 | 0.000131975 | 0.000114507 |
| BP | GO:0001819 | positive regulation of cytokine production | 117/3673 | 464/18670 | 0.001846801 | 0.043963541 | 0.038144644 |
| BP | GO:0009167 | purine ribonucleoside monophosphate metabolic process | 97/3673 | 340/18670 | 4.72518E-05 | 0.003195025 | 0.00277214 |
| BP | GO:0036293 | response to decreased oxygen levels | 109/3673 | 370/18670 | 3.45939E-06 | 0.000429552 | 0.000372698 |

| | | | | | | | |
|---|---|---|---|---|---|---|---|
| BP | GO:0001666 | response to hypoxia | 106/3673 | 359/18670 | 4.18986E-06 | 0.000484195 | 0.000420108 |
| BP | GO:0070482 | response to oxygen levels | 118/3673 | 394/18670 | 5.55748E-07 | 9.81204E-05 | 8.51334E-05 |
| BP | GO:0001523 | retinoid metabolic process | 42/3673 | 104/18670 | 9.16384E-07 | 0.000138679 | 0.000120324 |
| BP | GO:0006721 | terpenoid metabolic process | 48/3673 | 120/18670 | 2.21993E-07 | 4.86548E-05 | 4.2215E-05 |
| BP | GO:0006805 | xenobiotic metabolic process | 51/3673 | 125/18670 | 4.39706E-08 | 1.21512E-05 | 1.05429E-05 |

| geneID |
| --- |
| ACLY/ACO1/ACO2/ADH1A/ADH1B/ADH1C/ALDH1A1/ALDH2/CAT/AKR1C4/ABCC2/CYBA/CYP1A2/CYP4B1/AKR1C2/DLST/EGFR/FH/GPX3/HBA2/HBB/HBG1/HBG2/HBQ1/HP/IDH1/IDH2/IDH3A/IDH3G/LPO/MDH1/MDH2/PRDX1/PCK2/RENBP/SDHA/SDHB/ST6GAL1/SOD1/STAR/STAT3/SULT2A1/ALDH5A1/AKR7A2/AKR1C3/SUCLG1/PRDX6/HDAC6/AKR1A1/NOXA1/FTCD/PRDX3/PARK7/PRDX5/NOX1/DUOX1/UGT1A1/ACSS2/AKR1B10/DUOXA1/AMDHD1/MPV17L |
| ADCYAP1/AK2/ALDOA/ALDOB/APP/RHOA/ATP1B1/ATP6V1A/ATP6V1B1/TSPO/CBFA2T3/COX4I1/COX5B/COX6C/COX7A2/COX7C/COX8A/COX15/CYC1/GALK1/GAPDH/GCK/GPI/GUK1/HIF1A/HSPA1A/HSPA1B/HSPA8/HTR2A/LDHA/MYH7/NDUFA1/NDUFA2/NDUFA3/NDUFA4/NDUFA8/NDUFA10/NDUFAB1/NDUFB5/NDUFB6/NDUFB7/NDUFB8/NDUFB10/NDUFC1/NDUFS2/NDUFS3/NDUFV1/NDUFS5/NDUFS8/NDUFV2/PFKFB1/PFKFB3/PFKL/PGM1/SDHA/SEC13/SHMT2/SLC4A1/STAT3/SURF1/UQCRC1/UQCRFS1/UQCRH/OGT/SLC25A12/NUP93/SLC25A13/NUDT5/SLC2A6/TREX1/PARK7/ISCU/ZBTB20/NUPR1/UQCRQ/DNAJC15/STOML2/MLXIPL/NDUFB11/DDIT4/NUP133/NDUFA12/S |
| ADH1A/ADH1B/ADH1C/ALDH1A1/APOA1/APOA2/APOB/APOC2/APOC3/APOE/AKR1C4/CLPS/CYP1A2/CYP3A7/CYP2C8/CYP3A4/CYP3A5/EGFR/GPC3/HSPG2/PNLIP/RARRES2/RBP1/RBP4/OPN1LW/RDH5/SDC4/STAR/TTR/RDH16/AKR1C3/DGAT1/LRAT/DHRS3/SDC3/CYP2S1/UGT1A1/UGT1A3/APOM/CYP26B1/AKR1B10/RDH12/PLB1/CYP4V |
| ACAA1/ACACB/ACADVL/ACAT1/ACLY/AKT1/AKT2/ALOX12B/APOC1/APOC2/APOC3/ATP6V1B1/AUH/AVP/BAAT/C3/CBR1/CD74/CES1/AKR1C4/MAPK14/CYP1A2/CYP2C8/CYP2E1/CYP2F1/CYP2J2/CYP3A4/CYP4A11/AKR1C2/DECR1/ECH1/ECHS1/EHHADH/EPHX2/ETFA/ETFB/ETFDH/FABP1/GHSR/GIP/GPX4/GSTA1/GSTM2/HADHA/HSD17B4/IL1B/INSIG1/IRS1/LEP/LIPC/MIF/NDUFAB1/PCCB/PCK2/PEX7/PHYH/PON2/POR/PRKAB2/MAPK3/SCD/SCP2/TH/TNFRSF1A/UCP3/XBP1/ALDH5A1/ACOX2/PLA2G10/GNPAT/DEGS1/PLA2G4C/AKR1C3/CES2/ADIPOQ/QKI/LPIN2/NR1H3/LYPLA1/PTGES3/SLC27A3/ERLIN2/TREX1/SCAP/PTGR1/LPIN1/AMACR/HACL1/DECR2/ANGPTL3/CYP2S1/CRYL1/MLXIPL/MECR/AIG1/ACSL5/ECHDC2/ACSS2/CYP4F11/MID1IP1/ELOVL5/ELOVL6/SCD5/PTGES2/LONP2/ACAD11/THEM4/ACOT12/PLA2G4F/CYP4V |
| ACAA1/ADH1A/ADH1B/ADH1C/ADM/ALDH1A1/ALDH9A1/APOA1/ATP1A1/TSPO/SCARB1/CES1/CHAT/AKR1C4/COMT/CTSZ/CYP1A2/CYP3A7/CYP2C8/CYP3A4/CYP3A5/CYP27A1/AKR1C2/DIO2/ENPEP/FSHB/GATA3/GHR/GNB3/PRLHR/FFAR3/HIF1A/HPN/HSD17B4/IDE/IL1B/LEP/FURIN/PCSK6/POR/KLK6/RBP1/RBP4/RDH5/SAFB/SCG5/SPP1/STAR/TTR/UGT2B7/YWHAH/PAX8/COLQ/RDH16/HSD17B6/AKR1C3/DGAT1/LRAT/DHRS3/ATP6AP2/STUB1/CYP2S1/HSD17B11/DUOX1/UGT1A1/UGT1A3/CYP26B1/ |
| CAT/CYBA/CYP1A2/EGFR/GPX3/HBA2/HBB/HBG1/HBG2/HBQ1/HP/LPO/PRDX1/SOD1/STAT3/PRDX6/HDAC6/NOXA1/PRDX3/PARK7/PRDX5/NOX1/DUOX1/DUOXA1/MPV17 |
| ADH1A/ADH1B/ADH1C/ALDH1A1/APOA1/APOA2/APOB/APOC2/APOC3/APOE/AKR1C4/CLPS/CYP1A2/CYP3A7/CYP2C8/CYP2E1/CYP3A4/CYP3A5/EGFR/FDFT1/GPC3/HMGCS1/HMGCS2/HSPG2/PHYH/PNLIP/RARRES2/RBP1/RBP4/OPN1LW/RDH5/SDC4/STAR/TH/TTR/RDH16/AKR1C3/DGAT1/DPM1/LRAT/DHRS3/SDC3/NPC2/CYP2S1/UGT1A1 |
| ABL1/ADCYAP1/AGT/BIRC2/APOA2/APP/ATF4/AZU1/B2M/C3/C5/CD14/TNFRSF8/CD74/CD81/CEBPB/CCR7/CREB1/ATF2/MAPK14/CSF1R/CYBA/DDT/DDX1/DDX3X/EP300/F2R/FCER1G/XRCC6/GAPDH/GATA3/FFAR3/FFAR2/HDAC2/HIF1A/HLA-DPA1/HLA-E/HMGB1/HSPA1A/HSPA1B/HSPB1/HSPD1/IL1B/IL6/IL6R/IL6ST/IL10/IL18/IRAK1/ISL1/KIR2DL4/LBP/LEP/LUM/LY9/CD46/MIF/ORM1/ORM2/FURIN/PAEP/PDE4B/PIK3R1/SERPINF2/POLR2F/POLR2H/PRKCQ/PRKDC/MAPK3/MAPK13/RARA/RPS3/CCL19/CX3CL1/SOD1/SPTBN1/SRC/STAT1/STAT3/STAT6/THBS1/WNT11/XBP1/TNFRSF14/ARHGEF2/ADIPOQ/PUM1/ATP6AP2/LILRB2/LILRB1/PARK7/LY96/LSM14A/ZBTB20/CLEC4E/NOX1/CRCP/IL23A/POLR3B/GSDMD/AFAP1L2/IL1F10/TSLP/NLR |
| ADA/ADCYAP1/AK2/ALDOA/ALDOB/AMPD1/APP/RHOA/ATIC/ATP1B1/ATP6V1A/ATP6V1B1/TSPO/CBFA2T3/COX4I1/COX5B/COX6C/COX7A2/COX7C/COX8A/COX15/CYC1/GALK1/GAPDH/GCK/GPI/GUK1/HIF1A/HSPA1A/HSPA1B/HSPA8/HTR2A/IMPDH2/LDHA/MYH7/NDUFA1/NDUFA2/NDUFA3/NDUFA4/NDUFA8/NDUFA10/NDUFAB1/NDUFB5/NDUFB6/NDUFB7/NDUFB8/NDUFB10/NDUFC1/NDUFS2/NDUFS3/NDUFV1/NDUFS5/NDUFS8/NDUFV2/PFKFB1/PFKFB3/PFKL/PGM1/SDHA/SEC13/SHMT2/SLC4A1/STAT3/SURF1/UQCRC1/UQCRFS1/UQCRH/OGT/SLC25A12/NUP93/SLC25A13/NUDT5/NUDT3/SLC2A6/TREX1/PARK7/NT5C2/ISCU/ZBTB20/NUPR1/UQCRQ/DNAJC15/STOML2/AK3 |
| ACTN4/ADA/ADM/AKT1/ALDH3A1/ANG/BIRC2/RHOA/ATF4/ATP1B1/ZFP36L1/CA9/CAT/CBFA2T3/CHRNA4/CLDN3/CREB1/CYBA/DPP4/EDNRA/EP300/EPAS1/EPO/ERCC2/ETS1/F7/FABP1/GNB1/HIF1A/IRAK1/ITPR2/KCNMA1/LDHA/LEP/LMNA/LOXL2/SMAD4/MYC/NF1/NFE2L2/NOS1/NOS2/SLC11A2/PGF/PIN1/PLOD1/PSMA1/PSMA5/PSMA6/PSMB1/PSMB3/PSMB4/PSMB7/PSMB10/PSMC2/PSMC3/PSMC5/PSMC6/PSMD4/PTEN/RPS27A/SCN2A/SLC8A1/SRC/SUV39H1/TMBIM6/TERC/TH/THBS1/HSP90B1/UBA52/UBB/UBC/UCP3/USF1/VCAM1/VEGFB/XRCC1/CXCR4/ADAM15/ADIPOQ/PICK1/NAMPT/STUB1/NDRG1/HYOU1/PHB2/SCAP/P2RX2/PSME4/HIGD1A/ANKRD1/B3GAT1 |

ACTN4/ADA/ADM/AKT1/ALDH3A1/ANG/BIRC2/RHOA/ATP1B1/ZFP36L1/CA9/CAT/CBFA2T3/CHRNA4/CLDN3/CREB1/CYBA/DPP4/EDNRA/EP300/EPAS1/EPO/ERCC2/ETS
1/F7/FABP1/GNB1/HIF1A/IRAK1/ITPR2/KCNMA1/LDHA/LEP/LMNA/LOXL2/SMAD4/MYC/NF1/NFE2L2/NOS1/NOS2/SLC11A2/PGF/PIN1/PLOD1/PSMA1/PSMA5/PSMA6/
PSMB1/PSMB3/PSMB4/PSMB7/PSMB10/PSMC2/PSMC3/PSMC5/PSMC6/PSMD4/PTEN/RPS27A/SCN2A/SLC8A1/SRC/SUV39H1/TMBIM6/TERC/TH/THBS1/HSP90B1/UBA52/
UBB/UBC/UCP3/USF1/VCAM1/VEGFB/XRCC1/CXCR4/ADAM15/ADIPOQ/STUB1/NDRG1/HYOU1/PHB2/SCAP/P2RX2/PSME4/HIGD1A/ANKRD1/B3GAT1/HIPK2/UBQLN1/FIS
ACTN4/ADA/ADM/AKT1/ALDH3A1/ANG/BIRC2/RHOA/ATF4/ATP1B1/ATP6V1A/ATP6AP1/ZFP36L1/CA9/CAT/CBFA2T3/CHRNA4/COL1A1/CLDN3/CREB1/CYBA/DPP4/EDN
RA/EP300/EPAS1/EPO/ERCC2/ETS1/F7/FABP1/GNB1/HDAC2/HIF1A/IRAK1/ITPR2/KCNMA1/LDHA/LEP/LMNA/LOXL2/SMAD4/MYC/NF1/NFE2L2/NOS1/NOS2/SLC11A2
/PDGFRB/PGF/PIN1/PLOD1/POU4F2/PSMA1/PSMA5/PSMA6/PSMB1/PSMB3/PSMB4/PSMB7/PSMB10/PSMC2/PSMC3/PSMC5/PSMC6/PSMD4/PTEN/RPS27A/SCN2A/SLC8A1
/SRC/SUV39H1/TMBIM6/TERC/TH/THBS1/HSP90B1/UBA52/UBB/UBC/UCP3/USF1/VCAM1/VEGFB/XRCC1/CXCR4/ADAM15/ADIPOQ/PICK1/ATP6V1G1/NAMPT/STUB1/ND
RG1/HYOU1/ATG7/PHB2/SCAP/P2RX2/PSME4/HIGD1A/NOX1/ANKRD1/B3GAT1/HIPK2/UBQLN1/FIS1/ANGPTL4/ANGPT4/DDIT4/EGLN1/ALKBH5/CPEB1/BRIP1/MYOCD/
ADH1A/ADH1B/ADH1C/ALDH1A1/APOA1/APOA2/APOB/APOC2/APOC3/APOE/AKR1C4/CLPS/CYP1A2/CYP3A7/CYP2C8/CYP3A4/CYP3A5/GPC3/HSPG2/PNLIP/RARRES2/R
BP1/RBP4/OPN1LW/RDH5/SDC4/TTR/RDH16/AKR1C3/DGAT1/LRAT/DHRS3/SDC3/CYP2S1/UGT1A1/UGT1A3/APOM/CYP26B1/AKR1B10/RDH12/PLB1/CYP4V2
ADH1A/ADH1B/ADH1C/ALDH1A1/APOA1/APOA2/APOB/APOC2/APOC3/APOE/AKR1C4/CLPS/CYP1A2/CYP3A7/CYP2C8/CYP2E1/CYP3A4/CYP3A5/EGFR/FDFT1/GPC3/HMG
CS1/HMGCS2/HSPG2/PNLIP/RARRES2/RBP1/RBP4/OPN1LW/RDH5/SDC4/STAR/TTR/RDH16/AKR1C3/DGAT1/LRAT/DHRS3/SDC3/CYP2S1/UGT1A1/UGT1A3/APOM/CYP26
AADAC/ACAA1/ACY1/AHR/ALDH3A1/BAAT/CES1/CYP1A2/CYP3A7/CYP2C8/CYP2E1/CYP2F1/CYP2J2/CYP3A4/CYP3A5/CYB5R3/EPHX1/EPHX2/FMO2/FMO3/GHR/GRIN1
/GSTA1/GSTM1/GSTM2/GSTM5/HNF4A/HSP90AB1/LPO/MGST1/MGST2/POR/STAR/UGT2B15/AKR7A2/CES2/GSTO1/PTGES3/AKR7A3/N6AMT1/CYP2S1/UGT1A6/UGT1A4/

| Count |
| --- |
| 62 |
| 89 |
| 44 |
| 115 |
| 73 |
| 25 |
| 52 |
| 117 |
| 97 |
| 109 |

106

118

42

48

51
